# Direct Stereodivergent Olefination of Carbonyl Compounds with Sulfur Ylides

**DOI:** 10.1021/jacs.2c05637

**Published:** 2022-06-30

**Authors:** Jérémy Merad, Phillip S. Grant, Tobias Stopka, Juliette Sabbatani, Ricardo Meyrelles, Alexander Preinfalk, Ján Matyasovsky, Boris Maryasin, Leticia González, Nuno Maulide

**Affiliations:** † Institute of Organic Chemistry, University of Vienna, 1090 Vienna, Austria; ‡ Institute of Theoretical Chemistry, University of Vienna, 1090 Vienna, Austria; § Doctoral School in Chemistry,University of Vienna, 1090 Vienna, Austria

## Abstract

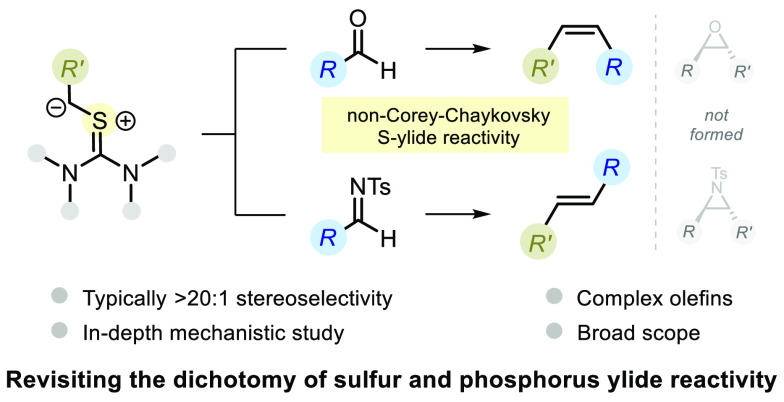

The reactivity of phosphorus and sulfur ylides toward carbonyl compounds constitutes a well-known dichotomy that is a common educational device in organic chemistry—the former gives olefins, while the latter gives epoxides. Herein, we report a stereodivergent carbonyl olefination that challenges this dichotomy, showcasing thiouronium ylides as valuable olefination reagents. With this method, aldehydes are converted to *Z*-alkenes with high stereoselectivity and broad substrate scope, while *N-*tosylimines provide a similarly proficient entry to *E*-alkenes. In-depth computational and experimental studies clarified the mechanistic details of this unusual reactivity.

## Introduction

Alkenes are among the most prevalent functional groups in natural products and industrial chemicals, with one cheminformatics study estimating that 40% of the former contain an alkene.^1^ As such, the development of olefination methods has been a central and rewarding challenge to organic chemistry,^2^ contributing some of the most valued reactions in the “synthetic toolbox”.^3^ Nevertheless, the wide structural and electronic parameters of olefin chemical space continue to pose a challenge, implying that no single method is universally apt for their synthesis. As a result, the development of complementary olefination methods remains an active area of research.

**Figure 1:**
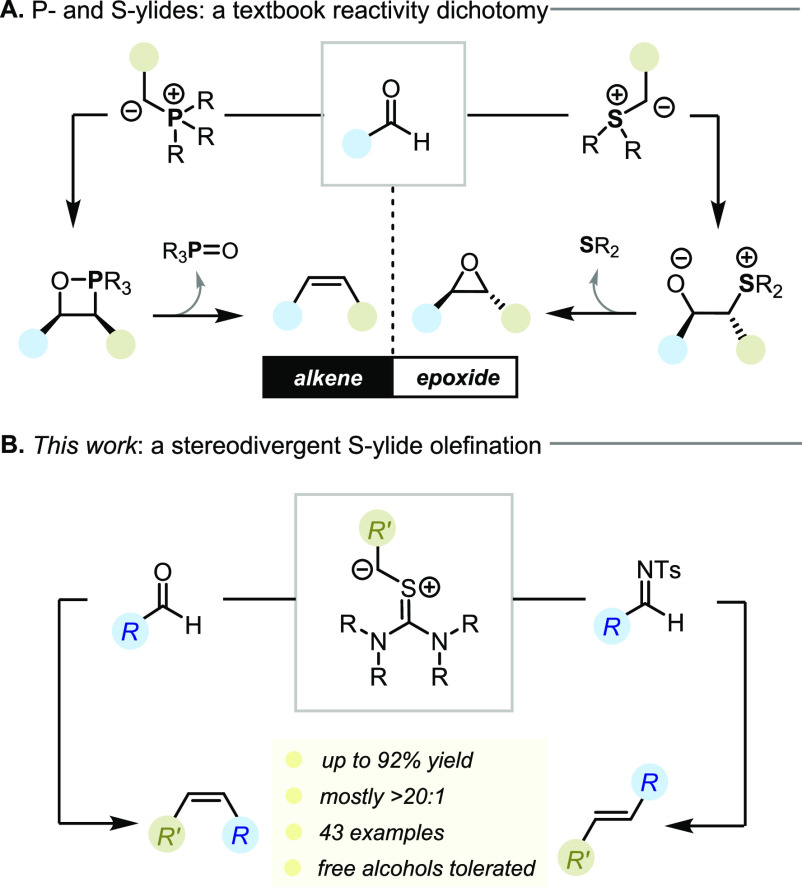
Revisiting the textbook reactivity dichotomy of phosphorus and sulfur ylides with carbonyl compounds.

The Wittig olefination is part of a mechanistic dichotomy that is a common educational device in organic chemistry.^4,5^ It is generally accepted to proceed by the addition of a phosphorus ylide to an aldehyde or ketone to give an oxaphosphetane, which then undergoes cycloreversion to produce an alkene and a phosphine oxide (Figure 1A).^6,7^ The major thermodynamic driving force for this reaction is known to be the strength of the resulting phosphorus–oxygen double-bond.^5^ Notably, the reaction of a sulfur-ylide—the Corey–Chaykovsky reaction—follows a different pathway, involving an intermediate betaine and resulting in the formation of an epoxide by displacement of the sulfonium group (Figure 1A).^8−10^ This textbook difference in reactivity is attributed to the lower oxophilicity of sulfur, the better leaving-group ability of the sulfonium group, and kinetic factors.^5,11,12^ The sulfur–phosphorus ylide dichotomy is therefore commonly used in chemical education to convey the concepts of leaving group ability, oxophilicity, as well as kinetic/thermodynamic reaction control.^4^

Our group’s long-standing interest in novel olefination methods^13,14^ and sulfur ylide reactivity^15^ led us to interrogate the universality of the phosphorus/sulfur ylide dichotomy in organic chemistry. Herein, we report a novel carbonyl olefination method relying on thiouronium ylides, which challenges this dichotomy (Figure 1B). This method selectively affords *Z*-alkenes from aldehydes and *E*-alkenes from *N-*tosylimines, typically in greater that 20:1 selectivity, while exhibiting broad substrate scope, making it suitable for late-stage functionalization.

## Results and Discussion

Our group recently reported the reaction of thiouronium salts with alcohols to afford thioethers without requiring the use of thiol reactants.^16^ The formation of a stable urea (C=O) in exchange for a less stable thiourea (C=S) derivative was identified as a plausible thermodynamic driving force of the reaction.^17^

By analogy, we surmised that the reaction of thiouronium ylides with carbonyl compounds might also be thermodynamically biased toward the formation of a urea byproduct and thus favor an olefination pathway, in a manner akin to the Wittig reaction. These considerations, along with the potential tuneability of reactivity that is offered by thiouronium salts (by modulation of their N- substituents), prompted us to investigate them as olefination reagents.

**Figure 2:**
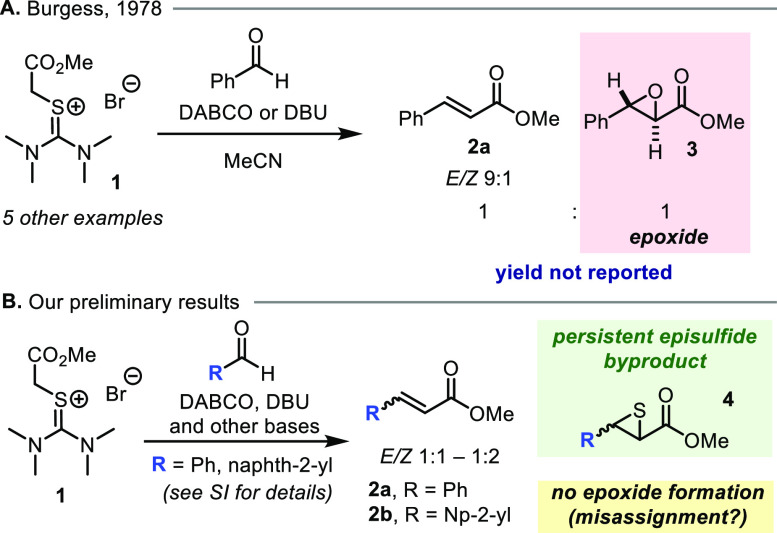
Revisiting Burgess’ observations in carbonyl olefination with thiouronium ylides.

Between 1976 and 1978, Burgess and co-workers described syntheses of thiouronium compounds and a preliminary assessment of their reactivity with aldehydes.^18^ Interestingly, the authors reported the formation of both epoxide and olefin products, as typified by the reaction of **1** with benzaldehyde to give methyl cinnamate (**2a**) and **3** in a 1:1 ratio (Figure 2A). This precedent provided initial support for our hypotheses and a starting point for our investigations.^18a^

First, we examined the reaction of **1** with 2-naphthaldehyde and benzaldehyde. While we did observe the formation of alkenes (**2**) with low *E*/*Z* selectivity, no epoxide products were detected under a range of different reaction conditions (Figure 2B and the Supporting Information (SI)). Instead, we found that episulfide **4** was the major byproduct of the reaction, prompting us to consider the possibility that **3** had been misassigned by Burgess and co-workers.^18a^ Unfortunately, characterization data for the compound **3** was not reported by Burgess, and we can only speculate that the true identity of originally described epoxide **3** was that of its episulfide congener **4**. With proof of principle in hand, we sought to optimize this reaction to improve its stereoselectivity and yield, as well as to suppress the formation of the episulfide byproduct.

Early in our investigations, we found that the solubility of bromide **1** was poor in ethereal solvents, preventing us from investigating strong bases at cryogenic temperatures. We later found that solubility could be increased by exchanging the bromide counterion for bistriflimide (NTf_2_), and thiouronium **5a** thus became the starting point for optimization. First, we examined the influence of the base on the reaction outcome, noting that olefin **2b** was produced in moderate to high yield (55–93%, Table 1, entries 1–4, and SI) with several bases stronger than triethylamine, including DBU, LDA, and Barton’s base (BMTG). Unfortunately, the stereoselectivity observed with thiouronium **5a** was poor even at low temperature, and we decided to explore modulation of the reagent structure. To this end, we treated **5b** and **5c**, carrying bulkier *N*-substituents, with BTMG in the presence of 2-naphthaldehyde. Pleasingly, a marked increase in stereoselectivity was observed. In the case of reagent **5c**, *Z-*alkene **2b** was delivered as the single detectable isomer in 92% yield when 1.2 eq of BTMG was deployed.

**Table 1:**
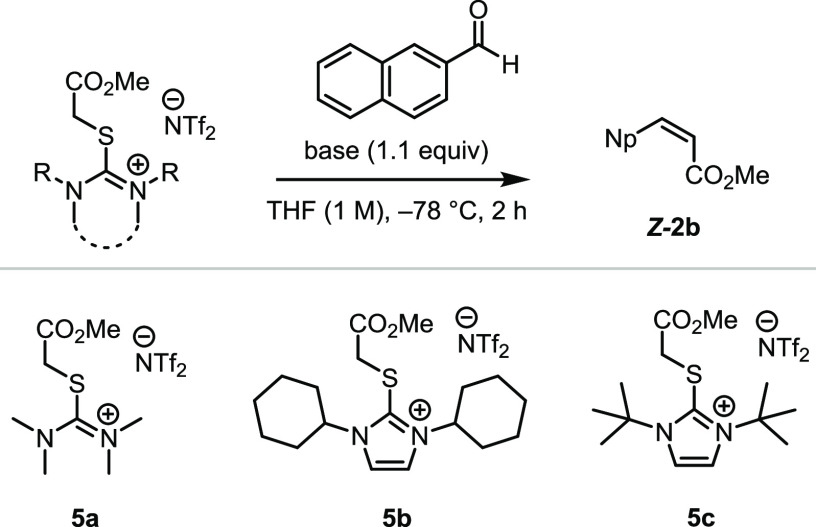
Optimization of the *Z*-Selective Olefination^a^

entry	thiouronium salt	base	olefin (*E*:*Z*)
1	**5a**	Et_3_N	
2	**5a**	DBU	55% (1/2.6)
3	**5a**	LDA	91% (1.2/1)
4	**5a**	BTMG	93% (1/3.6)
5	**5b**	BTMG	60% (1/18)
6	**5c**	BTMG	92%^b^ (only *Z*)

a BTMG = 2-*tert*-butyl-1,1,3,3,-tetramethylguanidine; DBU = 1,8-diazabiocyclo[5.4.0]-undec-7-ene; LDA = lithium diisopropylamide;

b 1.2 BTMG, 0.3 M, isolated yield. See the SI for full details.

**Figure 3:**
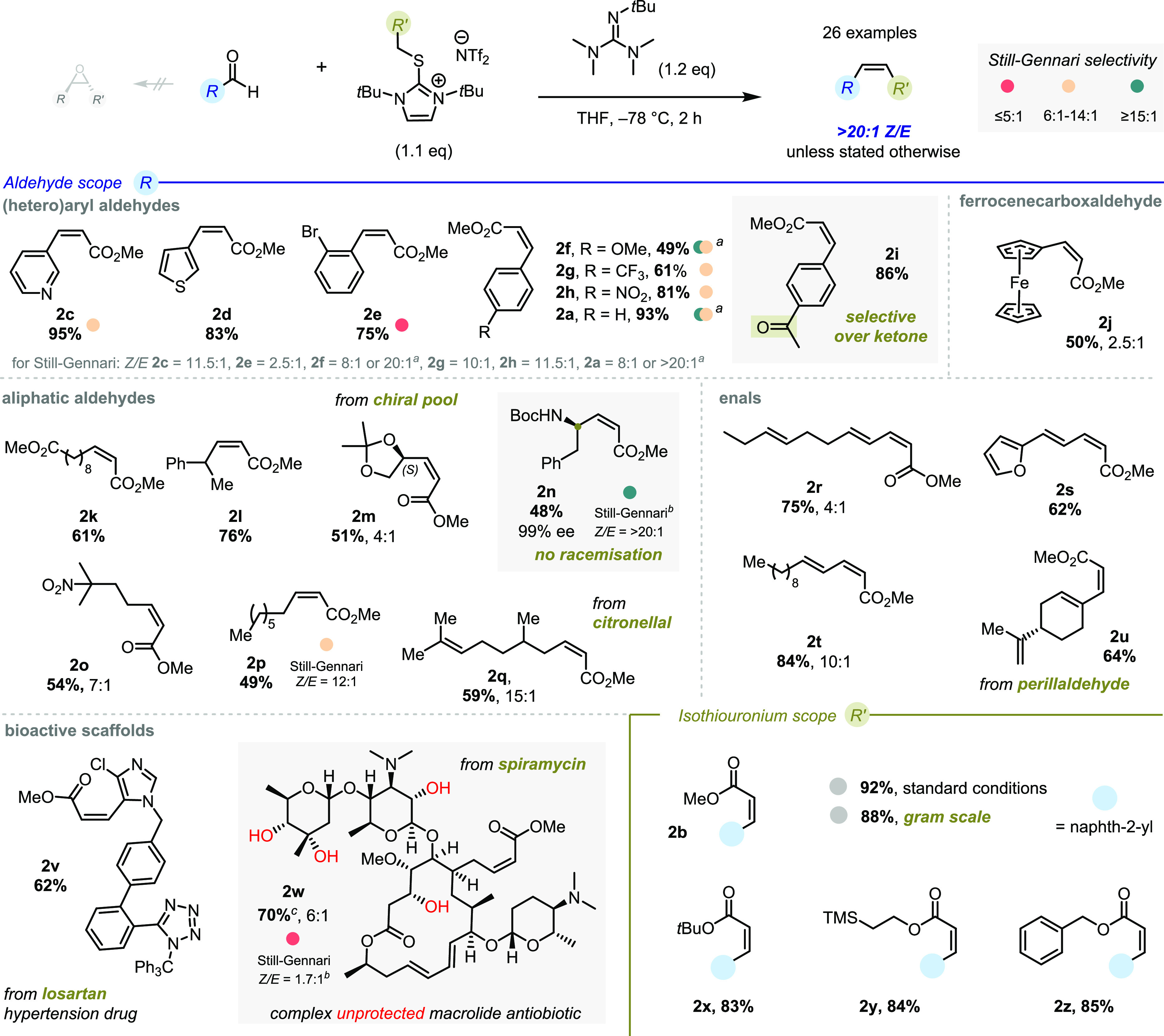
Substrate scope of the *Z*-selective olefination of aldehydes; *reaction conditions*: aldehyde (0.2 mmol), thiouronium salt (0.22 mmol), BMTG (0.24 mmol) at −78 °C in THF (1.0 M) for 2 h; ^[a]^Still–Gennari *Z/E* ratios given from two separate literature reports; ^[b]^Still–Gennari reaction executed in-house—see the SI refs to all other Still–Gennari data; ^[c]^combined yield by ^1^H NMR.

These optimized conditions for *Z*-selective olefination were then applied to a broad range of substrates (Figure 3, **2c**). Aromatic and heteroaromatic aldehydes performed well, delivering a range of substituted acrylates (**2a** and **c**–**i**) in high yield and >20:1 stereoselectivity, which compared favorably with the bench-mark Still–Gennari protocol (*Z/E* 2.5:1–11.5:1), as did several other examples—see color coding in Figure 3. Ferrocenecarboxaldehyde was also found to be a competent substrate (**2j**). Aliphatic aldehydes performed well, being cleanly converted to the respective *Z-*alkenes—again with typically high stereoselectivity. Among these substrates were notable chiral pool building blocks *N*-Boc-d-phenylalaninal, citronellal and (*R*)-glyceraldehyde acetonide. Importantly, no racemization of the sensitive chiral center of *N*-Boc-d-phenylalaninal took place and **2n** was formed with 99% *ee* (100% *es*). Next, we extended the scope of aliphatic aldehydes to enals, which were found to react with similarly high yields and selectivities, including important monoterpene perillaldehyde (giving **2u**).

We then sought to validate this *Z*-selective olefination on complex bioactive scaffolds. A derivative of hypertension drug losartan was found to smoothly undergo olefination to give **2v** in >20:1 stereoselectivity. Pleasingly, even spiramycin, a large macrolide antibiotic bearing unprotected alcohols, tertiary amines, a 1,3-diene, and glycosides, could be converted into the desired *Z*-acrylate **2w** in 61% yield, showcasing the synthetic potential of this olefination.

Regarding the thiouronium reactant, modification of the ester group was well--tolerated, and synthetically useful *tert*-butyl, ethylene-TMS, and benzyl esters were installed (**2x**, **2y**, and **2z**) in essentially identical yield and selectivity compared to the model methyl ester **2b**. Additionally, gram-scale synthesis of **2b** proceeded with near identical efficiency (88%).

Having established that thiouronium ylides can indeed be competent olefination reagents, we sought to probe how general this divergence from canonical *S*-ylide reactivity was. *N*-Tosylimines are known to react with sulfur ylides to give *N*-tosylaziridines, in analogy to the Corey–Chaykovsky epoxidation.^8−10,19^ We surmised that thiouronium ions might also contradict this reactivity paradigm.

Preliminary investigations of the reactivity of **1** with *N-*tosylimines indeed showed a clear bias toward olefination.^20^ Interestingly, the *E*-olefin was formed preferentially, presenting the possibility of developing a general method for divergent access to both olefin geometries. We optimized the reaction for *E*-stereoselectivity, finding the sterically unencumbered thiouronium bromide **1** to be ideal and the reaction to proceed smoothly at −40 °C.

**Figure 4:**
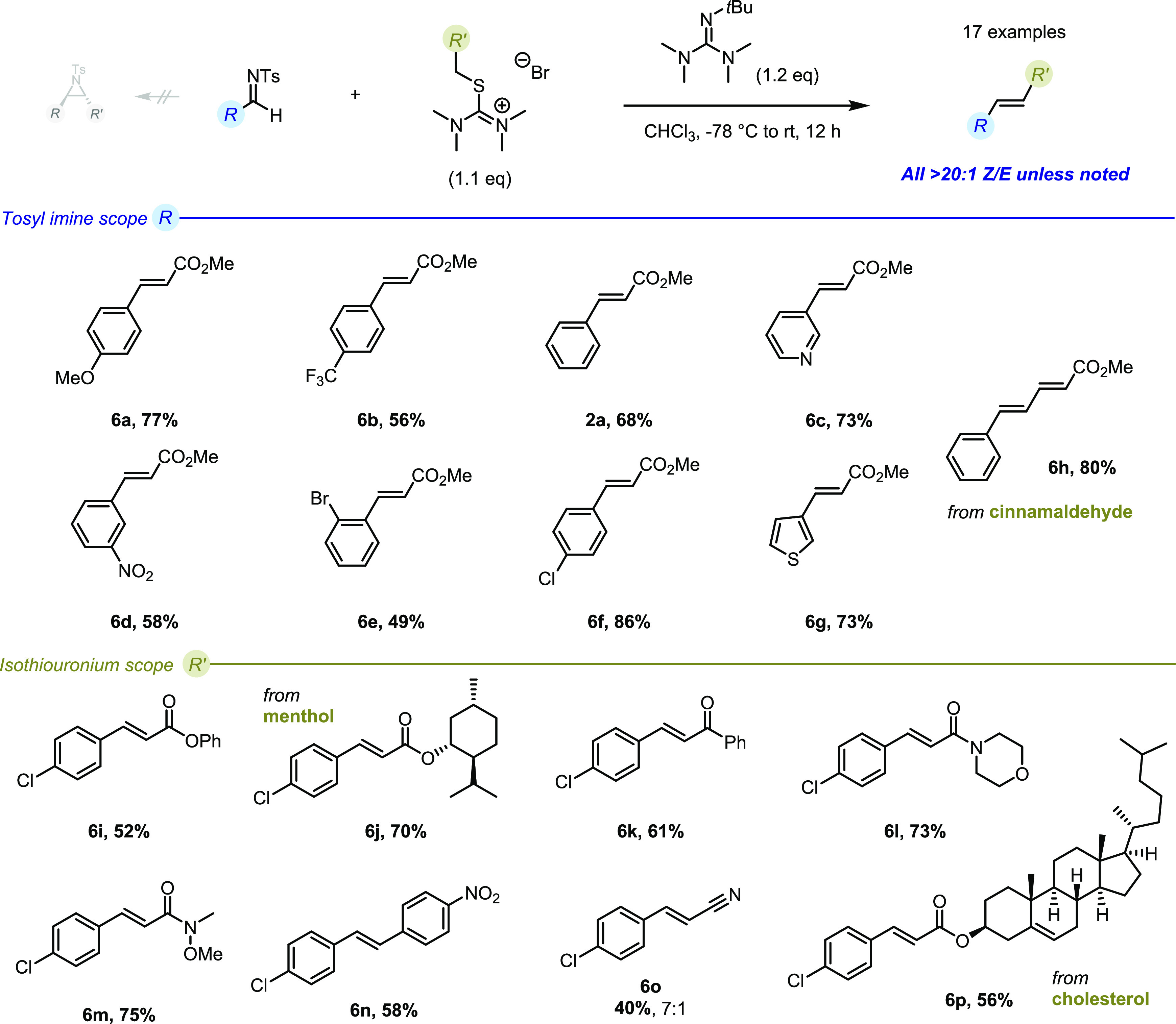
Substrate scope of the *E*-selective olefination of tosyl imines; *reaction conditions*: tosyl imine (0.2 mmol), thiouronium (0.22 mmol), BMTG (0.24 mmol) at −78 °C in CHCl_3_ (0.1 M) for 12 h.

We then examined the substrate scope of the reaction, focusing initially on the imine component (Figure 4). Treatment of a range of *N*-tosylimines with 1.1 equiv of **1** and 1.2 equiv of Barton’s base delivered the respective olefins as single stereoisomers in good to excellent yields (**6a**–**6p**, **2a**). Next, we examined the use of different thiouronium ylides carrying ester, ketone, amide, nitrile, steroid, and aromatic substituents—all delivering the products in moderate to high yield, in greater than 20:1 selectivity in all cases but one (**6p**).

**Figure 5:**
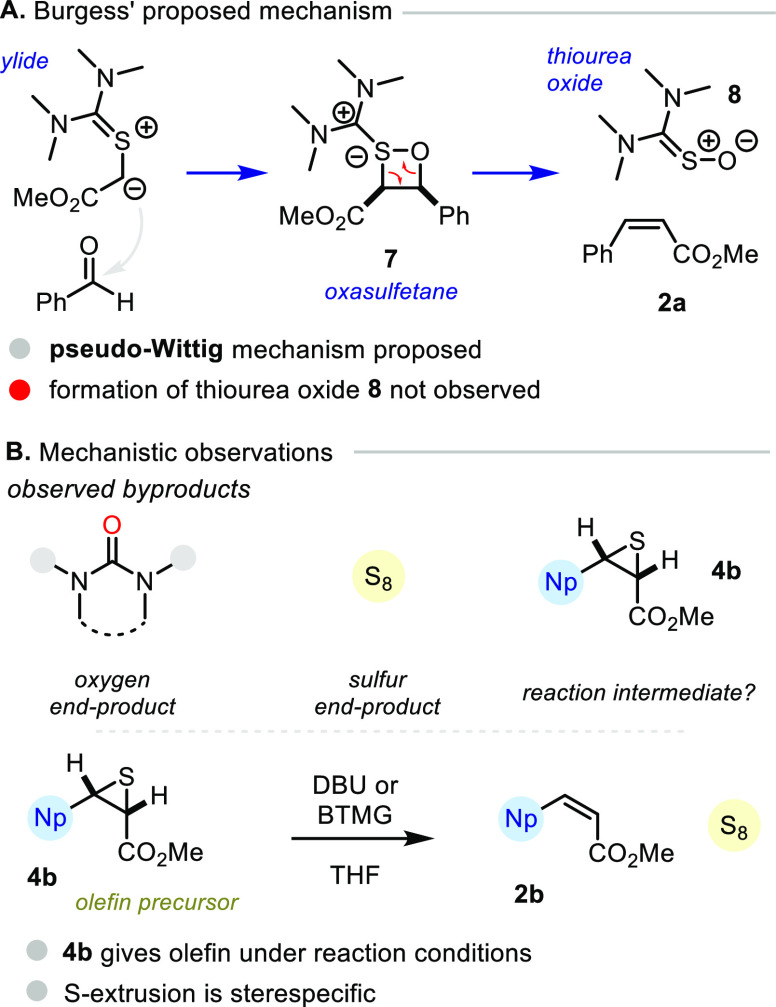
Experimental observations of mechanistic relevance.

At this stage we sought to shed light on the mechanisms at play. In the early work of Burgess, a quasi-Wittig reaction mechanism involving oxasulfetane **7** was proposed (Figure 5A).^18a^ However, we deemed the presence of such an intermediate unlikely due to the necessary production of thiourea *S*-oxide **8**, which was never observed in our investigations. Instead, we persistently observed urea byproducts, alongside elemental sulfur and in some cases episulfide **4b** (Figure 5B). This led us to consider episulfide **4b** as an intermediate en route to the olefin **2b**, and indeed, we observed the stereospecific formation of olefin **2b** when diastereomerically pure episulfide **4b** was treated with DBU or BTMG.^21^ With these experimental observations in mind, we initiated an in-depth computational study to interrogate the precise mechanism of the olefination reactions.

**Figure 6:**
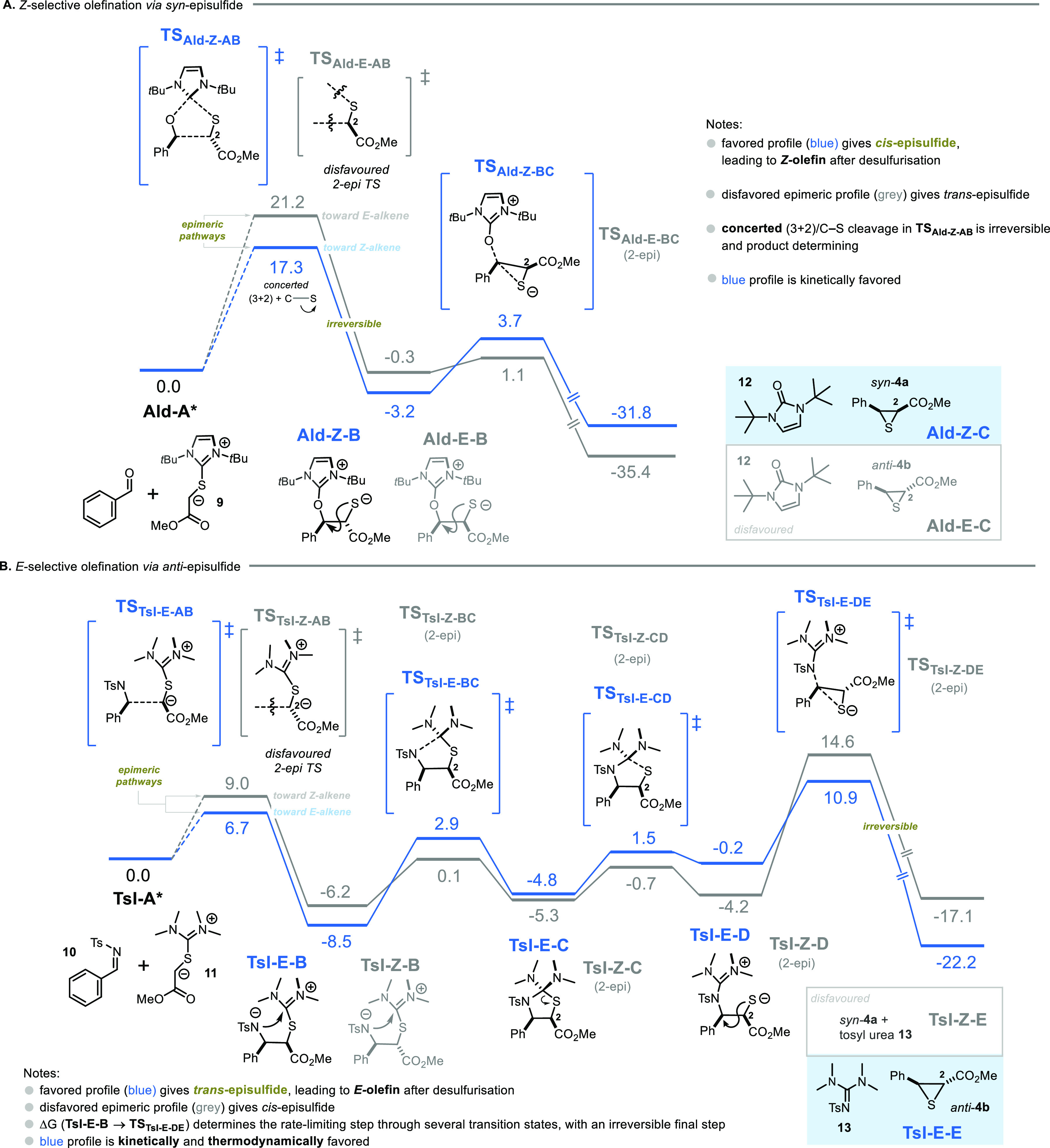
Energy profiles of the C–C coupling of ylide **9** with benzaldehyde (A), and **11** and tosyl imine **10** (B) for the two possible epimers: *cis-*episulfide or *trans-*episulfide. Favored profile shown in blue and disfavored shown in gray. Relative Gibbs free energies are presented in kcal mol^–1^ (298 K). The separated reactants (Ald/TsI-A*) serve as a reference (0.0 kcal mol^–1^). Calculations were performed at the PBE0-D3BJ/def2-TZVP,SMD//PBE0-D3BJ/def2-SVP,SMD level of theory (see the SI for details and discussion).

Density functional theory (DFT) calculations were performed at the PBE0-D3BJ/def2-TZVP,SMD//PBE0-D3BJ/def2-SVP,SMD level of theory (see the SI for details and discussion). The mechanisms for the formation of products *syn-***4a** and *anti-***4a** were calculated for the coupling of the *in situ* generated thiouronium ylide **9** with benzaldehyde (Figure 6a) and *N*-tosyl imine **10** with thiouronium ylide **11** (Figure 6b).

The Gibbs free energy profile for the reaction with benzaldehyde (Figure 6) shows an irreversible (3 + 2)-cycloaddition-type transition state (**TS**_**Ald-Z-AB**_), with simultaneous C–S bond cleavage to give a diastereomeric pair of acyclic intermediates (trans, **Ald-Z-B** or cis, **Ald-E-B**). From both of these structures, an S_N_2-type attack of the sulfide breaks the C–O bond, forming the urea and leading to the corresponding episulfides **Ald-Z-C**, via **TS**_**Ald-Z-BC**_ (profile in blue, *major*), and **Ald-E-C** via **TS**_**Ald-E-BC**_ (profile in gray, *minor*). The lower activation barrier for the formation of the major episulfide, **Ald-Z-C** (Δ*G*^*‡*^(Ald-A*→ **Ald-Z-B**) = 17.3 kcal mol^–1^ while

Δ*G*^*‡*^(Ald-A*→ **Ald-E-B**) = 21.2 kcal mol^–1^) strongly suggests that the reaction is kinetically controlled. Energy decomposition analysis revealed that a greater steric clash in **TS**_**Ald-E-BC**_ compared with **TS**_**Ald-Z-BC**_ accounts for this kinetic selectivity (see the SI for details). Unlike the case of aldehyde olefination, episulfide formation of *N*-tosylimine **10** with **11** (Figure 6B) is a stepwise process. This first entails C–C bond formation via an acyclic transition state (**TS**_**TsI-E-AB**_ or **TS**_**TsI-Z-AB**_), yet again generating two possible epimeric pathways (steps TsI-A*→ **TsI-E-B** in the profile in blue and TsI-A*→ **TsI-Z-B** in the profile in gray).

Nucleophilic attack of the tosyl amide at the thiouronium moiety of **TsI-E-B** or **TsI-Z-B** leads to C–N bond formation, producing discrete thiazolidines **TsI-E-C** and **TsI-Z-C** via **TS**_**TsI-E-BC**_ and **TS**_**TsI-Z-BC**_, respectively. Thiazolidine intermediates **TsI-E-C** and **TsI-Z-C** readily ring open by C–S bond cleavage, forming the intermediates **TsI-E-D** and **TsI-Z-D**, respectively. Similarly to the scenario described in Figure 6A, the last step is an S_N_2-type attack, which cleaves the C–N bond and yields the episulfides with inversion of the configuration. Therefore, in contrast to the reaction with the aldehyde electrophile, the bulkiness of the *N-*tosylimine promotes a stepwise mechanism toward formation of the experimentally observed *trans-*episulfide **TsI-E-E**, which is both thermodynamically and kinetically favorable.

**Figure 7:**
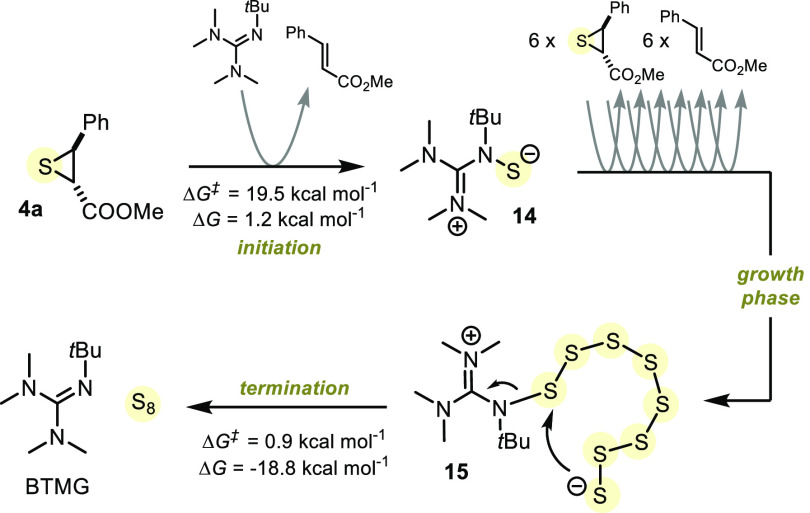
Sulfur extrusion by BMTG; For *syn*-**4a**: Δ*G*^*‡*^ = 21.2 kcal mol^–1^ and Δ*G* = 5.4 kcal mol^–1^.

The observed selective formation of *Z*-olefin from the *cis*-episulfide and *E*-olefin from the *trans-*episulfide (Figure 5 and the SI) indicated that the sulfur extrusion mechanism is stereospecific. Our calculations were consistent with this observation, showing that excision of a sulfur atom from either episulfide *anti-***4a** or *syn*-**4a** by BTMG to selectively yield the corresponding olefin was thermodynamically feasible under the reaction conditions (Figure 7 and the SI). Together with the formation of an olefin, the BMTG-sulfur adduct **14** would then be generated. The initial stoichiometry of BTMG is 1.2 equiv, of which 1 equiv is required to form the thiouronium ylide. Given that only 0.2 equiv of base would remain, desulfurization evidently did not require stoichiometric base, and we sought to interrogate if BTMG could be regenerated in a pseudocatalytic process. As such, the pathway for base regeneration was also studied (Figure 7), considering the experimentally observed formation of elemental sulfur.

The obtained Gibbs free energy profile showed that nucleophilic attack on the episulfide was kinetically more favorable when performed by the BTMG-sulfur adduct **14** [Δ*G*^*‡*^(growth phase, first step) = 13.3 kcal mol^–1^] than by BTMG alone (Δ*G*^*‡*^ = 19.5 kcal mol^–1^). This suggested that the formation of BTMG-sulfur adduct **14** served as an initiation step and that ensuing sulfur extrusion steps would form a BTMG-polysulfide adduct through iterative S–S bond formation (termed the *growth phase*, Figure 7). Our calculations showed that early termination of the growth phase through release of S_2_ was kinetically and thermodynamically unfavorable. Instead, termination of the growth phase by release of S_8_ from BTMG-octasulfide adduct **15** was shown to be a favorable pathway to BTMG regeneration.

## Conclusion

In summary, we have developed a stereodivergent olefination method based on thiouronium ylides. This selective transformation, suitable for complex molecule synthesis and late-stage functionalization, challenges the canonical reactivity of S-ylides toward carbonyl derivatives. In-depth computational studies revealed that selective episulfide generation is at the heart of the olefination process, while clarifying the role of the base in a domino sulfur extrusion event. While enhancing the “synthetic toolbox” for carbonyl olefination, we believe this work adds a subtle new layer to the textbook phosphorus/sulfur ylide dichotomy.

## Supporting Information Available

Discussions of general information, reaction optimization, mechanistic studies, computational studies, Cartesian coordinates, preparation of substrates, *Z*-selective olefination of aldehydes, *E*-selective olefination of *N*-tosylimines, and limitations, tables of preliminary investigations, and figures of NMR study, energy profiles, Newman representation, results of SAPT analysis, 3D and schematic representations of geometry, direct Gibbs free energy comparison, and NMR spectra (PDF)
