# Supplementary material for: Direct Stereodivergent Olefination of Carbonyl Compounds with Sulfur Ylides
Source: J Am Chem Soc. 2022 Jun 30;144(27):12536–43. doi: 10.1021/jacs.2c05637 (PMC9284548; doi:10.1021/jacs.2c05637)
Supplement: Supplementary file 1 — ja2c05637_si_001.pdf [file ja2c05637_si_001.pdf]

Direct stereodivergent olefination of carbonyl compounds with sulfur ylides

Jérémy Merad[‡,a], Phillip S. Grant[‡,a], Tobias Stopka[‡,a], Juliette Sabbatani[a], Ricardo Meyrelles[a,b,c], Alexander Preinfalk[a], Ján Matyasovsky[a], Boris Maryasin[a,b], Leticia González[b], Nuno Maulide[a]*

[a]*Institute of Organic Chemistry, University of Vienna, 1090 Vienna, Austria*
[b]*Institute of Theoretical Chemistry, University of Vienna, 1090 Vienna, Austria*
[c]*Doctoral School in Chemistry, University of Vienna, 1090 Vienna, Austria*

E-Mail: nuno.maulide@univie.ac.at, Homepage: http://maulide.univie.ac.at

Contents

1. General Information S2
2. Reaction optimisation S3
  2.1 Preliminary investigations S3
  2.2 Optimisation of the *Z*-selective olefination S4
3. Mechanistic studies S6
  3.1 Mechanistic experiments S6
  3.2 Computational studies S10
    3.2.1 Description of the energy profiles S10
    3.2.2 XYZ Structures S18
4. Preparation of substrates S37
  4.1 Aldehydes S37
  4.2 *N*-Tosylimines S37
  4.3. Isothiouronium salts S38
5. *Z*-selective olefination of aldehydes S48
  5.1 General Procedure S48
  5.2 Characterisation S48
6. *E*-selective olefination of *N*-tosylimines S59
  6.1 General Procedure S59
  6.2 Characterisation S59
7. Limitations S64
8. NMR spectra S67
  8.1 Thioureas S70
  8.2 Thiouronium bromides S74
  8.3 Thiouronium bistriflimides S91
  8.4 *Z*-olefins S102
  8.5 *E*-olefins S128
9. References S145

1. General Information

Unless otherwise stated, all glassware was flame-dried before use and all reactions were performed under an atmosphere of argon. All solvents were distilled from appropriate drying agents prior to use or directly taken from commercial sealed bottles under an atmosphere of argon. All reagents and commercially available substrates were used as received from commercial suppliers unless otherwise stated. Experiments conducted without external heating or cooling are designated as at “room temperature” (rt), which ranged between 21 °C and 23 °C. Reaction progress was monitored by thin layer chromatography (TLC) performed on aluminium plates coated with silica gel F254 with 0.2 mm thickness. Chromatograms were visualised by fluorescence quenching with UV light at 254 nm or by staining using potassium permanganate. Flash column chromatography was performed using silica gel 60 (230-400 mesh, Merck and co.). Neat infrared spectra were recorded using a Bruker Vertex 70 FT-IR spectrometer. Wavenumbers are reported in cm^{-1}. Mass spectra were obtained using a Bruker maXis UHR-TOF spectrometer, using electrospray ionization (ESI) and by Agilent 7200B GC/Q-TOF spectrometer, using electron impact (EI). All ^{1}H NMR, ^{13}C NMR and ^{19}F NMR spectra were recorded using a Bruker AV III 400, AV NEO 500, AV III 600 or AV III HD 700 spectrometer in $CDCl_3$ or DMSO-d_6. Chemical shifts are given in parts per million (ppm, δ), referenced to the solvent residual peak of $CDCl_3$ or DMSO-d_6, defined at δ = 7.26 ppm (^{1}H NMR) and δ = 77.16 (^{13}C NMR) for $CDCl_3$, and δ = 2.52 ppm (^{1}H NMR) and δ = 39.52 (^{13}C NMR) for DMSO-d_6. Coupling constants are quoted in Hz (J). ^{1}H NMR splitting patterns were designated as singlet (s), doublet (d), triplet (t), quartet (q), pentet (p). Splitting patterns that could not be interpreted or easily visualised were designated as multiplet (m), apparent (app) or broad (br). Enantiomeric excess was measured on a Shimadzu LC-8A preparative HPLC system using Lux Cellulose-1 or Lux Cellulose-3 chiral columns.

2. Reaction optimisation

2.1 Preliminary investigations

Table S1. Investigation of isothiouronium bromides for the olefination of 2-naphthaldehyde

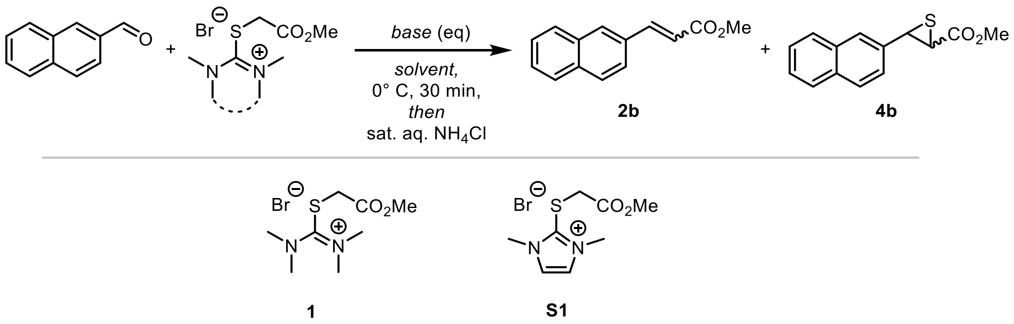

entry	sulfonium salt	base	eq	solvent	olefin (*E*:*Z*)	thiirane (*syn:anti*)
1	**1**	DBU	1	$CHCl_3$	85% (54:46)	not observed
2	**1**	Et_3N	1	$CHCl_3$	3% (100:0)	19% (48:52)
3	**1**	DABCO	1	$CHCl_3$	7% (50:50)	not observed
4	**1**	DIPEA	1	$CHCl_3$	1% (100:0)	30% (49:51)
5	**1**	DBU	3	$CHCl_3$	83% (57:43)	not observed
6	**1**	DBU	0.5	$CHCl_3$	5% (100:0)	50% (48:52)
7	**1**	DBU	1	CH_3CN	60% (47:53)	4% (0:100)
8	**1**	DBU	1	CH_2Cl_2	66% (47:53)	2% (0:100)
9	**1**	DBU	1	DMF	46% (47:53)	4% (0:100)
10	**1**	DBU	1	THF	5% (100:0)	not observed
11	**S1**	DBU	1	$CHCl_3$	83% (34:66)	not observed

Procedure: To a solution of 2-naphthaldehyde (0.1 – 0.2 mmol, 1 eq) and isothiouronium bromide (1.1 eq) in the solvent (0.1 M) at 0 °C was added base. After 30 min stirring at this temperature, the reaction mixture was allowed to warm to rt before the addition of sat. aq. NH_4Cl. The resulting mixture was extracted with CH_2Cl_2, and the combined organic extracts were washed with brine and dried over magnesium sulfate, before being concentrated *in vacuo*. The crude mixture was analysed by ^{1}H NMR using mesitylene as an internal standard.

Preliminary investigations into the olefination of aldehydes with isothiouronium bromides were characterised by poor diastereoselectivity and/or low yield. Thiirane **4b** was a persistent by-product and no epoxides were observed, in contrast to the work of Burgess *et al*.[1] It was also observed that with sub-stoichiometric amounts of DBU, a greater amount of thiirane **4b** was observed (entry 6). In general, solubility

of isothiouronium bromides was poor, particularly in THF. Later investigations (Table S2) were focused on isothiouronium bistriflimides, which exhibited better solubility (particularly in THF), allowing us to investigate cryogenic reaction temperatures to enhance diastereoselectivity.

2.2 Optimisation of the *Z*-selective olefination

Table S2. Investigation of a simple isothiouronium bistriflimide for the olefination of 2-naphthaldehyde

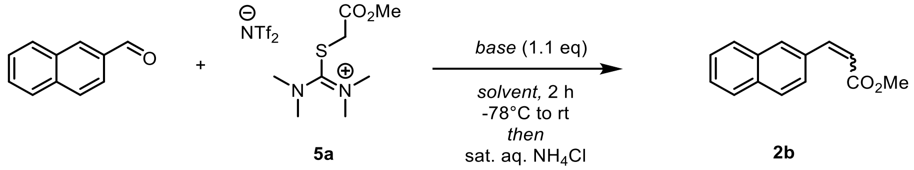

entry	base	solvent	olefin (*E*:*Z*)
1	LDA	THF	91% (1.2:1)
2	KHDMS	THF	93% (1.3:1)
4	NaH	THF	not observed
5	KH	THF	not observed
6	Et_3N	THF	not observed
7	DBU	THF	55% (1:2.6)
8	BTMG	THF	93% (1:3.6)
9	BTMG	CH_2Cl_2	90% (2.2:1)
10	BTMG	Toluene	57% (1:1.3)

Procedure: To a solution of 2-naphthaldehyde (0.1 – 0.2 mmol, 1 eq) and isothiouronium bistriflimide (1.1 eq) in the solvent (0.1 M) at –78 °C was added base. After 2 h stirring at this temperature, the reaction mixture was allowed to warm to rt before the addition of sat. aq. NH_4Cl. The resulting mixture was extracted with CH_2Cl_2, and the combined organic extracts were washed with brine and dried over magnesium sulfate, before being concentrated *in vacuo*. The crude mixture was analysed by ^1H NMR using mesitylene as an internal standard.

The use of isothiouronium bistriflimides enabled investigation of cryogenic temperature to enhance diastereoselectivity of the olefination. Several bases and solvents were investigated, however, the diastereoselectivity was uniformly poor. Therefore, attention was turned increasing steric bulk of the thiouronium salt.

Table S3. Investigation of bulky isothiouronium bistriflimides for the olefination of 2-naphthaldehyde

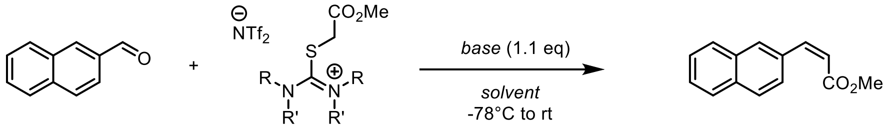

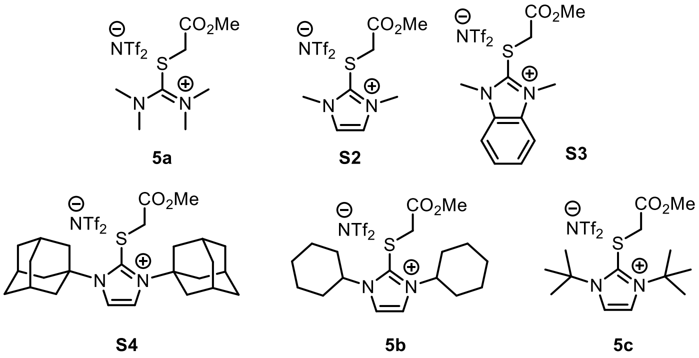

entry	thiouronium salt	olefin yield (*E:Z*)
1	**5a**	93% (1:3.6)
2	**S2**	97% (1:2.8)
4	**S3**	22% (1:1.5)
5	**S4**	91% (only *Z*)
6	**5b**	60% (1:18)
7	**5c**	73% (only *Z*)
8	**5c**	85% (only *Z*)*
9	**5c**	92% (only *Z*)*†
10	**5c** (in situ generation from bromide salt)	80% (1:3)*‡

Procedure: To a solution of 2-naphthaldehyde (0.1 – 0.2 mmol, 1 eq) and isothiouronium bromide (1.1 eq) in the solvent (1 M) at –78 °C was added BTMG (1.2 eq). After 2 h stirring at this temperature, the reaction mixture was allowed to warm to rt before the addition of sat. aq. NH_4Cl. The resulting mixture was extracted with CH_2Cl_2, and the combined organic extracts were washed with brine and dried over magnesium sulfate, before being concentrated *in vacuo*. The crude mixture was analysed by ^{1}H NMR using mesitylene as an internal standard.*Conducted with 1.2 equivalents of BTMG. †Conducted at 0.3 M concentration; isolated yield.‡ 1.2 eq lithium bistriflimde additive.

We observed that increasing steric bulk of the thiouronium salt had a marked effect on the diastereoselectivity of the reaction. Both isothiouronium salts **5b** and **5c** performed well, and salt **5c** was chosen for further studies in the interest of improved atom economy. In situ generation of **5c** from the corresponding bromide salt was found to have a negative effect on stereoselectivy, potentially due to a deleterious effect of the lithium counterion.

3. Mechanistic studies

3.1 Mechanistic experiments

Identification of thiirane as an olefin precursor

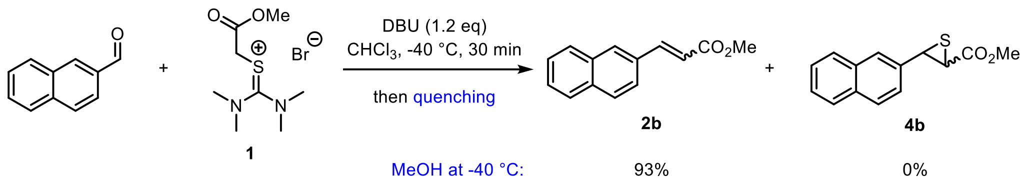

Quenching	2b	4b
MeOH at -40 °C:	93%	0%
AcOH at -40 °C:	2%	87%
rt then AcOH:	94%	0%

A flame-dried Schlenk tube equipped with a stir bar was charged with 2-napthaldehyde (0.20 mmol, 1 eq) and isothiouronium salt **1** (62 mg, 0.22 mmol, 1.1 eq) under argon. Chloroform (2.0 mL, 0.1 M) was added and the resulting mixture was stirred at –40 °C for 10 minutes. DBU (0.24 mmol, 36 µL, 1.2 eq) was then added and the resulting mixture was stirred at –40°C for 30 minutes, after which the reaction was quenched under the indicated conditions [MeOH or AcOH (0.5 mL) at -40 °C or rt]. The crude material was extracted three times with 2 mL of dichloromethane; the organic phases were combined and dried over magnesium sulfate. The solvent was then removed under reduced pressure and the residue was purified by chromatography on silica gel to give the corresponding olefin and/or thiirane.

The same reaction performed in $CDCl_3$ and monitored by ^{1}H NMR from –40 °C to rt demonstrated that the thiirane is an olefin precursor at low temperature:

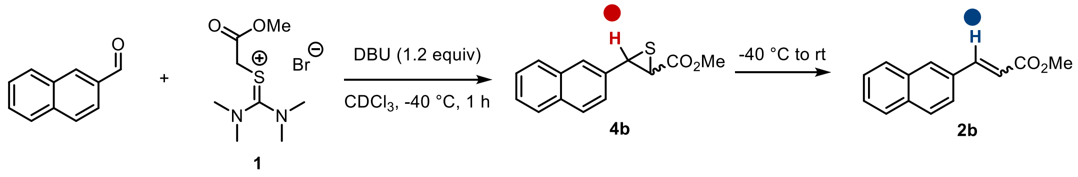

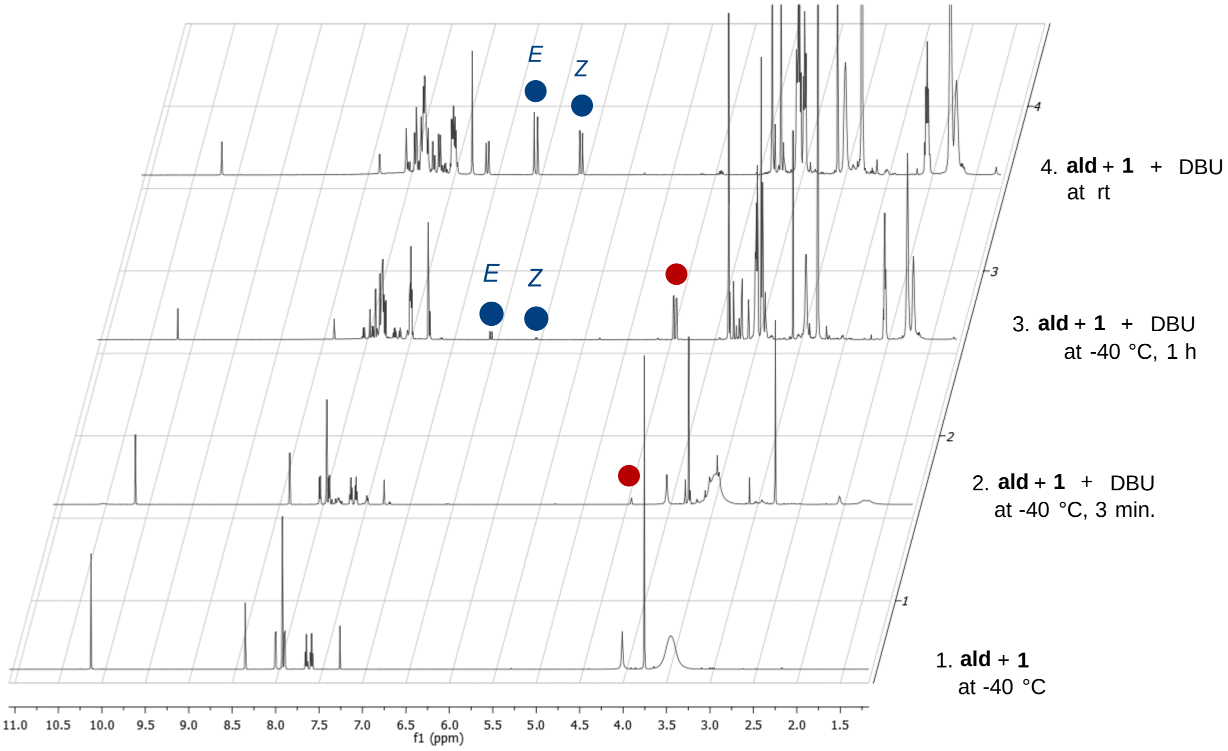

Figure S1. NMR study of desulfurisation process

Desulfurisation stereospecificity

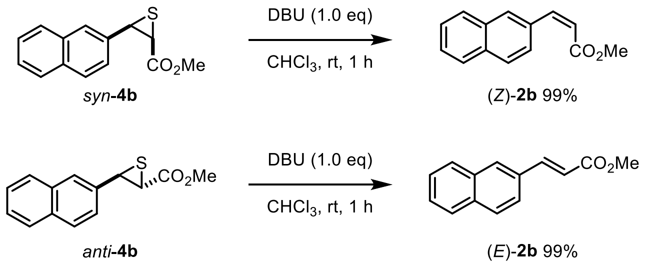

A flame-dried Schlenk tube equipped with a stir bar was charged with the indicated thiirane **4b** (48.9 mg, 0.20 mmol, 1 eq) and chloroform (2.0 mL, 0.1 M) under argon atmosphere. DBU (0.20 mmol, 30 μL, 1 eq) was then added and the resulting mixture was stirred at rt for 1 hour. The reaction was then quenched with saturated aq. NH_4Cl, extracted three times with 2 mL of dichloromethane; the organic phases were

combined and dried over magnesium sulfate. The crude mixture was analysed by ^{1}H NMR using mesitylene as an internal standard.

Catalytic turnover in desulfurisation process

Under the standard conditions, 0.2 eq of BTMG should remain unreacted after deprotonation of the thiouronium salt, which is then available to mediate desulfurisation of the newly formed thiirane. Our calculations suggested that 1 eq of BTMG is able to convert 8 eq of thiirane to the respective olefin, producing S_8 and regenerating BTMG. Therefore, 0.2 eq of BTMG is able to completely mediate desulfurisation as a sub-stoichiometric process that does not necessitate catalytic turnover. Nevertheless, our computational investigations suggested this should be possible, and we sought to prove this experimentally. To this end, we demonstrated that 2 mol % BTMG was able to completely convert thiirane *syn*-**4b** to olefin (*Z*)-**2b**, demonstrating that catalytic turnover is possible at room temperature.

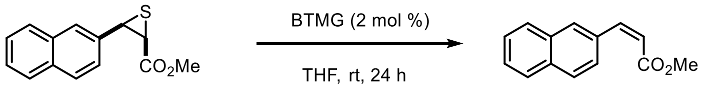

syn-**4a** (*Z*)-**2b**

single isomer

70:1 E/Z selectivity
full conversion

Experimental procedure: To episulfide *syn*-**4b** (11.0 mg, 0.045 mmol, 1.0 eq) in THF (0.5 mL) at rt was added BTMG (0.2 µl, 0.0009 mmol, 2 mol %) as solution in THF (20 µl). The resulting solution was stirred at rt for 24 hrs then concentrated *in vacuo*. The resulting crude oil was analysed by ^{1}H-NMR, revealing complete conversion to olefin (*Z*)-**2b** (70:1 *E/Z*).

Preparation of thiirane diastereomers:

- *Syn*-**4a** synthesis:

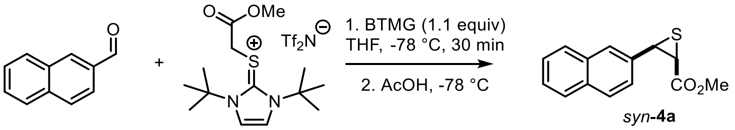

5c *syn*-**4a**

BTMG (0.44 mmol, 86 µL, 1.1 eq) was added dropwise to a solution of sulfonium salt **5c** (0.60 mmol, 340 mg, 1.5 eq) and 2-napthaldehyde (0.40 mmol, 62 mg, 1.0 eq) in THF (4 mL) at –78 °C. The yellow solution was then stirred at this temperature over 30 min and quenched by addition of AcOH (1 mL). The colorless solution was allowed to reach room temperature and dichloromethane (6 mL) and saturated aq. NH_4Cl (10 mL) were added. The layers were separated and the aqueous layer was extracted with dichloromethane (3 x 6 mL). The combined organic layers were dried with Na_2SO_4, filtered and concentrated under vacuum. *Syn* methyl 3-(naphthalen-2-yl)thiirane-2-carboxylate **4a** was isolated as a white solid (64 mg, 66% yield) by flash chromatography (5% heptane in EtOAc). **^{1}H NMR** (400 MHz, $CDCl_3$) δ 7.89 (s, 1H), 7.85 – 7.75 (m, 3H), 4.41 (d, J = 7.3 Hz, 1H), 3.81 (d, J = 7.3 Hz, 1H), 3.45 (s, 3H); **^{13}C NMR**

(101 MHz, $CDCl_3$) δ 168.3, 133.2, 133.1, 131.7, 128.6, 128.1, 127.79, 127.76, 126.42, 126.37, 126.3, 52.7, 41.5, 38.1; **HRMS** (ESI^+) m/z: $[M+Na]^+$ calculated 267.0450 for $C_{14}H_{12}O_2SNa^+$, 267.0449 found; **IR** (neat) *v* 3053, 2922, 2852, 1747, 1723, 1599, 1507, 1434, 1350, 1286, 1246, 1194, 1164, 1005, 860, 822, 747, 646, 611 cm^{-1}.

- *Anti*-**4a** synthesis:

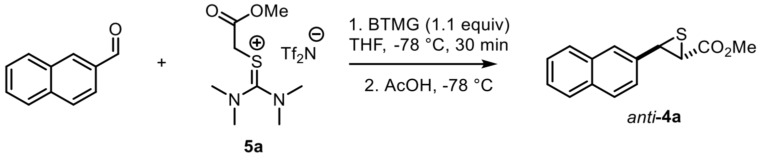

BTMG (0.66 mmol, 135 μL, 1.1 eq) was added dropwise to a solution of sulfonium salt **5a** (0.60 mmol, 292 mg, 1.0 eq) and 2-napthaldehyde (0.60 mmol, 94 mg, 1.0 eq) in THF (6 mL) at –78 °C. The yellow solution was then stirred at this temperature over 30 min and quenched by addition of AcOH (1 mL). The colorless solution was allowed to reach room temperature and dichloromethane (6 mL) and a saturated aq. NH_4Cl (10 mL) were added. The layers were separated and the aqueous one was extracted with DICHLOROMETHANE (3 x 6 mL). The combined organic layers were dried with Na_2SO_4, filtered and concentrated under vacuum. *Anti* methyl 3-(naphthalen-2-yl)thiirane-2-carboxylate **4a** was isolated as a white solid (44 mg, 16% yield) by flash chromatography (5% heptane in EtOAc). **^{1}H NMR** (400 MHz, $CDCl_3$) δ 7.87 – 7.74 (m, 4H), 7.59 – 7.44 (m, 2H), 7.29 (dd, *J* = 8.6 Hz, *J* = 1.7 Hz, 1H), 4.47 (d, *J* = 4.7 Hz, 1H), 3.83 (s, 3H), 3.66 (d, *J* = 4.7 Hz, 1H); **^{13}C NMR** (101 MHz, $CDCl_3$) δ 170.6, 134.1, 133.33, 133.30, 128.9, 127.9 (2C), 127.3, 126.8, 126.5, 123.7, 53.2, 41.8, 37.3; **HRMS** (ESI^+) m/z: $[M+Na]^+$ calculated 267.0450 for $[C_{14}H_{12}O_2SNa]^+$, 267.0456 found, **IR** (neat) *v* 3052, 2950, 2848, 1734, 1694, 1436, 1349, 1282, 1195, 1167, 1010, 818, 749, 633, 604 cm^{-1}.

Identification of elemental sulfur as the sulfur end-product

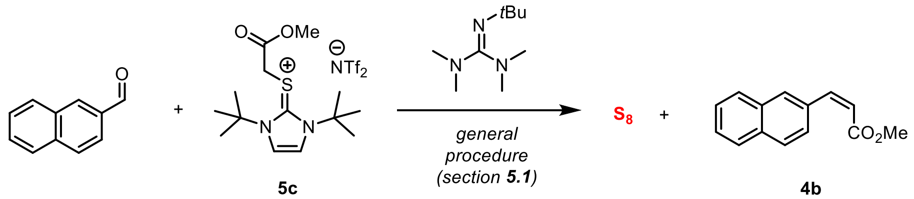

According to the general procedure for *Z*-selective olefination (see section 5.1), 2-naphthaldehyde (0.2 mmol) was reacted with isothiouronium **5c** (0.22 mmol) to afford elemental sulfur in 52% yield based on sulfur atom equivalents (3.3 mg, 0.013 mmol based on S_8) as a yellow solid after flash column chromatography (pentane). Elemental analysis: C, 7.58; H, 1.08; S, 89,93% (carbon and hydrogen attributed to grease, which was observed by ^{1}H-NMR and ^{13}C-NMR).

3.2 Computational studies

The conformational space of all molecules has been initially searched using meta-dynamics simulations based on semiempirical tight-binding quantum chemical calculations as implemented in CREST. [2,3]

Structures located with CREST have then been subjected to PBE0-D3BJ/def2-SVP[4–8] single point calculation. Structures within a window of 25 KJ mol^{-1} to the structure with the lowest energy have been subjected to geometry optimization at the same level of theory. The nature of all stationary points (minima and transition states) was verified through the computation of the vibrational frequencies. The thermal corrections to the Gibbs free energies were combined with the single point energies calculated at the PBE0-D3BJ/def2-TZVP level of theory to yield Gibbs free energies ("G_{298}") at 298.15 K. All energies are reported in kcal mol^{-1}. The energy profiles were constructed using the most stable conformation (the global minimum) of each intermediate and transition state.

The DFT calculations have been performed with the Gaussian 16 program package.[9] The polarizable continuum model (PCM) with SMD parameters[10,11] was applied to consider solvent effects for both geometries and energies. SMD parameters of dichloromethane were considered for the aldehyde reaction and tetrahydrofuran for the tosyl imine reaction and base regeneration.

Free energies in solution have been corrected to a reference state of 1 mol l^{-1} at 298.15 K through the addition of RTln(24.46) = +7.925 kJ mol^{-1} to the gas phase (1 atm) free energies.

Symmetry-adapted perturbation theory (SAPT)[12,13] calculations have been performed for the most stable conformations of transition states **$TS_{Ald-Z-AB}$**, **$TS_{Ald-E-AB}$**, **$TS_{TsI-E-AB}$**, and **$TS_{TsI-E-AB}$** with the Psi4 software package using the SAPT0 method along with the jun-cc-pVDZ basis[14,15].

3.2.1 Description of the energy profiles

In this study, the deprotonation of the thiouronium salt is not considered. Therefore, the thiouronium ylide form was used for the C–C coupling calculations.

The obtained Gibbs free energy profile (Figure S2) for the reaction with the aldehyde starts from the isolated reactants, phenyl aldehyde and thiouronium ylide (**Ald-A***). The formation of the reactant complex **Ald-A** is endergonic (Δ*G*(**Ald-A***→**Ald-Z-A**) = 5.6 kcal mol^{-1}). As discussed in the main text, the reaction with the aldehyde presents a single concerted step for the C–C coupling, nucleophilic attack of the oxygen, and S–C bond dissociation. The coupling step that leads to the *trans*-intermediate, **Ald-Z-B**, is exergonic (Δ*G*(**Ald-A***→**Ald-Z-B**) = -3.2 kcal mol^{-1}) and kinetically favorable (Δ*G*‡(**Ald-A***→**Z-B**) = 17.3 kcal mol^{-1}). The following step is an S_N2 type attack of the sulfide on the α-carbon to the oxygen, cleaving the C–O bond and forming the second S–C bond of the episulfide, **Ald-Z-C**. This step is highly exergonic (Δ*G*(**Ald-Z-B**→**Ald-Z-C**) = -28.6 kcal mol^{-1}) and has a low activation barrier (Δ*G*‡(**Ald-Z-B**→**Ald-Z-C**) = 6.9 kcal mol^{-1}). The competing C–C coupling step, leading to the *cis*-intermediate **Ald-E-B**, is slightly exergonic

(ΔG(**Ald-A*→ Ald-E-B**) = -0.3 kcal mol^{-1}). However, it has a significantly higher activation barrier than the competing step **Ald-A*→ Ald-Z-B** ($\Delta G^{‡}$(**Ald-A*→Ald-E-B**) = 21.2 kcal mol^{-1}). The following step, the formation of the *cis*-episulfide, is also exergonic (ΔG(**Ald-E-B→Ald-E-C**) = -35.1 kcal mol^{-1}). These results show that the observed product is both kinetically and thermodynamically favored in the reaction with tosylimine, while the kinetic product is observed in the reaction with aldehyde. According to the energy span model[16], the apparent activation barrier of both profiles shown in Figure S2 is determined in the first step, **Ald-A*→Ald-Z-B** or **Ald-A*→Ald-E-B**, since the rate-limiting states are the separated reactants **Ald-A*** and the transition states **TS$_{\text{Ald-Z-AB}}$** and **TS$_{\text{Ald-E-AB}}$**.

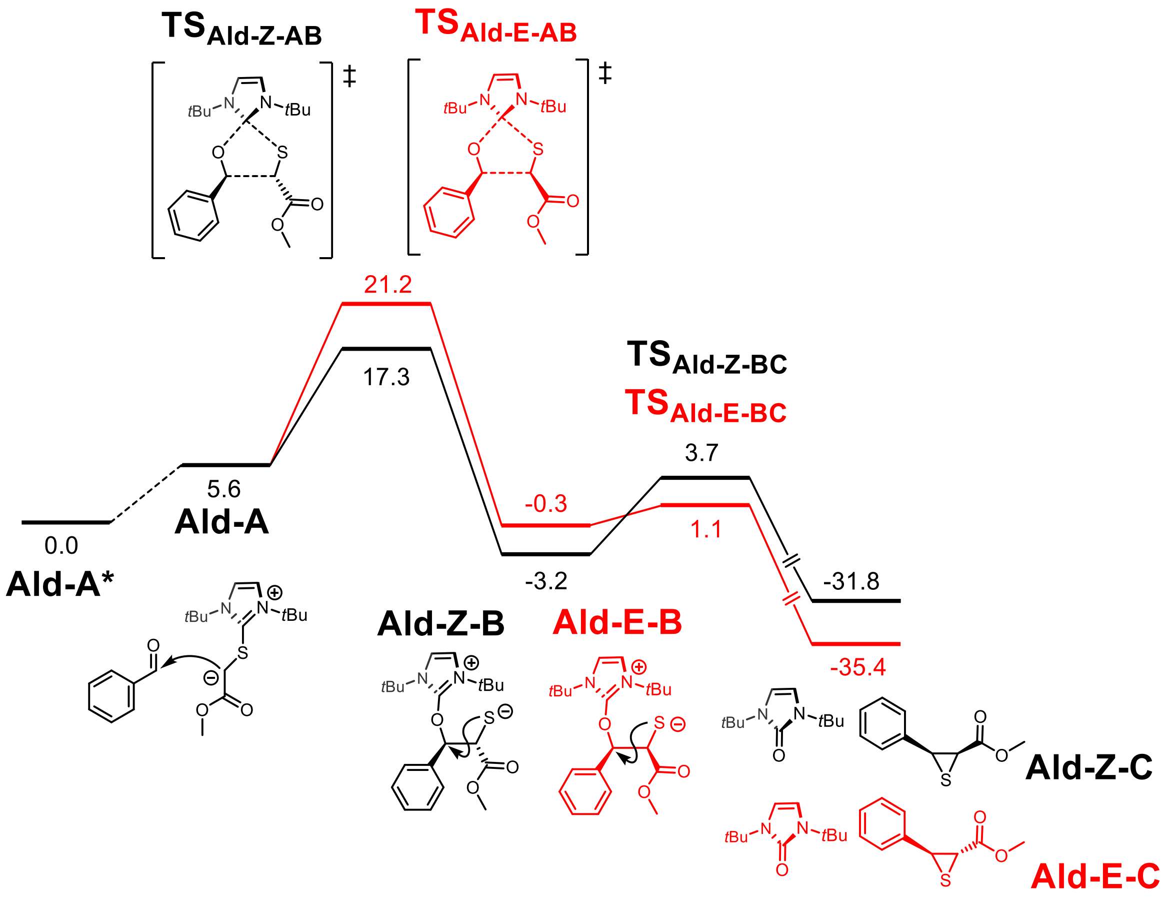

Figure S2. Energy profile of the C–C coupling of aldehyde with thiouronium ylide leading to *cis*-episulfide (black) and *trans*-episulfide (red). Relative Gibbs Free Energies are presented in kcal mol^{-1}. The separated reactants (**Ald-A***) serve as a reference 0.0 kcal mol^{-1}.

For the reaction with tosylimine, the separated reactants (**Tsl-A***) are taken as a reference to present the Gibbs free energy profile of the olefin formation (Figure S3). The formation of the reactant complex (**Tsl-A**) is an endergonic process with ΔG(**Tsl-A*→Tsl-A**) = 4.9 kcal mol^{-1}. Subsequently, the C–C coupling can lead either to the *cis*- or *trans*- intermediate (**Tsl-E-B** and **E-B'**, respectively). The mechanisms leading to both possible episulfides (**Tsl-E-E** and **E-E'**) are equivalent. However, the stabilities of the intermediates

and transition states are significantly different. The profile leading to episulfide **TsI-E-E** (Figure S3, black), which ultimately converts into the experimentally observed olefin, requires a minimal activation barrier to form the C–C bond ($\Delta G^{\ddagger}$(**TsI-A*→TsI-E-B**) = 6.7 kcal mol^{-1}). This step is exergonic (ΔG(**TsI-A*→TsI-E-B**) = -8.5 kcal mol^{-1}). The following step is the endergonic N–C bond formation through a nucleophilic attack of the imine (ΔG(**TsI-E-B→TsI-E-C**) = 3.7 kcal mol^{-1}), which has $\Delta G^{\ddagger}$(**TsI-E-B→TsI-E-C**) = 11.4 kcal mol^{-1} kinetic barrier. Afterwards, an endergonic S–C bond cleavage forms intermediate **TsI-E-D** (ΔG(**TsI-C→TsI-E-D**) = 4.6 kcal mol^{-1}) with the barrier $\Delta G^{\ddagger}$(**TsI-E-C→TsI-E-D**) = 6.3 kcal mol^{-1}. A rotation of the C–C bond allows a nucleophilic attack of the sulfur on the carbon atom bound to the imine, simultaneously breaking the C–N bond to yield the episulfide. This step is highly endergonic (ΔG(**TsI-E-D→ TsI-E-E**) = 22.0 kcal mol^{-1}), and kinetically favored ($\Delta G^{\ddagger}$(**TsI-E-D→TsI-E-E**) = 11.1 kcal mol^{-1}). The apparent activation barrier of this process is the energy gap between the intermediate with the lowest energy and the following transition state with the highest energy (between **TsI-E-B** and $\mathbf{TS_{E\text{-}DE}}$), resulting in $\Delta G^{\ddagger}_{apparent}$ = 19.4 kcal mol^{-1}. The profile leading to the intermediate **TsI-Z-E** (Figure S3, red) starts with a C–C coupling from a conformation that yields the *trans-* intermediate **TsI-E-B** . This step is exergonic (ΔG(**TsI-A*→TsI-Z-B**) = -6.2 kcal mol^{-1}) and presents $\Delta G^{\ddagger}$(**TsI-A*→TsI-Z-B**) = 9.0 kcal mol^{-1} barrier, which is 2.3 kcal mol^{-1} higher than the competing transition state, $\mathbf{TS_{TsI\text{-}E\text{-}AB}}$. Until this point, the formation of the experimentally observed product is more favorable both kinetically and thermodynamically. However, the two following steps, **TsI-Z-B→TsI-Z-C** and **TsI-Z-C→TsI-Z-D**, are slightly endergonic (ΔG(**TsI-Z-B→TsI-Z-C**) = 0.9 kcal mol^{-1} and ΔG(**TsI-Z-C→TsI-Z-D**) = 1.1 kcal mol^{-1}) and have a lower overall activation barrier than the competing profile ($\Delta G^{\ddagger}$(**TsI-Z-B→TsI-Z-D**) = 6.3 kcal mol^{-1}, while $\Delta G^{\ddagger}$(**TsI-E-B→TsI-E-D**) = 8.3 kcal mol^{-1}). The transition state for the last step, $\mathbf{TS_{TsI\text{-}Z\text{-}DE}}$, is less stabilized than $\mathbf{TS_{TsI\text{-}E\text{-}DE}}$ by 3.9 kcal mol^{-1}. The apparent activation barrier for this profile is the energy gap between **TsI-Z-B** and $\mathbf{TS_{TsI\text{-}Z\text{-}DE}}$ ($\Delta G^{\ddagger}_{apparent}$ = 20.8 kcal mol^{-1}), which is 1.4 kcal mol^{-1} higher than for the competing energy profile. The formation of the *trans*-episulfide is therefore kinetically favored, which ultimately leads to the *E*-olefin. This feature contrasts to the energy profile of the reaction with the aldehyde, in which the rate-determining transition state corresponds to the first step, the C–C coupling.

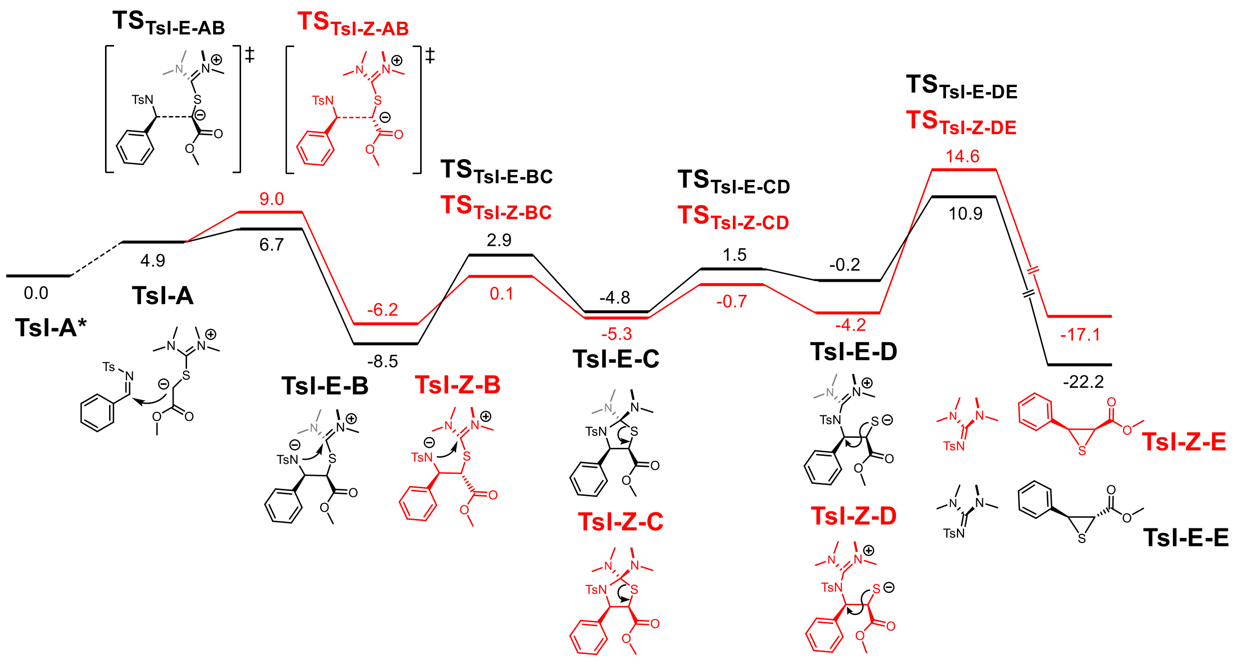

Figure S3. Energy profile of the C–C coupling of tosylimine with thiouronium ylide leading to *trans*-episulfide (black) and *cis*-episulfide (red). Relative Gibbs Free Energies are presented in kcal mol^{-1}. The separated reactants (**TsI-A***) serve as a reference 0.0 kcal mol^{-1}.

Both of the presented energy profiles (Figures S2 and S3) reveal that the first step, the C-C coupling is highly exergonic. The low reversibility of this process strongly suggests that the first transition state is crucial for the selectivity of each substrate. The competing transition state structures were studied using the energy decomposition analysis method, SAPT, which allows the differentiation of the contributions of electrostatics, exchange (repulsion), induction (polarization), and dispersion (London forces) between the two reagents at the point which the C–C bond is formed (Figure S4). For the system with the tosylimine, the transition state that leads to the *trans*-intermediate (**TS$_{\text{TsI-Z-AB}}$**) presents higher attraction forces between the reactants (electrostatics, induction, and dispersion). However, their effect is countered by the significantly higher repulsion forces that mainly derive from the steric clash between the tosyl and acetate groups. The total interaction energy between the reagents in the transition state structure is very similar for both **TS$_{\text{TsI-E-AB}}$** and **TS$_{\text{TsI-Z-AB}}$** (-13.91 and -14.64 kcal mol^{-1}, respectively). However, this interaction equilibrium is achieved at a longer C–C distance in **TS$_{\text{TsI-E-AB}}$**, which leads to the experimentally observed product, suggesting that the selectivity of the *E*-olefin with tosylimines is mainly derived from the tosyl group being bulkier than the aryl moiety of the imine.

For the reaction of the aldehyde, the opposing effect can be observed. Besides the C–C bond formation, the transition state involves more concerted steps. Therefore, the C–C bonding distance is shorter than the tosylimine system, and the substituent groups are closer, increasing the individual contributions of all the energy components. The transition state leading to the *cis*-intermediate (**TS$_{\text{Ald-E-AB}}$**) presents both higher

attractive and repulsive forces. However, the overall interaction energy of both structures is again similar (-7.34 kcal mol^{-1} for $\mathbf{TS_{Ald\text{-}Z\text{-}AB}}$ and -2.66 kcal mol^{-1} for $\mathbf{TS_{Ald\text{-}E\text{-}AB}}$), while the C–C bond is formed at a longer distance for in $\mathbf{TS_{Ald\text{-}Z\text{-}AB}}$ (0.18 Å longer than $\mathbf{TS_{Ald\text{-}E\text{-}AB}}$). This suggests that the reduced bulkiness of the carbonyl (opposed to tosylimine) not only allows the C–C coupling to occur in fewer steps but also favours the formation of a *trans*-intermediate to allow lower steric repulsion between the aryl and the electron-withdrawing substituents.

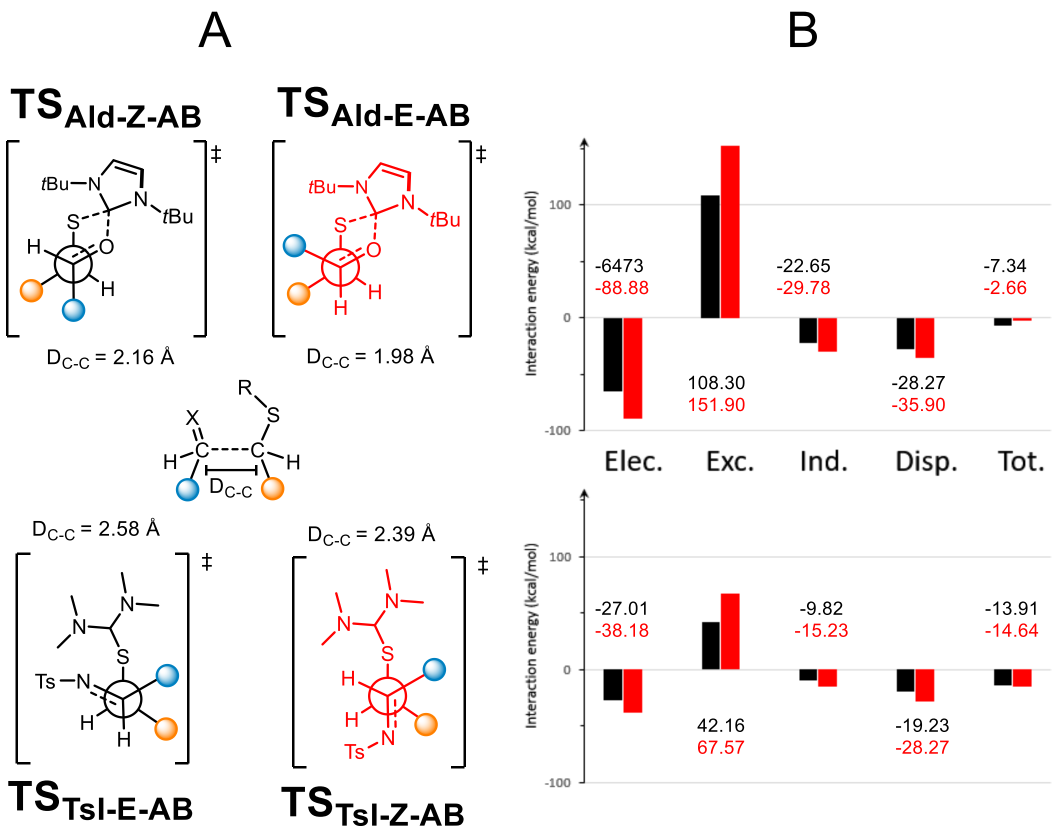

Figure S4. A) Newman representation of the C–C coupling transition states; B) Results of SAPT analysis: Electrostatic (Elec.), Exchange (Exc.), Induction (Ind.), Dispersion (Disp.), and Total (Tot.) energy contributions

All the obtained conformers of $\mathbf{TS_{TsI\text{-}Z\text{-}AB}}$ have an eclipsed geometry, as shown in Figure S5, while the conformers of $\mathbf{TS_{TsI\text{-}E\text{-}AB}}$ present a hyperconjugated conformation of the substituent groups in the vicinity of the C–C bond formation (Figure S6). For ease of representation, the substituent groups of carbons performing the coupling are labeled in pink 1, 2, and 3 for the tosylimine (tosylimine, aryl, and hydro groups, respectively) and 1', 2', and 3' for the thiouronium ylide (thiourea, esther, and hydro groups, respectively).

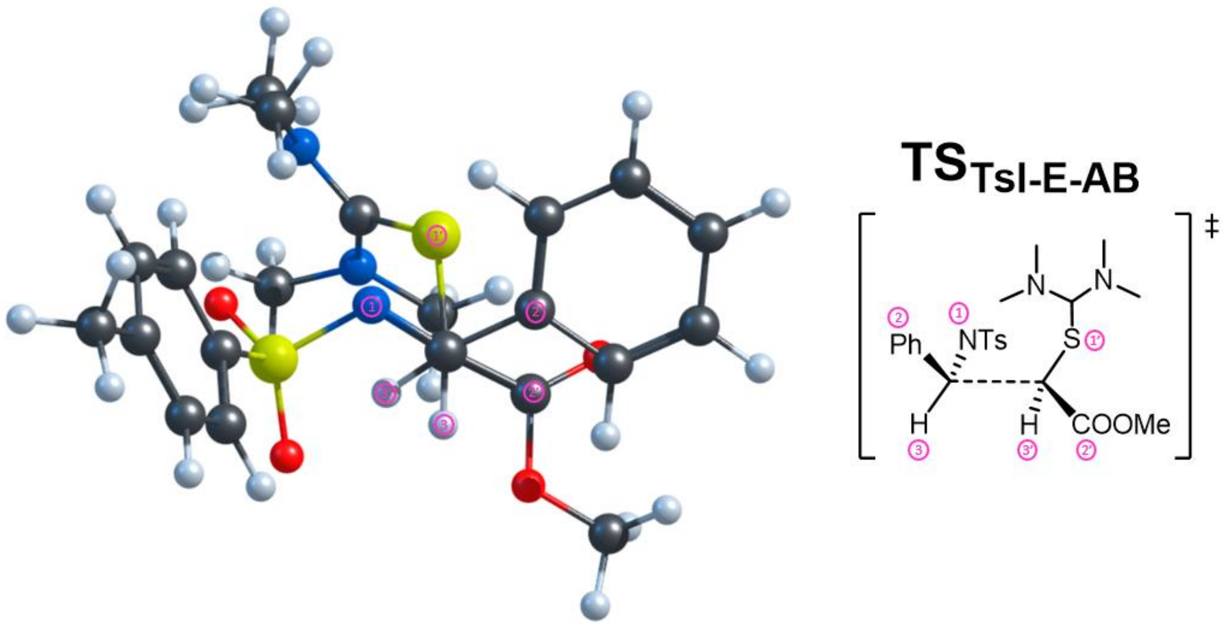

***Figure S5*.** 3D and schematic representations of the geometry of **TS$_{\textbf{TsI-E-AB}}$** with a projection along the C–C bond, substituent groups of each carbon are labeled in pink.

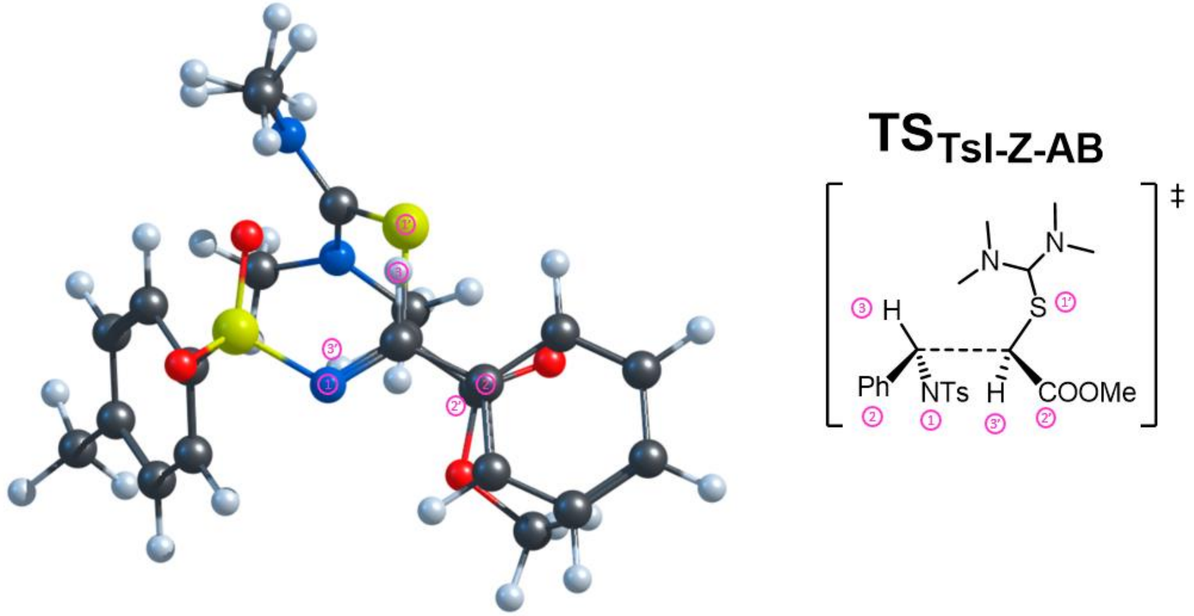

***Figure S6*.** 3D and schematic representations of the geometry of **TS$_{\textbf{TsI-Z-AB}}$** with a projection along the C–C bond, substituent groups of each carbon are labeled in pink.

Given the presented results which show that the step that determines the selectivity of the reaction is the inicial C–C coupling, we briefly probed if substituent group repalcement would have a significant impact on the selectivity of the reaction of the tosyl imine Figure S7-A. For this purpose, we considered the

replacement of the bulky tosyl imine group by a mesyl imine (Figure S7-B) and thiouronium **5a** used in the reaction with the imine by thiouronium **5c**, wich was used in the reaction with the aldehyde (Figure S7-C). The obtained results show that altough some slight variation of the relative activation energy can be observed, the selectivity towards the *E*-olefin should be conserved.

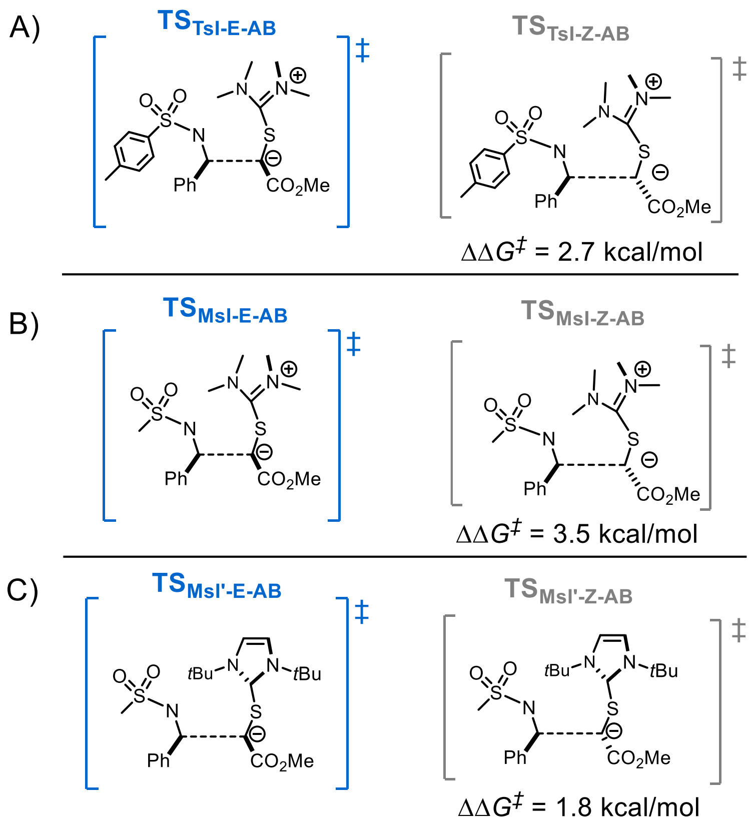

***Figure S7*.** Direct Gibbs free energy comparison between the selectivity-determining transition states with different functional groups. The transition state that leads to the *E*-olefin serves as a reference for each system.

The Gibbs free energy profiles for the conversion of the *cis*-episulfide into the *Z*-olefin and *trans*-episulfide into the *E*-olefin are shown in Figure S8. The intermediate **Z-A** is the product complex analogous to the intermediate **Ald-Z-C** (Figure S2), but with Barton's base instead of the urea. The Barton's base can perform a nucleophilic attack on the sulfur of the *cis*-episulfide to cleave both C-S bonds, releasing the olefin and a sulfonated base. This reaction proceeds stepwise (Figure S8, A), as the nucleophilic attack of Barton's base (step **Z-A**→**Z-B**) leads to a transient intermediate **Z-B** in which only one of the C-S bonds has been cleaved, and the electron-withdrawing group can stabilise the negative charge. This high-energy intermediate is likely stabilised by the interaction of the carbonyl with the hydrogen in the *ortho*-position of the aromatic ring, which is only possible in the Z-configuration of the double bond. The following step is the second C-S bond dissociation that forms the *Z*-olefin. It has a very low activation barrier ($\Delta G^{\ddagger}$(**Z-B**→**Z-D**) = 1.5 kcal mol^{-1}), which does not allow the C–C bond rotation that could potentially lead to the isomerization of the

olefin product. The global activation barrier corresponds to the first step ($\Delta G^{\ddagger}$(**Z-A**→**Z-B**) = 20.3 kcal mol^{-1}), and the overall process is highly endergonic with respect to the isolated products as reference (ΔG(**Z-A**→**Z-C***) = 5.4 kcal mol^{-1}). The formation of the *E*-olefin (Figure S8-B) occurs in a single step, with an activation barrier of 20.5 kcal mol^{-1}. This step is highly endergonic if the formation of the product complex is considered (ΔG(**E-A**→**E-B**) = 9.1 kcal mol^{-1}) and slightly endergonic if the separated products are taken into account (ΔG(**E-A**→**E-B***) = 1.2 kcal mol^{-1}). The computed Gibbs free energy profiles show the importance of an electron-withdrawing group in the presented olefination reaction, especially in the sulfur extrusion process.

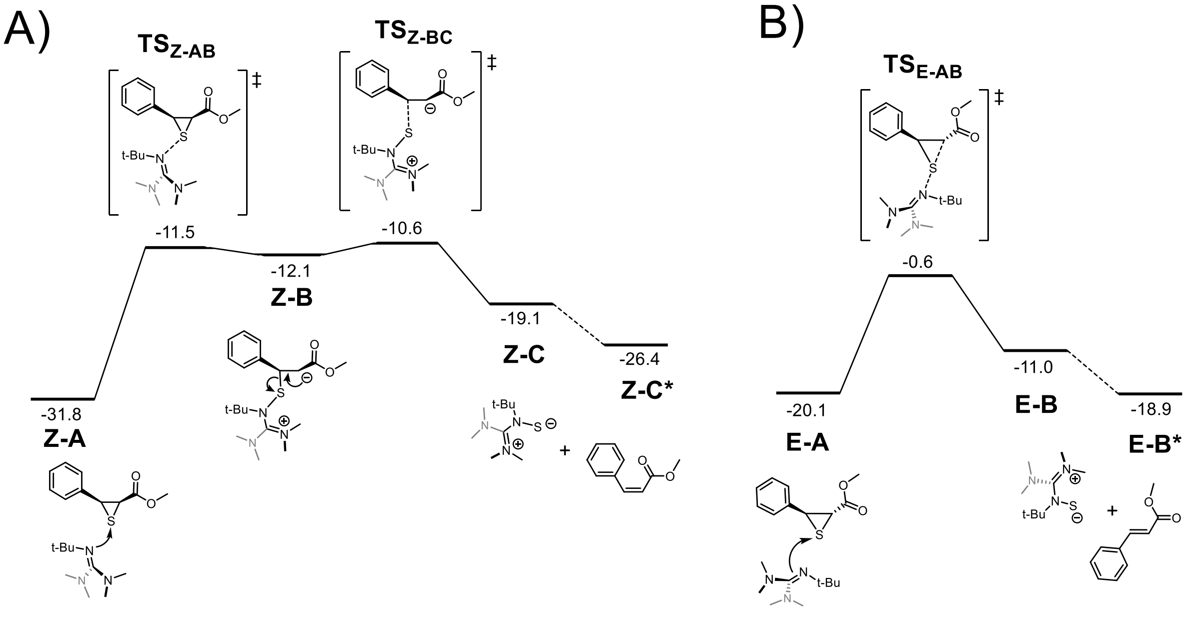

Figure S8. Energy profile of the sulfur extrusion to form: A) *Z*-olefin; B) *E*-olefin. Relative Gibbs free energies are presented in kcal mol^{-1}. The separated reactants (**Ald-A*** and **Tsl-A***) serve as a reference 0.0 kcal mol^{-1}

The mechanism for Barton's base regeneration is presented in Figure S9. The reference (0.0 kcal mol^{-1}) of the computed Gibbs free energy profile shown in Figure S9, **B-A***, is the *cis*-episulfide and the sulfonated base considered separately. The intermediate **B-A** is the respective reactant complex. The nucleophilic attack of the sulfur on the episulfide leads to the disulfonated base and a new *E*-olefin (complex **B-B**). This step is exergonic (ΔG(**B-A***→**B-B**) = -1.1 kcal mol^{-1}) with respect to the reference **B-A*** and has a relatively low activation barrier ($\Delta G^{\ddagger}$(**B-A***→**B-B**) **=** 13.3 kcal mol^{-1}). This event is kinetically more favorable than the nucleophilic attack of the base on the episulfide presented in Figure S8 ($\Delta G^{\ddagger}$(**E-E***→**E-F**) = 20.5 kcal mol^{-1}), which strongly suggests that the sulfonated base can actively perform the sulfur extrusion of the formed episulfides. For the pathway of regeneration of the base by releasing S_2, the removal of the *E*-olefin from the computational model yields structure **B-B***. From this structure, the dissociation of the N-S bond that splits the sulfonated base into Barton's base and the S_2 sulfur allotrope (step **B-B***→**B-C**) has a high

activation barrier ($\Delta G^{\ddagger}$(**B-B*→B-C**) = 31.6 kcal mol^{-1}) and is highly endergonic, even if the isolated products are considered: ΔG(**B-B*→B-C***) = 26.4 kcal mol^{-1}. Thus, the calculations suggest that the S_2 release is very unfavourable. Suppose S_2 sulfur species is not released from the sulfonated base. In that case, the extrusion process can continue until an 8-membered sulfur chain is formed (structure **B-D**), from which the release of S_8 species (step **B-D→B-E**) is a highly exergonic process (ΔG(**B-D→B-E***) = -18.8 kcal mol^{-1}), presenting a marginal activation barrier ($\Delta G^{\ddagger}$(**B-D→B-E**) = 0.7 kcal mol^{-1}). This N-S bond cleavage regenerates Barton's base and forms S_8 in a crown conformation. It may be possible for this bond cleavage to occur prior to the formation of the 8-sulfur chain. However, the presented results clearly show that the sulfur accumulation at the base not only is a favourable process but also facilitates the following extrusion steps.

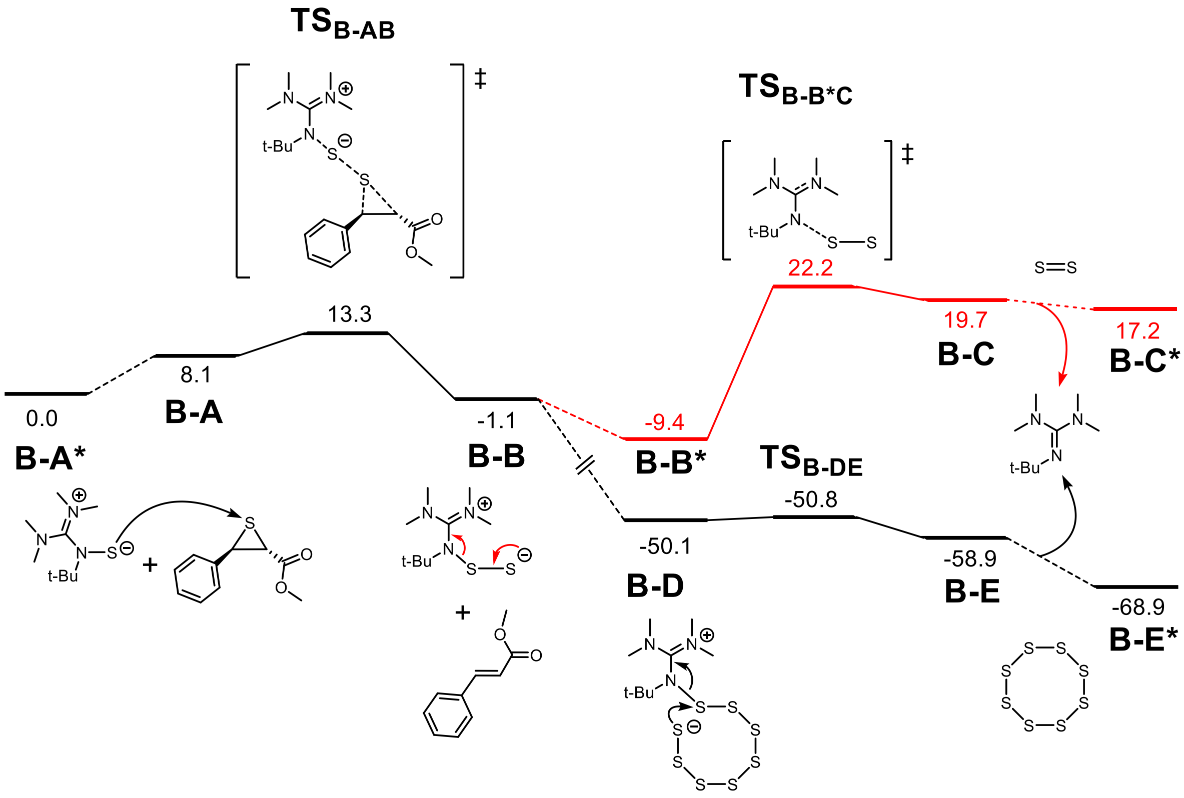

Figure S9. Energy profile for the base regeneration. Relative Gibbs Free Energies are presented in kcal mol^{-1}. The separated reactants (**B-A***) serve as a reference 0.0 kcal mol^{-1}.

3.2.2 XYZ Structures

Cartesian coordinates of the most stable (ΔG298) conformations, computed at the PBE0-D3BJ/def2-TZVP,SMD//PBE0-D3BJ/def2-SVP,SMD level of theory.

43
Ald-Ylide

C	2.071352000	-1.800093000	-0.774444000
N	2.244952000	-2.831970000	-1.643243000
C	3.415923000	-2.638489000	-2.335592000
C	3.969248000	-1.487666000	-1.885538000
N	3.152364000	-0.984443000	-0.897404000
C	3.527463000	0.253154000	-0.129292000
C	2.876976000	1.462178000	-0.797706000
H	1.781969000	1.397932000	-0.769265000
H	3.204232000	1.539426000	-1.845970000
H	3.189613000	2.377818000	-0.273888000
C	3.140467000	0.110460000	1.338584000
H	3.502455000	0.999309000	1.874739000
H	3.619762000	-0.775479000	1.782362000
H	2.060221000	0.035455000	1.493448000
C	5.048598000	0.413652000	-0.182526000
H	5.416782000	0.666142000	-1.186708000
H	5.567822000	-0.486798000	0.178687000
H	5.319924000	1.249006000	0.477586000
H	4.889014000	-1.005480000	-2.193741000
H	3.779660000	-3.324504000	-3.090933000
C	1.356765000	-4.027095000	-1.842552000
C	0.050972000	-3.578710000	-2.493058000
H	-0.611191000	-4.450142000	-2.604047000
H	-0.457929000	-2.807627000	-1.902566000
H	0.250630000	-3.167317000	-3.494199000
C	1.138115000	-4.716302000	-0.496861000
H	0.568752000	-4.091337000	0.202887000
H	0.570548000	-5.644271000	-0.658512000
H	2.102591000	-4.978927000	-0.035866000
C	2.057309000	-5.015767000	-2.770973000
H	2.218795000	-4.603751000	-3.777571000
H	3.018047000	-5.362268000	-2.361669000
H	1.403484000	-5.892359000	-2.877714000
S	0.581171000	-1.598094000	0.210099000
C	0.000000000	0.000000000	0.000000000
C	-0.608865000	0.483964000	-1.182747000
O	-0.969111000	1.639952000	-1.388816000
O	-0.770701000	-0.467211000	-2.159940000
C	-1.376372000	-0.027925000	-3.348398000
H	-1.420292000	-0.896984000	-4.020103000
H	-2.398574000	0.348877000	-3.178205000
H	-0.798379000	0.776230000	-3.832970000
H	0.244204000	0.742673000	0.762328000

14
Ald-Aldehyde

C	0.000000000	0.000000000	0.000000000
O	0.465671000	-0.044399000	1.115124000
C	-0.300023000	-1.182352000	-0.832532000
C	-0.841835000	-1.008042000	-2.111252000
C	-1.129561000	-2.117291000	-2.903533000
C	-0.875011000	-3.400154000	-2.416783000
C	-0.333158000	-3.578730000	-1.139593000
C	-0.045868000	-2.473216000	-0.348063000
H	0.377413000	-2.585496000	0.653129000
H	-0.136767000	-4.586942000	-0.766381000
H	-1.100532000	-4.271430000	-3.037341000
H	-1.552832000	-1.984114000	-3.902070000
H	-1.035618000	0.003976000	-2.479375000
H	-0.240184000	0.978914000	-0.485072000

57
Ald-A

C	2.083176000	-1.806703000	-0.763133000
N	2.263509000	-2.863795000	-1.600170000
C	3.472070000	-2.722247000	-2.238262000
C	4.038606000	-1.576850000	-1.789891000
N	3.191104000	-1.024285000	-0.855878000
C	3.549409000	0.230972000	-0.109256000
C	2.884610000	1.417210000	-0.802811000
H	1.790518000	1.328500000	-0.775139000
H	3.215113000	1.481436000	-1.850855000
H	3.176054000	2.347408000	-0.292760000
C	3.151784000	0.107732000	1.357919000
H	3.496401000	1.009756000	1.883407000
H	3.638706000	-0.764788000	1.819638000
H	2.071028000	0.019862000	1.504627000
C	5.067267000	0.411425000	-0.157769000
H	5.437910000	0.635896000	-1.167707000
H	5.597889000	-0.468167000	0.236690000
H	5.320995000	1.271853000	0.476583000
H	4.985276000	-1.129044000	-2.066998000
H	3.850162000	-3.436429000	-2.959597000
C	1.342303000	-4.031053000	-1.825533000
C	0.070937000	-3.539886000	-2.512438000
H	-0.609870000	-4.391914000	-2.654073000
H	-0.437640000	-2.762671000	-1.930353000
H	0.314594000	-3.124502000	-3.502251000
C	1.060467000	-4.716502000	-0.489327000
H	0.489515000	-4.076552000	0.195030000
H	0.467730000	-5.624460000	-0.672923000
H	2.001301000	-5.012659000	-0.000503000
C	2.036565000	-5.039950000	-2.736601000
H	2.246561000	-4.628588000	-3.734564000
H	2.969261000	-5.425535000	-2.298444000
H	1.354072000	-5.890551000	-2.870295000
S	0.591240000	-1.598017000	0.197259000
C	0.000000000	0.000000000	0.000000000
C	-0.711186000	0.455588000	-1.134722000
O	-1.249933000	1.553176000	-1.256919000
O	-0.764177000	-0.453540000	-2.158506000
C	-1.497078000	-0.068356000	-3.292503000
H	-1.483581000	-0.924115000	-3.982108000
H	-2.541685000	0.183673000	-3.048786000
H	-1.048210000	0.806019000	-3.792477000
H	0.178684000	0.717961000	0.801078000
C	-2.401799000	-0.510877000	1.577629000
O	-2.050954000	-0.571752000	2.736761000
C	-2.708382000	-1.695691000	0.741949000
C	-3.262264000	-1.526765000	-0.531045000
C	-3.560255000	-2.636460000	-1.317674000
C	-3.298252000	-3.920388000	-0.835388000
C	-2.742755000	-4.094579000	0.435779000
C	-2.452234000	-2.985554000	1.224108000
H	-2.019323000	-3.094366000	2.221516000
H	-2.537166000	-5.101543000	0.808308000
H	-3.526600000	-4.792286000	-1.454231000
H	-3.990829000	-2.502107000	-2.313251000
H	-3.451926000	-0.514678000	-0.899094000
H	-2.586533000	0.467035000	1.073818000

57
TS_Ald-Z-AB

C	-0.109963000	0.594293000	-2.078017000
H	-0.170676000	-0.478060000	-2.373480000
O	0.908209000	1.256469000	-2.349081000
C	2.751475000	0.658500000	-0.713952000
N	3.618403000	-0.013493000	-1.516169000
C	3.627648000	-1.476429000	-1.861353000
C	4.522960000	0.879008000	-2.033476000
H	5.318752000	0.595686000	-2.710986000
N	3.138767000	1.957316000	-0.700767000
C	4.221660000	2.100579000	-1.530304000
H	4.703342000	3.056810000	-1.705987000
C	2.550754000	3.185026000	-0.067502000
S	1.692799000	-0.158193000	0.456076000
C	0.000000000	0.000000000	0.000000000
H	-0.511116000	0.807676000	0.531318000
C	-0.723380000	-1.253934000	0.018900000
O	-0.327570000	-2.356204000	-0.325282000
C	2.255105000	-1.877364000	-2.390787000
H	2.322306000	-2.894806000	-2.804320000
H	1.488153000	-1.885684000	-1.605756000
H	1.944814000	-1.191135000	-3.191700000
C	4.035479000	-2.285917000	-0.632098000
H	3.295479000	-2.200510000	0.173337000
H	4.116639000	-3.346365000	-0.913209000
H	5.015454000	-1.954204000	-0.255632000
C	4.659596000	-1.713438000	-2.961867000
H	5.685807000	-1.486542000	-2.637561000
H	4.627518000	-2.780389000	-3.221466000
H	4.431141000	-1.141328000	-3.873322000
C	3.610455000	3.765975000	0.866753000
H	3.216886000	4.678703000	1.337246000
H	3.861036000	3.047917000	1.662531000
H	4.534277000	4.034514000	0.333798000
C	1.278305000	2.915399000	0.716719000
H	0.503235000	2.481344000	0.075470000
H	1.443240000	2.264628000	1.583524000
H	0.911622000	3.886671000	1.079392000
C	2.211435000	4.145616000	-1.210046000
H	1.689356000	5.023495000	-0.803095000
H	3.106639000	4.504071000	-1.737751000
H	1.554680000	3.630422000	-1.925930000
O	-2.006108000	-1.062814000	0.411466000
C	-2.863963000	-2.176797000	0.298046000
H	-2.558513000	-2.991179000	0.974157000
H	-3.867206000	-1.827611000	0.575869000
H	-2.883332000	-2.573731000	-0.729110000
C	-1.462865000	1.240083000	-2.015547000
C	-2.621468000	0.467460000	-2.151438000
C	-1.585837000	2.628063000	-1.880090000
C	-3.880807000	1.064103000	-2.124235000
C	-2.843354000	3.226730000	-1.844436000
C	-3.995826000	2.445880000	-1.962453000
H	-2.530219000	-0.613675000	-2.289025000
H	-0.678295000	3.231391000	-1.807701000
H	-4.778200000	0.448840000	-2.232625000
H	-2.928293000	4.311156000	-1.731628000
H	-4.982674000	2.915773000	-1.938726000

57
Ald-Z-B

C	0.599385000	0.917156000	-1.064132000
H	0.259209000	1.935069000	-0.850457000
O	0.152016000	0.544226000	-2.394345000
C	-1.103136000	0.598207000	-2.774570000
N	-1.826242000	1.722768000	-3.023363000
C	-1.468089000	3.155434000	-2.781974000
C	-3.038185000	1.329189000	-3.555624000
H	-3.831983000	2.029045000	-3.786705000
N	-1.767521000	-0.457901000	-3.298188000
C	-2.996914000	-0.010237000	-3.733563000
H	-3.747844000	-0.677926000	-4.142876000
C	-1.426820000	-1.913085000	-3.347671000
S	-1.819281000	0.054375000	-0.002363000
C	0.000000000	0.000000000	0.000000000
H	0.378305000	-1.024509000	-0.143316000
C	0.460502000	0.406693000	1.374484000
O	0.788906000	-0.360450000	2.247717000
C	-2.308067000	4.023458000	-3.720847000
H	-2.160034000	3.743483000	-4.774773000
H	-3.380309000	3.987176000	-3.484417000
H	-1.985664000	5.066588000	-3.596347000
C	-0.002763000	3.401850000	-3.144021000
H	0.182018000	4.484592000	-3.103746000
H	0.713896000	2.926202000	-2.468617000
H	0.205588000	3.059218000	-4.168662000
C	-1.800030000	3.526059000	-1.336810000
H	-2.890774000	3.522366000	-1.188868000
H	-1.386127000	2.814482000	-0.609693000
H	-1.425915000	4.537795000	-1.120461000
C	-1.602504000	-2.368418000	-4.796913000
H	-0.932993000	-1.807695000	-5.467173000
H	-1.349516000	-3.436039000	-4.869796000
H	-2.635490000	-2.248346000	-5.152936000
C	-2.385233000	-2.647684000	-2.411848000
H	-3.427970000	-2.565466000	-2.754265000
H	-2.121873000	-3.715386000	-2.381844000
H	-2.309594000	-2.214737000	-1.400611000
C	0.008937000	-2.188497000	-2.927576000
H	0.178010000	-1.951901000	-1.871836000
H	0.190179000	-3.264043000	-3.065466000
H	0.732893000	-1.637010000	-3.541332000
O	0.452414000	1.736779000	1.546199000
C	0.831127000	2.208650000	2.825222000
H	0.133642000	1.854998000	3.599937000
H	1.847323000	1.876502000	3.086937000
H	0.800239000	3.304215000	2.773198000
C	2.104225000	0.906872000	-1.092788000
C	2.807902000	2.029125000	-0.641964000
C	2.821525000	-0.223684000	-1.502894000
C	4.201799000	2.023208000	-0.597648000
C	4.214281000	-0.227596000	-1.465448000
C	4.908723000	0.894801000	-1.011003000
H	2.254874000	2.913419000	-0.315850000
H	2.287792000	-1.108026000	-1.855011000
H	4.737184000	2.907115000	-0.241570000
H	4.762214000	-1.115744000	-1.790650000
H	6.001309000	0.888683000	-0.979374000

57
TS_Ald-Z-BC

C	1.237160000	-0.633858000	0.503807000
H	1.836614000	0.116204000	1.022935000
O	2.240007000	-0.666881000	-1.004888000
C	3.455873000	-1.019713000	-0.924830000
N	3.963952000	-2.240161000	-1.262223000
C	3.321402000	-3.365615000	-2.004820000
C	5.304767000	-2.267058000	-0.931312000
H	5.925317000	-3.143990000	-1.087037000
N	4.494987000	-0.268116000	-0.447296000
C	5.630853000	-1.056905000	-0.423723000
H	6.584857000	-0.698987000	-0.052792000
C	4.511361000	1.177350000	-0.069017000
S	-0.888969000	0.115922000	1.592020000
C	0.000000000	0.000000000	0.000000000
H	0.256592000	1.001976000	-0.372321000
C	-0.833825000	-0.647531000	-1.067263000
O	-1.011060000	-1.825896000	-1.276790000
C	1.405874000	-1.970119000	1.121010000
C	3.341601000	-4.597979000	-1.105592000
H	2.751091000	-4.424002000	-0.196044000
H	2.903345000	-5.451234000	-1.643775000
H	4.365243000	-4.874994000	-0.812082000
C	4.148076000	-3.602084000	-3.271092000
H	3.685940000	-4.408176000	-3.859205000
H	4.175070000	-2.693943000	-3.892557000
H	5.181494000	-3.903204000	-3.046356000
C	1.889391000	-3.045776000	-2.411612000
H	1.514945000	-3.905814000	-2.986318000
H	1.224816000	-2.890206000	-1.556142000
H	1.833244000	-2.155792000	-3.052443000
C	3.361775000	1.938264000	-0.723723000
H	3.359907000	1.801361000	-1.814731000
H	2.378720000	1.650055000	-0.338141000
H	3.506766000	3.007689000	-0.514014000
C	5.821899000	1.778465000	-0.584592000
H	5.933794000	1.611318000	-1.666530000
H	5.806654000	2.862448000	-0.403884000
H	6.705238000	1.375444000	-0.070030000
C	4.451466000	1.298014000	1.452008000
H	4.556137000	2.353674000	1.742819000
H	3.494759000	0.932484000	1.851610000
H	5.266245000	0.729770000	1.925667000
O	-1.436719000	0.298026000	-1.795059000
C	-2.351582000	-0.155178000	-2.778001000
H	-1.851159000	-0.793054000	-3.522189000
H	-3.172915000	-0.726709000	-2.319450000
H	-2.751203000	0.742260000	-3.266050000
C	0.477637000	-3.020320000	1.029793000
C	2.590218000	-2.187870000	1.844524000
C	0.725438000	-4.234853000	1.660188000
C	2.842332000	-3.410087000	2.465865000
C	1.906011000	-4.437957000	2.381084000
H	-0.442244000	-2.860384000	0.470419000
H	3.317842000	-1.378628000	1.942138000
H	-0.014610000	-5.036359000	1.589949000
H	3.769895000	-3.552145000	3.026037000
H	2.091983000	-5.395422000	2.874470000

57
Ald-Z-C

C	1.269643000	0.201051000	-0.734827000
H	1.566556000	-0.641109000	-1.368218000
O	-0.895933000	1.151135000	-4.663157000
C	-1.662313000	0.447121000	-4.006288000
N	-2.642160000	0.870950000	-3.125573000
C	-2.930856000	2.294282000	-2.854840000
C	-3.310958000	-0.232884000	-2.614279000
H	-4.122689000	-0.152563000	-1.899847000
N	-1.733678000	-0.934101000	-4.001447000
C	-2.752389000	-1.340194000	-3.153517000
H	-3.000309000	-2.382965000	-2.991732000
C	-0.831240000	-1.800707000	-4.786438000
S	1.478074000	-0.158486000	1.048119000
C	0.000000000	0.000000000	0.000000000
H	-0.624653000	0.874188000	0.209991000
C	-0.751669000	-1.280457000	-0.200427000
O	-0.331890000	-2.247515000	-0.785595000
C	1.648582000	1.539057000	-1.261586000
C	-3.364249000	2.964725000	-4.159295000
H	-2.569751000	2.882588000	-4.913563000
H	-4.276083000	2.488735000	-4.552973000
H	-3.578277000	4.030086000	-3.985128000
C	-1.671443000	2.954994000	-2.294525000
H	-1.855893000	4.023254000	-2.105654000
H	-1.383674000	2.482724000	-1.343615000
H	-0.836154000	2.858559000	-3.000749000
C	-4.051980000	2.397407000	-1.827577000
H	-4.990427000	1.955123000	-2.194512000
H	-3.779583000	1.912724000	-0.877540000
H	-4.241987000	3.460278000	-1.619790000
C	-1.031027000	-1.501885000	-6.272598000
H	-0.815563000	-0.445374000	-6.482949000
H	-0.359485000	-2.128337000	-6.879072000
H	-2.068681000	-1.717023000	-6.572679000
C	-1.161350000	-3.261782000	-4.504761000
H	-2.187931000	-3.518493000	-4.807151000
H	-0.474301000	-3.895397000	-5.084020000
H	-1.030776000	-3.506494000	-3.440052000
C	0.610323000	-1.519933000	-4.359898000
H	0.736835000	-1.748873000	-3.290853000
H	1.304352000	-2.149396000	-4.937095000
H	0.861076000	-0.464867000	-4.532001000
O	-1.934920000	-1.216850000	0.397744000
C	-2.730348000	-2.393822000	0.361175000
H	-2.917699000	-2.708415000	-0.674099000
H	-2.232846000	-3.213220000	0.901454000
H	-3.675963000	-2.141247000	0.855118000
C	1.414911000	2.717393000	-0.540435000
C	2.223904000	1.623934000	-2.533372000
C	1.729844000	3.955048000	-1.092154000
C	2.540203000	2.865228000	-3.086352000
C	2.289678000	4.033933000	-2.370050000
H	0.991973000	2.658941000	0.466191000
H	2.413202000	0.708313000	-3.098997000
H	1.538378000	4.867163000	-0.521130000
H	2.982513000	2.916167000	-4.084464000
H	2.535426000	5.007190000	-2.802374000

57
TS_Ald-E-AB

C	-0.135343000	0.221105000	1.962670000
H	-0.813680000	-0.669187000	1.968952000
O	1.003795000	0.137220000	2.486355000
C	1.771431000	2.256796000	0.087349000
N	2.847129000	2.058755000	0.887331000
C	3.799109000	0.894681000	1.047828000
C	3.158962000	3.247649000	1.492928000
H	3.977446000	3.349615000	2.196734000
N	1.451262000	3.579099000	0.148143000
C	2.300358000	4.191564000	1.029831000
H	2.247094000	5.245654000	1.272475000
C	0.487478000	4.346892000	-0.722386000
S	0.990026000	1.114239000	-0.995955000
C	0.000000000	0.000000000	0.000000000
H	0.515214000	-0.962683000	0.082879000
C	-1.362839000	-0.216871000	-0.478883000
O	-2.020346000	-1.196638000	-0.176723000
C	-0.937139000	1.485353000	2.195814000
C	5.059641000	1.274892000	0.270529000
H	5.515298000	2.200661000	0.652509000
H	5.799520000	0.466715000	0.366072000
H	4.834027000	1.409626000	-0.798569000
C	3.251060000	-0.426049000	0.532137000
H	4.006434000	-1.192592000	0.760161000
H	2.327577000	-0.675873000	1.072869000
H	3.086691000	-0.424198000	-0.551333000
C	4.086320000	0.736068000	2.540679000
H	4.610598000	1.599623000	2.973576000
H	3.138495000	0.564385000	3.069282000
H	4.737106000	-0.139320000	2.677769000
C	0.500725000	5.811225000	-0.293271000
H	0.191787000	5.935429000	0.755126000
H	1.479140000	6.289827000	-0.445317000
H	-0.228203000	6.344739000	-0.918539000
C	-0.930731000	3.815976000	-0.569016000
H	-1.589692000	4.406005000	-1.223428000
H	-1.020399000	2.762480000	-0.852306000
H	-1.279154000	3.938762000	0.464559000
C	0.983386000	4.252136000	-2.164747000
H	0.351889000	4.886744000	-2.803578000
H	2.022102000	4.607712000	-2.244429000
H	0.927918000	3.224424000	-2.548082000
O	-1.886412000	0.784946000	-1.212201000
C	-3.246554000	0.657385000	-1.574028000
H	-3.425566000	-0.270602000	-2.137745000
H	-3.900509000	0.660868000	-0.688036000
H	-3.480910000	1.525402000	-2.203704000
C	-0.301201000	2.584918000	2.778967000
C	-2.316761000	1.548328000	1.964270000
C	-1.018066000	3.735736000	3.103797000
C	-3.038136000	2.697470000	2.285001000
C	-2.390812000	3.796990000	2.854788000
H	0.766908000	2.496156000	2.991424000
H	-2.833195000	0.676191000	1.553337000
H	-0.507020000	4.587974000	3.561241000
H	-4.115664000	2.732516000	2.101582000
H	-2.958262000	4.695086000	3.113031000

57
Ald-E-B

C	0.701618000	0.086863000	-1.330612000
H	1.182601000	-0.879160000	-1.509295000
O	-0.218942000	0.292566000	-2.543195000
C	-1.321823000	-0.370090000	-2.708590000
N	-2.553625000	0.030280000	-2.312347000
C	-3.043001000	1.387020000	-1.885951000
C	-3.443330000	-0.980043000	-2.620914000
H	-4.498772000	-0.920452000	-2.375443000
N	-1.438621000	-1.560814000	-3.341595000
C	-2.755430000	-1.958051000	-3.257533000
H	-3.112200000	-2.900997000	-3.654228000
C	-0.387111000	-2.243616000	-4.165685000
S	1.307437000	-0.327301000	1.243219000
C	0.000000000	0.000000000	0.000000000
H	-0.563948000	0.911671000	0.244702000
C	-0.897432000	-1.192337000	0.123937000
O	-0.803701000	-2.230818000	-0.497212000
C	1.687396000	1.209435000	-1.498687000
C	-4.083838000	1.821834000	-2.921984000
H	-3.645789000	1.840131000	-3.931450000
H	-4.965608000	1.166107000	-2.931872000
H	-4.426741000	2.837138000	-2.676659000
C	-1.930142000	2.426192000	-1.871702000
H	-2.393053000	3.391090000	-1.620158000
H	-1.163642000	2.219406000	-1.118961000
H	-1.443960000	2.526192000	-2.851085000
C	-3.676834000	1.260449000	-0.505405000
H	-4.500127000	0.530218000	-0.504098000
H	-2.938658000	0.947660000	0.244395000
H	-4.091567000	2.235148000	-0.210048000
C	0.055608000	-1.265228000	-5.252148000
H	0.490182000	-0.354806000	-4.816791000
H	0.821761000	-1.743512000	-5.879827000
H	-0.792536000	-0.983120000	-5.894630000
C	-1.009954000	-3.475825000	-4.813115000
H	-1.865462000	-3.222249000	-5.455807000
H	-0.246001000	-3.946281000	-5.447005000
H	-1.326077000	-4.217274000	-4.064242000
C	0.792986000	-2.683610000	-3.304181000
H	0.449086000	-3.144276000	-2.369212000
H	1.391592000	-3.411497000	-3.870258000
H	1.453710000	-1.843121000	-3.060499000
O	-1.853433000	-0.993912000	1.041412000
C	-2.706827000	-2.090418000	1.305978000
H	-3.207546000	-2.438261000	0.389592000
H	-2.145609000	-2.931534000	1.741845000
H	-3.454272000	-1.735163000	2.026482000
C	1.632041000	2.382246000	-0.736909000
C	2.646702000	1.107541000	-2.511636000
C	2.516055000	3.428821000	-0.987634000
C	3.535334000	2.153215000	-2.762885000
C	3.470508000	3.319107000	-2.001656000
H	0.912273000	2.466271000	0.078721000
H	2.700534000	0.193007000	-3.108967000
H	2.465325000	4.336538000	-0.380607000
H	4.282323000	2.053929000	-3.554748000
H	4.165692000	4.140788000	-2.192813000

57
TS-Ald-E-BC

C	0.793876000	0.086790000	-1.246928000
H	1.212579000	-0.881427000	-1.519318000
O	-0.214966000	0.296803000	-2.670066000
C	-1.318052000	-0.326974000	-2.818789000
N	-2.551368000	0.081111000	-2.401729000
C	-3.019612000	1.437967000	-1.975073000
C	-3.459347000	-0.919437000	-2.697715000
H	-4.510443000	-0.847038000	-2.436920000
N	-1.471901000	-1.529995000	-3.439581000
C	-2.794232000	-1.907814000	-3.339972000
H	-3.169389000	-2.848526000	-3.725312000
C	-0.436967000	-2.225702000	-4.261307000
S	1.373497000	-0.354765000	1.162376000
C	0.000000000	0.000000000	0.000000000
H	-0.538080000	0.919952000	0.263127000
C	-0.915515000	-1.183179000	0.079304000
O	-0.821266000	-2.206316000	-0.560813000
C	1.702326000	1.239504000	-1.473421000
C	-4.037220000	1.908509000	-3.018757000
H	-3.582034000	1.936560000	-4.020529000
H	-4.926364000	1.263306000	-3.055487000
H	-4.371786000	2.924474000	-2.763755000
C	-1.885571000	2.452425000	-1.927542000
H	-2.326806000	3.424244000	-1.663041000
H	-1.134991000	2.207971000	-1.170717000
H	-1.380411000	2.555729000	-2.896524000
C	-3.675669000	1.315192000	-0.603059000
H	-4.517874000	0.607023000	-0.617252000
H	-2.954298000	0.975745000	0.152010000
H	-4.068280000	2.296687000	-0.299533000
C	-0.048737000	-1.291148000	-5.406406000
H	0.367980000	-0.350171000	-5.020460000
H	0.714859000	-1.774249000	-6.033942000
H	-0.921405000	-1.061486000	-6.037086000
C	-1.042764000	-3.505811000	-4.826872000
H	-1.914129000	-3.307728000	-5.467808000
H	-0.281009000	-3.999130000	-5.446148000
H	-1.333327000	-4.206920000	-4.030050000
C	0.781903000	-2.588870000	-3.417435000
H	0.479015000	-3.038943000	-2.462867000
H	1.401882000	-3.305190000	-3.975637000
H	1.402563000	-1.708732000	-3.212895000
O	-1.870933000	-0.988819000	0.993569000
C	-2.768264000	-2.064568000	1.203696000
H	-3.288462000	-2.333945000	0.271993000
H	-2.238970000	-2.951777000	1.583199000
H	-3.494745000	-1.720095000	1.949962000
C	1.574753000	2.449725000	-0.777912000
C	2.671138000	1.138672000	-2.479460000
C	2.395661000	3.530048000	-1.085909000
C	3.495550000	2.219786000	-2.787164000
C	3.358213000	3.421006000	-2.092964000
H	0.847883000	2.538853000	0.031156000
H	2.779248000	0.198190000	-3.025956000
H	2.288693000	4.465532000	-0.530742000
H	4.249537000	2.121752000	-3.572428000
H	4.003029000	4.271017000	-2.330602000

57
Ald-E-C

C	1.269643000	0.201051000	-0.734827000
H	1.566556000	-0.641109000	-1.368218000
O	-0.895933000	1.151135000	-4.663157000

C	-1.662313000	0.447121000	-4.006288000
N	-2.642160000	0.870950000	-3.125573000
C	-2.930856000	2.294282000	-2.854840000
C	-3.310958000	-0.232884000	-2.614279000
H	-4.122689000	-0.152563000	-1.899847000
N	-1.733678000	-0.934101000	-4.001447000
C	-2.752389000	-1.340194000	-3.153517000
H	-3.000309000	-2.382965000	-2.991732000
C	-0.831240000	-1.800707000	-4.786438000
S	1.478074000	-0.158486000	1.048119000
C	0.000000000	0.000000000	0.000000000
H	-0.624653000	0.874188000	0.209991000
C	-0.751669000	-1.280457000	-0.200427000
O	-0.331890000	-2.247515000	-0.785595000
C	1.648582000	1.539057000	-1.261586000
C	-3.364249000	2.964725000	-4.159295000
H	-2.569751000	2.882588000	-4.913563000
H	-4.276083000	2.488735000	-4.552973000
H	-3.578277000	4.030086000	-3.985128000
C	-1.671443000	2.954994000	-2.294525000
H	-1.855893000	4.023254000	-2.105654000
H	-1.383674000	2.482724000	-1.343615000
H	-0.836154000	2.858559000	-3.000749000
C	-4.051980000	2.397407000	-1.827577000
H	-4.990427000	1.955123000	-2.194512000
H	-3.779583000	1.912724000	-0.877540000
H	-4.241987000	3.460278000	-1.619790000
C	-1.031027000	-1.501885000	-6.272598000
H	-0.815563000	-0.445374000	-6.482949000
H	-0.359485000	-2.128337000	-6.879072000
H	-2.068681000	-1.717023000	-6.572679000
C	-1.161350000	-3.261782000	-4.504761000
H	-2.187931000	-3.518493000	-4.807151000
H	-0.474301000	-3.895397000	-5.084020000
H	-1.030776000	-3.506494000	-3.440052000
C	0.610323000	-1.519933000	-4.359898000
H	0.736835000	-1.748873000	-3.290853000
H	1.304352000	-2.149396000	-4.937095000
H	0.861076000	-0.464867000	-4.532001000
O	-1.934920000	-1.216850000	0.397744000
C	-2.730348000	-2.393822000	0.361175000
H	-2.917699000	-2.708415000	-0.674099000
H	-2.232846000	-3.213220000	0.901454000
H	-3.675963000	-2.141247000	0.855118000
C	1.414911000	2.717393000	-0.540435000
C	2.223904000	1.623934000	-2.533372000
C	1.729844000	3.955048000	-1.092154000
C	2.540203000	2.865228000	-3.086352000
C	2.289678000	4.033933000	-2.370050000
H	0.991973000	2.658941000	0.466191000
H	2.413202000	0.708313000	-3.098997000
H	1.538378000	4.867163000	-0.521130000
H	2.982513000	2.916167000	-4.084464000
H	2.535426000	5.007190000	-2.802374000

34
Ald-Sideproduct

O	0.000000000	0.000000000	0.000000000
C	-0.382040000	0.011166000	-1.169994000
N	-0.942118000	-1.037210000	-1.877405000
C	-1.235323000	-0.618275000	-3.167116000
C	-0.866240000	0.678889000	-3.275345000
N	-0.341298000	1.074616000	-2.053544000
C	0.192390000	2.405294000	-1.696340000
C	1.661347000	2.254885000	-1.297475000
H	2.078889000	3.233389000	-1.015986000
H	1.758997000	1.569642000	-0.444657000
H	2.249530000	1.857478000	-2.139479000
C	0.084949000	3.336881000	-2.897660000
H	-0.959092000	3.478050000	-3.215702000
H	0.484844000	4.321437000	-2.615313000
H	0.669990000	2.970996000	-3.754928000
C	-0.631139000	2.970786000	-0.538010000
H	-1.684878000	3.087132000	-0.836308000
H	-0.243850000	3.958620000	-0.246070000
H	-0.582645000	2.300254000	0.330520000
H	-0.939207000	1.343908000	-4.129081000
H	-1.682542000	-1.268799000	-3.910948000
C	-1.167992000	-2.377448000	-1.298104000
C	-1.797282000	-3.286236000	-2.347202000
H	-1.144098000	-3.415951000	-3.223318000
H	-1.959363000	-4.277947000	-1.900840000
H	-2.773538000	-2.907951000	-2.686143000
C	-2.115472000	-2.244165000	-0.104750000
H	-1.686158000	-1.574694000	0.652788000
H	-3.086043000	-1.836263000	-0.427996000
H	-2.290016000	-3.230421000	0.351166000
C	0.178155000	-2.958135000	-0.861942000
H	0.655215000	-2.304521000	-0.119241000
H	0.033775000	-3.954414000	-0.417409000
H	0.851471000	-3.060188000	-1.727451000

29
TsI-Ylide

H	0.458324000	5.057293000	1.315998000
C	0.818890000	4.565481000	2.226723000
N	0.563900000	3.138052000	2.162912000
C	0.204337000	2.533211000	1.024216000
N	0.634638000	3.006176000	-0.149552000
C	-0.081735000	2.780065000	-1.385259000
H	-0.096467000	3.720042000	-1.959292000
H	-1.113765000	2.476888000	-1.174380000
H	0.405271000	2.000026000	-1.991298000
C	1.968227000	3.560273000	-0.300040000
H	2.562129000	3.360265000	0.598745000
H	1.949756000	4.643625000	-0.498176000
H	2.456147000	3.058492000	-1.150179000
S	-0.745765000	1.056047000	1.139556000
C	0.000000000	0.000000000	0.000000000
C	1.386922000	-0.220974000	0.130746000
O	2.146050000	0.216189000	1.001002000
O	1.883770000	-1.011655000	-0.875801000
C	3.264176000	-1.269274000	-0.828000000
H	3.554311000	-1.816407000	0.084712000
H	3.860719000	-0.342228000	-0.867841000
H	3.499901000	-1.887080000	-1.706145000
H	-0.536499000	-0.267432000	-0.911400000
C	0.638002000	2.433579000	3.429694000
H	-0.295674000	2.525600000	4.008179000
H	0.856486000	1.375098000	3.244935000
H	1.455656000	2.873991000	4.019587000
H	1.889374000	4.787100000	2.361355000
H	0.268614000	4.978417000	3.086053000

31
TsI-Imine

C	0.000000000	0.000000000	0.000000000
N	0.000535000	1.164886000	0.527353000
S	1.245513000	2.256274000	0.265332000
O	0.631208000	3.384990000	-0.425197000
O	1.865277000	2.457715000	1.570556000
C	2.432114000	1.509608000	-0.816480000
C	3.476713000	0.760906000	-0.274161000
C	4.389825000	0.159429000	-1.133877000
C	4.274709000	0.295293000	-2.525278000
C	3.211241000	1.052625000	-3.039125000
C	2.288248000	1.661374000	-2.195952000
H	1.464425000	2.254937000	-2.597723000
H	3.106839000	1.167825000	-4.121268000
C	5.281393000	-0.327825000	-3.442228000
H	4.829501000	-0.608778000	-4.404530000
H	5.740369000	-1.219357000	-2.991566000
H	6.094511000	0.385710000	-3.659534000
H	5.212291000	-0.427987000	-0.717239000
H	3.571800000	0.658159000	0.808835000
C	-1.081626000	-0.953856000	0.227510000
C	-1.009001000	-2.214465000	-0.382877000
C	-2.023977000	-3.146996000	-0.184647000
C	-3.114581000	-2.823626000	0.622882000
C	-3.192730000	-1.567633000	1.234203000
C	-2.182535000	-0.634830000	1.039414000
H	-2.227761000	0.350350000	1.509285000
H	-4.049569000	-1.320097000	1.865451000
H	-3.912107000	-3.554599000	0.779021000
H	-1.965103000	-4.128543000	-0.660713000
H	-0.149215000	-2.457227000	-1.013501000
H	0.816490000	-0.350209000	-0.655562000

60
TsI-A

C	2.977001000	-0.161141000	3.159194000
N	3.058006000	0.951937000	3.787277000
S	4.249650000	2.068097000	3.453980000
O	3.596124000	3.157914000	2.725852000
O	4.878320000	2.367739000	4.736927000
C	5.465561000	1.332072000	2.394807000
C	6.648055000	0.857697000	2.962623000
C	7.609502000	0.283349000	2.137690000
C	7.404397000	0.172438000	0.754720000
C	6.199454000	0.648467000	0.216951000
C	5.226558000	1.230132000	1.023474000
H	4.273596000	1.555870000	0.603498000
H	6.017372000	0.555163000	-0.857095000
C	8.456395000	-0.419501000	-0.131916000
H	8.011670000	-0.928228000	-0.999538000
H	9.087775000	-1.135537000	0.413454000
H	9.119183000	0.371537000	-0.522493000
H	8.540154000	-0.087337000	2.575474000
H	6.808778000	0.948015000	4.038665000
C	1.894085000	-1.105847000	3.423531000
C	1.746145000	-2.218550000	2.584018000
C	0.695371000	-3.110204000	2.782692000

C -0.206135000 -2.901079000 3.826786000
C -0.056666000 -1.799677000 4.675267000
C 0.986973000 -0.904234000 4.475547000
H 1.116624000 -0.039490000 5.130133000
H -0.761346000 -1.642257000 5.495477000
H -1.031226000 -3.600796000 3.982984000
H 0.576571000 -3.969571000 2.118592000
H 2.449167000 -2.366439000 1.760825000
H 3.712586000 -0.469202000 2.402807000
H 0.460626000 5.123178000 1.071819000
C 0.720370000 4.716907000 2.055723000
N 0.429753000 3.295999000 2.109987000
C 0.203620000 2.577605000 1.006381000
N 0.693072000 2.984817000 -0.171871000
C 0.034760000 2.693655000 -1.425457000
H 0.012378000 3.615754000 -2.029089000
H -0.996141000 2.366603000 -1.248305000
H 0.566896000 1.913911000 -1.992554000
C 2.031708000 3.537667000 -0.286608000
H 2.568631000 3.433775000 0.663418000
H 2.014500000 4.595592000 -0.594278000
H 2.575862000 2.963002000 -1.052230000
S -0.667612000 1.059072000 1.185627000
C 0.000000000 0.000000000 0.000000000
C 1.392084000 -0.154309000 -0.094789000
O 2.267462000 0.375399000 0.602532000
O 1.756314000 -0.988372000 -1.121828000
C 3.136361000 -1.117852000 -1.354349000
H 3.675373000 -1.510953000 -0.477108000
H 3.598061000 -0.156811000 -1.638031000
H 3.247558000 -1.825191000 -2.188298000
H -0.654054000 -0.341674000 -0.803303000
C 0.298985000 2.766510000 3.452615000
H -0.662547000 3.066129000 3.901486000
H 0.365065000 1.674080000 3.436189000
H 1.121225000 3.162349000 4.063719000
H 1.781016000 4.912397000 2.273992000
H 0.106004000 5.222558000 2.815721000

60
TS_TsI-E-AB
C -1.224619000 -0.911509000 2.076873000
H -0.222358000 -0.732192000 2.489066000
N -1.598761000 -2.094034000 1.686778000
H 1.500984000 -1.168609000 -4.790189000
C -0.634647000 -0.970231000 -2.518384000
N 0.519989000 -0.519304000 -3.017242000
C 0.992754000 0.832788000 -2.795638000
H 0.206788000 1.453356000 -2.350091000
H 1.289127000 1.259720000 -3.767364000
H 1.867885000 0.837914000 -2.127531000
C 1.451371000 -1.389820000 -3.712502000
H 1.172081000 -2.439712000 -3.567465000
H 2.451968000 -1.235847000 -3.279927000
H -3.279720000 -2.440002000 -2.525213000
N -1.302453000 -1.960100000 -3.124997000
C -1.260278000 -2.142795000 -4.565456000
H -0.803029000 -1.272631000 -5.049501000
H -0.707737000 -3.052309000 -4.848344000
C -2.247551000 -2.813684000 -2.424137000
H -2.197146000 -3.817523000 -2.871588000
H -1.975982000 -2.884843000 -1.364146000
H -2.294403000 -2.237420000 -4.930225000
S -1.328598000 -0.327170000 -1.046529000
C 0.000000000 0.000000000 0.000000000
H 0.663839000 -0.819846000 0.272618000
C 0.317627000 1.341335000 0.327067000
O -0.226747000 2.366931000 -0.069280000
S -0.405700000 -3.241194000 1.577055000
O -0.407827000 -3.742017000 0.198877000
O 0.872345000 -2.775169000 2.135336000
C -1.038720000 -4.518389000 2.626388000
C -0.583780000 -4.614476000 3.938770000
C -1.998917000 -5.399599000 2.126649000
C -1.106012000 -5.608658000 4.762838000
C -2.511216000 -6.382671000 2.964334000
C -2.075733000 -6.504114000 4.293884000
H 0.174573000 -3.919716000 4.305538000
H -2.332438000 -5.315974000 1.090203000
H -0.751700000 -5.692232000 5.793668000
H -3.263795000 -7.076765000 2.580027000
C -2.633494000 -7.577195000 5.178292000
H -2.366212000 -8.576479000 4.797407000
H -2.253963000 -7.491487000 6.205868000
H -3.733577000 -7.531460000 5.209518000
O 1.342750000 1.402684000 1.231658000
C 1.732827000 2.696363000 1.629386000
H 2.076450000 3.300491000 0.773700000
H 0.911595000 3.241699000 2.121708000
H 2.560998000 2.569140000 2.339885000
C -2.196060000 0.174533000 2.198128000
C -3.516864000 0.020776000 1.748325000
C -1.801491000 1.390410000 2.776180000
C -4.422548000 1.069377000 1.872498000
C -2.711022000 2.435656000 2.902074000
C -4.022521000 2.277767000 2.449128000
H -3.815191000 -0.930727000 1.303311000
H -0.769904000 1.508431000 3.117223000
H -5.449537000 0.946656000 1.519038000
H -2.396141000 3.380470000 3.352025000
H -4.736513000 3.099833000 2.545475000

60
TsI-E-B
C -0.290348000 -1.529601000 0.122355000
H 0.593796000 -1.926569000 0.656508000
N -0.405175000 -2.046469000 -1.206565000
H 2.514030000 -0.714141000 -4.323991000
C -0.075057000 0.036691000 -2.721878000
N 1.260032000 0.083742000 -2.805000000
C 2.068315000 1.116078000 -2.193713000
H 1.434284000 1.921658000 -1.804637000
H 2.742716000 1.536267000 -2.956769000
H 2.690803000 0.706775000 -1.381146000
C 2.001634000 -1.015962000 -3.397087000
H 1.333330000 -1.865920000 -3.579609000
H 2.757148000 -1.343434000 -2.665684000
H -2.914204000 -0.193511000 -3.722963000
N -0.804819000 -0.402440000 -3.743400000
C -0.304267000 -0.397683000 -5.104660000
H 0.546748000 0.287069000 -5.194202000
H -0.002568000 -1.404837000 -5.431556000
C -2.134023000 -0.953762000 -3.560400000
H -2.271548000 -1.762391000 -4.291572000
H -2.192952000 -1.380679000 -2.552452000
H -1.111416000 -0.043820000 -5.762825000
S -0.929415000 0.768931000 -1.366170000
C 0.000000000 0.000000000 0.000000000
H 1.072735000 0.116976000 -0.197030000
C -0.336477000 0.758109000 1.259150000
O -1.253671000 1.531401000 1.378409000
C -1.504412000 -1.813973000 0.993966000
S 0.190399000 -3.472285000 -1.503530000
O 0.114778000 -3.698369000 -2.962561000
O 1.501270000 -3.729012000 -0.863511000
C -0.935690000 -4.658335000 -0.770433000
C -0.640400000 -5.237997000 0.460729000
C -2.173259000 -4.888863000 -1.375238000
C -1.595278000 -6.029410000 1.098519000
C -3.117034000 -5.681467000 -0.731425000
C -2.851658000 -6.253729000 0.523144000
H 0.337056000 -5.060075000 0.914183000
H -2.390241000 -4.443309000 -2.348941000
H -1.361161000 -6.478427000 2.068115000
H -4.085846000 -5.859478000 -1.207354000
C -3.898468000 -7.055414000 1.236901000
H -4.501758000 -7.649957000 0.534586000
H -3.452450000 -7.734852000 1.977525000
H -4.593273000 -6.389680000 1.777444000
C -2.801142000 -1.723995000 0.475645000
C -1.341045000 -2.186231000 2.332784000
C -3.908023000 -1.998008000 1.276965000
C -2.446563000 -2.464869000 3.137783000
C -3.734695000 -2.371831000 2.611268000
H -2.935785000 -1.448417000 -0.571840000
H -0.331519000 -2.272033000 2.744548000
H -4.914362000 -1.926383000 0.855700000
H -2.299334000 -2.766451000 4.178317000
H -4.602682000 -2.595400000 3.237079000
O 0.500765000 0.432034000 2.234701000
C 0.227510000 0.985652000 3.517280000
H 0.293500000 2.083052000 3.488857000
H -0.775577000 0.692008000 3.859402000
H 0.989252000 0.580528000 4.193337000

60
TS_TsI-E-BC
C 0.009225000 -1.561037000 0.074658000
H 0.980128000 -1.840251000 0.519037000
N -0.102846000 -2.025862000 -1.288436000
H 2.950296000 -0.939152000 -3.914130000
C -0.018067000 -0.609447000 -2.559075000
N 1.305493000 -0.295494000 -2.787661000
C 1.969371000 0.880204000 -2.280220000
H 1.244724000 1.666254000 -2.037430000
H 2.645171000 1.267943000 -3.059729000
H 2.588620000 0.663347000 -1.389236000
C 2.226490000 -1.337630000 -3.185767000
H 1.700366000 -2.187853000 -3.628472000
H 2.782291000 -1.714499000 -2.307341000
H -2.836500000 -0.882692000 -3.895881000
N -0.744485000 -1.009019000 -3.642974000
C -0.197114000 -1.078177000 -4.974160000

H 0.643934000 -0.382339000 -5.079501000
H 0.138111000 -2.098425000 -5.231872000
C -2.066371000 -1.563031000 -3.492619000
H -2.131513000 -2.520437000 -4.032819000
H -2.269935000 -1.754603000 -2.434637000
H -0.979680000 -0.782353000 -5.690441000
S -0.932435000 0.506752000 -1.459132000
C 0.000000000 0.000000000 0.000000000
H 1.038025000 0.346308000 -0.090850000
C -0.581053000 0.651196000 1.230540000
O -1.644859000 1.213261000 1.286893000
C -1.081978000 -2.067151000 1.001737000
S 0.341489000 -3.543572000 -1.616129000
O 0.249910000 -3.720297000 -3.073784000
O 1.607124000 -3.907726000 -0.956885000
C -0.901919000 -4.607722000 -0.911861000
C -0.688558000 -5.193521000 0.334414000
C -2.097895000 -4.812181000 -1.598037000
C -1.697530000 -5.958476000 0.910514000
C -3.097175000 -5.583340000 -1.011467000
C -2.921896000 -6.155978000 0.255985000
H 0.263489000 -5.042722000 0.845855000
H -2.242690000 -4.370885000 -2.584981000
H -1.533714000 -6.410146000 1.892877000
H -4.036610000 -5.743645000 -1.547606000
C -4.020392000 -6.934750000 0.912981000
H -4.779763000 -7.257056000 0.186530000
H -3.630269000 -7.823476000 1.431552000
H -4.527294000 -6.315124000 1.672610000
C -2.418828000 -2.112528000 0.595106000
C -0.757070000 -2.461541000 2.302927000
C -3.408962000 -2.557678000 1.468196000
C -1.746181000 -2.902862000 3.181964000
C -3.075995000 -2.956125000 2.764709000
H -2.679032000 -1.802589000 -0.418220000
H 0.287251000 -2.432123000 2.625572000
H -4.448905000 -2.597320000 1.133762000
H -1.474647000 -3.217075000 4.193169000
H -3.853201000 -3.310351000 3.446913000
O 0.252571000 0.520071000 2.255161000
C -0.205875000 1.003485000 3.512246000
H -0.396187000 2.085669000 3.467111000
H -1.129072000 0.486301000 3.812212000
H 0.593833000 0.792060000 4.231445000

60
TsI-E-C
C 0.174028000 -1.511094000 0.288473000
H 1.197236000 -1.659887000 0.660168000
N 0.015875000 -2.170839000 -1.010623000
H 2.417966000 -0.090419000 -1.685947000
C -0.269217000 -1.388175000 -2.211589000
N 0.903102000 -0.984810000 -2.901867000
C 0.755155000 0.117861000 -3.820701000
H -0.257168000 0.154826000 -4.240009000
H 1.465755000 0.011275000 -4.656670000
H 0.942976000 1.090566000 -3.325360000
C 2.198906000 -1.005657000 -2.272415000
H 2.968198000 -1.075645000 -3.058319000
H 2.321609000 -1.878971000 -1.624639000
H -3.129952000 -1.244434000 -2.901381000
N -1.176476000 -2.081349000 -3.050983000
C -0.917403000 -2.315634000 -4.447061000
H 0.161643000 -2.400025000 -4.616022000
H -1.385611000 -3.270185000 -4.737283000
C -2.555993000 -2.160216000 -2.654239000
H -3.031347000 -3.006573000 -3.171274000
H -2.643959000 -2.339965000 -1.574619000
H -1.327728000 -1.532665000 -5.115384000
S -1.148423000 0.177221000 -1.378237000
C 0.000000000 0.000000000 0.000000000
H 0.981885000 0.413647000 -0.275303000
C -0.450567000 0.759370000 1.227002000
O -1.461224000 1.404235000 1.328395000
C -0.781909000 -2.034815000 1.340503000
S 0.443665000 -3.784257000 -1.163417000
O 1.098783000 -3.985590000 -2.451220000
O 1.169081000 -4.097921000 0.066777000
C -1.039508000 -4.750688000 -1.167820000
C -1.687434000 -5.029684000 0.037081000
C -1.512787000 -5.259724000 -2.373978000
C -2.845386000 -5.797235000 0.016209000
C -2.671102000 -6.032717000 -2.374601000
C -3.361624000 -6.304889000 -1.186732000
H -1.285114000 -4.658195000 0.980659000
H -0.972416000 -5.053259000 -3.298572000
H -3.358252000 -6.014149000 0.957227000
H -3.046252000 -6.435127000 -3.319238000
C -4.620025000 -7.117425000 -1.188692000
H -4.822772000 -7.546558000 -2.179700000
H -4.562797000 -7.939265000 -0.457922000
H -5.484873000 -6.495997000 -0.903374000

C -2.168804000 -1.906626000 1.200651000
C -0.271161000 -2.650658000 2.486683000
C -3.027178000 -2.398525000 2.182642000
C -1.127723000 -3.148578000 3.467839000
C -2.509836000 -3.027163000 3.315801000
H -2.571182000 -1.406051000 0.317645000
H 0.810718000 -2.755058000 2.597897000
H -4.108077000 -2.292914000 2.059114000
H -0.713868000 -3.636624000 4.354011000
H -3.183133000 -3.420151000 4.082066000
O 0.449074000 0.623072000 2.197068000
C 0.122654000 1.204070000 3.453015000
H -0.023828000 2.289710000 3.356735000
H -0.793313000 0.750667000 3.860357000
H 0.970081000 0.996140000 4.116635000

60
TS_TsI-E-CD
C -0.782077000 -1.279829000 -0.352758000
H -0.549050000 -2.024884000 0.420519000
N -0.228164000 -1.794501000 -1.611564000
H 1.432573000 -0.260299000 -5.385552000
C -0.082857000 -0.937062000 -2.742778000
N 1.107100000 -0.980943000 -3.414809000
C 2.367493000 -0.800803000 -2.744863000
H 2.211870000 -0.638610000 -1.673913000
H 2.894488000 0.081603000 -3.148219000
H 3.006439000 -1.688184000 -2.884915000
C 1.186181000 -1.187462000 -4.836900000
H 0.238313000 -1.583950000 -5.218252000
H 1.975545000 -1.927382000 -5.046016000
H -3.260542000 -1.062898000 -3.129798000
N -1.205484000 -0.768969000 -3.512714000
C -1.324289000 0.413499000 -4.323942000
H -0.427627000 1.036612000 -4.220560000
H -1.456020000 0.165196000 -5.391034000
C -2.367540000 -1.616024000 -3.465998000
H -2.575590000 -2.021926000 -4.472256000
H -2.203864000 -2.452763000 -2.781431000
H -2.196630000 1.008396000 -3.998950000
S 0.103502000 1.056699000 -1.453017000
C 0.000000000 0.000000000 0.000000000
H 1.013783000 -0.337992000 0.284033000
C -0.557548000 0.572474000 1.286519000
S 0.667216000 -3.231191000 -1.483323000
O 1.046025000 -3.644881000 -2.828531000
O 1.691784000 -3.058276000 -0.456089000
C -0.512734000 -4.397049000 -0.870974000
C -0.472674000 -4.776661000 0.467886000
C -1.451272000 -4.936548000 -1.753277000
C -1.415062000 -5.689299000 0.936078000
C -2.384106000 -5.842176000 -1.266813000
C -2.388492000 -6.227215000 0.084613000
H 0.284426000 -4.357080000 1.133135000
H -1.446375000 -4.652773000 -2.807372000
H -1.394374000 -5.988757000 1.986908000
H -3.126370000 -6.265323000 -1.948823000
C -3.414761000 -7.192465000 0.591208000
H -3.252426000 -7.436537000 1.649893000
H -4.428308000 -6.772544000 0.486305000
H -3.395511000 -8.128395000 0.010752000
C -2.296844000 -1.132819000 -0.351409000
C -2.942992000 0.076509000 -0.633755000
C -3.079907000 -2.248953000 -0.032599000
C -4.335580000 0.159002000 -0.616518000
C -4.471400000 -2.172384000 -0.028630000
C -5.106513000 -0.966107000 -0.324933000
H -2.337494000 0.949939000 -0.890200000
H -2.595318000 -3.193299000 0.220887000
H -4.821171000 1.112860000 -0.839633000
H -5.060741000 -3.059420000 0.218318000
H -6.197759000 -0.901502000 -0.319228000
O -0.768287000 -0.115748000 2.258783000
O -0.760494000 1.881415000 1.262330000
C -1.277946000 2.457766000 2.453387000
H -0.598282000 2.286749000 3.301259000
H -1.371444000 3.532723000 2.258136000
H -2.263663000 2.032604000 2.694781000

60
TsI-E-D
C -0.806256000 -1.244904000 -0.433281000
H -0.634810000 -2.002440000 0.344353000
N -0.210294000 -1.820797000 -1.656329000
H 1.231972000 -0.171277000 -5.445594000
C -0.159884000 -1.156686000 -2.876010000
N 1.002994000 -1.043653000 -3.526545000
C 2.256854000 -0.824512000 -2.839708000
H 2.049223000 -0.347247000 -1.872521000
H 2.856996000 -0.119887000 -3.434704000
H 2.821662000 -1.762005000 -2.726415000

C	1.082812000	-1.152596000	-4.966161000
H	0.174495000	-1.618062000	-5.365317000
H	1.940222000	-1.795319000	-5.216258000
H	-3.354948000	-1.210108000	-3.100819000
N	-1.313601000	-0.887122000	-3.514890000
C	-1.436958000	0.302187000	-4.325165000
H	-0.621162000	0.992083000	-4.076973000
H	-1.433242000	0.080733000	-5.405028000
C	-2.471222000	-1.751652000	-3.468761000
H	-2.684454000	-2.124033000	-4.485394000
H	-2.281768000	-2.607328000	-2.814782000
H	-2.391645000	0.792512000	-4.076352000
S	0.162977000	1.263884000	-1.277391000
C	0.000000000	0.000000000	0.000000000
H	1.000982000	-0.400252000	0.247658000
C	-0.549346000	0.393747000	1.361604000
S	0.708038000	-3.241918000	-1.409474000
O	1.141397000	-3.698145000	-2.724035000
O	1.684687000	-2.979828000	-0.359205000
C	-0.481249000	-4.386849000	-0.784735000
C	-0.501940000	-4.679438000	0.576618000
C	-1.374719000	-4.986886000	-1.675058000
C	-1.458153000	-5.571225000	1.055785000
C	-2.322146000	-5.870086000	-1.176457000
C	-2.385964000	-6.171173000	0.194823000
H	0.218540000	-4.210588000	1.249615000
H	-1.324527000	-4.766637000	-2.743174000
H	-1.485264000	-5.804023000	2.123146000
H	-3.029043000	-6.341747000	-1.864079000
C	-3.427537000	-7.113400000	0.712585000
H	-3.306423000	-7.298370000	1.788571000
H	-4.437444000	-6.705052000	0.545167000
H	-3.382132000	-8.079059000	0.184818000
C	-2.312844000	-1.061578000	-0.481893000
C	-2.911593000	0.166089000	-0.787174000
C	-3.134674000	-2.152433000	-0.171197000
C	-4.301410000	0.289298000	-0.803185000
C	-4.522678000	-2.033795000	-0.200566000
C	-5.112486000	-0.810573000	-0.522261000
H	-2.256787000	1.012833000	-1.023447000
H	-2.682195000	-3.109128000	0.098056000
H	-4.754588000	1.255269000	-1.042776000
H	-5.145562000	-2.899691000	0.038996000
H	-6.201231000	-0.713283000	-0.543716000
O	-0.697030000	-0.412161000	2.255710000
O	-0.841519000	1.678244000	1.500923000
C	-1.367565000	2.065059000	2.760628000
H	-0.656511000	1.845968000	3.571230000
H	-1.545316000	3.145754000	2.700876000
H	-2.312866000	1.540873000	2.968207000

60
TS_TsI-E-DE

C	-0.822978000	-1.179904000	-0.317226000
H	-0.568510000	-2.059987000	0.274099000
N	-0.069366000	-1.927885000	-1.997914000
H	1.054967000	0.174736000	-5.694089000
C	-0.147357000	-1.289910000	-3.192996000
N	0.945874000	-0.865357000	-3.845725000
C	2.207353000	-0.612638000	-3.181095000
H	2.055116000	-0.405405000	-2.118852000
H	2.673458000	0.269281000	-3.645018000
H	2.889339000	-1.470114000	-3.290595000
C	1.015035000	-0.850475000	-5.294142000
H	0.158738000	-1.378447000	-5.727241000
H	1.932309000	-1.378400000	-5.596971000
H	-3.343039000	-1.596099000	-3.301569000
N	-1.355824000	-1.073998000	-3.744035000
C	-1.657951000	0.094522000	-4.542283000
H	-0.884929000	0.860102000	-4.407432000
H	-1.749837000	-0.143749000	-5.614164000
C	-2.413444000	-2.057630000	-3.660183000
H	-2.593624000	-2.481298000	-4.662977000
H	-2.125051000	-2.860922000	-2.977131000
H	-2.620260000	0.504730000	-4.198249000
S	-0.895671000	0.389649000	1.546855000
C	0.000000000	0.000000000	0.000000000
H	-0.034944000	0.802960000	-0.748591000
C	1.437184000	-0.263642000	0.351072000
S	0.910210000	-3.271056000	-1.909237000
O	1.088706000	-3.793485000	-3.269026000
O	2.107441000	-3.029013000	-1.106959000
C	-0.121181000	-4.391574000	-1.005076000
C	0.034071000	-4.510495000	0.373450000
C	-1.097545000	-5.117740000	-1.689332000
C	-0.823597000	-5.352703000	1.078383000
C	-1.941222000	-5.954739000	-0.970395000
C	-1.825274000	-6.080500000	0.423982000
H	0.811971000	-3.941862000	0.886929000
H	-1.186158000	-5.029623000	-2.773979000
H	-0.711453000	-5.445822000	2.161647000
H	-2.708616000	-6.526230000	-1.499590000
C	-2.753253000	-6.979723000	1.181540000
H	-2.544270000	-6.960592000	2.259955000
H	-3.802074000	-6.679421000	1.027711000
H	-2.664585000	-8.020653000	0.831168000
C	-2.256500000	-1.066522000	-0.652262000
C	-2.814540000	0.103606000	-1.188644000
C	-3.085017000	-2.173011000	-0.425092000
C	-4.174169000	0.164782000	-1.473569000
C	-4.448637000	-2.109074000	-0.706186000
C	-4.998858000	-0.939263000	-1.229199000
H	-2.183400000	0.977687000	-1.360063000
H	-2.650051000	-3.087046000	-0.011957000
H	-4.600310000	1.084184000	-1.883433000
H	-5.083660000	-2.977561000	-0.513361000
H	-6.068356000	-0.883325000	-1.447422000
O	2.350095000	0.461196000	0.020301000
O	1.604451000	-1.341430000	1.108968000
C	2.932518000	-1.640006000	1.497313000
H	3.348042000	-0.831506000	2.117971000
H	2.883231000	-2.566243000	2.082697000
H	3.572269000	-1.790841000	0.617160000

60
TsI-E-E

C	-0.973801000	-0.976125000	0.554933000
H	-0.527581000	-1.879114000	0.983378000
N	0.453754000	-2.193030000	-2.620970000
H	1.899296000	-0.426757000	-6.408188000
C	0.489874000	-1.573268000	-3.794739000
N	1.614440000	-1.360818000	-4.521876000
C	2.919779000	-1.303169000	-3.901556000
H	2.822048000	-1.066677000	-2.835173000
H	3.505202000	-0.506136000	-4.386909000
H	3.462596000	-2.256170000	-4.009338000
C	1.619997000	-1.398446000	-5.968221000
H	0.636190000	-1.693507000	-6.350878000
H	2.354285000	-2.150559000	-6.301692000
H	-2.379646000	-0.971027000	-3.037144000
N	-0.673127000	-1.081276000	-4.283164000
C	-0.745232000	0.144048000	-5.049395000
H	0.230025000	0.644312000	-5.067290000
H	-1.075537000	-0.032854000	-6.086210000
C	-1.941276000	-1.602584000	-3.826779000
H	-2.643252000	-1.634624000	-4.674658000
H	-1.810354000	-2.614732000	-3.429708000
H	-1.469721000	0.823214000	-4.570654000
S	-0.723319000	0.505785000	1.589858000
C	0.000000000	0.000000000	0.000000000
H	-0.312968000	0.593057000	-0.864825000
C	1.471216000	-0.282142000	-0.005850000
S	1.388147000	-3.481964000	-2.296465000
O	1.765911000	-4.213287000	-3.517212000
O	2.469811000	-3.155857000	-1.356925000
C	0.241506000	-4.501585000	-1.395805000
C	0.372124000	-4.629397000	-0.017010000
C	-0.767614000	-5.173957000	-2.086452000
C	-0.531497000	-5.431084000	0.680479000
C	-1.661976000	-5.967138000	-1.377611000
C	-1.563941000	-6.105274000	0.017056000
H	1.175874000	-4.100495000	0.498527000
H	-0.845669000	-5.079532000	-3.171966000
H	-0.433210000	-5.532303000	1.764722000
H	-2.455251000	-6.494460000	-1.914787000
C	-2.561005000	-6.932014000	0.770586000
H	-2.216215000	-7.151095000	1.790897000
H	-3.523146000	-6.398244000	0.850236000
H	-2.763405000	-7.883985000	0.256686000
C	-2.293808000	-1.194397000	-0.098940000
C	-3.101323000	-0.132791000	-0.527713000
C	-2.729141000	-2.506623000	-0.311336000
C	-4.315949000	-0.381807000	-1.161070000
C	-3.945013000	-2.755739000	-0.948541000
C	-4.742477000	-1.695074000	-1.375408000
H	-2.779363000	0.895586000	-0.342263000
H	-2.103868000	-3.339050000	0.018438000
H	-4.937156000	0.455867000	-1.488456000
H	-4.268067000	-3.787573000	-1.109624000
H	-5.697190000	-1.888503000	-1.871093000
O	2.232335000	0.245944000	-0.780297000
O	1.836770000	-1.120576000	0.950789000
C	3.228125000	-1.390261000	1.066918000
H	3.787774000	-0.458368000	1.236410000
H	3.336478000	-2.053109000	1.933859000
H	3.590955000	-1.891318000	0.160814000

60
TS_TsI-Z-AB

C	-1.278335000	0.179158000	2.008555000
H	-1.877266000	0.902643000	1.437200000
N	-1.755081000	-1.017453000	2.267634000
H	0.218041000	-1.030122000	-5.082591000

C	-0.930546000	0.470207000	-2.591220000
N	0.086165000	-0.205607000	-3.130413000
C	1.466372000	0.026233000	-2.752864000
H	1.559445000	0.941794000	-2.158059000
H	2.064072000	0.128405000	-3.672211000
H	1.859971000	-0.821571000	-2.170988000
C	-0.110981000	-1.298573000	-4.066689000
H	-1.162775000	-1.599905000	-4.088482000
H	0.487822000	-2.155213000	-3.721831000
H	-3.590323000	1.983008000	-2.627942000
N	-2.079411000	0.641866000	-3.257502000
C	-2.116845000	0.748351000	-4.706484000
H	-1.105666000	0.873435000	-5.109086000
H	-2.592155000	-0.130006000	-5.169932000
C	-3.347665000	0.908140000	-2.598800000
H	-4.136201000	0.361426000	-3.137754000
H	-3.322410000	0.571147000	-1.555359000
H	-2.705814000	1.640356000	-4.968767000
S	-0.841958000	1.161269000	-0.984518000
C	0.000000000	0.000000000	0.000000000
H	-0.327121000	-1.036086000	-0.076633000
C	1.350786000	0.273400000	0.379526000
O	1.965189000	1.322454000	0.242739000
S	-3.073901000	-1.505772000	1.425811000
O	-3.869606000	-2.343998000	2.322929000
O	-3.763703000	-0.401655000	0.734316000
C	-2.380742000	-2.557437000	0.168123000
C	-1.375882000	-3.471383000	0.495167000
C	-2.876170000	-2.476485000	-1.129751000
C	-0.848068000	-4.283227000	-0.501582000
C	-2.347622000	-3.309563000	-2.113954000
C	-1.320761000	-4.215198000	-1.822309000
H	-0.992492000	-3.524139000	1.516203000
H	-3.665047000	-1.760290000	-1.364834000
H	-0.046966000	-4.984973000	-0.253241000
H	-2.737371000	-3.249074000	-3.133620000
C	-0.736236000	-5.094226000	-2.885908000
H	0.361739000	-5.011490000	-2.909720000
H	-0.971333000	-6.153937000	-2.692782000
H	-1.125697000	-4.837281000	-3.880912000
O	1.910528000	-0.797047000	1.000050000
C	3.211916000	-0.603982000	1.509651000
H	3.936941000	-0.414403000	0.701535000
H	3.251765000	0.240376000	2.214697000
H	3.483058000	-1.530665000	2.032620000
C	-0.224999000	0.736340000	2.866978000
C	0.454599000	-0.070497000	3.790784000
C	0.097420000	2.097530000	2.778473000
C	1.436649000	0.476426000	4.610457000
C	1.074891000	2.645181000	3.604823000
C	1.748343000	1.835830000	4.521459000
H	0.194739000	-1.128987000	3.855306000
H	-0.424132000	2.726070000	2.051578000
H	1.962751000	-0.158903000	5.327737000
H	1.316087000	3.708604000	3.531443000
H	2.517432000	2.264942000	5.169017000

60

TsI-Z-B

C	0.106507000	-1.548189000	-0.037243000
H	-0.834121000	-1.932742000	0.412462000
N	0.244653000	-1.897046000	-1.412528000
H	1.676729000	0.125355000	-4.840195000
C	-0.562664000	0.146915000	-2.684795000
N	0.664914000	0.559450000	-3.018295000
C	1.305298000	1.719219000	-2.438751000
H	0.578427000	2.322163000	-1.881703000
H	1.725456000	2.335149000	-3.249353000
H	2.132212000	1.425770000	-1.771058000
C	1.521645000	-0.287935000	-3.831264000
H	1.098995000	-1.299740000	-3.879547000
H	2.499521000	-0.354075000	-3.329904000
H	-3.420278000	-0.715602000	-3.201825000
N	-1.359068000	-0.433083000	-3.578076000
C	-1.155480000	-0.288201000	-5.006321000
H	-0.544586000	0.597374000	-5.214689000
H	-0.676156000	-1.179869000	-5.438348000
C	-2.472130000	-1.276868000	-3.191178000
H	-2.542728000	-2.103315000	-3.909802000
H	-2.291027000	-1.690689000	-2.194105000
H	-2.139411000	-0.153963000	-5.478762000
S	-1.297370000	0.584893000	-1.142024000
C	0.000000000	0.000000000	0.000000000
H	0.962852000	0.435828000	-0.291313000
C	-0.375507000	0.493415000	1.378704000
O	-1.465932000	0.350232000	1.874076000
C	1.246364000	-1.932375000	0.893446000
S	0.331090000	-3.392416000	-1.893684000
O	0.195548000	-3.380910000	-3.367603000
O	1.485227000	-4.153864000	-1.370318000
C	-1.096872000	-4.278407000	-1.263343000
C	-1.140710000	-4.648133000	0.081869000
C	-2.167828000	-4.581749000	-2.104823000
C	-2.271107000	-5.290206000	0.585359000
C	-3.286581000	-5.232696000	-1.593402000
C	-3.363665000	-5.589424000	-0.238588000
H	-0.283950000	-4.451854000	0.730597000
H	-2.103341000	-4.315884000	-3.161396000
H	-2.301302000	-5.575143000	1.640910000
H	-4.118907000	-5.476305000	-2.260519000
C	-4.584789000	-6.271117000	0.303088000
H	-4.865430000	-7.138611000	-0.314722000
H	-4.429367000	-6.618081000	1.334467000
H	-5.449691000	-5.586954000	0.304676000
C	0.984157000	-2.301811000	2.215634000
C	2.573963000	-1.855175000	0.460533000
C	2.030607000	-2.581843000	3.097076000
C	3.619727000	-2.129672000	1.337650000
C	3.351639000	-2.492261000	2.660589000
H	-0.052842000	-2.371230000	2.558135000
H	2.771394000	-1.601441000	-0.582999000
H	1.810880000	-2.873409000	4.127611000
H	4.653810000	-2.068255000	0.987655000
H	4.173541000	-2.711817000	3.347130000
O	0.651964000	1.071515000	1.982897000
C	0.445714000	1.482441000	3.330143000
H	-0.370306000	2.216843000	3.392902000
H	0.203535000	0.617176000	3.964621000
H	1.388072000	1.935745000	3.658374000

60

TS_TsI-Z-BC

C	1.163291000	-0.547345000	0.850762000
H	0.969180000	-0.256153000	1.897709000
N	1.148908000	-1.987108000	0.700005000
H	0.964448000	-4.557136000	-0.386162000
C	-0.555134000	-2.631951000	0.160353000
N	-0.349932000	-3.086873000	-1.127753000
C	-0.545011000	-2.250533000	-2.286984000
H	-1.420128000	-1.601627000	-2.162198000
H	-0.722770000	-2.902352000	-3.155927000
H	0.341238000	-1.627988000	-2.516009000
C	0.692286000	-4.074789000	-1.330046000
H	1.600153000	-3.590059000	-1.732053000
H	0.359718000	-4.838479000	-2.051344000
H	-0.547017000	-3.982470000	3.037659000
N	-1.041361000	-3.549727000	1.050713000
C	-1.325167000	-4.919117000	0.701466000
H	-2.208696000	-5.245815000	1.271953000
H	-1.557091000	-5.000572000	-0.367234000
C	-1.136067000	-3.253029000	2.460607000
H	-0.738651000	-2.254886000	2.672551000
H	-2.185608000	-3.294150000	2.798514000
H	-0.487209000	-5.594189000	0.948723000
S	-1.429275000	-1.049370000	0.346533000
C	0.000000000	0.000000000	0.000000000
H	0.253437000	-0.048092000	-1.066825000
C	-0.292955000	1.451803000	0.306130000
O	-0.183608000	2.339280000	-0.500841000
C	2.443661000	0.128908000	0.401201000
S	2.017625000	-2.802242000	1.810913000
O	1.939512000	-2.176046000	3.141166000
O	1.665773000	-4.225116000	1.701126000
C	3.716078000	-2.661006000	1.296086000
C	4.566434000	-1.777497000	1.957359000
C	4.168641000	-3.409576000	0.210599000
C	5.872809000	-1.622031000	1.504801000
C	5.478232000	-3.244104000	-0.230237000
C	6.347204000	-2.340643000	0.398199000
H	4.196336000	-1.215277000	2.816014000
H	3.501344000	-4.115620000	-0.286664000
H	6.538821000	-0.922902000	2.018047000
H	5.835880000	-3.827858000	-1.082825000
C	7.738314000	-2.121989000	-0.113432000
H	8.458710000	-2.013924000	0.711268000
H	8.066614000	-2.947147000	-0.761208000
H	7.786160000	-1.192931000	-0.707010000
C	3.084913000	-0.258668000	-0.780383000
C	2.978010000	1.177399000	1.153849000
C	4.249455000	0.382782000	-1.192938000
C	4.142819000	1.825562000	0.739514000
C	4.783530000	1.426795000	-0.432461000
H	2.674019000	-1.086823000	-1.362572000
H	2.483674000	1.478223000	2.082307000
H	4.749812000	0.063602000	-2.110986000
H	4.555420000	2.639458000	1.341289000
H	5.700888000	1.926263000	-0.754609000
O	-0.659402000	1.633500000	1.568955000
C	-0.915173000	2.974630000	1.969470000
H	-1.723698000	3.413233000	1.366851000
H	-1.213360000	2.927192000	3.023144000
H	-0.009576000	3.589311000	1.860593000

60

TsI-Z-C

C	1.000244000	0.316922000	-1.114829000
N	1.366334000	1.729621000	-0.972482000
S	2.620333000	2.283754000	-1.954281000
O	2.581650000	1.535133000	-3.212896000
O	2.553001000	3.738499000	-1.994158000
C	4.124033000	1.852559000	-1.128909000
C	4.395108000	2.406857000	0.121985000
C	5.599138000	2.104029000	0.743270000
C	6.545466000	1.268508000	0.126034000
C	6.253803000	0.754314000	-1.143062000
C	5.047322000	1.040197000	-1.777656000
H	4.813715000	0.627818000	-2.760293000
H	6.981764000	0.110126000	-1.643057000
C	7.824176000	0.923443000	0.825568000
H	8.559765000	0.491387000	0.132810000
H	8.269806000	1.807868000	1.305568000
H	7.639848000	0.183410000	1.622603000
H	5.815908000	2.526548000	1.728129000
H	3.667867000	3.062544000	0.602774000
C	0.351249000	2.712061000	-0.516975000
N	0.889403000	3.590122000	0.437291000
C	1.246154000	3.072604000	1.728368000
H	2.102344000	3.638886000	2.128750000
H	1.546826000	2.017002000	1.658944000
H	0.421031000	3.141198000	2.463307000
C	1.011912000	5.010925000	0.250622000
H	0.277825000	5.591683000	0.843425000
H	2.019630000	5.337727000	0.559864000
H	0.893875000	5.256201000	-0.809489000
N	-0.323595000	3.391659000	-1.563849000
C	-0.585521000	2.640388000	-2.769196000
H	-1.052062000	3.314822000	-3.501376000
H	-1.281613000	1.795049000	-2.590080000
H	0.333938000	2.250616000	-3.220989000
C	-1.510032000	4.109527000	-1.141973000
H	-1.747788000	4.895714000	-1.874883000
H	-2.385740000	3.434363000	-1.065480000
H	-1.366923000	4.587363000	-0.165878000
S	-0.964740000	1.492058000	0.331657000
C	0.000000000	0.000000000	0.000000000
C	-0.942635000	-1.106990000	-0.416581000
O	-1.214485000	-1.401561000	-1.554006000
O	-1.461615000	-1.706923000	0.648377000
C	-2.418902000	-2.728588000	0.397241000
H	-3.284309000	-2.326004000	-0.149699000
H	-2.735309000	-3.099829000	1.379004000
H	-1.972713000	-3.545865000	-0.188179000
H	0.513930000	-0.304591000	0.921584000
C	2.174850000	-0.633621000	-1.011730000
C	2.491489000	-1.469931000	-2.083678000
C	3.543780000	-2.381032000	-1.984362000
C	4.288415000	-2.463452000	-0.808620000
C	3.976973000	-1.630871000	0.268700000
C	2.927361000	-0.722800000	0.166374000
H	2.696588000	-0.065336000	1.008461000
H	4.559473000	-1.686074000	1.191803000
H	5.113763000	-3.175846000	-0.730320000
H	3.782470000	-3.029038000	-2.831558000
H	1.911391000	-1.398214000	-3.006662000
H	0.495761000	0.141047000	-2.077274000

60

TS_TsI-Z-CD

C	-0.968834000	-1.167821000	-0.213911000
H	-1.951703000	-0.703334000	-0.360554000
N	-0.632397000	-1.793877000	-1.505948000
H	0.393433000	-0.573919000	-5.632654000
C	-0.659907000	-0.924747000	-2.648624000
N	0.351735000	-1.046799000	-3.568030000
C	1.733320000	-0.958440000	-3.177982000
H	1.815928000	-0.729121000	-2.110374000
H	2.240207000	-0.151374000	-3.736059000
H	2.255501000	-1.907945000	-3.382303000
C	0.125492000	-1.397342000	-4.946076000
H	-0.926320000	-1.661528000	-5.103779000
H	0.742051000	-2.275009000	-5.202188000
H	-3.455646000	-0.294870000	-1.792309000
N	-1.911178000	-0.697691000	-3.191387000
C	-2.062104000	0.502550000	-3.982661000
H	-1.104383000	0.807279000	-4.419469000
H	-2.780823000	0.332856000	-4.799078000
C	-3.120117000	-1.073359000	-2.505359000
H	-3.919358000	-1.209105000	-3.249811000
H	-2.989249000	-2.017698000	-1.967013000
H	-2.429239000	1.332209000	-3.350177000
S	-0.131598000	1.004672000	-1.496617000
C	0.000000000	0.000000000	0.000000000
H	1.029611000	-0.358415000	0.146689000
C	-0.358465000	0.823573000	1.210143000
S	0.192790000	-3.275077000	-1.563995000
O	0.345078000	-3.641480000	-2.967607000
O	1.375387000	-3.202073000	-0.710721000
C	-0.931209000	-4.455377000	-0.873525000
C	-0.517653000	-5.230318000	0.205174000
C	-2.151476000	-4.686046000	-1.509009000
C	-1.366546000	-6.224475000	0.682391000
C	-2.990223000	-5.675411000	-1.011352000
C	-2.618039000	-6.453882000	0.096279000
H	0.452369000	-5.045884000	0.668287000
H	-2.441923000	-4.104121000	-2.385794000
H	-1.051538000	-6.831599000	1.535088000
H	-3.953068000	-5.855647000	-1.496675000
C	-3.548390000	-7.486478000	0.653595000
H	-2.998433000	-8.301176000	1.146150000
H	-4.211071000	-7.033827000	1.411154000
H	-4.192047000	-7.914908000	-0.128260000
C	-1.125747000	-2.078352000	0.982333000
C	-2.402426000	-2.569518000	1.284797000
C	-0.070443000	-2.357839000	1.856559000
C	-2.618837000	-3.341286000	2.422351000
C	-0.286929000	-3.124356000	3.001745000
C	-1.557891000	-3.621109000	3.286041000
H	-3.235469000	-2.335599000	0.616572000
H	0.931542000	-1.985283000	1.640351000
H	-3.620509000	-3.720123000	2.640778000
H	0.547041000	-3.334175000	3.676281000
H	-1.724644000	-4.222309000	4.183540000
O	0.446148000	1.291698000	1.978459000
O	-1.675195000	0.989369000	1.338936000
C	-2.111121000	1.756760000	2.449358000
H	-1.733560000	2.788390000	2.386259000
H	-3.207125000	1.758142000	2.411366000
H	-1.769229000	1.308764000	3.394348000

60

TsI-Z-D

C	-1.059592000	-1.073001000	-0.285193000
H	-1.977883000	-0.521412000	-0.521164000
N	-0.714664000	-1.801036000	-1.536109000
H	-0.060287000	-0.234150000	-5.585472000
C	-0.898617000	-1.149805000	-2.748567000
N	0.069473000	-1.132174000	-3.669087000
C	1.469465000	-1.027356000	-3.325368000
H	1.579356000	-0.852993000	-2.250519000
H	1.895672000	-0.155516000	-3.846280000
H	2.007782000	-1.936781000	-3.628766000
C	-0.215927000	-1.203339000	-5.084337000
H	-1.247333000	-1.534955000	-5.249598000
H	0.465119000	-1.940363000	-5.536800000
H	-3.779776000	-0.539596000	-1.761033000
N	-2.129991000	-0.716930000	-3.065636000
C	-2.300402000	0.493397000	-3.841077000
H	-1.332798000	0.992648000	-3.961090000
H	-2.749043000	0.289488000	-4.826061000
C	-3.334497000	-1.251627000	-2.476168000
H	-4.069543000	-1.440697000	-3.274135000
H	-3.124284000	-2.195187000	-1.963630000
H	-2.962869000	1.172921000	-3.282218000
S	0.034609000	1.254450000	-1.319095000
C	0.000000000	0.000000000	0.000000000
H	0.985113000	-0.474612000	0.127792000
C	-0.291029000	0.743936000	1.273426000
S	0.139952000	-3.285147000	-1.476813000
O	0.380552000	-3.682456000	-2.858379000
O	1.256132000	-3.120605000	-0.555494000
C	-0.992767000	-4.468033000	-0.812158000
C	-0.639953000	-5.160110000	0.342180000
C	-2.146657000	-4.787651000	-1.529289000
C	-1.485961000	-6.161665000	0.808237000
C	-2.984167000	-5.782114000	-1.040692000
C	-2.674623000	-6.478818000	0.138456000
H	0.281528000	-4.910158000	0.868796000
H	-2.389489000	-4.272263000	-2.460474000
H	-1.218253000	-6.705112000	1.718026000
H	-3.895917000	-6.031499000	-1.589694000
C	-3.606508000	-7.517489000	0.680828000
H	-3.066785000	-8.280837000	1.259386000
H	-4.342101000	-7.051797000	1.359021000
H	-4.170811000	-8.013261000	-0.122134000
C	-1.386454000	-1.963403000	0.897398000
C	-2.697874000	-2.427053000	1.063944000
C	-0.442713000	-2.255501000	1.888193000
C	-3.056123000	-3.175562000	2.181686000
C	-0.801703000	-2.994638000	3.015347000
C	-2.106692000	-3.460226000	3.165011000
H	-3.451094000	-2.187789000	0.310005000
H	0.585396000	-1.907327000	1.783157000
H	-4.084245000	-3.530124000	2.291353000
H	-0.051570000	-3.208873000	3.780853000
H	-2.386146000	-4.041257000	4.047647000
O	0.540175000	1.103366000	2.075609000
O	-1.598381000	0.993663000	1.430580000

C -1.964894000 1.720913000 2.586944000
H -1.530354000 2.732548000 2.573863000
H -3.059971000 1.789456000 2.576404000
H -1.632833000 1.206400000 3.501810000

60
TS_TsI-Z-DE
C -1.014550000 -1.086378000 0.033298000
H -1.900717000 -0.755149000 -0.506530000
N -0.536053000 -2.249694000 -1.469447000
H -0.049677000 -0.675605000 -5.554181000
C -0.783403000 -1.729135000 -2.691278000
N 0.161258000 -1.540159000 -3.627339000
C 1.559861000 -1.331134000 -3.320048000
H 1.703801000 -1.156870000 -2.249582000
H 1.904075000 -0.433974000 -3.859317000
H 2.161162000 -2.195736000 -3.634341000
C -0.136603000 -1.646728000 -5.041691000
H -1.143944000 -2.049138000 -5.196018000
H 0.587286000 -2.342611000 -5.493845000
H -3.771132000 -1.297712000 -1.752674000
N -2.039419000 -1.313592000 -2.956650000
C -2.314950000 -0.147958000 -3.771501000
H -1.399744000 0.435088000 -3.926073000
H -2.744388000 -0.415864000 -4.750139000
C -3.203118000 -1.975112000 -2.410391000
H -3.864744000 -2.287381000 -3.234862000
H -2.899334000 -2.859715000 -1.842772000
H -3.042209000 0.483429000 -3.237416000
S -0.896415000 1.050197000 1.185449000
C 0.000000000 0.000000000 0.000000000
H -0.015902000 0.443456000 -1.005458000
C 1.474747000 -0.182368000 0.275592000
S 0.498258000 -3.523881000 -1.240550000
O 0.950275000 -4.047746000 -2.532464000
O 1.496739000 -3.130671000 -0.246170000
C -0.520308000 -4.787462000 -0.525861000
C -0.154855000 -5.338885000 0.697379000
C -1.597348000 -5.297374000 -1.253373000
C -0.901737000 -6.395249000 1.213187000
C -2.337855000 -6.344429000 -0.718979000
C -2.008269000 -6.906548000 0.524821000
H 0.703364000 -4.937550000 1.237330000
H -1.855369000 -4.886162000 -2.231395000
H -0.621111000 -6.826731000 2.177672000
H -3.188112000 -6.741173000 -1.280121000
C -2.839181000 -8.004181000 1.114871000
H -2.238286000 -8.664110000 1.757185000
H -3.642006000 -7.580366000 1.742459000
H -3.320389000 -8.611556000 0.334744000
C -1.360221000 -2.007355000 1.144781000
C -2.637706000 -2.590638000 1.116190000
C -0.507286000 -2.337895000 2.207611000
C -3.056541000 -3.464884000 2.115660000
C -0.930828000 -3.200165000 3.214152000
C -2.203723000 -3.771178000 3.175506000
H -3.322837000 -2.338362000 0.305500000
H 0.480804000 -1.888989000 2.252744000
H -4.057447000 -3.901511000 2.068712000
H -0.253061000 -3.432958000 4.039731000
H -2.528701000 -4.452062000 3.966430000
O 2.311858000 0.098845000 -0.553894000
O 1.803381000 -0.578897000 1.497297000
C 3.184914000 -0.790931000 1.739122000
H 3.752510000 0.143696000 1.620329000
H 3.263070000 -1.145907000 2.774085000
H 3.586510000 -1.547874000 1.049855000

60
TsI-Z-E
C -1.198941000 -0.453191000 0.763116000
H -2.120895000 -0.260500000 0.203172000
N 0.159973000 -2.534025000 -2.053107000
H 0.634393000 -0.437712000 -5.915685000
C -0.077067000 -1.804988000 -3.136457000
N 0.822767000 -1.553734000 -4.118292000
C 2.248202000 -1.583238000 -3.875134000
H 2.449683000 -1.469033000 -2.803827000
H 2.714124000 -0.742000000 -4.412892000
H 2.698820000 -2.524065000 -4.229776000
C 0.437842000 -1.443386000 -5.508668000
H -0.625105000 -1.678678000 -5.635823000
H 1.020139000 -2.172051000 -6.096660000
H -2.769342000 -1.140413000 -1.737237000
N -1.295886000 -1.219292000 -3.247913000
C -1.484883000 0.080434000 -3.853255000
H -0.516609000 0.554850000 -4.051626000
H -2.055212000 0.024937000 -4.795504000
C -2.453742000 -1.769844000 -2.585511000
H -3.293266000 -1.826261000 -3.297858000
H -2.227355000 -2.774282000 -2.211886000
H -2.045650000 0.722437000 -3.154086000
S -0.465993000 1.091416000 1.385705000
C 0.000000000 0.000000000 0.000000000
H -0.198577000 0.434370000 -0.984340000
C 1.379772000 -0.579198000 0.045644000
S 1.110675000 -3.853805000 -2.094176000
O 1.135243000 -4.474210000 -3.429223000
O 2.410910000 -3.616230000 -1.451082000
C 0.217977000 -4.951182000 -1.016299000
C 0.720246000 -5.225389000 0.251176000
C -0.949931000 -5.563754000 -1.473902000
C 0.038855000 -6.121717000 1.072865000
C -1.620383000 -6.451063000 -0.640524000
C -1.139125000 -6.744252000 0.646018000
H 1.641867000 -4.744159000 0.582862000
H -1.320957000 -5.354378000 -2.479906000
H 0.429576000 -6.338111000 2.070580000
H -2.536096000 -6.933227000 -0.994211000
C -1.892415000 -7.675924000 1.545494000
H -1.304254000 -7.942005000 2.434899000
H -2.830644000 -7.209076000 1.889859000
H -2.171050000 -8.602876000 1.020802000
C -1.329012000 -1.664468000 1.627925000
C -2.213963000 -2.665478000 1.205742000
C -0.670199000 -1.819431000 2.851983000
C -2.428276000 -3.801327000 1.984714000
C -0.880863000 -2.956465000 3.628616000
C -1.761119000 -3.951398000 3.199494000
H -2.746629000 -2.548027000 0.259157000
H 0.004008000 -1.032075000 3.193707000
H -3.122831000 -4.570729000 1.639404000
H -0.359744000 -3.061667000 4.583812000
H -1.929185000 -4.840218000 3.813167000
O 2.224885000 -0.252776000 -0.753482000
O 1.588787000 -1.402042000 1.056905000
C 2.909301000 -1.908641000 1.210554000
H 3.620985000 -1.085200000 1.373115000
H 2.881068000 -2.555303000 2.095986000
H 3.196438000 -2.485680000 0.322086000

37
TsI-Sideproduct
N 0.000000000 0.000000000 0.000000000
C 0.703409000 0.761155000 0.821518000
N 0.754164000 2.121697000 0.782231000
C 0.774216000 2.851624000 -0.465533000
H 0.689893000 2.164227000 -1.313151000
H 1.718285000 3.418380000 -0.546135000
H -0.060340000 3.569012000 -0.517200000
C 0.817069000 2.932790000 1.976876000
H 0.622192000 2.322337000 2.866459000
H 0.036818000 3.709777000 1.916222000
H 1.790613000 3.438447000 2.095409000
N 1.412761000 0.134735000 1.790583000
C 1.099493000 -1.225698000 2.164001000
H 0.064752000 -1.458394000 1.888936000
H 1.229067000 -1.341317000 3.251378000
H 1.763199000 -1.947817000 1.657150000
C 2.664768000 0.647863000 2.303655000
H 2.590573000 0.929865000 3.366732000
H 3.438480000 -0.131337000 2.209073000
H 2.990295000 1.519971000 1.725439000
S -1.437572000 0.397015000 -0.641463000
O -2.231797000 -0.834661000 -0.639325000
O -1.306134000 1.084253000 -1.934795000
C -2.230325000 1.525106000 0.494170000
C -2.471956000 1.116420000 1.808053000
C -3.122839000 1.981077000 2.678956000
C -3.556685000 3.249180000 2.256109000
C -3.317299000 3.624097000 0.929422000
C -2.656664000 2.769948000 0.045843000
H -2.471132000 3.060841000 -0.990066000
H -3.654956000 4.602994000 0.578237000
C -4.260201000 4.166095000 3.210246000
H -5.174498000 3.696331000 3.607200000
H -4.542548000 5.111871000 2.727229000
H -3.620399000 4.400245000 4.076396000
H -3.307415000 1.666735000 3.710085000
H -2.151533000 0.128129000 2.146995000

50
TS_MsI-E-AB
C -6.377595000 -12.984808000 1.649592000
H -5.375381000 -12.776954000 2.049026000
N -6.724311000 -14.177893000 1.270309000
H -3.747687000 -13.230060000 -5.262585000
C -5.846257000 -13.051423000 -2.954517000
N -4.708432000 -12.584962000 -3.477212000
C -4.252914000 -11.224729000 -3.270619000
H -5.040193000 -10.614441000 -2.813261000
H -3.980523000 -10.797866000 -4.249380000

H	-3.366223000	-11.203031000	-2.618243000
C	-3.775567000	-13.445204000	-4.182867000
H	-4.036443000	-14.498241000	-4.026815000
H	-2.770183000	-13.273343000	-3.768450000
H	-8.458001000	-14.572189000	-2.885571000
N	-6.506228000	-14.058567000	-3.541446000
C	-6.490339000	-14.248979000	-4.981274000
H	-6.059223000	-13.373357000	-5.479412000
H	-5.927027000	-15.149799000	-5.270846000
C	-7.416876000	-14.927514000	-2.814311000
H	-7.362013000	-15.930144000	-3.264039000
H	-7.111693000	-14.994457000	-1.763137000
H	-7.529765000	-14.364598000	-5.324419000
S	-6.526341000	-12.408135000	-1.477364000
C	-5.185527000	-12.059226000	-0.454124000
H	-4.510430000	-12.871123000	-0.186007000
C	-4.883270000	-10.713866000	-0.132648000
O	-5.445559000	-9.695749000	-0.524254000
S	-5.486556000	-15.277362000	1.124560000
O	-5.390825000	-15.662075000	-0.289330000
O	-4.250630000	-14.823857000	1.779532000
O	-3.848394000	-10.636690000	0.760568000
C	-3.471058000	-9.337238000	1.150739000
H	-3.143549000	-8.730882000	0.290241000
H	-4.294086000	-8.800736000	1.649819000
H	-2.634230000	-9.451662000	1.853309000
C	-7.371350000	-11.918893000	1.769071000
C	-8.691141000	-12.103634000	1.328174000
C	-6.998592000	-10.690092000	2.334082000
C	-9.617860000	-11.073209000	1.449370000
C	-7.928802000	-9.662770000	2.456563000
C	-9.239504000	-9.851942000	2.013383000
H	-8.971599000	-13.064231000	0.891106000
H	-5.967409000	-10.547672000	2.667026000
H	-10.644062000	-11.219888000	1.102805000
H	-7.630873000	-8.707512000	2.895869000
H	-9.969748000	-9.043943000	2.107018000
C	-6.130165000	-16.653956000	2.026003000
H	-7.094133000	-16.940182000	1.586339000
H	-5.399000000	-17.467937000	1.922358000
H	-6.244471000	-16.365773000	3.079146000

50
TS_MsI-Z-AB

C	-6.655160000	-12.959768000	1.058254000
H	-7.183734000	-12.103345000	0.615020000
N	-7.259307000	-14.126542000	1.154397000
H	-5.207210000	-14.628539000	-5.753336000
C	-6.373059000	-12.847354000	-3.462876000
N	-5.322603000	-13.521839000	-3.940484000
C	-3.954430000	-13.146352000	-3.644582000
H	-3.917744000	-12.152304000	-3.184610000
H	-3.387923000	-13.126165000	-4.588938000
H	-3.482076000	-13.875248000	-2.968013000
C	-5.473943000	-14.755189000	-4.692602000
H	-6.502739000	-15.124961000	-4.616142000
H	-4.801585000	-15.507532000	-4.252199000
H	-9.137516000	-11.516370000	-3.700618000
N	-7.530432000	-12.816854000	-4.133887000
C	-7.570516000	-12.899449000	-5.584174000
H	-6.568479000	-12.758278000	-6.004029000
H	-7.981851000	-13.861077000	-5.927994000
C	-8.814953000	-12.552525000	-3.507302000
H	-9.559612000	-13.233179000	-3.947813000
H	-8.771147000	-12.718948000	-2.423430000
H	-8.219968000	-12.092160000	-5.954640000
S	-6.315638000	-12.008814000	-1.929405000
C	-5.364155000	-13.028801000	-0.878520000
H	-5.554247000	-14.100619000	-0.931810000
C	-4.033663000	-12.600434000	-0.556938000
O	-3.555974000	-11.486040000	-0.709559000
S	-8.677311000	-14.316884000	0.352755000
O	-9.576191000	-15.080874000	1.220467000
O	-9.196632000	-13.050808000	-0.200774000
O	-3.328256000	-13.600956000	0.024466000
C	-2.053545000	-13.254137000	0.521757000
H	-1.375699000	-12.945105000	-0.290103000
H	-2.113899000	-12.435307000	1.254586000
H	-1.653777000	-14.152978000	1.009177000
C	-5.575063000	-12.626794000	2.000651000
C	-4.983929000	-13.611460000	2.804340000
C	-5.148401000	-11.297176000	2.122283000
C	-3.984218000	-13.271791000	3.710406000
C	-4.153751000	-10.956889000	3.035738000
C	-3.567084000	-11.943475000	3.830390000
H	-5.327448000	-14.643610000	2.707922000
H	-5.602985000	-10.529546000	1.490683000
H	-3.527625000	-14.046047000	4.332603000
H	-3.832122000	-9.916300000	3.127033000
H	-2.784586000	-11.677170000	4.545719000
C	-8.254334000	-15.373660000	-1.011085000
H	-7.804216000	-16.289927000	-0.607950000
H	-9.193048000	-15.604089000	-1.534636000
H	-7.557584000	-14.856855000	-1.682046000

64
TS_MsI'-E-AB

C	-0.674200000	-1.787364000	1.767642000
H	-1.433930000	-2.556960000	1.560522000
N	0.416734000	-2.077604000	2.447699000
C	1.304529000	0.425924000	-0.486877000
N	2.351221000	0.355048000	0.376110000
C	3.363208000	-0.731268000	0.672196000
C	2.588060000	1.612435000	0.871183000
H	3.369429000	1.820941000	1.593705000
N	0.930135000	1.731686000	-0.568273000
C	1.715513000	2.465979000	0.280098000
H	1.609206000	3.535257000	0.415025000
C	-0.054802000	2.361511000	-1.521398000
S	0.619340000	-0.847528000	-1.499649000
C	-0.278157000	-1.985679000	-0.481708000
H	0.299041000	-2.891889000	-0.299768000
C	-1.638656000	-2.292322000	-0.844388000
O	-2.223137000	-3.300953000	-0.464003000
C	-1.217672000	-0.417211000	1.852250000
C	4.644105000	-0.325058000	-0.056902000
H	5.038818000	0.634788000	0.307403000
H	5.411300000	-1.094747000	0.111306000
H	4.467541000	-0.245522000	-1.140465000
C	2.923849000	-2.107213000	0.207487000
H	3.682439000	-2.823013000	0.554344000
H	1.974333000	-2.379106000	0.677804000
H	2.844997000	-2.186188000	-0.882533000
C	3.574013000	-0.784660000	2.184516000
H	3.938139000	0.163265000	2.603303000
H	2.645442000	-1.089838000	2.687760000
H	4.336074000	-1.547910000	2.395721000
C	-0.116736000	3.861083000	-1.244973000
H	-0.449508000	4.078669000	-0.219434000
H	0.841305000	4.367278000	-1.433766000
H	-0.858075000	4.291478000	-1.931979000
C	-1.443618000	1.776033000	-1.310772000
H	-2.139223000	2.275841000	-2.001165000
H	-1.482248000	0.698647000	-1.502500000
H	-1.785280000	1.966539000	-0.285138000
C	0.457448000	2.148070000	-2.945069000
H	-0.200362000	2.683905000	-3.644841000
H	1.477140000	2.547410000	-3.055777000
H	0.457743000	1.086753000	-3.226471000
O	-2.279341000	-1.351268000	-1.568905000
C	-3.646169000	-1.585573000	-1.841275000
H	-3.785382000	-2.507109000	-2.427044000
H	-4.234608000	-1.668821000	-0.914509000
H	-3.996273000	-0.722597000	-2.422392000
C	-0.446303000	0.645795000	2.340121000
C	-2.575187000	-0.201544000	1.572729000
C	-1.018942000	1.899485000	2.537302000
C	-3.151106000	1.048240000	1.786013000
C	-2.374286000	2.103602000	2.267903000
H	0.601996000	0.467664000	2.586686000
H	-3.188406000	-1.033454000	1.217114000
H	-0.406801000	2.721099000	2.918161000
H	-4.213521000	1.198933000	1.578616000
H	-2.825702000	3.084633000	2.436468000
S	0.628688000	-3.633329000	2.936874000
O	-0.443068000	-3.997523000	3.874695000
O	2.012691000	-3.738869000	3.406915000
C	0.456185000	-4.733582000	1.543222000
H	1.282449000	-4.573635000	0.839227000
H	0.516324000	-5.744714000	1.969733000
H	-0.516721000	-4.586298000	1.053204000

64
TS_MsI'-Z-AB

C	-0.989403000	-0.251644000	-1.446838000
H	-0.873500000	-1.328888000	-1.289634000
N	0.033169000	0.310575000	-2.050688000
C	1.983210000	0.093791000	0.323887000
N	2.712221000	-0.648295000	-0.544620000
C	2.771966000	-2.147058000	-0.659906000
C	3.491247000	0.196747000	-1.287460000
H	4.156280000	-0.142128000	-2.072502000
N	2.342111000	1.390936000	0.162965000
C	3.257087000	1.463262000	-0.851485000
H	3.686738000	2.394932000	-1.198110000
C	2.022530000	2.566729000	1.052115000
S	0.877062000	-0.516066000	1.553545000
C	-0.766002000	-0.293731000	0.956961000
H	-1.223995000	0.653963000	1.249164000
C	-1.599500000	-1.452900000	1.120111000
O	-1.287590000	-2.628352000	0.968218000
C	1.400458000	-2.699652000	-1.026147000
H	1.495230000	-3.784373000	-1.183472000

H	0.653332000	-2.538728000	-0.237315000
H	1.047998000	-2.245024000	-1.963153000
C	3.284295000	-2.711945000	0.663846000
H	2.580812000	-2.524360000	1.485447000
H	3.409250000	-3.799960000	0.563065000
H	4.261072000	-2.274443000	0.921101000
C	3.758373000	-2.513597000	-1.764670000
H	4.780087000	-2.169050000	-1.547869000
H	3.786175000	-3.609549000	-1.834396000
H	3.443477000	-2.125997000	-2.744731000
C	2.773687000	2.353570000	2.364661000
H	2.594887000	3.213120000	3.027517000
H	2.424043000	1.445715000	2.877015000
H	3.857063000	2.270468000	2.188529000
C	0.521739000	2.705541000	1.284619000
H	-0.016168000	2.636951000	0.330669000
H	0.143417000	1.955833000	1.987086000
H	0.333688000	3.697534000	1.720201000
C	2.519386000	3.836944000	0.367216000
H	2.240000000	4.689585000	1.000925000
H	3.612858000	3.859258000	0.256605000
H	2.040287000	3.963563000	-0.614100000
O	-2.877474000	-1.106935000	1.416293000
C	-3.809800000	-2.163114000	1.487920000
H	-3.593228000	-2.831028000	2.337573000
H	-4.795165000	-1.700345000	1.630801000
H	-3.817125000	-2.767397000	0.568375000
C	-2.391141000	0.215441000	-1.396511000
C	-3.398948000	-0.732800000	-1.609026000
C	-2.752557000	1.541224000	-1.115382000
C	-4.742958000	-0.365267000	-1.564524000
C	-4.093133000	1.902896000	-1.050086000
C	-5.093387000	0.952936000	-1.278616000
H	-3.121162000	-1.770611000	-1.812470000
H	-1.979046000	2.290792000	-0.937626000
H	-5.518008000	-1.115505000	-1.740767000
H	-4.363713000	2.936234000	-0.818386000
H	-6.146014000	1.243440000	-1.229066000
S	-0.029080000	1.754160000	-2.833205000
O	0.309614000	2.872125000	-1.931418000
O	-1.231344000	1.920759000	-3.661662000
C	1.322272000	1.543444000	-3.956694000
H	2.248904000	1.372441000	-3.398056000
H	1.096424000	0.693835000	-4.613661000
H	1.381862000	2.474124000	-4.537573000

33
Z-Barton

N	-1.323737000	1.889124000	-0.687947000
N	0.000000000	0.000000000	0.000000000
C	-0.545364000	1.145742000	0.197546000
N	-0.340007000	1.772544000	1.417925000
C	0.685821000	1.270163000	2.292398000
H	0.357076000	0.386034000	2.872983000
H	1.567808000	0.973086000	1.710786000
H	0.972824000	2.059179000	3.005758000
C	-1.409949000	2.474824000	2.086206000
H	-1.064819000	3.442258000	2.489044000
H	-2.237745000	2.664713000	1.393061000
H	-1.808662000	1.880767000	2.930904000
C	-1.132373000	3.303719000	-0.864775000
H	-0.245565000	3.637944000	-0.311258000
H	-0.979409000	3.541363000	-1.934359000
H	-1.999210000	3.898023000	-0.516364000
C	-2.467108000	1.328015000	-1.350875000
H	-3.381272000	1.894314000	-1.087529000
H	-2.378630000	1.347730000	-2.453788000
H	-2.617330000	0.287854000	-1.038934000
C	0.325931000	-0.628922000	-1.271914000
C	1.682913000	-1.310785000	-1.036233000
H	1.618755000	-1.997925000	-0.178463000
H	2.008091000	-1.882070000	-1.920593000
H	2.455538000	-0.559095000	-0.808248000
C	0.486059000	0.320550000	-2.466439000
H	0.893969000	-0.235972000	-3.325482000
H	-0.460010000	0.773659000	-2.787070000
H	1.192799000	1.132209000	-2.231269000
C	-0.696698000	-1.723968000	-1.599342000
H	-1.676913000	-1.304533000	-1.866916000
H	-0.353752000	-2.338789000	-2.446722000
H	-0.834927000	-2.384700000	-0.729122000

56
Z-A

C	0.003626000	1.240443000	-0.846441000
C	-0.935043000	1.454422000	-1.985413000
C	-1.028535000	0.582125000	-3.076757000
C	-1.941934000	0.830065000	-4.099142000
C	-2.768099000	1.953942000	-4.051699000
C	-2.672236000	2.834168000	-2.974152000
C	-1.758852000	2.587637000	-1.950292000
H	-1.680878000	3.282560000	-1.109694000
H	-3.306925000	3.723025000	-2.932056000
H	-3.478964000	2.148123000	-4.858862000
H	-2.001781000	0.142002000	-4.946352000
H	-0.374289000	-0.290163000	-3.118306000
C	0.000000000	0.000000000	0.000000000
C	-0.966468000	-1.107905000	-0.306052000
O	-0.894070000	-1.898522000	-1.209921000
O	-1.954510000	-1.084295000	0.585544000
C	-2.979148000	-2.055756000	0.414816000
H	-3.694632000	-1.894151000	1.229621000
H	-3.479912000	-1.927557000	-0.556293000
H	-2.567029000	-3.073989000	0.472397000
S	1.472215000	0.190112000	-1.014680000
H	0.128158000	0.166580000	1.075241000
H	0.189153000	2.156571000	-0.274449000
N	1.493657000	4.460731000	-4.317778000
N	2.824444000	2.577631000	-3.629817000
C	2.277989000	3.722313000	-3.433853000
N	2.473437000	4.346880000	-2.209358000
C	3.496301000	3.847973000	-1.329331000
H	3.164888000	2.965668000	-0.747310000
H	4.379568000	3.548001000	-1.907402000
H	3.781939000	4.639967000	-0.618486000
C	1.390657000	5.031371000	-1.543918000
H	1.716470000	6.006517000	-1.143115000
H	0.559106000	5.202638000	-2.237359000
H	1.004122000	4.432716000	-0.696523000
C	1.674024000	5.876840000	-4.491328000
H	2.562738000	6.215666000	-3.943612000
H	1.816842000	6.119706000	-5.561124000
H	0.805645000	6.463830000	-4.134029000
C	0.346573000	3.892803000	-4.969527000
H	-0.561522000	4.472951000	-4.718830000
H	0.434760000	3.886241000	-6.072826000
H	0.185581000	2.862439000	-4.632295000
C	3.140427000	1.941223000	-4.899985000
C	4.490374000	1.245782000	-4.665205000
H	4.418721000	0.561277000	-3.805844000
H	4.808315000	0.668896000	-5.548569000
H	5.271441000	1.989358000	-4.439202000
C	3.306157000	2.888006000	-6.095902000
H	3.705104000	2.327265000	-6.956414000
H	2.362938000	3.350341000	-6.411981000
H	4.021626000	3.692860000	-5.863513000
C	2.107243000	0.855244000	-5.224704000
H	1.125867000	1.281930000	-5.474265000
H	2.437323000	0.248864000	-6.083275000
H	1.976766000	0.185476000	-4.360892000

56
TS_AB

C	0.061437000	1.136073000	-0.958653000
C	-0.997018000	1.415072000	-1.991493000
C	-1.304389000	0.532611000	-3.035815000
C	-2.280185000	0.861103000	-3.974700000
C	-2.965489000	2.075811000	-3.893230000
C	-2.667921000	2.961483000	-2.858685000
C	-1.688218000	2.632059000	-1.920616000
H	-1.452143000	3.333771000	-1.114926000
H	-3.193959000	3.917046000	-2.783239000
H	-3.727838000	2.329750000	-4.634553000
H	-2.509286000	0.160067000	-4.782283000
H	-0.785395000	-0.424825000	-3.072100000
C	0.000000000	0.000000000	0.000000000
C	-0.423398000	-1.322533000	-0.291060000
O	-0.785326000	-1.821128000	-1.354776000
O	-0.394988000	-2.104090000	0.841281000
C	-0.762316000	-3.446082000	0.662386000
H	-0.695950000	-3.926272000	1.649202000
H	-1.791380000	-3.549093000	0.279117000
H	-0.092031000	-3.971429000	-0.039199000
S	1.678435000	0.556727000	-1.551681000
H	0.263328000	0.214351000	1.037040000
H	0.220781000	2.063090000	-0.390321000
N	1.604428000	3.748060000	-3.841205000
N	2.268319000	1.650644000	-2.989848000
C	2.108330000	2.965588000	-2.852679000
N	2.403304000	3.565648000	-1.677087000
C	3.434337000	3.068825000	-0.797118000
H	3.007778000	2.544389000	0.073390000
H	4.082858000	2.367825000	-1.335350000
H	4.039232000	3.919238000	-0.443018000
C	1.628188000	4.675583000	-1.165872000
H	2.205724000	5.614760000	-1.164812000
H	0.721063000	4.814521000	-1.765278000
H	1.328067000	4.457740000	-0.128341000
C	2.095982000	5.085288000	-4.082659000
H	2.994557000	5.277268000	-3.484149000
H	2.363026000	5.185180000	-5.148123000
H	1.339239000	5.852744000	-3.848428000
C	0.530082000	3.312213000	-4.701446000

H -0.270973000 4.069159000 -4.683795000
H 0.856343000 3.190303000 -5.748036000
H 0.117912000 2.365526000 -4.337110000
C 2.850656000 0.944299000 -4.160210000
C 3.954736000 0.034985000 -3.603270000
H 3.549146000 -0.697270000 -2.889463000
H 4.430188000 -0.523916000 -4.423283000
H 4.729475000 0.628110000 -3.093141000
C 3.508209000 1.912005000 -5.143983000
H 4.061583000 1.321072000 -5.889126000
H 2.787400000 2.532930000 -5.689026000
H 4.231352000 2.570917000 -4.639444000
C 1.807328000 0.081823000 -4.867957000
H 1.019521000 0.688554000 -5.333789000
H 2.290839000 -0.511656000 -5.658620000
H 1.337606000 -0.609881000 -4.154738000

56
Z-B
C 0.142197000 1.098331000 -0.985389000
C -0.901745000 1.379143000 -2.034097000
C -1.219116000 0.478986000 -3.060513000
C -2.190678000 0.800483000 -4.006556000
C -2.863595000 2.023515000 -3.948365000
C -2.558090000 2.925014000 -2.929720000
C -1.580810000 2.604268000 -1.986315000
H -1.338702000 3.317970000 -1.192923000
H -3.075549000 3.886531000 -2.871904000
H -3.623514000 2.270954000 -4.694397000
H -2.428034000 0.086316000 -4.800235000
H -0.713587000 -0.487016000 -3.070355000
C 0.000000000 0.000000000 0.000000000
C -0.350590000 -1.335881000 -0.296745000
O -0.621197000 -1.865394000 -1.376523000
O -0.379322000 -2.107874000 0.847957000
C -0.699725000 -3.458628000 0.659341000
H -0.690718000 -3.928554000 1.653703000
H -1.696831000 -3.593909000 0.206712000
H 0.029448000 -3.975935000 0.011974000
S 1.790205000 0.563835000 -1.617225000
H 0.232125000 0.232481000 1.040867000
H 0.309334000 2.034458000 -0.434392000
N 1.672681000 3.732518000 -3.842562000
N 2.315169000 1.626318000 -3.010501000
C 2.182264000 2.951808000 -2.861570000
N 2.509578000 3.542137000 -1.693701000
C 3.555322000 3.031340000 -0.838041000
H 3.141334000 2.505108000 0.037156000
H 4.189851000 2.330936000 -1.393425000
H 4.171992000 3.876365000 -0.492557000
C 1.755663000 4.657953000 -1.161550000
H 2.340467000 5.592116000 -1.174303000
H 0.834269000 4.803197000 -1.737185000
H 1.481654000 4.438652000 -0.117402000
C 2.163435000 5.071106000 -4.084085000
H 3.074107000 5.256590000 -3.502337000
H 2.408801000 5.174693000 -5.153957000
H 1.412389000 5.837421000 -3.830577000
C 0.593671000 3.300861000 -4.700938000
H -0.209387000 4.054462000 -4.667913000
H 0.916534000 3.195662000 -5.749829000
H 0.188797000 2.347638000 -4.346280000
C 2.912426000 0.928425000 -4.188147000
C 4.030839000 0.032836000 -3.639081000
H 3.639158000 -0.714198000 -2.933252000
H 4.513011000 -0.508240000 -4.466795000
H 4.797111000 0.633535000 -3.125050000
C 3.555456000 1.912665000 -5.163775000
H 4.119015000 1.332010000 -5.909009000
H 2.827082000 2.523378000 -5.709663000
H 4.268443000 2.579839000 -4.655922000
C 1.876138000 0.060061000 -4.896159000
H 1.082479000 0.661867000 -5.358547000
H 2.364482000 -0.526053000 -5.689101000
H 1.413990000 -0.638539000 -4.184655000

56
TS_Z-BC
C -0.104192000 1.018556000 -0.999656000
C -0.902926000 1.018854000 -2.256577000
C -1.213298000 -0.129445000 -3.002210000
C -1.953924000 -0.029863000 -4.178496000
C -2.399236000 1.210473000 -4.641037000
C -2.103628000 2.358151000 -3.905863000
C -1.366608000 2.258568000 -2.726655000
H -1.140254000 3.162666000 -2.153115000
H -2.449968000 3.336809000 -4.249425000
H -2.977825000 1.280685000 -5.565978000
H -2.185608000 -0.936118000 -4.745443000
H -0.851253000 -1.093388000 -2.635919000
C 0.000000000 0.000000000 0.000000000
C 0.117527000 -1.413430000 -0.162628000
O 0.055481000 -2.106414000 -1.174827000
O 0.322220000 -2.019223000 1.048717000
C 0.454115000 -3.417359000 1.019064000
H 0.587677000 -3.743096000 2.060175000
H -0.437899000 -3.908106000 0.595911000
H 1.326515000 -3.737435000 0.425001000
S 1.917027000 0.974759000 -1.468714000
H 0.181654000 0.350472000 1.018522000
H -0.179252000 2.006109000 -0.532075000
N 2.487555000 4.162011000 -3.501199000
N 2.682214000 1.920539000 -2.795037000
C 2.860591000 3.238093000 -2.587062000
N 3.400068000 3.674151000 -1.432383000
C 4.312700000 2.862290000 -0.661510000
H 3.802707000 2.386486000 0.191540000
H 4.741387000 2.073639000 -1.290871000
H 5.125765000 3.504150000 -0.287599000
C 2.949491000 4.885238000 -0.779925000
H 3.743276000 5.648330000 -0.738507000
H 2.078959000 5.300694000 -1.300368000
H 2.649331000 4.641444000 0.252315000
C 3.231366000 5.384941000 -3.707497000
H 4.187411000 5.345119000 -3.172297000
H 3.438937000 5.498282000 -4.784270000
H 2.669421000 6.272599000 -3.373215000
C 1.306072000 4.008176000 -4.317698000
H 0.634332000 4.866719000 -4.149219000
H 1.553370000 3.976470000 -5.391230000
H 0.774512000 3.091942000 -4.044012000
C 3.101119000 1.179457000 -4.026995000
C 4.055167000 0.072242000 -3.564405000
H 3.564589000 -0.585465000 -2.833142000
H 4.367431000 -0.536146000 -4.426435000
H 4.956546000 0.501899000 -3.099832000
C 3.868965000 2.071549000 -5.001122000
H 4.287920000 1.429360000 -5.790040000
H 3.239080000 2.821674000 -5.493909000
H 4.711472000 2.582392000 -4.510401000
C 1.892093000 0.547968000 -4.710545000
H 1.198236000 1.305139000 -5.101901000
H 2.222885000 -0.077474000 -5.553401000
H 1.347011000 -0.086193000 -3.997720000

56
Z-C
C -1.068264000 0.288633000 -0.781077000
C -1.551390000 -0.169932000 -2.082301000
C -0.945522000 -1.163166000 -2.876064000
C -1.477063000 -1.495687000 -4.118708000
C -2.622427000 -0.857987000 -4.599884000
C -3.240743000 0.121520000 -3.821903000
C -2.710555000 0.458458000 -2.580690000
H -3.193892000 1.232244000 -1.977451000
H -4.139322000 0.626951000 -4.185102000
H -3.033233000 -1.125846000 -5.576879000
H -0.990120000 -2.267384000 -4.721127000
H -0.057879000 -1.667849000 -2.494593000
C 0.000000000 0.000000000 0.000000000
C 1.099984000 -0.966701000 -0.158728000
O 1.212176000 -1.872299000 -0.958189000
O 2.003289000 -0.776137000 0.817837000
C 3.154991000 -1.592430000 0.763692000
H 3.800213000 -1.276656000 1.593221000
H 2.897481000 -2.656859000 0.878040000
H 3.686328000 -1.463930000 -0.191822000
S 1.717626000 1.664792000 -1.838854000
H 0.089301000 0.580954000 0.921159000
H -1.712014000 1.068873000 -0.359181000
N 4.359953000 4.146081000 -2.967501000
N 3.166309000 2.134317000 -2.742596000
C 3.945194000 3.089908000 -2.222241000
N 4.313529000 3.052354000 -0.922475000
C 4.395887000 1.796579000 -0.215200000
H 3.413702000 1.500116000 0.193164000
H 4.721472000 1.003129000 -0.899921000
H 5.137530000 1.895917000 0.591302000
C 4.335512000 4.241237000 -0.098957000
H 5.330495000 4.409795000 0.343778000
H 4.055760000 5.121051000 -0.690078000
H 3.603976000 4.132836000 0.720085000
C 5.660858000 4.748960000 -2.801178000
H 6.277388000 4.137216000 -2.131299000
H 6.164984000 4.811711000 -3.780737000
H 5.594091000 5.770014000 -2.388176000
C 3.487003000 4.775565000 -3.929315000
H 3.397605000 5.850318000 -3.694933000
H 3.870199000 4.686119000 -4.960011000
H 2.490204000 4.322266000 -3.878978000
C 3.462277000 1.407797000 -4.018117000
C 3.569018000 -0.078450000 -3.656663000
H 2.669087000 -0.402523000 -3.115374000

H	3.686331000	-0.681033000	-4.570052000
H	4.443769000	-0.257131000	-3.010958000
C	4.793775000	1.827112000	-4.638804000
H	5.013439000	1.136924000	-5.467221000
H	4.781820000	2.841864000	-5.055146000
H	5.622807000	1.750213000	-3.918655000
C	2.323355000	1.608709000	-5.014849000
H	2.265950000	2.650931000	-5.361297000
H	2.471457000	0.964955000	-5.894951000
H	1.367746000	1.346463000	-4.537596000

22
Z-Olefin

C	0.018660000	0.207271000	-1.337703000
C	-0.110470000	-0.629418000	-2.530111000
C	-0.037973000	0.040590000	-3.768760000
C	-0.147687000	-0.649066000	-4.971213000
C	-0.332752000	-2.031898000	-4.963891000
C	-0.406306000	-2.711785000	-3.746787000
C	-0.297509000	-2.025175000	-2.541302000
H	-0.355638000	-2.556839000	-1.591859000
H	-0.551273000	-3.795215000	-3.736070000
H	-0.419473000	-2.579696000	-5.905844000
H	-0.088382000	-0.106119000	-5.917781000
H	0.107607000	1.124303000	-3.777463000
C	0.000000000	0.000000000	0.000000000
C	-0.169088000	-1.227550000	0.794356000
O	-0.326832000	-2.366277000	0.408853000
O	-0.125545000	-0.922106000	2.097721000
C	-0.274140000	-2.004237000	3.000945000
H	-0.212696000	-1.576092000	4.008783000
H	-1.245989000	-2.502561000	2.864953000
H	0.524616000	-2.748447000	2.861552000
H	0.129759000	0.887972000	0.623647000
H	0.165165000	1.261365000	-1.600534000

34
Z-BartonS1

S	0.000000000	0.000000000	0.000000000
N	2.639322000	2.479240000	-1.133147000
N	1.452743000	0.464711000	-0.897235000
C	2.228257000	1.424490000	-0.382078000
N	2.600080000	1.398433000	0.918139000
C	2.691957000	0.149505000	1.631998000
H	1.705293000	-0.159131000	2.023024000
H	3.041175000	-0.643986000	0.958346000
H	3.415866000	0.263956000	2.452137000
C	2.607706000	2.593039000	1.733639000
H	3.597399000	2.768435000	2.185530000
H	2.331901000	3.467430000	1.132671000
H	1.867710000	2.489429000	2.545904000
C	3.942594000	3.079211000	-0.978418000
H	4.560969000	2.470034000	-0.307819000
H	4.442467000	3.135573000	-1.960757000
H	3.880717000	4.102820000	-0.570652000
C	1.757097000	3.112263000	-2.083555000
H	1.675066000	4.187663000	-1.848855000
H	2.125882000	3.021002000	-3.119579000
H	0.758885000	2.663666000	-2.019504000
C	1.747426000	-0.247555000	-2.182212000
C	1.846739000	-1.737829000	-1.837613000
H	0.941922000	-2.057784000	-1.302370000
H	1.962068000	-2.331912000	-2.756674000
H	2.717765000	-1.928097000	-1.190431000
C	3.083381000	0.169849000	-2.795206000
H	3.302221000	-0.515886000	-3.627606000
H	3.078161000	1.187364000	-3.204713000
H	3.909523000	0.083909000	-2.072761000
C	0.612620000	-0.028820000	-3.179512000
H	0.573832000	1.013014000	-3.529141000
H	0.747740000	-0.677702000	-4.057926000
H	-0.346168000	-0.270965000	-2.697917000

33
E-Barton

N	1.063052000	-1.663759000	1.376207000
N	0.000000000	0.000000000	0.000000000
C	0.055707000	-0.765552000	1.030054000
N	-1.005766000	-0.748274000	1.922940000
C	-2.233951000	-0.110791000	1.528605000
H	-2.199462000	0.990455000	1.642125000
H	-2.452566000	-0.327228000	0.475154000
H	-3.057389000	-0.491925000	2.153466000
C	-0.781405000	-0.792476000	3.348937000
H	-1.477680000	-1.490800000	3.843672000
H	0.243767000	-1.113439000	3.567499000
H	-0.924900000	0.206085000	3.804538000
C	0.770121000	-2.990051000	1.850168000
H	-0.304723000	-3.194352000	1.763730000
H	1.312286000	-3.742717000	1.247449000
H	1.064768000	-3.140317000	2.906862000
C	2.442026000	-1.268328000	1.443776000
H	2.840763000	-1.437113000	2.462590000
H	3.087484000	-1.832524000	0.743938000
H	2.544394000	-0.200307000	1.217996000
C	0.700443000	-0.155583000	-1.267392000
C	-0.323125000	0.259566000	-2.336382000
H	-0.694860000	1.275660000	-2.132618000
H	0.117223000	0.242599000	-3.346315000
H	-1.187442000	-0.423700000	-2.325823000
C	1.167204000	-1.578227000	-1.602329000
H	0.335349000	-2.295121000	-1.514574000
H	1.525603000	-1.611192000	-2.643728000
H	1.986409000	-1.925202000	-0.960737000
C	1.880664000	0.820711000	-1.346667000
H	2.698111000	0.533255000	-0.670376000
H	2.289623000	0.856931000	-2.368985000
H	1.551690000	1.835350000	-1.072269000

56
E-A

C	-1.323357000	-0.554838000	-0.387965000
C	0.000000000	0.000000000	0.000000000
C	1.241751000	-0.389870000	-0.737054000
O	2.154665000	0.375931000	-0.923420000
O	1.227549000	-1.645756000	-1.167136000
C	2.367466000	-2.083853000	-1.894567000
H	2.195767000	-3.141063000	-2.127762000
H	2.481648000	-1.507390000	-2.824336000
H	3.279266000	-1.976326000	-1.289148000
H	0.036466000	1.048904000	0.309663000
S	-0.693417000	-1.143315000	1.226483000
C	-2.539257000	0.299725000	-0.432690000
C	-2.704841000	1.419042000	0.394322000
C	-3.854125000	2.198372000	0.299227000
C	-4.855479000	1.868503000	-0.618294000
C	-4.700598000	0.750114000	-1.435605000
C	-3.548601000	-0.032478000	-1.344007000
H	-3.429986000	-0.929245000	-1.962725000
H	-5.483082000	0.478483000	-2.149201000
H	-5.757215000	2.482197000	-0.689116000
H	-3.972066000	3.069948000	0.948244000
H	-1.936759000	1.674734000	1.129192000
H	-1.304027000	-1.337345000	-1.153932000
N	-1.882374000	-4.934991000	-1.090427000
N	-2.903609000	-3.236451000	-2.450444000
C	-2.874443000	-4.022010000	-1.429477000
N	-3.932444000	-3.994224000	-0.537288000
C	-5.192430000	-3.420798000	-0.928558000
H	-5.259731000	-2.340690000	-0.698289000
H	-5.340729000	-3.543914000	-2.008432000
H	-6.009380000	-3.929713000	-0.390265000
C	-3.716368000	-4.065085000	0.887901000
H	-4.373256000	-4.814829000	1.361974000
H	-2.674828000	-4.328447000	1.106189000
H	-3.923002000	-3.087422000	1.363132000
C	-2.194247000	-6.255119000	-0.610494000
H	-3.271041000	-6.447284000	-0.699703000
H	-1.658927000	-7.015781000	-1.208701000
H	-1.903808000	-6.402998000	0.447279000
C	-0.496332000	-4.561422000	-1.030469000
H	-0.097021000	-4.738937000	-0.013868000
H	0.134289000	-5.136680000	-1.734467000
H	-0.375841000	-3.494311000	-1.243864000
C	-2.227273000	-3.418552000	-3.729302000
C	-3.262227000	-3.008418000	-4.789137000
H	-3.617419000	-1.983047000	-4.602623000
H	-2.836094000	-3.049238000	-5.804511000
H	-4.134985000	-3.680015000	-4.755505000
C	-1.783391000	-4.851541000	-4.051094000
H	-2.617437000	-5.560083000	-3.925934000
H	-1.456772000	-4.905316000	-5.101910000
H	-0.947438000	-5.193787000	-3.429102000
C	-1.034955000	-2.461764000	-3.845517000
H	-0.244342000	-2.705343000	-3.124111000
H	-0.597648000	-2.507034000	-4.855768000
H	-1.353581000	-1.423947000	-3.661201000

56
TS_E-AB

C	-1.118268000	-0.210415000	-0.878666000
C	0.000000000	0.000000000	0.000000000
C	1.358295000	0.042516000	-0.453393000
O	2.347860000	0.216211000	0.244736000
O	1.481206000	-0.125465000	-1.801124000
C	2.795815000	-0.137808000	-2.304813000
H	2.713907000	-0.283789000	-3.390486000
H	3.321260000	0.809361000	-2.101951000
H	3.390452000	-0.958350000	-1.871206000
H	-0.146304000	0.177641000	1.065641000
S	-0.876891000	-2.127385000	-0.523721000

C	-2.484135000	0.264586000	-0.528155000
C	-2.867570000	0.552242000	0.789922000
C	-4.170115000	0.951260000	1.081171000
C	-5.116738000	1.072557000	0.061377000
C	-4.743579000	0.801624000	-1.255282000
C	-3.438243000	0.406375000	-1.545004000
H	-3.149660000	0.191852000	-2.577930000
H	-5.472913000	0.901410000	-2.063694000
H	-6.138896000	1.383310000	0.292430000
H	-4.450133000	1.169169000	2.115346000
H	-2.143043000	0.452221000	1.600845000
H	-0.892437000	-0.077696000	-1.941762000
N	-2.086887000	-5.767243000	-0.192382000
N	-1.735281000	-3.692357000	-1.264779000
C	-2.389864000	-4.458004000	-0.392077000
N	-3.380051000	-3.930387000	0.357711000
C	-4.143981000	-2.790725000	-0.093248000
H	-3.843978000	-1.873510000	0.434593000
H	-3.987926000	-2.639191000	-1.166545000
H	-5.213446000	-2.976556000	0.097380000
C	-3.619424000	-4.331485000	1.726191000
H	-4.599054000	-4.822615000	1.845686000
H	-2.833424000	-5.013962000	2.069405000
H	-3.603261000	-3.435865000	2.368756000
C	-3.096458000	-6.765128000	0.079972000
H	-4.098365000	-6.342254000	-0.060689000
H	-2.973466000	-7.604417000	-0.625020000
H	-3.018816000	-7.166664000	1.104274000
C	-0.728017000	-6.250320000	-0.247178000
H	-0.514725000	-6.829525000	0.666667000
H	-0.554067000	-6.909948000	-1.114312000
H	-0.031364000	-5.405853000	-0.294074000
C	-1.426108000	-4.038315000	-2.672570000
C	-1.904160000	-2.847512000	-3.513207000
H	-1.378841000	-1.929271000	-3.214336000
H	-1.704162000	-3.028706000	-4.580329000
H	-2.985914000	-2.687778000	-3.384572000
C	-2.192888000	-5.278151000	-3.133954000
H	-3.273514000	-5.173264000	-2.950062000
H	-2.049840000	-5.397195000	-4.218561000
H	-1.844817000	-6.200973000	-2.652675000
C	0.075143000	-4.223527000	-2.900362000
H	0.462873000	-5.117637000	-2.393869000
H	0.279199000	-4.331507000	-3.976605000
H	0.621320000	-3.345044000	-2.527598000

56
E-B

C	-1.199780000	0.348817000	-0.512925000
C	0.000000000	0.000000000	0.000000000
C	1.228583000	-0.098539000	-0.794751000
O	2.296560000	-0.462568000	-0.349186000
O	1.068411000	0.264080000	-2.074857000
C	2.205019000	0.136336000	-2.907714000
H	1.901274000	0.486126000	-3.902241000
H	3.042741000	0.747079000	-2.537726000
H	2.534172000	-0.912775000	-2.968738000
H	0.129101000	-0.275281000	1.048479000
S	-1.162245000	-2.557536000	-0.955805000
C	-2.466287000	0.394205000	0.213647000
C	-2.602439000	-0.071474000	1.534056000
C	-3.837304000	-0.041900000	2.172326000
C	-4.965001000	0.454228000	1.510422000
C	-4.844279000	0.923301000	0.202352000
C	-3.607352000	0.892057000	-0.437889000
H	-3.515135000	1.250613000	-1.466932000
H	-5.719563000	1.312443000	-0.324221000
H	-5.934331000	0.472502000	2.015093000
H	-3.925997000	-0.414627000	3.196169000
H	-1.737013000	-0.483900000	2.056512000
H	-1.246372000	0.625125000	-1.569818000
N	-2.448337000	-5.961977000	0.127388000
N	-1.679111000	-4.230347000	-1.265211000
C	-2.609897000	-4.745567000	-0.451878000
N	-3.725552000	-4.048055000	-0.147043000
C	-4.253533000	-3.051892000	-1.048123000
H	-3.816523000	-2.058719000	-0.848009000
H	-4.014255000	-3.319172000	-2.085135000
H	-5.347196000	-3.016428000	-0.931155000
C	-4.290687000	-4.048180000	1.185036000
H	-5.317549000	-4.448537000	1.192236000
H	-3.670690000	-4.645856000	1.863297000
H	-4.319274000	-3.013914000	1.567147000
C	-3.550433000	-6.868516000	0.348039000
H	-4.448759000	-6.503856000	-0.164856000
H	-3.294764000	-7.861286000	-0.059852000
H	-3.778386000	-6.990436000	1.420625000
C	-1.163386000	-6.392858000	0.625449000
H	-1.257175000	-6.662773000	1.691308000
H	-0.782480000	-7.276713000	0.086064000
H	-0.436511000	-5.577461000	0.534462000
C	-1.068576000	-4.963812000	-2.418231000
C	-1.344072000	-4.116872000	-3.666123000
H	-0.980340000	-3.091553000	-3.510856000
H	-0.840961000	-4.557252000	-4.540103000
H	-2.425155000	-4.073559000	-3.873537000
C	-1.702580000	-6.335335000	-2.644976000
H	-2.798397000	-6.268749000	-2.726818000
H	-1.324126000	-6.731070000	-3.599476000
H	-1.451723000	-7.066358000	-1.866740000
C	0.439518000	-5.098087000	-2.218115000
H	0.680897000	-5.782944000	-1.392632000
H	0.911781000	-5.487632000	-3.132531000
H	0.866888000	-4.110880000	-1.987913000

22
E-Olefin

C	-1.167519000	0.470062000	-0.475542000
C	0.000000000	0.000000000	0.000000000
C	1.203667000	0.019687000	-0.848007000
O	1.264896000	0.433891000	-1.984531000
O	2.256715000	-0.488357000	-0.199292000
C	3.480226000	-0.521914000	-0.915810000
H	4.222832000	-0.961528000	-0.238954000
H	3.389524000	-1.138502000	-1.822970000
H	3.794224000	0.491713000	-1.207933000
H	0.112500000	-0.407514000	1.007334000
C	-2.450536000	0.529093000	0.218577000
C	-2.627806000	0.082352000	1.541297000
C	-3.873296000	0.162443000	2.152908000
C	-4.967495000	0.689229000	1.460114000
C	-4.807153000	1.136057000	0.148951000
C	-3.559539000	1.056399000	-0.465022000
H	-3.431495000	1.406263000	-1.493072000
H	-5.658007000	1.549086000	-0.398554000
H	-5.944833000	0.750335000	1.945561000
H	-3.995323000	-0.188444000	3.180751000
H	-1.782775000	-0.331162000	2.096370000
H	-1.162360000	0.854376000	-1.502103000

23
B-Episulfide

C	-0.620008000	0.621009000	-1.196562000
C	0.000000000	0.000000000	0.000000000
C	1.358722000	-0.609002000	-0.162212000
O	1.848236000	-0.936816000	-1.213794000
O	1.956370000	-0.740959000	1.015609000
C	3.256409000	-1.319069000	1.003821000
H	3.593634000	-1.336229000	2.046661000
H	3.223383000	-2.342420000	0.601720000
H	3.945568000	-0.716291000	0.394306000
H	-0.187397000	0.453886000	0.978229000
S	-1.376706000	-0.963332000	-0.685768000
C	-1.402475000	1.881367000	-1.098716000
C	-2.224864000	2.168313000	-0.000657000
C	-2.914657000	3.375057000	0.065857000
C	-2.797970000	4.311164000	-0.965016000
C	-1.988766000	4.029236000	-2.064144000
C	-1.295653000	2.820151000	-2.130764000
H	-0.659742000	2.599599000	-2.992476000
H	-1.894879000	4.753175000	-2.877656000
H	-3.342744000	5.257141000	-0.911288000
H	-3.552239000	3.587210000	0.927954000
H	-2.337519000	1.430621000	0.798617000
H	-0.027367000	0.521823000	-2.112404000

57
B-A

C	-1.434269000	-0.127252000	0.398324000
C	0.000000000	0.000000000	0.000000000
C	0.511587000	-0.613772000	-1.250145000
O	1.546695000	-0.285945000	-1.781982000
O	-0.279146000	-1.580744000	-1.718990000
C	0.169607000	-2.253338000	-2.884516000
H	-0.598237000	-2.999152000	-3.124473000
H	0.286404000	-1.552341000	-3.724916000
H	1.132855000	-2.754357000	-2.702861000
H	0.518215000	0.930128000	0.253667000
S	-0.339918000	-1.121891000	1.430458000
C	-2.163554000	1.034657000	0.981260000
C	-1.647843000	1.781148000	2.048419000
C	-2.353550000	2.868966000	2.555500000
C	-3.589786000	3.223409000	2.010204000
C	-4.113796000	2.480991000	0.951982000
C	-3.404366000	1.393591000	0.441956000
H	-3.818126000	0.809514000	-0.385188000
H	-5.082648000	2.747220000	0.521168000
H	-4.145343000	4.073666000	2.414122000
H	-1.939840000	3.441108000	3.390248000
H	-0.695023000	1.483032000	2.494467000
H	-2.043264000	-0.723875000	-0.289098000
S	-1.891678000	-2.197475000	3.817536000

N	-5.092567000	-4.054028000	4.606155000
N	-2.983605000	-3.581794000	3.684821000
C	-4.300722000	-3.345148000	3.763665000
N	-4.874524000	-2.366160000	3.033595000
C	-4.285970000	-1.924322000	1.793350000
H	-3.589269000	-1.088438000	1.964887000
H	-3.719726000	-2.743083000	1.332899000
H	-5.091663000	-1.613556000	1.110945000
C	-5.908193000	-1.509667000	3.573060000
H	-6.840370000	-1.580310000	2.989538000
H	-6.119150000	-1.774204000	4.615668000
H	-5.561781000	-0.462390000	3.546782000
C	-6.449563000	-4.418767000	4.272464000
H	-6.651761000	-4.196523000	3.217602000
H	-6.587439000	-5.501367000	4.434066000
H	-7.186949000	-3.885446000	4.895958000
C	-4.638414000	-4.433377000	5.923377000
H	-5.351354000	-4.058140000	6.677154000
H	-4.568364000	-5.528047000	6.040328000
H	-3.655687000	-3.989179000	6.119442000
C	-2.389573000	-4.940308000	3.475140000
C	-1.554935000	-4.858401000	2.192354000
H	-0.826710000	-4.038960000	2.262573000
H	-1.024813000	-5.808607000	2.027587000
H	-2.201376000	-4.666619000	1.321161000
C	-3.453923000	-6.015774000	3.260287000
H	-4.156876000	-5.739010000	2.459561000
H	-2.943657000	-6.938547000	2.945938000
H	-4.026484000	-6.252126000	4.165470000
C	-1.486317000	-5.310824000	4.648871000
H	-2.063776000	-5.476795000	5.569660000
H	-0.931046000	-6.233851000	4.423964000
H	-0.768474000	-4.497252000	4.829206000

57
TS_AB

C	-1.318846000	-0.092171000	0.643014000
C	0.000000000	0.000000000	0.000000000
C	0.366872000	-0.761674000	-1.150000000
O	1.440104000	-0.720664000	-1.739209000
O	-0.626125000	-1.599443000	-1.576814000
C	-0.310096000	-2.417328000	-2.676951000
H	-1.203703000	-3.021274000	-2.886404000
H	-0.053886000	-1.824206000	-3.569958000
H	0.538080000	-3.086823000	-2.458477000
H	0.722647000	0.738391000	0.351513000
S	-0.568928000	-1.335411000	1.780341000
C	-1.894613000	1.125086000	1.299375000
C	-1.220995000	1.799564000	2.326736000
C	-1.766150000	2.944814000	2.901506000
C	-3.000847000	3.433217000	2.466157000
C	-3.683068000	2.766302000	1.449708000
C	-3.131922000	1.621322000	0.871197000
H	-3.667822000	1.103462000	0.070436000
H	-4.651365000	3.137253000	1.102909000
H	-3.430352000	4.328797000	2.922649000
H	-1.226859000	3.458906000	3.701753000
H	-0.268464000	1.402057000	2.686631000
H	-2.058457000	-0.580524000	-0.002075000
S	-1.733903000	-1.983576000	3.666262000
N	-4.802333000	-3.918274000	4.438875000
N	-2.680934000	-3.450710000	3.547455000
C	-4.031352000	-3.301978000	3.522823000
N	-4.613685000	-2.512793000	2.612075000
C	-4.007588000	-2.235101000	1.330134000
H	-3.707950000	-1.179609000	1.275620000
H	-3.123620000	-2.863020000	1.177010000
H	-4.744133000	-2.433604000	0.535269000
C	-5.820362000	-1.761319000	2.892848000
H	-6.679275000	-2.137297000	2.315114000
H	-6.056769000	-1.801691000	3.961922000
H	-5.647419000	-0.710031000	2.613584000
C	-6.112821000	-4.447083000	4.123945000
H	-6.280567000	-4.431499000	3.040809000
H	-6.162853000	-5.492693000	4.468515000
H	-6.915917000	-3.881810000	4.623264000
C	-4.369917000	-4.092341000	5.808428000
H	-5.149193000	-3.695067000	6.478691000
H	-4.214179000	-5.154793000	6.056445000
H	-3.441042000	-3.538988000	5.984569000
C	-1.982443000	-4.780333000	3.551869000
C	-1.036253000	-4.799399000	2.348967000
H	-0.287374000	-3.999301000	2.414738000
H	-0.510957000	-5.765482000	2.314263000
H	-1.590756000	-4.670674000	1.406955000
C	-2.977095000	-5.926762000	3.376004000
H	-3.612643000	-5.781461000	2.488892000
H	-2.402125000	-6.851285000	3.221030000
H	-3.619617000	-6.084390000	4.250867000
C	-1.172207000	-4.962594000	4.832481000
H	-1.815645000	-5.048436000	5.718440000
H	-0.569185000	-5.879930000	4.760975000
H	-0.489507000	-4.112336000	4.976033000

57
B-B

C	-1.101071000	0.098320000	0.770105000
C	0.000000000	0.000000000	0.000000000
C	0.052848000	-0.740069000	-1.265374000
O	0.975465000	-0.687466000	-2.049118000
O	-1.058849000	-1.456772000	-1.517763000
C	-1.058101000	-2.197157000	-2.723153000
H	-2.018507000	-2.726159000	-2.765438000
H	-0.960433000	-1.535368000	-3.597465000
H	-0.232159000	-2.924908000	-2.739524000
H	0.938439000	0.495440000	0.254066000
S	1.495698000	-2.674960000	1.167798000
C	-1.229034000	0.824455000	2.030599000
C	-0.146296000	1.462023000	2.663692000
C	-0.318483000	2.114279000	3.879776000
C	-1.573496000	2.149077000	4.494661000
C	-2.658260000	1.525852000	3.876768000
C	-2.486250000	0.872771000	2.658556000
H	-3.336612000	0.379495000	2.178971000
H	-3.644724000	1.547140000	4.347593000
H	-1.702997000	2.660268000	5.451985000
H	0.536907000	2.598068000	4.358726000
H	0.845287000	1.432128000	2.206620000
H	-2.007154000	-0.410531000	0.431649000
S	0.189235000	-2.243925000	2.660746000
N	-3.154816000	-3.343513000	3.548610000
N	-0.850365000	-3.592614000	3.137535000
C	-2.132367000	-3.513716000	2.687460000
N	-2.389056000	-3.583621000	1.376865000
C	-1.499634000	-4.266327000	0.457347000
H	-0.926157000	-3.541454000	-0.134774000
H	-0.790943000	-4.885770000	1.015591000
H	-2.104641000	-4.902743000	-0.208174000
C	-3.496716000	-2.889086000	0.753124000
H	-4.287886000	-3.584352000	0.430266000
H	-3.923182000	-2.151606000	1.442463000
H	-3.114254000	-2.361605000	-0.133483000
C	-4.459337000	-3.935919000	3.341946000
H	-4.420710000	-4.673339000	2.531957000
H	-4.759024000	-4.453681000	4.267338000
H	-5.226120000	-3.180053000	3.108264000
C	-3.008136000	-2.573617000	4.764197000
H	-3.816372000	-1.826160000	4.810438000
H	-3.078505000	-3.211972000	5.660083000
H	-2.046987000	-2.048464000	4.767261000
C	-0.365836000	-4.599360000	4.132998000
C	0.819500000	-5.326595000	3.492073000
H	1.589895000	-4.614795000	3.162646000
H	1.261248000	-6.027355000	4.216981000
H	0.496867000	-5.900892000	2.609891000
C	-1.455759000	-5.626635000	4.432027000
H	-1.850814000	-6.076128000	3.507542000
H	-1.012591000	-6.436601000	5.030272000
H	-2.292619000	-5.210125000	5.008072000
C	0.096975000	-3.925019000	5.422710000
H	-0.734763000	-3.449330000	5.959708000
H	0.548495000	-4.672991000	6.091581000
H	0.852910000	-3.157707000	5.201231000

35
B-B*

S	2.345529000	1.516945000	1.591871000
S	1.077401000	1.417614000	-0.002993000
N	-0.569408000	-1.269437000	-1.899274000
N	0.000000000	0.000000000	0.000000000
C	0.304230000	-0.945506000	-0.922758000
N	1.492692000	-1.564131000	-0.896495000
C	2.255214000	-1.708127000	0.325834000
H	3.102109000	-1.007460000	0.350607000
H	1.621165000	-1.487444000	1.190663000
H	2.626993000	-2.743438000	0.389596000
C	2.177773000	-1.981807000	-2.101459000
H	2.237657000	-3.079051000	-2.180130000
H	1.672104000	-1.583162000	-2.988190000
H	3.203248000	-1.580349000	-2.077366000
C	-0.704993000	-2.618805000	-2.404258000
H	-0.158488000	-3.321917000	-1.764898000
H	-1.771967000	-2.894764000	-2.391568000
H	-0.339262000	-2.712765000	-3.439600000
C	-1.437146000	-0.286304000	-2.507390000
H	-1.306488000	-0.323980000	-3.601207000
H	-2.498536000	-0.482180000	-2.283345000
H	-1.173380000	0.718008000	-2.158406000
C	-1.235971000	-0.004921000	0.845297000
C	-0.777683000	0.062201000	2.305270000
H	-0.124594000	0.929060000	2.480454000
H	-1.656924000	0.127156000	2.964570000
H	-0.208825000	-0.840864000	2.576562000

C	-2.026082000	-1.299501000	0.659668000
H	-1.390851000	-2.186689000	0.806568000
H	-2.815561000	-1.329420000	1.425418000
H	-2.515894000	-1.373460000	-0.319539000
C	-2.117013000	1.203932000	0.539038000
H	-2.534507000	1.162686000	-0.476247000
H	-2.956786000	1.240206000	1.249086000
H	-1.536835000	2.132509000	0.640028000

35
TS_B-B*C

S	2.686775000	2.118471000	2.581164000
S	2.788404000	2.105659000	0.655692000
N	-0.626656000	-1.200045000	-1.996588000
N	0.000000000	0.000000000	0.000000000
C	0.245930000	-0.734054000	-1.019266000
N	1.562609000	-1.171552000	-1.180235000
C	2.465219000	-1.012521000	-0.099640000
H	2.822581000	0.055852000	-0.006215000
H	1.990796000	-1.245780000	0.862119000
H	3.363828000	-1.628115000	-0.255668000
C	2.165905000	-1.249632000	-2.491667000
H	2.687964000	-2.209839000	-2.635164000
H	1.402747000	-1.145429000	-3.270572000
H	2.902042000	-0.436209000	-2.627090000
C	-0.600113000	-2.571310000	-2.435303000
H	0.060489000	-3.165461000	-1.790501000
H	-1.614247000	-3.008113000	-2.379858000
H	-0.249955000	-2.679507000	-3.479892000
C	-1.441635000	-0.312084000	-2.779774000
H	-1.142463000	-0.346814000	-3.845472000
H	-2.514596000	-0.574444000	-2.732289000
H	-1.325967000	0.721750000	-2.434365000
C	-1.278588000	0.318371000	0.614157000
C	-1.003344000	0.322575000	2.126416000
H	-0.202585000	1.037189000	2.369867000
H	-1.906050000	0.598103000	2.694582000
H	-0.678470000	-0.676211000	2.459418000
C	-2.411635000	-0.680155000	0.345568000
H	-2.095116000	-1.708402000	0.581328000
H	-3.267883000	-0.438328000	0.995145000
H	-2.768998000	-0.659227000	-0.690975000
C	-1.709694000	1.732952000	0.208486000
H	-1.990488000	1.786986000	-0.853297000
H	-2.579068000	2.056180000	0.802337000
H	-0.889015000	2.445645000	0.381605000

35
B-C

S	2.918710000	1.718039000	2.763447000
S	3.505163000	1.739465000	0.918031000
N	-0.578524000	-1.293785000	-1.951518000
N	0.000000000	0.000000000	0.000000000
C	0.269944000	-0.760037000	-0.992661000
N	1.611348000	-1.141233000	-1.149309000
C	2.492904000	-0.982039000	-0.068004000
H	2.815717000	0.114517000	0.056919000
H	2.024206000	-1.222859000	0.895190000
H	3.422089000	-1.547912000	-0.224106000
C	2.211954000	-1.254996000	-2.460096000
H	2.710585000	-2.229291000	-2.590452000
H	1.448839000	-1.141788000	-3.237672000
H	2.965376000	-0.460646000	-2.604146000
C	-0.486101000	-2.672481000	-2.359129000
H	0.219323000	-3.215198000	-1.716649000
H	-1.473061000	-3.162079000	-2.269081000
H	-0.154760000	-2.783824000	-3.408927000
C	-1.453839000	-0.469477000	-2.740729000
H	-1.159437000	-0.502355000	-3.807547000
H	-2.506937000	-0.798903000	-2.680788000
H	-1.400377000	0.574160000	-2.410154000
C	-1.285404000	0.263317000	0.626035000
C	-0.987748000	0.294621000	2.134263000
H	-0.201847000	1.033293000	2.353013000
H	-1.889183000	0.553121000	2.712177000
H	-0.630052000	-0.690035000	2.475423000
C	-2.380205000	-0.782248000	0.384705000
H	-2.018904000	-1.794637000	0.624516000
H	-3.235666000	-0.569487000	1.045193000
H	-2.755071000	-0.785498000	-0.645778000
C	-1.772752000	1.656842000	0.212536000
H	-2.062068000	1.691216000	-0.848021000
H	-2.648792000	1.953594000	0.810263000
H	-0.975750000	2.399208000	0.373937000

2
B-S2

S	0.000000000	0.000000000	0.000000000
S	0.000000000	0.000000000	1.908580000

41
B-D

S	0.000000000	0.000000000	0.000000000
N	-2.387646000	-2.394500000	1.476022000
N	-0.761432000	-1.530933000	0.027278000
C	-2.059511000	-1.556189000	0.496100000
N	-2.957935000	-0.708098000	-0.013269000
C	-2.865468000	-0.205005000	-1.368228000
H	-2.538481000	0.847037000	-1.376437000
H	-2.166783000	-0.808052000	-1.958327000
H	-3.861180000	-0.265886000	-1.833153000
C	-3.998517000	-0.110240000	0.805828000
H	-4.992520000	-0.522522000	0.573291000
H	-3.768436000	-0.247782000	1.874843000
H	-4.012876000	0.969952000	0.593913000
C	-3.729198000	-2.916579000	1.637911000
H	-4.314801000	-2.765019000	0.724364000
H	-3.651380000	-3.996970000	1.833252000
H	-4.232576000	-2.441468000	2.494041000
C	-1.456913000	-2.777465000	2.519256000
H	-1.890710000	-2.462916000	3.482520000
H	-1.288318000	-3.865348000	2.517984000
H	-0.506401000	-2.252334000	2.395532000
C	-0.018060000	-2.709042000	-0.550826000
C	0.379894000	-2.336678000	-1.981437000
H	1.029992000	-1.449067000	-2.004725000
H	0.939165000	-3.168922000	-2.433112000
H	-0.507505000	-2.142175000	-2.602426000
C	-0.934670000	-3.926636000	-0.610940000
H	-1.867366000	-3.707454000	-1.152384000
H	-0.407148000	-4.716039000	-1.165411000
H	-1.181446000	-4.326691000	0.380007000
C	1.231788000	-3.007904000	0.267119000
H	0.985245000	-3.346828000	1.281947000
H	1.810783000	-3.802888000	-0.225054000
H	1.876363000	-2.118658000	0.339699000
S	2.209227000	-0.116972000	2.557623000
S	1.983903000	-1.874484000	3.626601000
S	1.513852000	-1.266797000	5.585826000
S	-0.490920000	-1.479518000	5.931369000
S	0.300590000	0.510986000	1.979766000
S	-3.298639000	-0.392111000	4.504930000
S	-1.510830000	0.290402000	5.195901000

41
TS_B-DE

S	0.000000000	0.000000000	0.000000000
N	-2.414352000	-2.407152000	2.038619000
N	-0.909155000	-1.672827000	0.386284000
C	-2.097108000	-1.614552000	0.987549000
N	-3.007740000	-0.699260000	0.592956000
C	-3.082295000	-0.227695000	-0.771039000
H	-2.703257000	0.802919000	-0.861774000
H	-2.491083000	-0.877450000	-1.426684000
H	-4.134092000	-0.247550000	-1.099061000
C	-3.878353000	-0.023238000	1.531117000
H	-4.934399000	-0.305165000	1.389728000
H	-3.579702000	-0.249581000	2.561021000
H	-3.788700000	1.064373000	1.377573000
C	-3.753324000	-2.909462000	2.252207000
H	-4.381137000	-2.704019000	1.377176000
H	-3.706867000	-4.001174000	2.398351000
H	-4.224068000	-2.464645000	3.144434000
C	-1.439441000	-2.780769000	3.035858000
H	-1.803737000	-2.476358000	4.031057000
H	-1.262630000	-3.868566000	3.055064000
H	-0.491412000	-2.269515000	2.839987000
C	-0.201712000	-2.881002000	-0.102043000
C	0.074158000	-2.655039000	-1.596296000
H	0.711385000	-1.773203000	-1.763046000
H	0.599131000	-3.526358000	-2.016247000
H	-0.866881000	-2.516830000	-2.150702000
C	-1.068989000	-4.133369000	0.021878000
H	-2.044961000	-3.998247000	-0.468744000
H	-0.552862000	-4.961603000	-0.486177000
H	-1.236963000	-4.439386000	1.061554000
C	1.127600000	-3.075472000	0.626401000
H	0.981875000	-3.305278000	1.690809000
H	1.681468000	-3.910991000	0.172118000
H	1.749238000	-2.171699000	0.550876000
S	1.275471000	-0.190767000	3.110959000
S	3.030139000	0.913373000	3.184553000
S	4.332057000	0.137249000	1.758263000
S	4.462596000	1.527793000	0.215190000
S	-0.148434000	0.735390000	1.919231000
S	1.309259000	2.072497000	-0.801387000
S	3.044204000	1.113263000	-1.257164000

41
B-E

S	0.000000000	0.000000000	0.000000000
N	-2.779586000	-2.833355000	2.549150000
N	-1.452865000	-2.104744000	0.691271000
C	-2.477106000	-2.012260000	1.474413000
N	-3.390704000	-1.003659000	1.266315000
C	-3.499040000	-0.358254000	-0.013346000
H	-2.923533000	0.585030000	-0.061052000
H	-3.117650000	-1.021620000	-0.798762000
H	-4.556271000	-0.118541000	-0.217559000
C	-4.037134000	-0.316825000	2.357858000
H	-5.136634000	-0.410380000	2.312603000
H	-3.691946000	-0.714983000	3.319448000
H	-3.788404000	0.758877000	2.330003000
C	-4.117436000	-3.301450000	2.802228000
H	-4.781956000	-3.016320000	1.976861000
H	-4.125291000	-4.404050000	2.882634000
H	-4.537199000	-2.896655000	3.741912000
C	-1.796651000	-3.188672000	3.534897000
H	-2.139212000	-2.881309000	4.540463000
H	-1.596813000	-4.275638000	3.573302000
H	-0.852971000	-2.671738000	3.327976000
C	-0.716173000	-3.315393000	0.333901000
C	-0.455818000	-3.202631000	-1.177304000
H	0.110243000	-2.288312000	-1.410735000
H	0.122556000	-4.065023000	-1.545107000
H	-1.407385000	-3.163344000	-1.730641000
C	-1.483242000	-4.624360000	0.563564000
H	-2.475231000	-4.588764000	0.086019000
H	-0.924294000	-5.456918000	0.107757000
H	-1.621773000	-4.863436000	1.624997000
C	0.643380000	-3.375371000	1.042492000
H	0.538111000	-3.534102000	2.124481000
H	1.245899000	-4.205647000	0.640863000
H	1.202291000	-2.440494000	0.889423000
S	0.325576000	-0.126829000	3.315236000
S	1.986351000	0.974877000	3.886964000
S	3.637002000	0.198483000	2.898965000
S	4.048321000	1.456382000	1.303153000
S	-0.622176000	0.866028000	1.773496000
S	1.387837000	1.428638000	-0.748849000
S	3.253467000	0.608645000	-0.415087000

8
B-S8

S	0.000000000	0.000000000	0.000000000
S	0.402781000	-0.295059000	3.306199000
S	2.013170000	0.858600000	3.912576000
S	3.698742000	0.142574000	2.943928000
S	4.060392000	1.374754000	1.318751000
S	-0.601869000	0.757163000	1.830367000
S	1.445102000	1.274671000	-0.762196000
S	3.295099000	0.436144000	-0.363579000

4. Preparation of substrates

4.1 Aldehydes

Aldehyde S5

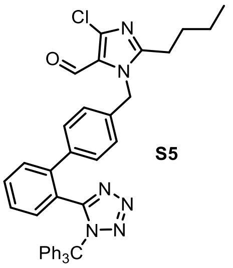

S5

Dess–Martin periodinane (268 mg, 0.63 mmol, 1.25 eq) was added to a stirred and cooled solution (0 °C) of trityl-losartan (340 mg, 0.51 mmol, 1.00 eq) in anhydrous dichloromethane (8 mL) under an atmosphere of argon. The cooling bath was removed, and the mixture was stirred at rt for 6 h. The mixture was then cooled to 0 °C and poured into a stirred mixture of 5% aq. sodium thiosulfate (5 mL) and saturated aq. sodium hydrogen carbonate (5 mL). After stirring at rt for 20 min, the mixture was extracted with ethyl acetate (15 mL) and the organic phase was separated, washed with brine (10 mL), then dried over anhydrous magnesium sulfate and concentrated under reduced pressure. The resulting crude material was purified by flash column chromatography (0-30% EtOAc in heptane) to give aldehyde **S5** as a yellow solid (306 mg, 91%).

^{1}H NMR (600 MHz, $CDCl_3$) δ 9.73 (s, 1H), 7.93 (dd, *J* = 7.5, 1.3 Hz, 1H), 7.47 (dqd, *J* = 14.9, 7.4, 1.4 Hz, 2H), 7.37 – 7.31 (m, 4H), 7.29 – 7.24 (m, 6H), 7.09 (t, *J* = 7.3 Hz, 2H), 6.94 – 6.88 (m, 6H), 6.84 (d, *J* = 8.1 Hz, 2H), 5.45 (s, 2H), 2.60 – 2.42 (m, 2H), 1.64 (dt, *J* = 15.4, 7.7 Hz, 2H), 1.35 – 1.23 (m, 2H), 0.86 (t, *J* = 7.4 Hz, 3H); **^{13}C NMR** (151 MHz, $CDCl_3$) δ 178.0, 164.0, 154.7, 143.2, 141.5, 141.4 (3C), 141.2, 134.2, 130.9, 130.5, 130.4 (6C), 130.1, 130.0 (2C), 128.4 (3C), 127.9, 127.8 (6C), 126.4, 126.2 (2C), 124.4, 83.1, 48.1, 29.3, 26.7, 22.5, 13.8; **IR** (neat) v 2959, 1710, 1666, 1516, 1489, 1448, 1276, 1074, 759, 701, 634 cm^{-1}; **HRMS** (ESI^+) m/z: $[M + Na]^+$ calculated 685.2453 for $C_{41}H_{35}N_6O(^{35}Cl)Na^+$, 685.2427 found.

4.2 *N*-Tosylimines

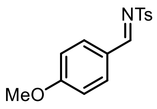

S6

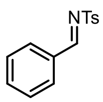

10

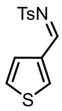

S7

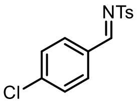

S8

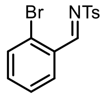

S9

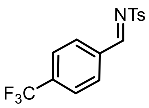

S10

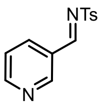

S11

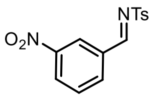

S12

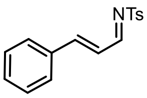

S13

All *N*-Tosylimines were prepared according to previously described procedures: **S6-9** and **10** by the protocol of Liu,[17] **S10-11** by the protocol of Schneider,[18] and **S12-13** by the protocol of Njardarson.[19]

4.3. Isothiouronium salts

A. General procedures

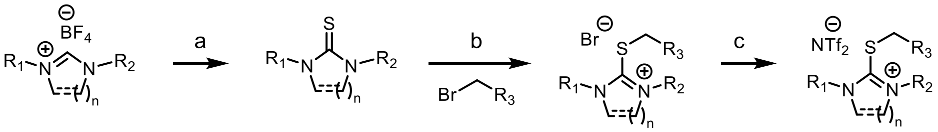

a) General procedure for the preparation of the non-commercially available thioureas: To a suspension of imidazolium salt (1.00 eq) in tetrahydrofuran (0.25 M) at rt was added potassium *tert*-butoxide (1.05 eq). The resulting mixture was stirred at rt for 1.5 h. Elemental sulfur (1.05 eq) was added and the resulting mixture was stirred at rt for 12 h. The crude mixture was filtered through a thin pad of celite, washed with dichloromethane and the solvent was removed under vacuum. The resulting solid was triturated with heptane (3 × 5 mL) to afford the pure thiourea.

b) General procedure for the preparation isothiuronium bromide salts: To a solution of thiourea (1.00 eq) in dichloromethane (0.5 M) at rt was added the corresponding electrophile (1.00 – 1.00 eq). The resulting mixture was stirred at rt for 10 h whereupon the solvent was removed under vacuum. The resulting solid was then washed with 5 mL of cooled acetone (0 °C), and dried under vacuum to afford the desired salt.

c) General procedure for the preparation isothiuronium bis(trifluoromethyl)sulfonyl)amide salts: To a solution of isothiouronium bromide (1.00 eq) in water (1.0 M) at rt was added lithium bis(trifluoromethanesulphonyl)imide (1.05 eq). The resulting mixture was stirred at rt for 10 min. The crude material was extracted with dichloromethane (3 × 5 mL). The organic phases were combined, washed with brine, and dried over magnesium sulfate. The solvent was removed under reduced pressure to afford the desired isothiouronium salt.

B. Characterisation

B1. Non-commercial thioureas

Thiourea S14

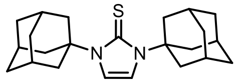

Thiourea **S14** was prepared from 1,3-di-(1-adamantyl)imidazolium tetrafluoroborate (120 mg, 0.283 mmol) according to general procedure A and obtained as white solid in 56% yield (58 mg, 0.157 mmol).

^{1}H NMR (400 MHz, $CDCl_3$) *δ* 6.78 (s, 2H), 2.64 (d, *J* = 2.6 Hz, 2H), 2.18 (s, 6H), 1.72 (m, 12H); **^{13}C NMR** (101 MHz, $CDCl_3$) *δ* 159.5, 113.3 (2C), 60.6 (2C), 39.7 (6C), 36.2 (6C), 30.2 (6C); **IR** (neat) *v* 2905, 2847, 1455, 1383, 1359, 1337, 1320, 1306, 1286, 1251, 1240, 1202, 1190, 1174, 1096, 1016, 983, 835, 713, 677 cm^{-1}; **HRMS** (ESI$^+$) m/z: [M+H]$^+$ calcd for $[C_{23}H_{33}N_2S]^+$ 369.2359, found 369.2362

Thiourea S15

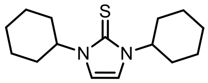

Thiourea **S15** was prepared from 1,3-dicyclohexylimidazolium tetrafluoroborate (1.25 g, 3.90 mmol) according to general procedure A and obtained as white solid in 82% yield (840 mg, 3.18 mmol).

^{1}H NMR (400 MHz, $CDCl_3$) *δ* 6.71 (s, 2H), 4.77 (tt, *J* = 11.9, 3.8 Hz, 2H), 2.08–2.06 (m, 4H), 1.88–1.82 (m, 4H), 1.77–1.71 (m, 2H), 1.56–1.13 (m, 10H); **^{13}C NMR** (100 MHz, $CDCl_3$) *δ* 160.1, 113.7 (2C), 55.9 (2C), 32.6 (4C), 25.58 (4C), 25.55 (4C); **IR** (neat) ν 2932, 2854, 1411, 1310, 1242 cm^{-1}; **HRMS** (ESI$^+$) m/z: [M+H]$^+$ calcd for $C_{15}H_{25}N_2S$ 265.1738, found 265.1732.

Thiourea S16

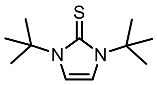

Thiourea **S16** was prepared from 1,3-di-*tert*-butyllimidazolium tetrafluoroborate (2.40 g, 8.95 mmol) according to general procedure A and obtained as white solid in 91% yield (1.73 g, 8.15 mmol).

^{1}H NMR (400 MHz, $CDCl_3$) *δ* 6.80 (s, 2H), 1.82 (s, 18H); **^{13}C NMR** (100 MHz, $CDCl_3$): *δ* 160.9, 113.6 (2C), 59.5 (2C), 28.5 (6C); **IR** (neat) ν 3359, 3176, 2961, 2921, 2851, 1465, 1403, 1363, 1221, 1206 cm^{-1}; **HRMS** (ESI) m/z [M+Na]$^+$ calcd for $C_{11}H_{20}N_2NaS$ 235.1245; found 235.1239.

Thiourea S17

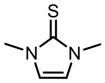

Thiourea **S17** was prepared from dimethyl-1H-imidazol-3-ium iodide (22.0 g, 98.2 mmol) according to general procedure A and obtained as white solid in 57% yield (7.18 g, 56.0 mmol).

^{1}H NMR (400 MHz, $CDCl_3$) *δ* 6.65 (s, 2H), 1.58 (s, 6H).

Spectral data were in agreement with those previously reported.[20]

B2. Non-commercial bromides

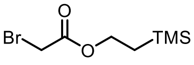

S18

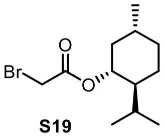

S19

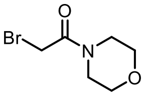

S20

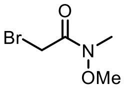

S21

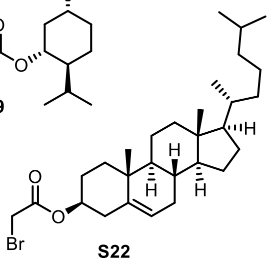

S22

Bromides **S18**-**22** were not commercially available and were prepared according to previously described procedures. **S18** according to Lumb,[21] **S19** according to Stentzel,[22] **S20** and **S21** according to Waser,[23] and **S22** according to Yan.[24]

B3. Isothiouronium bromides

Isothiouronium bromide 1

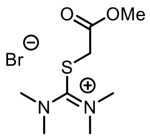

Isothiouronium bromide **1** was prepared from tetramethylthiourea (1.32 g, 10.0 mmol) according to general procedure A and obtained as a white solid in 97% yield (1.38 g, 4.84 mmol).

^{1}H NMR (400 MHz, $CDCl_3$) *δ* 3.99 (s, 2H), 3.69 (s, 3H), 3.39 (s, 12H); **^{13}C NMR** (101 MHz, $CDCl_3$) *δ* 174.2, 168.1, 53.7, 44.8 (4C), 36.0; **IR** (neat) *v* 3370, 2924, 1731, 1604, 1439, 1396, 1309, 1259, 1203, 1168, 1112, 870 cm^{-1}; **HRMS** (ESI) m/z: $[M]^+$ calcd for $C_8H_{17}N_2O_2S$ 205.1001; found 205.1005.

Isothiouronium bromide S23

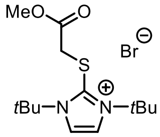

Isothiouronium bromide **S23** was prepared from thiourea **S16** (1.59 g, 7.50 mmol, 1.0 eq) and methyl bromoacetate (0.71 mL, 7.50 mmol, 1.00 eq) according to general procedure B and obtained as white solid in 95% yield (2.60 g, 7.12 mmol).

^{1}H NMR (400 MHz, $CDCl_3$) *δ* 7.99 (s, 2H), 4.19 (s, 2H), 3.74 (s, 3H), 1.86 (s, 18H); **^{13}C NMR** (101 MHz, $CDCl_3$) *δ* 167.4, 134.0, 124.0 (2C), 65.1 (2C), 53.4, 41.8, 30.6 (6C); **IR** (neat) *v* 3456, 3408, 1727, 1638, 1556, 1435, 1301, 1190, 1170 cm^{-1}; **HRMS** (ESI) m/z: $[M^+]$ calcd for $C_{14}H_{25}N_2O_2S^+$ 285.1631; found 285.1632.

Isothiouronium bromide S24

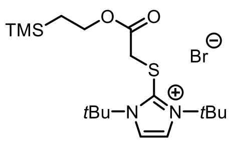

Isothiouronium bromide **S24** was prepared from thiourea **S16** (127 mg, 0.60 mmol, 1.0 eq) and bromide **S18** (151 mg, 0.63 mmol, 1.05 eq) according to general procedure B and obtained as yellow oil in 72% yield (194 mg, 0.43 mmol). The product was not able to be purified by crystallisation but was used in the following step without further purification.

^{1}H NMR (400 MHz, $CDCl_3$) *δ* 8.00 (s, 2H), 4.23 – 4.11 (m, 2H), 4.00 (s, 2H), 1.81 (s, 18H), 0.99 – 0.86 (m, 2H), -0.05 (s, 9H); **^{13}C NMR** (101 MHz, $CDCl_3$) *δ* 166.7, 134.0, 123.9 (2C), 65.2, 65.0 (2C), 42.1, 30.5 (6C), 17.3, -1.7 (3C); **IR** (neat) *v* 3456, 3402, 2955, 1724, 1185, 1044, 860 cm^{-1}; **HRMS** (ESI) m/z $[M^+]$ calcd for $C_{18}H_{35}N_2O_2SSi^+$, 371.2183; found 371.2185.

Contaminated with a small amount of *N,N*-di-tert-butylthiourea starting material (<5%).

Isothiouronium bromide S25

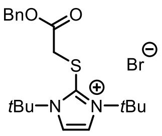

Isothiouronium bromide **S25** was prepared from thiourea **S16** (127 mg, 0.6 mmol, 1.00 eq) and benzyl bromoacetate (144 mg, 0.63 mmol, 1.05 eq) according to general procedure B and obtained a yellow oil 65% yield (172 mg, 0.39 mmol). The product could not be purified by crystallisation and but was used in the following step without further purification

^{1}H NMR (700 MHz, $CDCl_3$) *δ* 7.99 (s, 2H), 7.32 (s, 5H), 5.16 (s, 2H), 4.20 (s, 2H), 1.83 (s, 18H); **^{13}C NMR** (176 MHz, $CDCl_3$): *δ* 166.8, 134.6, 133.94, 128.9, 128.8 (4C), 123.9 (2C), 68.3, 65.1, 42.0 (2C), 30.6 (6C); **IR** (neat) *ν* 2982, 1733, 1556, 1402, 1182, 747, 700 cm^{-1}; **HRMS** (ESI) m/z [M^+] calcd for $C_{20}H_{29}N_2O_2S^+$ 361.1944; found 316.1942.

Contaminated with a small amount of *N,N*-di-tert-butylthiourea starting material (<5%)

Isothiouronium bromide S26

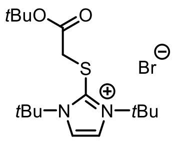

Isothiouronium bromide **S26** was prepared from thiourea **S16** (127 mg, 0.6 mmol, 1.00 eq) and *tert*-butyl bromoacetate (125 mg, 0.63 mmol, 1.05 eq) according to general procedure B, and obtained as a yellow oil in 65% yield (172 mg, 0.39 mmol). The product was not able to be purified by crystallisation but was used in the following step without further purification.

^{1}H NMR (700 MHz, $CDCl_3$) *δ* 8.06 (s, 2H), 3.97 (s, 2H), 1.87 (s, 18H), 1.45 (s, 9H); **^{13}C NMR** (176 MHz, $CDCl_3$) *δ* 165.9, 134.5, 124.0 (2C), 84.5, 65.1, 43.4, 30.6 (6C), 28.0 (3C); **HRMS** (ESI) m/z [M^+] calcd for $C_{17}H_{31}N_2O_2S^+$ 327.2101; found: 327.2113; **IR** (neat) *ν* 2976, 2933, 1721, 1556, 1145, 925, 677 cm^{-1}.

Isothiouronium bromide S1

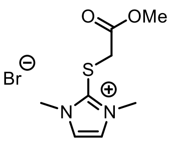

Isothiouronium bromide **S1** was prepared from thiourea **S17** (961 mg, 7.50 mmol, 1.00 eq) and methyl bromoacetate (0.71 mL, 7.50 mmol, 1.00 eq) according to general procedure B, and obtained as a white solid in quantitative yield (2.11 g, 7.50 mmol).

^{1}H NMR (400 MHz, DMSO-d_6) *δ* 7.97 (s, 2H), 4.03 (s, 2H), 3.93 (s, 6H), 3.65 (s, 3H); **^{13}C NMR** (101 MHz, DMSO): *δ* 168.9, 138.6, 125.2 (2C), 52.8, 36.3 (2C), 35.3; **IR** (neat) *ν* 3397, 2956, 1731, 1632, 1567, 1504, 1437, 1307, 1241, 1202, 1169, 998 cm^{-1}; **HRMS** (ESI) m/z: [M^+] calcd for $C_8H_{13}N_2O_2S$: 201.0692; found 201.0684.

Isothiouronium bromide S27

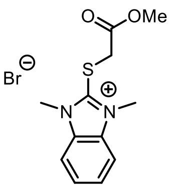

Isothiouronium bromide **S27** was prepared from 1,3-dimethyl-1,3-dihydro-2*H*-benzo[*d*]imidazole-2-thione (891 mg, 5.00 mmol, 1.00 eq) and methyl bromoacetate (0.50 mL, 5.25 mmol, 1.05 eq) according to general procedure B, and obtained as a white solid in 97% yield (1.60 g, 4.83 mmol).

^{1}H NMR (400 MHz, CD_3OD) δ 8.02 – 7.95 (m, 2H), 7.81 – 7.73 (m, 2H), 4.28 (s, 6H), 4.12 (s, 2H), 3.71 (s, 3H); **^{13}C NMR** (101 MHz, CD_3OD) *δ* 170.0, 148.8, 134.0, 128.9, 114.4, 53.7, 36.8, 34.1.; **IR** (neat) *ν* 3348, 2947, 2837, 1652, 1407, 1015 cm^{-1}; **HRMS** (ESI) m/z: [M^+] calcd for $C_{10}H_{17}N_2O_2S^+$ 251.0849, found 251.0841.

Isothiouronium bromide S28

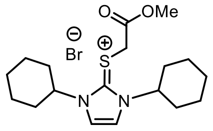

Isothiouronium bromide **S28** was prepared from thiourea **S15** (397 mg, 1.50 mmol, 1.00 eq) and methyl bromoacetate (0.156 mL, 1.65 mmol, 1.05 eq) according to general procedure B, and obtained as a white solid in 82% yield (513 mg, 4.56 mmol).

^{1}H NMR (400 MHz, $CDCl_3$) δ 8.02 (s, 2H), 4.75 (tt, *J* = 12.1, 3.8 Hz, 2H), 4.06 (s, 2H), 3.74 (s, 3H), 2.16 (t, *J* = 5.4 Hz, 4H), 1.97 (d, *J* = 13.7 Hz, 4H), 1.82 (ddd, *J* = 24.9, 12.4, 3.5 Hz, 6H), 1.57 – 1.43 (m, 4H), 1.41 – 1.30 (m, 2H); **^{13}C NMR** (101 MHz, $CDCl_3$) δ 168.0, 135.0, 123.4 (2C), 60.2 (2C), 53.5, 38.0, 33.8 (4C), 25.5 (4C), 24.8 (2C); **IR** (neat) *v* 3381, 3311, 2313, 2186, 2166, 1271, 1261, 995 cm^{-1}; **HRMS** (ESI) m/z [M^+] calcd for $C_{18}H_{29}N_2O_2S$, 337.1944; found 337.1945.

Isothiouronium bromide S29

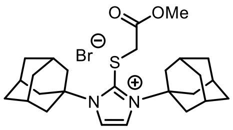

Isothiouronium bromide **S29** was prepared from thiourea **S14** (255 mg, 0.692 mmol, 1.00 eq) and methyl bromoacetate (69 µL, 0.727 mmol, 1.05 eq) according to general procedure B, and obtained as a white solid in 96% yield (345 mg, 0.661 mmol).

^{1}H NMR (400 MHz, $CDCl_3$) δ 8.11 (s, 2H), 4.13 (s, 2H), 3.77 (s, 3H), 2.55 (br s, 6H), 2.45 (br s, 6H), 2.30 (br s, 6H), 1.85–1.70 (m, 12H); **^{13}C NMR** (101 MHz, $CDCl_3$) δ 167.4, 133.4, 124.0 (2C), 67.0, 53.4, 43.1, 42.2, 35.4, 30.3; **IR** (neat): *v* 3463, 3399, 2907, 2853, 1733, 1457, 1436, 1337, 1308, 1266, 1184, 1165, 1062 cm^{-1}; **HRMS** (ESI) m/z: [M^+] calcd for $C_{26}H_{37}N_2O_2S$ 441.2570; found 441.2580.

Isothiouronium bromide S30

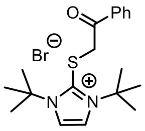

Isothiouronium bromide **S30** was prepared from thiourea **S16** (85 mg, 0.40 mmol, 1.00 eq) and methyl bromoacetate (88 mg, 0.44 mmol, 1.10 eq) according to general procedure B, and obtained as a white solid in 95% yield (156 mg, 0.38 mmol).

^{1}H NMR (400 MHz, $CDCl_3$) δ 8.25 (d, *J* = 8.3 Hz, 2H), 7.67 (s, 2H), 7.58 (t, *J* = 7.2 Hz, 1H), 7.49 (t, *J* = 7.2 Hz, 2H) 5.49 (s, 2H), 1.91 (s, 18H); **^{13}C NMR** (101 MHz, $CDCl_3$) δ 194.0, 134.6, 134.5, 129.3, 129.1, 124.0 (2C), 122.2, 64.6, 50.9, 30.2 (6C); **IR** (neat) *v* 2986, 1770, 1677, 1429, 1375, 1275, 1265, 1154, 1082 cm^{-1}; **HRMS** (ESI) m/z: [M^+] calcd for $C_{19}H_{27}N_2OS$ 331.1839, found 331.1834.

Isothiouronium bromide S31

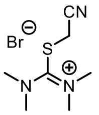

Isothiouronium bromide **S31** was prepared from tetramethyl thiourea (172 mg, 1.30 mmol, 1.00 eq) and bromoacetonitrile (100 µl, 1.43 mmol, 1.10 eq) according to general procedure B, and obtained as a white solid in 92% yield (301 mg, 1.19 mmol).

^{1}H NMR (400 MHz, DMSO-d_6) δ 4.35 (s, 2H), 3.28 (s, 12H); **^{13}C NMR** (101 MHz, DMSO-d_6) δ 171.5, 116.6, 43.8 (4C), 19.1; **IR** (neat) v 3392, 2938, 2248, 2076, 1606, 1504, 1461, 1393, 1251, 1204, 1168, 1111, 1056, 933, 870, 728, 697 cm^{-1}; **HRMS** (ESI^+) m/z: [M^+] calcd for $C_7H_{14}N_3S^+$ 172.0903, found 172.0908.

Isothiouronium bromide S32

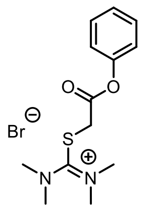

Isothiouronium bromide **S32** was prepared from tetramethylthiourea (132 mg, 1.00 mmol, 1.00 eq) and phenyl bromoacetate (215 mg, 1.00 mmol, 1.00 eq) according to general procedure B, and obtained as orange solid in 95% yield (329 mg, 0.950 mmol).

^{1}H NMR (400 MHz, $CDCl_3$) *δ* 7.43 – 7.34 (m, 2H), 7.29 – 7.22 (m, 1H), 7.21 – 7.13 (m, 2H), 4.42 (s, 2H), 3.50 (s, 12H); **^{13}C NMR** (101 MHz, $CDCl_3$) *δ* 174.5, 167.1, 150.5, 129.8 (2C), 126.8, 121.4 (2C), 45.0 (4C), 36.8; **HRMS** (ESI) m/z: $[M]^+$ calcd for $C_{13}H_{19}N_2O_2S$ 267.1162; found 267.1167; **IR** (neat) *ν* 1276, 1262, 764, 750 cm^{-1}.

Isothiouronium bromide S33

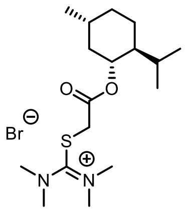

Isothiouronium bromide **S33** was prepared from tetramethylthiourea (132 mg, 1.00 mmol, 1.00 eq) and bromoacetate **S19** (277 mg, 1.00 mmol, 1.00 eq) according to general procedure B, and obtained as orange solid in 98% yield (402 mg, 0.980 mmol).

^{1}H NMR (400 MHz, $CDCl_3$) *δ* 4.70 (td, *J* = 10.9, 4.4 Hz, 1H), 3.92 (d, *J* = 4.1 Hz, 2H), 3.48 (s, 12H), 1.97 – 1.88 (m, 1H), 1.79 (dtd, *J* = 14.0, 7.0, 2.7 Hz, 1H), 1.74 – 1.64 (m, 2H), 1.54 – 1.35 (m, 2H), 1.09 – 0.97 (m, 2H), 0.90 (dd, *J* = 6.8, 4.7 Hz, 6H), 0.90 – 0.81 (m, 1H), 0.74 (d, *J* = 7.0 Hz, 3H); **^{13}C NMR** (101 MHz, $CDCl_3$) *δ* 174.8, 167.3, 77.7, 46.9, 45.0 (4C), 40.8, 36.4, 34.0, 31.6, 26.5, 23.4, 22.0, 20.8, 16.4; **HRMS** (ESI) m/z: $[M]^+$ calcd for $C_{17}H_{33}N_2O_2S$ 329.2257, found 329.2260; **IR** (neat) *ν* 1263, 1055, 1033, 764, 749, 703 cm^{-1}.

Isothiouronium bromide S34

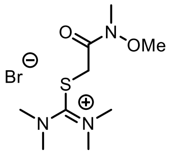

Isothiouronium bromide **S34** was prepared from tetramethylthiourea (132 mg, 1.00 mmol, 1.00 eq) and bromide **S21** (182 mg, 1.00 mmol, 1.00 eq) according to general procedure B, and obtained as orange solid in 98% yield (308 mg, 0.98 mmol).

^{1}H NMR (400 MHz, $CDCl_3$) *δ* 4.31 (s, 2H), 3.87 (s, 3H), 3.46 (s, 12H), 3.20 (s, 3H); **^{13}C NMR** (101 MHz, $CDCl_3$) *δ* 175.5, 167.0, 62.6, 44.8, 36.1, 32.9; **HRMS** (ESI) m/z $[M]^+$ calcd for $C_9H_{20}N_3O_2S$ 234.1271, found 234.1267; **IR** (neat) *ν* 1656, 1602, 1461, 1392, 1263, 1168, 1114, 993, 748, 725, 696 cm^{-1}.

Isothiouronium bromide S35

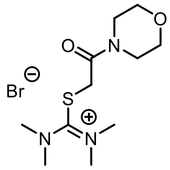

Isothiouronium bromide **S35** was prepared from tetramethylthiourea (132 mg, 1.00 mmol, 1.00 eq) and bromide **S20** (208 mg, 1.00 mmol, 1.00 eq) according to general procedure B, and obtained as orange solid in 99% yield (336 mg, 0.99 mmol).

^{1}H NMR (400 MHz, $CDCl_3$) *δ* 4.55 (s, 2H), 3.76 (d, *J* = 3.9 Hz, 4H), 3.66 – 3.61 (m, 2H), 3.56 – 3.51 (m, 2H), 3.41 (s, 12H); **^{13}C NMR** (101 MHz, $CDCl_3$) *δ* 175.8, 164.8, 66.9, 66.5, 46.8, 44.6 (4C), 42.9, 38.6;

HRMS (ESI) m/z: $[M]^+$ calcd for $C_{11}H_{22}N_3O_2S$ 260.1427, found 260.1426; **IR** (neat) *ν* 1521, 1451, 1276, 1261, 1115, 1022, 750 cm^{-1}.

Isothiouronium bromide S36

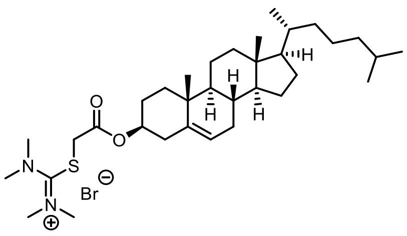

Isothiouronium bromide **S36** was prepared from tetramethylthiourea (132 mg, 1.00 mmol, 1.00 eq) and bromide **S22** (508 mg, 1.00 mmol, 1.00 eq) according to general procedure B, and obtained as orange solid in 99% yield (632 mg, 0.99 mmol).

^{1}H NMR (400 MHz, $CDCl_3$) δ 5.39 (d, *J* = 5.0 Hz, 1H), 4.60 (ddd, *J* = 16.3, 10.9, 5.1 Hz, 1H), 4.04 (d, *J* = 1.6 Hz, 2H), 3.49 (s, 12H), 2.64 (t, *J* = 5.6 Hz, 1H). 2.45 – 2.25 (m, 2H), 2.09 – 1.93 (m, 2H), 1.93 – 1.76 (m, 3H), 1.69 – 1.23 (m, 12H), 1.23 – 1.05 (m, 7H), 1.02 (s, 3H), 1.00 – 0.94 (m, 1H), 0.91 (d, *J* = 6.5 Hz, 3H), 0.86 (dd, *J* = 6.6, 1.7 Hz, 6H), 0.68 (s, 3H); **^{13}C NMR** (101 MHz, $CDCl_3$) δ 174.1, 166.6, 138.5, 123.0, 76.6, 56.3, 55.7, 49.6, 44.4, 41.9, 39.3, 39.1, 37.5, 36.5, 36.13, 36.11, 35.8, 35.4, 34.5, 31.5, 31.4, 27.8, 27.6, 27.3, 23.9, 23.4, 22.5, 22.2, 20.6, 18.9, 18.4; **HRMS** (ESI) m/z: $[M]^+$ calcd for $C_{34}H_{59}N_2O_2S$ 559.4292, found 559.4295; **IR** (neat) *ν* 904, 723, 646 cm^{-1}.

Isothiouronium bromide S37

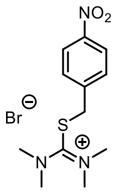

Isothiouronium bromide **S37** was prepared from tetramethylthiourea (1.74 g, 13.2 mmol, 1.00 eq) and *p*-nitrobenzyl bromide (6.70 g, 13.2 mmol, 1.00 eq) according to general procedure B, and obtained as yellow solid in quantitative yield (4.58 g, 1.00 mmol).

^{1}H NMR (400 MHz, $CDCl_3$) δ 8.18 (d, *J* = 9.0 Hz, 2H), 7.84 (d, *J* = 9.0 Hz, 2H), 4.82 (s, 2H), 3.46 (s, 12H).

Spectral data were in agreement with those previously reported.[25]

B3. Thiouronium bistriflimides

Isothiouronium bistriflimide 5a

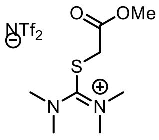

Isothiouronium bistriflimide **5a** was prepared from bromide **1** (856 mg, 3.00 mmol) according to general procedure C, and obtained as clear oil in 91% yield (1.33 g, 2.74 mmol).

^{1}H NMR (400 MHz, $CDCl_3$) δ 3.79 (s, 2H), 3.77 (s, 3H), 3.33 (s, 12H); **^{13}C NMR** (101 MHz, $CDCl_3$): δ 174.5, 168.0, 119.9 (*q*, $J_{C–F}$ = 321.1 Hz, 2C), 53.8, 44.1 (4C), 35.0; **^{19}F NMR** (377 MHz, $CDCl_3$) δ -78.87; **IR** (neat) *ν* 2950, 1739, 1606, 1468, 1349, 1307, 1229, 1196, 1168, 1052, 910 cm^{-1}; **HRMS** (ESI) m/z: $[M]^+$ calcd for $C_8H_{17}N_2O_2S$ 205.1003; found 205.1005.

Isothiouronium bistriflimide 5b

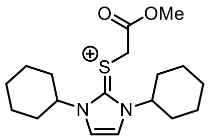

Isothiouronium bistriflimide **5b** was prepared from bromide **S28** (626 mg, 1.50 mmol) according to general procedure C, and obtained as white solid in 65% yield (625 mg, 0.986 mmol).

^{1}H NMR (400 MHz, $CDCl_3$) δ 7.68 (s, 2H), 4.76 – 4.65 (m, 2H), 3.75 (s, 5H), 2.03 (m, 8H), 1.84 – 1.67 (m, 6H), 1.58 – 1.42 (m, 4H), 1.38 – 1.23 (m, 2H); **^{13}C NMR** (101 MHz, $CDCl_3$): *δ* 167.6, 135.1, 122.5 (2C), 119.9 (*q*, $J_{C\text{–}F}$ = 321.1 Hz, 2C), 60.1, 53.5, 37.5, 33.5, 25.2, 24.6; **IR** (neat) *v* 2926, 2856, 1743, 1455, 1353, 1306, 1195, 1138, 1057 cm^{-1}; **HRMS** (ESI) m/z $[M]^+$ calcd for $C_{18}H_{29}N_2O_2S$ 337.1945; found 337.1947.

Isothiouronium bistriflimide 5c

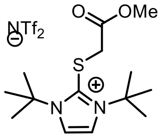

Isothiouronium bistriflimide **5c** was prepared from bromide **S23** (548 mg, 1.50 mmol) according to general procedure C, and obtained as yellow oil in 93% yield (814 mg, 1.40 mmol).

^{1}H NMR (400 MHz, $CDCl_3$) *δ* 7.61 (s, 2H), 3.89 (s, 2H), 3.77 (s, 3H), 1.82 (s, 18H); **^{13}C NMR** (101 MHz, $CDCl_3$): *δ* 167.2, 134.7, 130.0 (2C), 119.9 (*q*, $J_{C\text{–}F}$ = 321.1 Hz, 2C), 65.3, 53.4, 41.6, 30.2; **^{19}F NMR** (377 MHz, $CDCl_3$) δ -78.85; **IR** (neat) *v* 2956, 2921, 2851, 1740, 1376, 1350, 1227, 1187, 1137, 1057 cm^{-1}; **HRMS** (ESI) m/z $[M]^+$ calcd for $C_{14}H_{25}N_2O_2S$ 285.1631; found 285.1632.

Isothiouronium bistriflimide S38

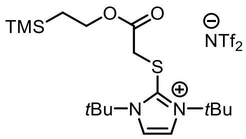

Isothiouronium bistriflimide **S38** was prepared from bromide **S24** (548 mg, 1.50 mmol) according to general procedure C, and obtained as yellow oil in 58% yield (156 mg, 0.239 mmol).

^{1}H NMR (400 MHz, $CDCl_3$) *δ* 7.65 (s, 2H), 4.31 – 4.24 (m, 2H), 3.88 (s, 2H), 1.85 (s, 18H), 1.07 – 0.99 (m, 2H), 0.04 (s, 9H); **^{13}C NMR** (101 MHz, $CDCl_3$) *δ* 166.9, 134.9, 123.0 (2C), 119.9 (*q*, J_{CF} = 321.1 Hz, 2C), 65.5, 65.3, 42.2 (2C), 30.3 (6C), 17.5, -1.5 (3C); **^{19}F NMR** (377 MHz, $CDCl_3$) *δ* -78.78; **HRMS** (ESI) m/z $[M]^+$ calcd for $C_{18}H_{35}N_2O_2SSi^+$: 371.2183; found: 371.2195; **IR** (neat) *v* 3192, 2956, 1733, 1560, 1431, 1376, 1181, 1136, 1058, 866, 653 cm^{-1}.

Isothiouronium bistriflimide S39

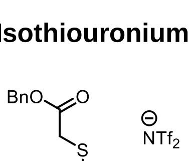

Isothiouronium bistriflimide **S39** was prepared from bromide **S25** (230 mg, 0.521 mmol) according to general procedure C, and obtained as yellow oil in 60% yield (199 mg, 0.310 mmol).

^{1}H NMR (400 MHz, $CDCl_3$) *δ* 7.62 (s, 2H), 7.36 (d, *J* = 0.9 Hz, 5H), 5.21 (s, 2H), 3.93 (s, 2H), 1.80 (s, 18H); **^{13}C NMR** (101 MHz, $CDCl_3$) *δ* 166.8, 134.6, 134.0, 128.9, 128.8 (4C), 123.9 (2C), 119.8 (*q*, $J_{C\text{–}F}$ = 321.5 Hz, 2 × C), 68.4, 65.3, 42.0 (2C), 30.2 (6C); **^{19}F NMR** (377 MHz, $CDCl_3$) *δ* -78.77; **HRMS** (ESI) m/z $[M^+]$

calcd for $C_{20}H_{29}N_2O_2S^+$ 361.1944; found 316.1960; **IR** (neat) *ν* 3179, 2991, 1737, 1559, 1349, 1181, 1057, 740 cm^{-1}

Isothiouronium bistriflimide S40

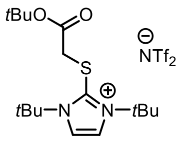

Isothiouronium bistriflimide **S40** was prepared from bromide **S26** (150 mg, 0.368 mmol) according to general procedure C, and obtained as yellow oil in 91% yield (204 mg, 0.336 mmol).

^{1}H NMR (700 MHz, $CDCl_3$) δ 7.61 (s, 2H), 3.79 (s, 2H), 1.82 (s, 18H), 1.46 (s, 9H); **^{13}C NMR** (176 MHz, $CDCl_3$) δ 165.8, 135.3, 122.8 (2C), 119.9 (*q*, J_{CF} = 321.1 Hz, 2C), 84.5, 65.2, 43.1 (2C), 30.2 (6C), 27.9 (3C); **^{19}F NMR** (377 MHz, $CDCl_3$) δ -78.8; **HRMS** (ESI) m/z: [M^+] calcd for $C_{17}H_{31}N_2O_2S^+$ 327.2101; found 327.2112; **IR** (neat) *ν* 3451, 3391, 2975, 1729, 1433, 1367, 1184, 1131, 851, 684 cm^{-1}

5. *Z*-selective olefination of aldehydes

5.1 General Procedure

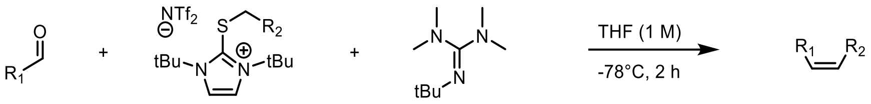

A flame-dried Schlenk tube equipped with a stir bar was charged with aldehyde (0.20 mmol, 1.00 eq) and isothiouronium salt (128 mg, 0.22 mmol, 1.10 eq), then the atmosphere was exchanged for argon. Tetrahydrofuran (0.20 mL, 1 M) was added, then the resulting mixture was stirred at –78°C for 10 min. At this point, Barton's base (0.24 mmol, 48 µL, 1.20 eq) was added and the resulting mixture was stirred at –78°C for 2 h. The reaction mixture was then allowed to warm up to rt and was neutralised by the addition of 1 mL of saturated aqueous solution of ammonium chloride. The mixture was extracted three times with 2 mL of dichloromethane; the organic phases were then combined and dried over magnesium sulfate. The solvent was then removed under reduced pressure and the crude residue* was purified by chromatography on silica gel to give the corresponding olefin.

NB: Where high molecular weight aldehydes were used (e.g. spiramycin), it was advantageous to the lower concentration of the reaction to prevent the mixture becoming prohibitively viscous at cryogenic temperature. This modification was not found to be detrimental to yield or stereoselectivity.

*Stereoselectivity was determined by analysis of the ^{1}H-NMR of the crude material; unless otherwise stated, the *Z/E* selectivity was >20:1.

5.2 Characterization

Olefin 2c

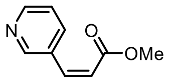

Olefin **2c** was prepared from 3-pyridinecarboxyaldehyde (18.8 µL, 0.200 mmol) according to general procedure, and obtained as white solid in 95% yield (28.1 mg, 0.172 mmol).

^{1}H NMR (400 MHz, $CDCl_3$) δ 8.59 (s, 1H), 8.48 (d, *J* = 4.2 Hz, 1H), 8.03 (d, *J* = 7.9 Hz, 1H), 7.22 (dd, *J* = 7.9, 5.3 Hz, 1H), 6.87 (d, *J* = 12.6 Hz, 1H), 6.01 (d, *J* = 12.6 Hz, 1H), 3.65 (s, 3H); **^{13}C NMR** (101 MHz, $CDCl_3$) δ 166.2, 150.9, 149.9, 140.1, 136.8, 130.7, 123.0, 121.7, 51.7; **IR** (neat) ν 3189, 2356, 1759, 1628, 1453, 1247, 1182, 1180, 956 cm^{-1}; **HRMS** (ESI) m/z $[M+H]^+$ calcd for $C_9H_{10}NO_2$ 164.0706; found 164.0707.

The equivalent Still-Gennari reaction gave an *Z/E* selectivity of 11.5:1[26]

Olefin 2d

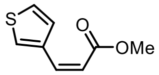

Olefin **2d** was prepared from 3-thiophenecarboxyaldehyde (17.5 µL, 0.200 mmol) according to general procedure, and obtained as clear oil in 83% yield (28 mg, 0.166 mmol).

^{1}H NMR (400 MHz, $CDCl_3$) *δ* 8.08 (d, *J* = 2.8 Hz, 1H), 7.54 (dd, *J* = 5.0, 1.1 Hz, 1H), 7.27 (dd, *J* = 5.0, 3.0 Hz, 1H), 6.88 (d, *J* = 12.6 Hz, 1H), 5.83 (d, *J* = 12.6 Hz, 1H), 3.75 (s, 3H).

Spectral data were in agreement with those previously reported.[27]

Olefin 2e

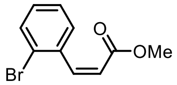

Olefin **2e** was prepared from 2-bromobenzaldehyde (23.3 µL, 0.200 mmol) according to general procedure, and obtained as white solid in 75% yield (36.0 mg, 0.149 mmol).

^{1}H NMR (400 MHz, $CDCl_3$) *δ* 7.58 (dd, *J* = 8.0, 1.2 Hz, 1H), 7.48 (dd, *J* = 7.8, 1.5 Hz, 1H), 7.28 (td, *J* = 7.6, 1.2 Hz, 1H), 7.18 (td, *J* = 7.8, 1.8 Hz, 1H), 7.09 (d, *J* = 12.2 Hz, 1H), 6.07 (d, *J* = 12.1 Hz, 1H), 3.65 (s, 3H).

Spectral data were in agreement with those previously reported.[28] The equivalent Still-Gennari reaction gave an *Z/E* selectivity of 2.5:1[29]

Olefin 2f

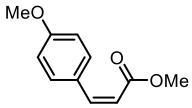

Olefin **2f** was prepared from *p*-methoxybenzaldehyde (27.2 mg, 0.200 mmol) according to general procedure, and obtained as clear oil in 49% yield (19.0 mg, 0.0988 mmol).

^{1}H NMR (400 MHz, $CDCl_3$) *δ* 7.69 (d, *J* = 8.6 Hz, 2H), 6.89–6.83 (m, 3H), 5.82 (d, *J* = 12.9 Hz, 1H), 3.82 (s, 3H), 3.72 (s, 3H).

Spectral data were in agreement with those previously reported.[27] The equivalent Still-Gennari reaction gave an *Z/E* selectivity of 8.1:1 to 20:1.[26,30]

Olefin 2g

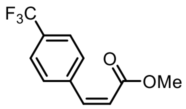

Olefin **2g** was prepared from *p*-trifluoromethylbenzaldehyde (34.8 mg, 0.200 mmol) according to general procedure, and obtained as clear oil in 61% yield (28.0 mg, 0.122 mmol).

^{1}H NMR (400 MHz, $CDCl_3$) *δ* 7.62 (m, 4H), 6.98 (d, *J* = 12.6 Hz, 1H), 6.06 (d, *J* = 12.2 Hz, 1H), 3.71 (s, 3H).

Spectra were in agreement with those previously reported.[31] The equivalent Still-Gennari reaction gave an *Z/E* selectivity of 10:1.[26]

Olefin 2h

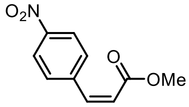

Olefin **2h** was prepared from 3-pyridinecarboxaldehyde (30.2 mg, 0.200 mmol) according to general procedure, and obtained a clear oil in 81% yield (30.5 mg, 0.162 mmol).

^{1}H NMR (400 MHz, $CDCl_3$) *δ* 8.24 (d, *J* = 8.4 Hz, 2H), 7.77 – 7.61 (m, 3H), 6.56 (d, *J* = 16.1 Hz, 1H), 3.83 (s, 3H).

Spectral data were in agreement with those previously reported.[27] The equivalent Still-Gennari reaction gave an *Z/E* selectivity of 11.5:1.[32]

Olefin *Z*-2a

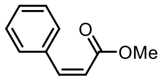

Olefin ***Z*-2a** was prepared from benzaldehyde (20 μL, 0.200 mmol) according to general procedure, and obtained as clear oil in 93% yield (30 mg, 0.185 mmol).

^{1}H NMR (400 MHz, $CDCl_3$) *δ* 7.59 (d, *J* = 7.9 Hz, 2H), 7.38–7.30 (m, 3H), 6.95 (d, *J* = 12.6 Hz, 1H), 5.95 (d, *J* = 12.7 Hz, 1H), 3.72 (s, 3H).

Spectral data were in agreement with those previously reported.[27] The equivalent Still-Gennari reaction gave an *Z/E* selectivity as low as 8:1, but in some cases >20:1.[26,30,33]

Olefin 2i

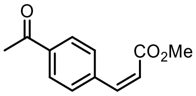

2i was prepared according to the general procedure from 4-acetylbenzaldehyde (29.6 mg, 0.200 mmol) and obtained as clear oil in 86% yield (35.0 mg, 0.172 mmol).

^{1}H NMR (600 MHz, $CDCl_3$) *δ* 7.97 – 7.88 (m, 2H), 7.62 (d, *J* = 8.2 Hz, 2H), 7.00 (d, *J* = 12.6 Hz, 1H), 6.06 (d, *J* = 12.6 Hz, 1H), 3.71 (s, 3H), 2.61 (s, 3H); **^{13}C NMR** (151 MHz, $CDCl_3$) *δ* 197.7, 166.3, 142.2, 139.6, 137.1, 129.7 (2C), 128.2 (2C), 121.6, 51.7, 26.8; **IR** (neat) *v* 2975, 1702, 1682, 1604, 1396, 1362, 1265, 1166, 853, 597 cm^{-1}; **HRMS** (ESI^+) m/z: $[M]^+$ calculated for $C_{12}H_{12}O_3Na^+$ 227.0679, found 227.0675.

Olefin 2j

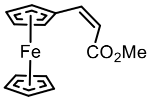

Olefin **2j** was prepared according to the general procedure from ferrocene carboxaldehyde (42.8 mg, 0.200 mmol) and obtained as clear oil in 50% yield (28 mg, 0.104 mmol) after flash column chromatography (0–30% EtOAc in heptane). ^{1}H NMR analysis of the crude reaction mixture indicated a 2.5:1 diastereomeric ratio.

^{1}H NMR (600 MHz, $CDCl_3$) δ 6.69 (d, *J* = 12.4 Hz, 1H), 5.72 (d, *J* = 12.4 Hz, 1H), 4.85 (t, *J* = 1.8 Hz, 2H), 4.39 (t, *J* = 1.8 Hz, 2H), 4.15 (s, 5H), 3.73 (s, 3H); **^{13}C NMR** (151 MHz, $CDCl_3$) δ 167.0, 144.2, 113.8, 77.6,

72.1 (2C), 70.8 (2C), 69.5 (5C), 51.1; **IR** (neat) *v* 3136, 2988, 2853, 1717, 1618, 1374, 1204, 1161, 816 cm^{-1}; **HRMS** (ESI^+) m/z: $[M – e]^+$ calcd for $[C_{14}H_{14}FeO_2]^+$ 270.0338, found 270.0332.

Olefin 2k

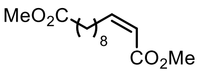

Olefin **2k** was prepared according to the general procedure from methyl 9-formylnonanoate (41 μl, 0.200 mmol) and obtained as colorless oil in 61% yield (31.2 mg, 0.122 mmol) after flash column chromatography (0–30% EtOAc in heptane).

^{1}H NMR (400 MHz, $CDCl_3$) *δ* 6.22 (dt, *J* = 11.5, 7.5 Hz, 1H), 5.76 (dt, *J* = 11.5, 1.6 Hz, 1H), 3.70 (s, 3H), 3.66 (s, 3H), 2.63 (qd, *J* = 7.5, 1.6 Hz, 2H), 2.29 (t, *J* = 7.5 Hz, 2H), 1.62 – 1.54 (m, 2H), 1.44 – 1.39 (m, 2H), 1.35 – 1.24 (m, 8H); **^{13}C NMR** (101 MHz, $CDCl_3$) *δ* 174.4, 167.0, 151.1, 119.3, 51.6, 51.1, 34.2, 29.3 (2C), 29.3, 29.2, 29.1 (2C), 25.1; **HRMS** (ESI) m/z $[M+ Na]^+$ calcd for $C_{14}H_{24}O_4Na^+$ 279.1567; found 279.1568; **IR** (neat) *v* 2926, 2855, 1739, 1124, 1645, 1437, 1408, 1364, 1195, 1172, 1015, 820 cm^{-1}.

Olefin 2l

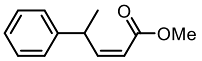

Olefin **2l** was prepared according to the general procedure from 2-phenylpropionaldehyde (27 μL, 0.200 mmol) according to general procedure, and obtained as a clear oil in 76% yield (29.0 mg, 0.152 mmol).

^{1}H NMR (400 MHz, $CDCl_3$): *δ* 7.33–7.28 (m, 4H), 7.23–7.19 (m, 1H), 6.28 (dd, *J* = 11.4, 1.0 Hz, 1H), 5.75 (dd, *J* = 11.4, 1.0 Hz, 1H), 4.94–4.86 (m, 1H), 3.74 (s, 3H), 1.40 (d, *J* = 6.9 Hz, 3H); **^{13}C NMR** (101 MHz, $CDCl_3$) *δ* 166.6, 154.0, 144.5, 128.6 (2C), 127.0 (2C), 126.4, 117.3, 51.1, 37.7, 20.8; **IR** (neat) *v* 2950, 2927, 2841, 1713, 1650, 1410, 1186, 1166, 1012 cm^{-1}; **HRMS** (ESI) m/z: $[M+Na]^+$ calcd for $C_{12}H_{14}NaO_2$ 213.0891; found 213.0889.

Olefin 2m

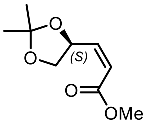

Olefin **2m** was prepared according to the general procedure from (R)-(+)-2,2-dimethyl-1,3-dioxolane-4-carboxaldehyde (26.0 mg, 0.200 mmol) and obtained as clear oil in 51% yield (19.0 mg, 0.102 mmol) after flash column chromatography (5–50% EtOAc in heptane). ^{1}H NMR analysis of the crude reaction mixture indicated a 4:1 Z/E ratio.

^{1}H NMR (400 MHz, $CDCl_3$) δ 6.37 (dd, *J* = 11.6, 6.7 Hz, 1H), 5.86 (d, *J* = 11.6 Hz, 1H), 5.50 (q, *J* = 6.7 Hz, 1H), 4.38 (t, *J* = 7.6 Hz, 1H), 3.72 (s, 3H), 3.62 (t, *J* = 7.5 Hz, 1H), 1.45 (s, 3H), 1.39 (s, 3H); **$[\alpha]_D^{20}$** = +98 (*c* = 0.1, $CHCl_3$).

Spectra data were in agreement with those previously reported.[34]

Olefin 2n

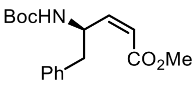

Olefin **2n** was prepared according to the general procedure from (*R*)-(+)-Boc-3-phenylpropanal (49.9 mg, 0.200 mmol) and obtained as white solid in 48% yield (29.3 mg, 0.096 mmol, 99% *ee*) after flash column chromatography (10–50% EtOAc in heptane). ^{1}H NMR analysis of the crude reaction mixture indicated a 5:1 Z/E ratio, and the enantiomeric excess of the purified product was determined to be 99%.

^{1}H NMR (400 MHz, $CDCl_3$) δ 7.35 – 7.29 (m, 2H), 7.29 – 7.19 (m, 3H), 6.21 (br s, 1H), 5.84 (dd, J = 11.6, 0.7 Hz, 1H), 5.35 (br s, 1H), 4.77 (br s, 1H), 3.74 (s, 3H), 3.08 – 2.95 (m, 1H), 2.90 (br s, 1H), 1.39 (s, 9H); **^{13}C NMR** (101MHz, $CDCl_3$) δ 166.3, 155.4, 150.6, 137.3, 129.7 (2C), 128.6 (2C), 126.8, 119.5, 79.7, 51.5, 50.7, 40.4, 28.4 (3C); **HRMS** (ESI) m/z: $[M+Na]^+$ calcd for $C_{17}H_{23}O_4NNa^+$ 328.1519; found 328.1508; **IR** (neat) v 3360, 2981, 2925, 2853, 1717, 1686, 1640, 1526, 1439, 1251, 1230, 1199, 1171, 703 cm^{-1}.

The equivalent Still-Gennari reaction was performed according to the procedure of List *et al.* and delivered the alkene in >20:1 selectivity.[35]

Determination of enantiopurity. Method description: Lux-3 Cellulose-3 250 x 4.6 mm, particle size 3 µm, solvent system: n-heptane/iPrOH 85:15; flow 0.7 mL/min, 25 °C. Peak area 0.675% (R_t = 6.787 min), 99.325% (R_t = 7.634 min).

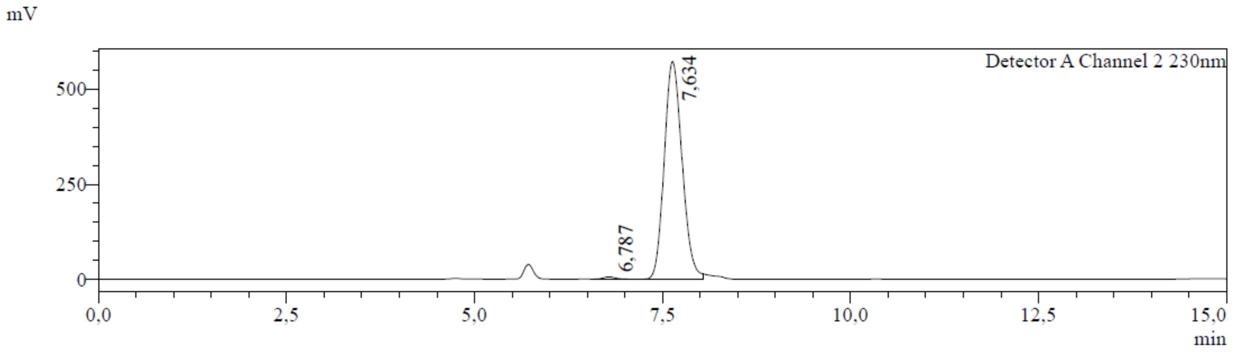

Scalemic standard, prepared accordingly to the general procedure.

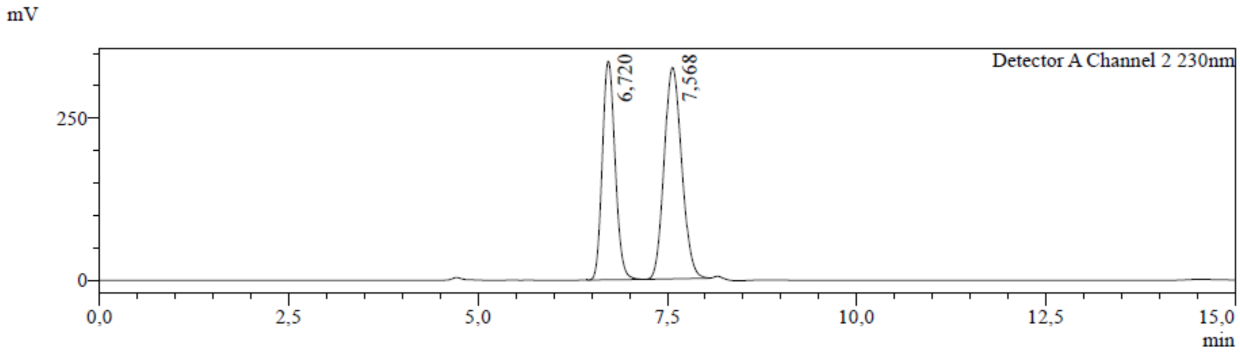

Olefin 2o

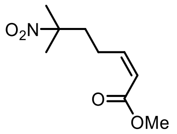

Olefin **2o** was prepared according to the general procedure from 4-methyl-4-nitrovaleraldehyde (29 mg, 0.200 mmol) and obtained as clear oil in 54% yield (21.6 mg, 93% purity, 0.102 mmol) after flash column chromatography (5–50% EtOAc in heptane). ^{1}H NMR analysis of the crude reaction mixture indicated a 7:1 Z/E ratio.

^{1}H NMR (600 MHz, $CDCl_3$) δ 6.25 – 6.10 (m, 1H), 5.81 (d, *J* = 11.4 Hz, 1H), 3.71 (s, 3H), 2.69 – 2.60 (m, 2H), 2.05 (dd, *J* = 9.9, 6.7 Hz, 2H), 1.62 (s, 6H); **^{13}C NMR** (151 MHz, $CDCl_3$) δ 166.6, 147.8, 120.7, 88.0, 51.3, 39.7, 25.9 (2C), 24.1; **HRMS** (ESI$^+$) m/z: [M + Na]$^+$ calcd 224.0893 for $C_9H_{15}O_4NNa^+$, 224.0893 found; **IR** (film) *v* 2990, 2951, 1720, 1648, 1537, 1197, 1100, 734 cm^{-1}.

Olefin 2p

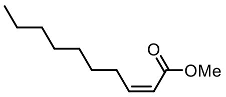

Olefin **2p** was prepared from octanal (31 μL, 0.200 mmol) according to general procedure, and obtained as a clear oil in 49% yield (18 mg, 0.0977 mmol).

^{1}H NMR (400 MHz, $CDCl_3$) *δ* 6.21 (dt, *J* = 11.4, 7.5 Hz, 1H), 5.75 (dt, *J* = 11.5, 1.7 Hz, 1H), 3.69 (s, 3H), 2.63 (qd, *J* = 7.3, 1.7 Hz, 2H), 1.46–1.39 (m, 2H), 1.35–1.26 (m, 8H), 0.86 (t, *J* = 7.0 Hz, 3H); **^{13}C NMR** (101 MHz, $CDCl_3$) *δ* 167.1, 151.2, 119.3, 51.1, 31.9, 29.4, 29.24 (2C), 29.17, 22.8, 14.2; **IR** (neat) *v* 2922, 2851, 1632, 1275, 1261 cm^{-1}; **HRMS** (ESI$^+$) m/z: [M+H]$^+$ calcd for $C_{11}H_{21}O_2$ 185.1542; found 185.1538.

The equivalent Still-Gennari reaction gave an *Z/E* selectivity of 11.5:1[30]

Olefin 2q

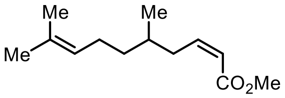

Olefin **2q** was prepared according to the general procedure and obtained as yellow oil in 59% yield (25 mg, 0.12 mmol) after flash column chromatography (0–30% EtOAc in heptane). ^{1}H NMR analysis of the crude reaction mixture indicated a 15:1 Z/E ratio.

^{1}H NMR (400 MHz, $CDCl_3$) *δ* 6.24 (dt, *J* = 11.6, 7.5 Hz, 1H), 5.81 (dt, *J* = 11.6, 1.6 Hz, 1H), 5.08 (t, *J* = 7.1 Hz, 1H), 3.70 (s, 3H), 2.71 – 2.47 (m, 2H), 2.10 – 1.87 (m, 2H), 1.67 (s, 3H), 1.59 (s, 3H), 1.41 – 1.31 (m, 1H), 1.28 – 1.11 (m, 2H), 0.91 (d, *J* = 6.7 Hz, 3H); **^{13}C NMR** (101MHz, $CDCl_3$) *δ* 167.1, 150.0, 131.4, 124.8, 120.0, 51.1, 36.9, 36.1, 33.0, 25.9, 25.7, 19.6, 17.8; **HRMS** (ESI$^+$) m/z: [M+Na]$^+$ calcd for $C_{13}H_{22}O_2Na^+$ 233.1512; found 233.1513; **IR** (neat) *v* 2956, 2924, 2854, 1727, 1645, 1438, 1408, 1378, 1194, 1174, 817 cm^{-1}

Olefin 2r

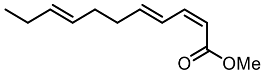

Olefin **2r** was prepared according to the general procedure from *trans*-2-*trans*-6-nonadienal (33 μl, 0.200 mmol) and obtained as clear oil in 75% yield (29.2 mg, 0.152 mmol) after flash column chromatography (5–30% EtOAc in heptane). ^{1}H-NMR analysis of the crude reaction mixture indicated a 4:1 diastereomeric ratio.

^{1}H NMR (400 MHz, $CDCl_3$) δ 7.36 (dd, *J* = 15.3, 11.4 Hz, 1H), 6.55 (t, *J* = 11.4 Hz, 1H), 6.14* (d, *J* = 6.0 Hz, 0.2H), 6.06 (dd, *J* = 14.9, 7.3 Hz, 1H), 5.79* (d, *J* = 15.3 Hz, 0.2H), 5.57 (d, *J* = 11.4 Hz, 1H), 5.52 – 5.43 (m, 1.2H), 5.42 – 5.32 (m, 1.2H), 3.72 (m, 3.6H), 2.30 – 2.23 (m, 2.5H), 2.13 (d, *J* = 7.1 Hz, 2.5H), 1.99 (p, *J* = 7.2 Hz, 2.7H), 0.96 (t, *J* = 7.5 Hz, 3.8H); **^{13}C NMR** (101 MHz, $CDCl_3$) δ 167.9*, 167.1, 145.6, 145.42*, 145.3, 144.3*, 133.3*, 133.1, 128.7*, 128.0, 127.8*, 127.2, 119.0*, 115.3, 51.6*, 51.2, 33.19, 33.16*, 31.9, 31.8*, 25.7†, 14.0†; **HRMS** (ESI$^+$) m/z: $[M+Na]^+$ calcd 217.1199 for $C_{12}H_{18}O_2Na^+$, 217.1199 found; **IR** (film) *v* 2961, 1717, 1639, 1602, 1437, 1268, 1194, 1139, 1000, 964 cm^{-1}.

Olefin 2s

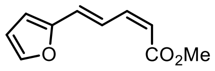

Olefin **2s** was prepared according to the general procedure from 3-(2-furyl)acrolein (24.4 mg, 0.200 mmol) and obtained as white solid in 62% yield (22 mg, 0.123 mmol) after flash column chromatography (0–30% EtOAc in heptane).

^{1}H NMR (400 MHz, $CDCl_3$) *δ* 7.96 (dd, *J* = 15.6, 11.8 Hz, 1H), 7.44 (d, *J* = 1.4 Hz, 1H), 6.66 (appt, *J* = 11.5 Hz, 1H), 6.60 (d, *J* = 15.6 Hz, 1H), 6.48 (d, *J* = 3.4 Hz, 1H), 6.42 (dd, *J* = 3.4, 1.8 Hz, 1H), 5.70 (d, *J* = 11.2 Hz, 1H), 3.76 (s, 3H); **^{13}C NMR** (101 MHz, $CDCl_3$) *δ* 167.1, 152.6, 144.4, 143.9, 128.0, 123.5, 117.0, 112.2, 111.8, 51.3; **HRMS** (ESI$^+$) m/z $[M+Na]^+$ calcd. for $C_{10}H_{10}O_3Na^+$ 201.0522; found 201.0524; **IR** (neat) *v* 2949, 2854, 1707, 1610, 1435, 1263, 1240, 1202, 1168, 1134, 1014, 995, 955, 737 cm^{-1}.

Olefin 2t

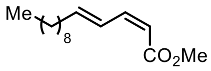

Olefin **2t** was prepared according to the general procedure from *trans*-2-dodecenal (48 μl, 0.200 mmol) and obtained as yellow oil in 84% yield (39.8 mg, 0.167 mmol) after flash column chromatography (0–30% EtOAc in heptane). ^{1}H-NMR analysis of the crude reaction mixture indicated a 10:1 Z/E ratio.

^{1}H NMR (400 MHz, $CDCl_3$) *δ* 7.40 - 7.30 (m, 1H), 6.55 (appt, *J* = 11.3 Hz, 1H), 6.11 – 6.02 (m, 1H), 5.56 (d, *J* = 11.3 Hz, 1H), 3.71 (s, 3H), 2.25 - 2.14 (m, 2H), 1.46 - 1.37 (m, 2H), 1.33 – 1.22 (m, 12H), 0.87 (t, *J* = 6.8 Hz, 3H); **^{13}C NMR** (101 MHz, $CDCl_3$) *δ* 167.1, 146.2, 145.8, 127.0, 115.1, 51.2, 33.2, 32.0, 29.7, 29.6, 29.4, 29.4, 28.9, 22.8, 14.2; **HRMS** (ESI$^+$) m/z $[M+Na]^+$ calcd. for $C_{15}H_{26}O_2Na^+$ 261.1825; found 261.1828; **IR** (neat) *v* 2924, 2853, 1719, 1639, 1601, 1459, 1437, 1412, 1193, 1171, 1140, 999, 962, 818 cm^{-1}.

Olefin 2u

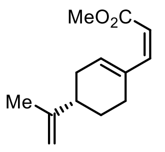

Olefin **2u** was prepared according to the general procedure from (*S*)-(−)-perillaldehyde (34 µl, 0.200 mmol) and obtained as light-yellow oil in 64% yield (26.2 mg, 0.127 mmol) after flash column chromatography (0–30% EtOAc in heptane). ^{1}H NMR analysis of the crude reaction mixture indicated an >20:1 Z/E ratio, which decreased to 5:1 after column chromatography.

^{1}H NMR (400 MHz, $CDCl_3$) δ 6.35 (d, *J* = 12.7 Hz, 1H), 6.04 (br s, 1H), 5.60 (d, *J* = 12.7 Hz, 1H), 4.73 (s, 1H), 4.71 (s, 1H), 3.71 (s, 3H), 2.51 – 2.37 (m, 1H), 2.34 – 2.04 (m, 4H), 1.90 – 1.80 (m, 1H), 1.74 (s, 3H), 1.51 – 1.39 (m, 1H); **^{13}C NMR** (101 MHz, $CDCl_3$) δ 167.4, 149.5, 144.5, 135.4, 135.1, 116.0, 109.0, 51.5, 40.5, 31.8, 27.7, 27.4, 20.9; **HRMS** (ESI$^+$) m/z [M+Na]$^+$ calcd. for $C_{13}H_{18}O_2Na^+$ 229.1199; found 229.1199; **IR** (neat) *v* 2924, 2854, 1728, 1624, 1438, 1376, 1192, 1168, 1000, 889, 840, 816 cm^{-1}.

Olefin 2v

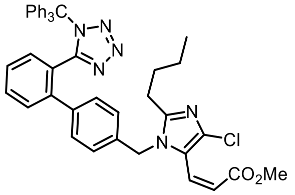

Olefin **2v** was prepared according to the general procedure from **S5** and obtained as yellow oil in 62% yield (89.0 mg, 0.124 mmol) after flash column chromatography (0–15% EtOAc in heptane). ^{1}H NMR analysis of the crude reaction mixture indicated a >20:1 diastereomeric ratio.

^{1}H NMR (600 MHz, $CDCl_3$) δ 7.95 (dd, *J* = 7.5, 1.2 Hz, 1H), 7.51 – 7.45 (m, 2H), 7.37 – 7.31 (m, 4H), 7.27 – 7.23 (m, 6H), 7.11 (d, *J* = 8.2 Hz, 2H), 6.93 – 6.91 (m, 6H), 6.74 (d, *J* = 8.2 Hz, 2H), 6.27 (d, *J* = 11.9 Hz, 1H), 5.84 (d, *J* = 11.9 Hz, 1H), 4.92 (s, 2H), 3.75 (s, 3H), 2.51 – 2.45 (m, 2H), 1.68 – 1.61 (m, 2H), 1.32 – 1.25 (m, 2H), 0.85 (t, *J* = 7.4 Hz, 3H); **^{13}C NMR** (151 MHz, $CDCl_3$) δ 166.2, 164.1, 149.2, 141.44 (3C), 141.40, 141.3, 134.2, 130.9, 130.4, 130.4 (6C), 130.13, 130.10 (2C), 129.9, 128.5 (3C), 127.9, 127.8 (6C), 126.8, 126.4, 125.3 (2C), 123.4, 122.4, 83.0, 52.0, 47.8, 29.7, 27.0, 22.5, 13.9; **HRMS** (ESI$^+$) m/z [M]$^+$ calcd 719.2896 for $C_{44}H_{39}N_6O_2(^{35}Cl)H^+$, found 719.2853; **IR** (neat) *v* 2926, 1711, 1444, 1358, 1254, 1168, 1028, 1003, 824, 747, 698 cm^{-1}.

Olefin 2w

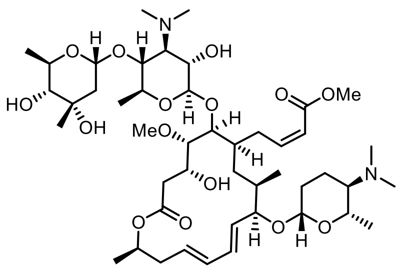

The general procedure was modified for the preparation of olefin **2w** which was unsuitable for purification by flash column chromatography.

A flame-dried Schlenk tube equipped with a stir bar was charged with spiramycin (0.10 mmol, 1.0 eq) and isothiouronium salt (62.2 mg, 0.11 mmol, 1.10 eq) under argon. Tetrahydrofuran (0.5 mL, 0.2 M) was added. The resulting mixture was stirred at -78°C for 10 minutes. Barton's base (0.12 mmol, 24.2 µL) was then added and the resulting mixture was stirred at -78°C for 2 hours. The reaction mixture was allowed to room temperature, then it was concentrated *in vacuo*. Analysis of this crude mixture by ^{1}H NMR with the internal standard ethyl *p*-bromobenzoate revealed alkene **2w** was

delivered in 70% yield in 6:1 Z/E ratio (61% w.r.t *Z*-**2w**). An analytical sample *Z*-**2w** was purified by RP-LC (Rt = 12.96 min; Waters AutoPurification Core System with Qda; XSelect CSH PREP C18 5 μm OBD 30 x 150 mm; gradient elution with 10mM NH_4HCO_3 and MeCN; 50%–95% MeCN in 15 min; flow 20 mL/min; separation monitoring MS and UV 220 and 254 nm).

^{1}H NMR (600 MHz, $CDCl_3$) *δ* 6.50 – 6.39 (m, 1H), 6.22 (dd, *J* = 15.0, 10.7 Hz, 1H), 6.00 (dd, *J* = 15.0, 10.8 Hz, 1H), 5.85 (d, *J* = 11.3 Hz, 1H), 5.70 (dd, *J* = 15.0, 9.6 Hz, 1H), 5.52 (ddd, *J* = 15.0, 11.3, 3.9 Hz, 1H), 5.27 – 5.19 (m), 5.06 (d, *J* = 3.3 Hz, 1H), 4.47 (d, *J* = 9.2 Hz, 1H), 4.36 (d, *J* = 7.4 Hz, 1H), 4.18 (dd, *J* = 9.4, 3.9 Hz, 1H), 4.14 – 4.03 (m, 2H), 3.89 (s, 1H), 3.73 (d, *J* = 12.9 Hz, 1H), 3.70 (s, 3H), 3.57 (dd, *J* = 10.3, 7.6 Hz, 1H), 3.51 (s, 3H), 3.44 (dq, *J* = 12.4, 6.2 Hz, 1H), 3.27 – 3.19 (m, 2H), 3.07 (d, *J* = 8.7 Hz, 1H), 2.95 – 2.89 (m, 2H), 2.77 – 2.68 (m, 1H), 2.65 (dd, *J* = 14.6, 11.2 Hz, 1H), 2.48 – 2.35 (m, 8H), 2.23 – 2.11 (m, 8H), 2.09 (dd, *J* = 24.9, 11.4 Hz, 1H), 2.01 (d, *J* = 14.2 Hz, 1H), 1.89 – 1.80 (m, 2H), 1.74 (dd, *J* = 14.4, 3.7 Hz, 1H), 1.57 (td, *J* = 13.8, 3.7 Hz, 1H), 1.51 – 1.43 (m, 2H), 1.28 (t, *J* = 6.3 Hz, 6H), 1.24 – 1.20 (m, 6H), 1.19 (d, *J* = 6.1 Hz, 3H), 0.98 (d, *J* = 6.7 Hz, 3H); **^{13}C NMR** (151 MHz, $CDCl_3$) *δ* 174.6, 166.8, 150.6, 135.0, 133.2, 130.8, 128.8, 120.0, 104.9, 100.4, 96.4, 85.6, 79.9, 78.8, 76.6, 74.7, 73.8 (2C), 73.5, 72.4, 69.5, 69.4, 68.8, 68.3, 66.1, 65.0, 62.0, 51.0, 42.2, 41.0, 40.8 (4C), 37.8, 31.5, 31.3, 29.8, 28.0, 25.5, 20.2, 19.2, 19.0, 18.6, 18.4, 15.3; **HRMS** (ESI$^+$) $[M+H]^+$ calcd 899.5476 for $C_{46}H_{79}O_{15}N_2$, found 899.5476; **$[\alpha]_D^{20}$** = -67.4 (c = 1, $CHCl_3$); **IR** (film) *v* 3474, 2931, 2785, 1719, 1439, 1163, 906, 753, 460 cm^{-1}.

The equivalent Still-Gennari reaction was performed according to the procedure of List *et al.* and delivered the alkene in >1.7:1 selectivity.[35]

*LC-RP trace for the purification of olefin **2w** (Rt = 12.96 min)*

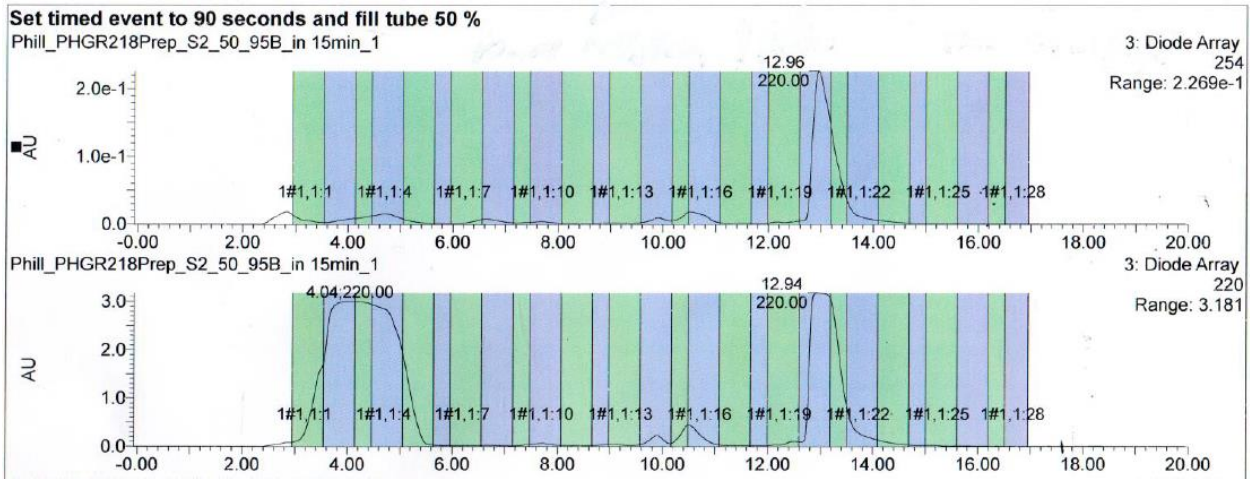

Olefin 2b

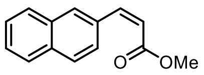

Olefin **2b** was prepared according to the general procedure (*c* = 0.3 M) from 2-napthaladehyde (93.7 mg, 0.200 mmol) and obtained as clear oil in 92% yield (42.4 mg, 0.170 mmol) after flash column chromatography (65% toluene in heptane).

^{1}H NMR (400 MHz, $CDCl_3$) δ 8.06 (s, 1H), 7.88 – 7.69 (m, 4H), 7.56 – 7.41 (m, 2H), 7.12 (d, *J* = 12.6 Hz, 1H), 6.05 (d, *J* = 12.6 Hz, 1H), 3.75 (s, 3H).

Spectral data were in agreement with those previously reported.[36]

Gram-scale experiment. According to the otherwise unaltered general procedure, 2-napthaldehyde (1.17 g, 7.52 mmol, 1.00 eq) was reacted with thiouronium **S37** (4.80 g, 8.27 mmol, 1.10 eq) and Barton's base (1.82 mL, 9.02 mmol, 1.2 eq) to afford olefin **2b** as a clear oil in 88% yield (1.50 g, 7.52 mmol).

Robustness experiment. The general procedure was altered to be conducted at room temperature, using 'wet' THF, without dried glassware, and without an inert atmosphere. Under these conditions, olefin **2b** was formed in 51% yield and 9:1 Z/E ratio as indicated by ^{1}H NMR analysis. Based on the presence of starting material 2-napthaldehyde in the crude mixture, the conversion of the reaction was judged to be 71%.

Olefin 2x

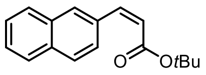

Olefin **2x** was prepared according to the general procedure from 2-napthaladehyde (93.7 mg, 0.200 mmol) and obtained as clear oil in 83% yield (42.0 mg, 0.165 mmol) after flash column chromatography (65% toluene in heptane).

^{1}H NMR (400 MHz, $CDCl_3$) δ 7.96 (s, 1H), 7.86 – 7.76 (m, 3H), 7.68 (dd, *J* = 8.5, 1.6 Hz, 1H), 7.54 – 7.42 (m, 2H), 7.02 (d, *J* = 12.5 Hz, 1H), 5.97 (d, *J* = 12.5 Hz, 1H), 1.45 (s, 9H); **^{13}C NMR** (101 MHz, $CDCl_3$) *δ* 166.0, 141.1, 133.4, 133.1, 133.0, 129.4, 128.5, 127.8, 127.5, 127.1, 126.7, 126.3, 122.5, 81.0, 28.2 (3C); **HRMS** (ESI^+) m/z: $[M + Na]^+$ calculated 277.1199 for $C_{17}H_{18}O_2Na^+$, found 277.1209; **IR** (film) *v* 3037, 2927, 1713, 1626, 1495, 1163, 817, 736, 478 cm^{-1}.

Olefin 2y

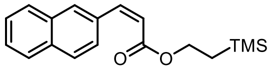

Olefin **2y** was prepared according to the general procedure from 2-napthaladehyde (93.7 mg, 0.200 mmol) and obtained as clear oil in 84% yield (50.0 mg, 0.168 mmol) after flash column chromatography (65% toluene in heptane).

^{1}H NMR (400 MHz, $CDCl_3$) δ 8.06 (s, 1H), 7.87 – 7.73 (m, 4H), 7.53 – 7.45 (m, 2H), 7.09 (d, *J* = 12.6 Hz, 1H), 6.03 (d, *J* = 12.6 Hz, 1H), 4.31 – 4.22 (m, 2H), 1.05 – 0.95 (m, 2H), 0.04 (s, 9H); **^{13}C NMR** (151 MHz, $CDCl_3$) δ 166.6, 142.9, 133.6, 133.1, 132.6, 130.0, 128.6, 127.7, 127.5, 127.2, 126.9, 126.3, 120.4, 62.7, 17.4, -1.4 (3C); **HRMS** (ESI^+) m/z $[M + Na]^+$ calcd 321.1281 for $C_{18}H_{22}O_2SiNa^+$, found 321.1293; **IR** (film) *v* 2952, 2896, 1712, 1505, 1154, 832, 748 cm^{-1}.

Olefin 2z

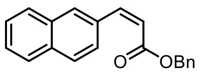

Olefin **2z** was prepared according to the general procedure from 2-napthaladehyde (93.7 mg, 0.200 mmol) and obtained as clear oil in 85% yield (49.0 mg, 0.170 mmol) after flash column chromatography (65% toluene in heptane).

^{1}H NMR (600 MHz, $CDCl_3$) δ 8.02 (s, 1H), 7.81 (appt, *J* = 8.6 Hz, 2H), 7.77 (d, *J* = 8.6 Hz, 1H), 7.70 (dd, *J* = 8.6, 1.3 Hz, 1H), 7.52 – 7.45 (m, 2H), 7.30 (brs, 5H), 7.14 (d, *J* = 12.6 Hz, 1H), 6.08 (d, *J* = 12.6 Hz, 1H), 5.18 (s, 2H); **^{13}C NMR** (151 MHz, $CDCl_3$) δ 166.2, 143.8, 135.9, 133.6, 133.1, 132.6, 130.0, 128.7, 128.6 (2C), 128.5 (2C), 128.3, 127.74, 127.65, 127.1, 127.0, 126.4, 119.9, 66.4; **HRMS** (ESI$^+$) m/z $[M+Na]^+$, calcd 311.1043 for $C_{20}H_{16}O_2Na^+$, found 311.1054; **IR** (film) *v* 2924, 2853, 1714, 1626, 1419, 1211, 1187, 906, 737, 480 cm^{-1}.

6. *E*-selective olefination of *N*-tosylimines

6.1 General Procedure

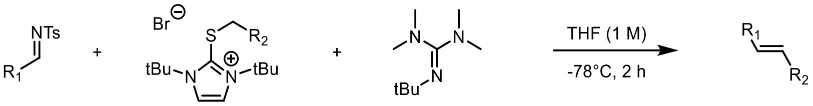

The respective aldimine (0.20 mmol, 1.0 eq) and the sulfonium salt (0.22 mmol, 1.1 eq) were dissolved in dry $CHCl_3$ (2 mL, 0.1 M) and cooled to –78 °C. Barton's base (0.24 mmol, 1.2 eq) was added dropwise and the reaction mixture was stirred at –78 °C for 2 h, then was allowed to reach rt over the course of 12 h. A solution of sat. aq. NH_4Cl was added and the aq. phase was extracted with DICHLOROMETHANE (3 × 20 ml). The combined organic layers were dried over magnesium sulfate and the solvent removed under reduced pressure. The crude residue* was purified by column chromatography.

*In all cases, ^{1}H NMR analysis of the crude reaction mixture indicated a >20:1 E/Z ratio.

6.2 Characterisation

Olefin 6a

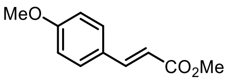

Olefin **6a** was prepared according to the general procedure from **S6** and **1** and obtained as a white solid in 77% yield (29.5 mg, 0.15 mmol) after flash column chromatography (0–5% EtOAc in heptane).

^{1}H NMR (400 MHz, $CDCl_3$) δ 7.65 (d, *J* = 16.0 Hz, 1H), 7.47 (d, *J* = 8.7 Hz, 2H), 6.90 (d, *J* = 8.7 Hz, 2H), 6.30 (d, *J* = 16.0 Hz, 1H), 3.83 (s, 3H), 3.79 (s, 3H).

Spectral data were in agreement with those previously reported.[37]

Olefin 6b

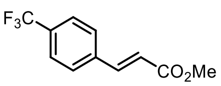

Olefin **6b** was prepared according to the general procedure from **S10** and **1** obtained a white solid in 56% yield (25.8 mg, 0.11 mmol) after flash column chromatography (3% EtOAc in heptane).

^{1}H NMR (400 MHz, $CDCl_3$) δ 7.70 (d, *J* = 16.1 Hz, 1H), 7.67 – 7.59 (m, 4H), 6.51 (d, *J* = 16.1 Hz, 1H), 3.82 (s, 3H).

Spectral data were in agreement with those previously reported.[38]

Olefin 2a

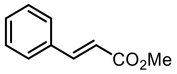

Olefin **2a** was prepared according to the general procedure from **10** and **1** and obtained a white solid in 68% yield (22.1 mg, 0.14 mmol) after flash column chromatography (5% EtOAc in heptane).

^{1}H NMR (400 MHz, $CDCl_3$) δ 7.70 (d, *J* = 16.0 Hz, 1H), 7.59 – 7.49 (m, 2H), 7.45 – 7.33 (m, 3H), 6.44 (d, *J* = 16.0 Hz, 1H), 3.81 (s, 3H).

Spectral data were in agreement with those previously reported.[37]

Olefin 6c

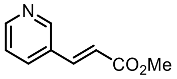

Olefin **6c** was prepared according to the general procedure from **S11** and **1** and obtained as a white solid in 73% yield (23.8 mg, 0.15 mmol) after flash column chromatography on basic aluminium oxide (10–25% EtOAc in heptane).

^{1}H NMR (400 MHz, $CDCl_3$) *δ* 8.65 (d, *J* = 5.8 Hz, 2H), 7.59 (d, *J* = 16.1 Hz, 1H), 7.35 (dd, *J* = 4.6, 1.5 Hz, 2H), 6.58 (d, *J* = 16.1 Hz, 1H), 3.82 (s, 3H).

Spectral data were in agreement with those previously reported.[38]

Olefin 6d

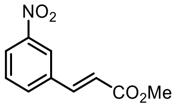

Olefin **6d** was prepared according to the general procedure from **S12** and **1** and obtained a white solid in 58% yield (24.0 mg, 0.12 mmol) after flash column chromatography (3% EtOAc in heptane).

^{1}H NMR (400 MHz, $CDCl_3$) δ 8.37 (app s, 1H), 8.26 – 8.18 (m, 1H), 7.82 (d, *J* = 7.7 Hz, 1H), 7.72 (d, *J* = 16.0 Hz, 1H), 7.58 (t, *J* = 8.0 Hz, 1H), 6.56 (d, *J* = 16.0 Hz, 1H), 3.84 (s, 3H).

Spectral data were in agreement with those previously reported.[39]

Olefin 6e

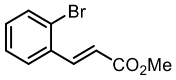

Olefin **6e** was prepared according to the general procedure from **S9** and **1** and obtained a colourless oil in 49% yield (23.4 mg, 0.1 mmol) after flash column chromatography (3% EtOAc in heptane).

^{1}H NMR (400 MHz, $CDCl_3$) δ 8.05 (d, *J* = 16.0 Hz, 1H), 7.61 (m, 2H), 7.36 – 7.29 (m, 1H), 7.21 (dd, *J* = 7.6, 1.7 Hz, 1H), 6.39 (d, *J* = 16.0 Hz, 1H), 3.83 (s, 3H).

Spectral data were in agreement with those previously reported.[40]

Olefin 6f

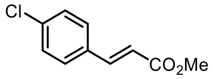

Olefin **6f** was prepared according to the general procedure from **S8** and **1** and obtained as a white solid in 86% yield (33.8 mg, 0.17 mmol) after flash column chromatography (10% EtOAc in heptane).

^{1}H NMR (400 MHz, $CDCl_3$) *δ* 7.63 (d, *J* = 16.0 Hz, 1H), 7.48 – 7.41 (m, 2H), 7.41 – 7.29 (m, 2H), 6.40 (d, *J* = 16.0 Hz, 1H), 3.80 (s, 3H).

Spectral data were in agreement with those previously reported.[37]

Olefin 6g

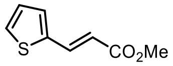

Olefin **6g** was prepared according to the general procedure from **S7** and **1** and obtained as a pale yellow solid in 73% yield (24.4 mg, 0.15 mmol, *E/Z* 25:1) after flash column chromatography (10% EtOAc in heptane).

^{1}H NMR (400 MHz, $CDCl_3$) δ 7.78 (d, *J* = 15.7 Hz, 1H), 7.37 (d, *J* = 5.1 Hz, 1H), 7.25 (d, *J* = 3.6 Hz, 1H), 7.04 (dd, *J* = 5.1, 3.6 Hz, 1H), 6.24 (d, *J* = 15.7 Hz, 1H), 3.78 (s, 3H).

Spectral data were in agreement with those previously reported.[41]

Olefin 6h

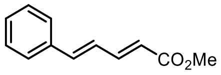

Olefin **6h** was prepared according to the general procedure from **S13** and **1** and obtained a white solid in 80% yield (30.0 mg, 0.16 mmol) after flash column chromatography (5% EtOAc in heptane).

^{1}H NMR (400 MHz, $CDCl_3$) δ 7.51 – 7.41 (m, 3H), 7.39 – 7.28 (m, 3H), 6.91 – 6.84 (m, 2H), 6.00 (d, *J* = 15.2 Hz, 1H), 3.77 (s, 3H).

Spectral data were in agreement with those previously reported.[38]

Olefin 6i

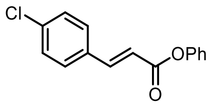

Olefin **6i** was prepared according to the general procedure from **S31** and **S8** and obtained a white solid in 52% yield (26.9 mg, 0.10 mmol) after flash column chromatography (2% EtOAc in heptane).

^{1}H NMR (400 MHz, $CDCl_3$) δ 7.82 (d, *J* = 16.0 Hz, 1H), 7.56 – 7.49 (m, 2H), 7.45 – 7.37 (m, 4H), 7.30 – 7.23 (m, 1H), 7.17 (dd, *J* = 8.6, 1.1 Hz, 2H), 6.61 (d, *J* = 16.0 Hz, 1H).

Spectral data were in agreement with those previously reported.[42]

Olefin 6j

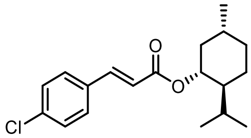

Olefin **6j** was prepared according to the general procedure from **S32** and **S8** and obtained a pale yellow oil in 70% yield (44.6 mg, 0.14 mmol) after flash column chromatography (1% EtOAc in heptane).

^{1}H NMR (400 MHz, $CDCl_3$) δ 7.61 (d, *J* = 16.0 Hz, 1H), 7.48 – 7.42 (m, 2H), 7.38 – 7.32 (m, 2H), 6.40 (d, *J* = 16.0 Hz, 1H), 4.86 – 4.78 (m, 1H), 2.10 – 2.00 (m, 1H), 1.94 – 1.88 (m, 1H), 1.75 – 1.66 (m, 2H), 1.56 – 1.49 (m, 1H), 1.47 – 1.42 (m, 1H), 1.11 – 1.02 (m, 2H), 0.96 – 0.88 (m, 7H), 0.79 (d, *J* = 7.0 Hz, 3H).

Spectral data were in agreement with those previously reported.[43]

Olefin 6k

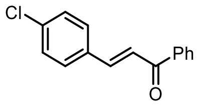

Olefin **6k** was prepared according to the general procedure from **S29** and **S8** and obtained a white solid in 61% yield (29.8 mg, 0.12 mmol) after flash column chromatography (2% EtOAc in heptane).

^{1}H NMR (400 MHz, $CDCl_3$) δ 8.02 (dd, *J* = 8.3, 1.3 Hz, 2H), 7.76 (d, *J* = 15.7 Hz, 1H), 7.63 – 7.54 (m, 3H), 7.54 – 7.47 (m, 3H), 7.43 – 7.37 (m, 2H).

Spectral data were in agreement with those previously reported.[38]

Olefin 6l

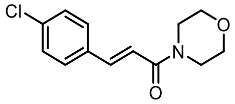

Olefin **6l** was prepared according to the general procedure from **S34** and **S8** and obtained a white solid in 73% yield (36.7 mg, 0.15 mmol) after flash column chromatography (15–50% EtOAc in heptane).

^{1}H NMR (400 MHz, $CDCl_3$) δ 7.63 (d, *J* = 15.4 Hz, 1H), 7.48 – 7.40 (m, 2H), 7.37 – 7.31 (m, 2H), 6.80 (d, *J* = 15.4 Hz, 1H), 3.72 (m, 8H).

Spectral data were in agreement with those previously reported.[44]

Olefin 6m

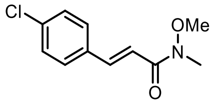

Olefin **6m** was prepared according to the general procedure from **S33** and **S8** and obtained a colourless oil in 75% yield (33.9 mg, 0.15 mmol) after flash column chromatography (2–20% EtOAc in heptane).

^{1}H NMR (400 MHz, $CDCl_3$) δ 7.67 (d, *J* = 15.8 Hz, 1H), 7.51 – 7.45 (m, 2H), 7.38 – 7.30 (m, 2H), 6.99 (d, *J* = 15.8 Hz, 1H), 3.75 (s, 3H), 3.30 (s, 3H).

Spectral data were in agreement with those previously reported.[45]

Olefin 6n

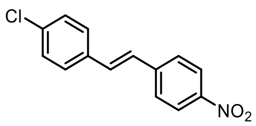

Olefin **6n** was prepared according to the general procedure from **S36** and **S8** obtained a yellow solid in 58% yield (30.0 mg, 0.12 mmol) after flash column chromatography (2% EtOAc in heptane).

^{1}H NMR (400 MHz, $CDCl_3$) δ 8.26 – 8.19 (m, 1H), 7.66 – 7.59 (m, 1H), 7.51 – 7.45 (m, 1H), 7.40 – 7.34 (m, 1H), 7.16 (ABq, 2H, $\Delta\delta_{AB}$ = 0.10 ppm, J_{AB} = 16.3 Hz).

Spectral data were in agreement with those previously reported.[46]

Olefin 6o

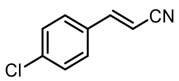

Olefin **6o** was prepared according to the general procedure from **S30** and **S8** and obtained a white solid in 40% yield (13.0 mg, 0.08 mmol, *E/Z* 7:1) after flash column chromatography (5% EtOAc in heptane).

^{1}H NMR (400 MHz, $CDCl_3$) δ 7.39 (app s, 4H), 7.36 (d, *J* = 16.7 Hz, 1H), 5.86 (d, *J* = 16.7 Hz, 1H).

Spectral data were in agreement with those previously reported.[38]

Olefin 6p

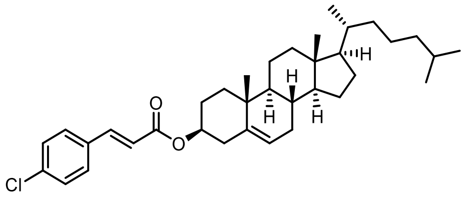

Olefin **6p** was prepared according to the general procedure from **S35** and **S8** and obtained a white solid in 56% yield (62.1 mg, 0.11 mmol) after flash column chromatography (2% EtOAc in heptane).

^{1}H NMR (400 MHz, $CDCl_3$) δ 7.61 (d, *J* = 16.0 Hz, 1H), 7.45 (d, *J* = 8.5 Hz, 2H), 7.39 – 7.32 (m, 2H), 6.39 (d, *J* = 16.0 Hz, 1H), 5.41 (d, *J* = 4.1 Hz, 1H), 4.78 – 4.70 (m, 1H), 2.40 (d, *J* = 7.7 Hz, 2H), 2.05 – 1.78 (m, 5H), 1.73 – 0.97 (m, 24H), 0.92 (d, *J* = 6.5 Hz, 3H), 0.87 (dd, *J* = 6.6, 1.7 Hz, 6H), 0.69 (s, 3H).

Spectral data were in agreement with those previously reported.[42]

7. Limitation

Direct stereodivergent olefination of carbonyl compounds with sulfur ylides

Jérémy Merad[‡,a], Phillip Grant[‡,a], Tobias Stopka[‡,a], Juliette Sabbatani[a], Ricardo Meyrelles[a,b,c], Alexander Preinfalk[a], Ján Matyasovsky[a], Boris Maryasin[a,b], Leticia González[b] and Nuno Maulide[a,*].

[a]Institute of Organic Chemistry, University of Vienna, 1090 Vienna, Austria; [b]Institute of Theoretical Chemistry, University of Vienna, 1090 Vienna, Austria; [c]Doctoral School in Chemistry, 1090 Vienna, University of Vienna, Austria

Olefination, Sulfur ylide, Alkene, Thiouronium

ABSTRACT: The reactivity of phosphorus and sulfur ylides toward carbonyl compounds constitutes a well-known dichotomy that is a common educational device in organic chemistry—the former gives olefins, while the latter gives epoxides. Herein, we report a stereodivergent carbonyl olefination which challenges this dichotomy, showcasing thiouronium ylides as valuable olefination reagents. With this method, aldehydes are converted to *Z*-alkenes with high stereoselectivity and broad substrate scope, while *N*-tosylimines provide a similarly proficient entry to *E*-alkenes. In-depth computational and experimental studies clarified the mechanistic details of this unusual reactivity.

Alkenes are among the most prevalent functional groups in natural products and industrial chemicals, with one cheminformatics study estimating that 40% of the former contain an alkene.[1] As such, the development of olefination methods has been a central and rewarding challenge to organic chemistry,[2] contributing some of the most valued reactions in the 'synthetic toolbox'.[3] Nevertheless, the wide structural and electronic parameters of olefin chemical space continue to pose a challenge, implying that no single method is universally apt for their synthesis. As a result, the development of complementary olefination methods remains an active area of research.

The Wittig olefination is part of a mechanistic dichotomy that is a common educational device in organic chemistry.[4,5] It is generally accepted to proceed by addition of a phosphorous ylide to an aldehyde or ketone to give an oxaphosphetane, which then undergoes cycloreversion to produce an alkene and a phosphine oxide (Figure 1A).[6,7] The major thermodynamic driving force for this reaction is known to be the strength of the resulting phosphorus-oxygen double-bond.[5] Notably, the reaction of a sulfur-ylide—the Corey-Chaykovsky reaction—follows a different pathway, involving an intermediate betaine and resulting in formation of an epoxide by displacement the sulfonium group (Figure 1A).[8–10] This textbook difference in reactivity is attributed to the lower oxophilicity of sulfur, the better leaving-group ability of the sulfonium group, and kinetic factors.[5,11,12] The sulfur-phosphorus ylide dichotomy is therefore commonly used in chemical education to convey the concepts of leaving group ability, oxophilicity, as well as kinetic/thermodynamic reaction control.[4]

Our group's long-standing interest in novel olefination methods[13,14] and sulfur ylide reactivity[15] led us to interrogate the universality of the phosphorus/sulfur ylide dichotomy in organic chemistry. Herein, we report a novel carbonyl olefination method relying on thiouronium ylides which challenges this dichotomy (Figure 1B). This method selectively affords *Z*-alkenes from aldehydes and *E*-alkenes from *N*-tosylimines, typically in greater that 20:1 selectivity, while exhibiting broad substrate scope, making it suitable for late-stage functionalization.

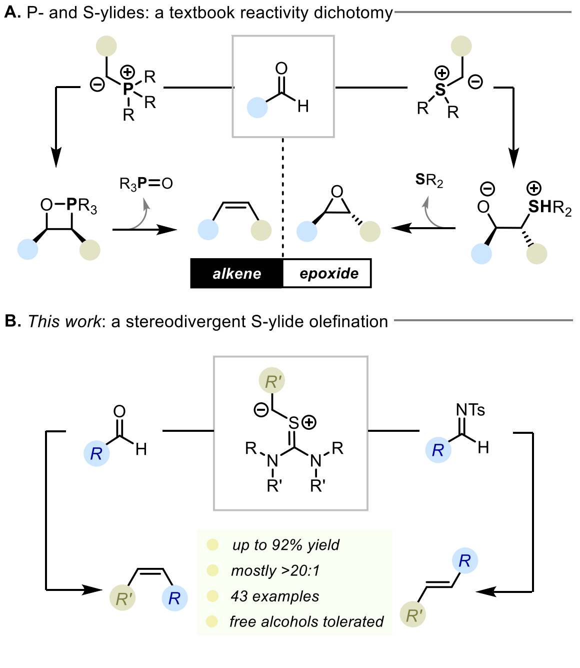

Figure 1. Revisiting the textbook reactivity dichotomy of phosphorus and sulfur ylides with carbonyl compounds

Our group recently reported the reaction of thiouronium salts with alcohols to afford thioethers without requiring

S

During the course of our investigations, we identified certain substrates that did not perform satisfactorily under the typical conditions. These are summarised as follows:

- *Highly electron rich aldehydes:* aldehydes bearing strong electron-donating groups in conjugation with the aldehyde (effectively vinylogous esters/vinylogous amides) were poor substrates. This is most likely due to a lack of electrophilicity.
- *Unstable ylides*: the presented conditions are generally not suitable for the olefination of ylides that are not stabilised by an EWG – efforts to extend this methodology to such ylides are ongoing in our group.
- *Highly bulky aldehydes*: aldehydes bearing a quaternary center at the α-position were poor substrates likely due to high steric hindrance.
- *Alkyl tosyl imines:* for the *E*-selective protocol, alkyl tosyl imines were generally observed to be poor substrates. This may be due to competitive deprotonation by BTMG.
- *Trisubsituted olefin products*: This method can be used to access trisubstituted olefins, under the present conditions the stereoselectivity is modest. Efforts to improve this selectivity are ongoing.

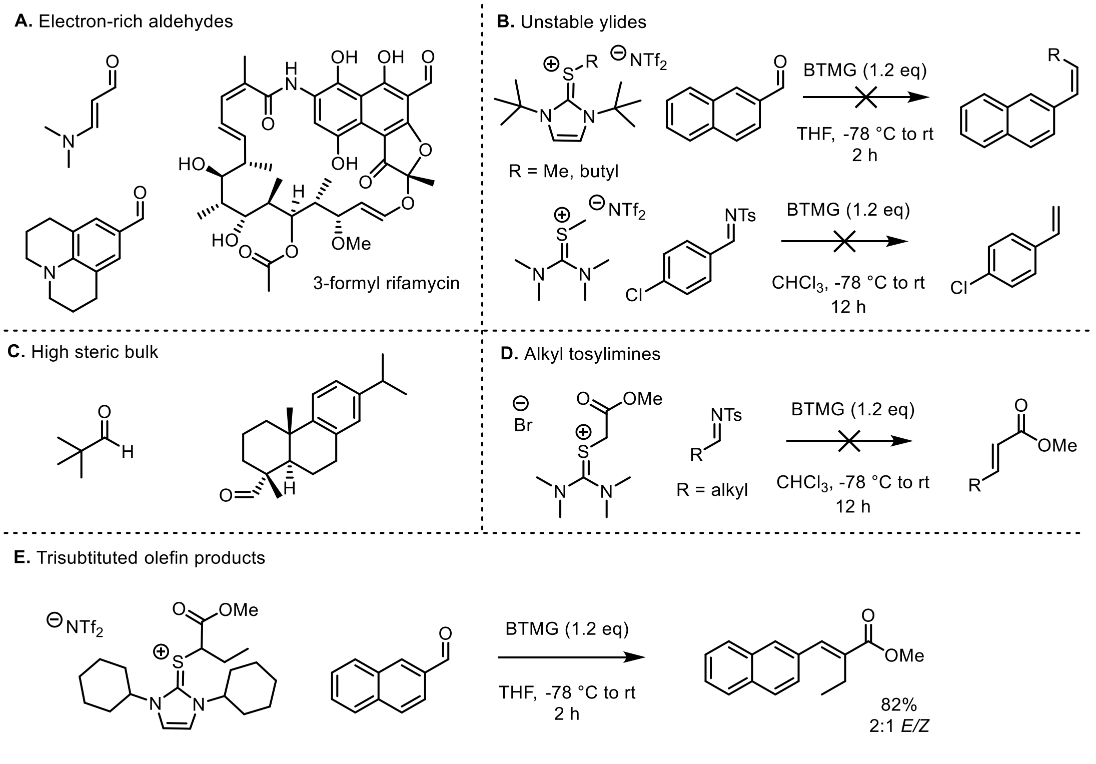

8. NMR spectra

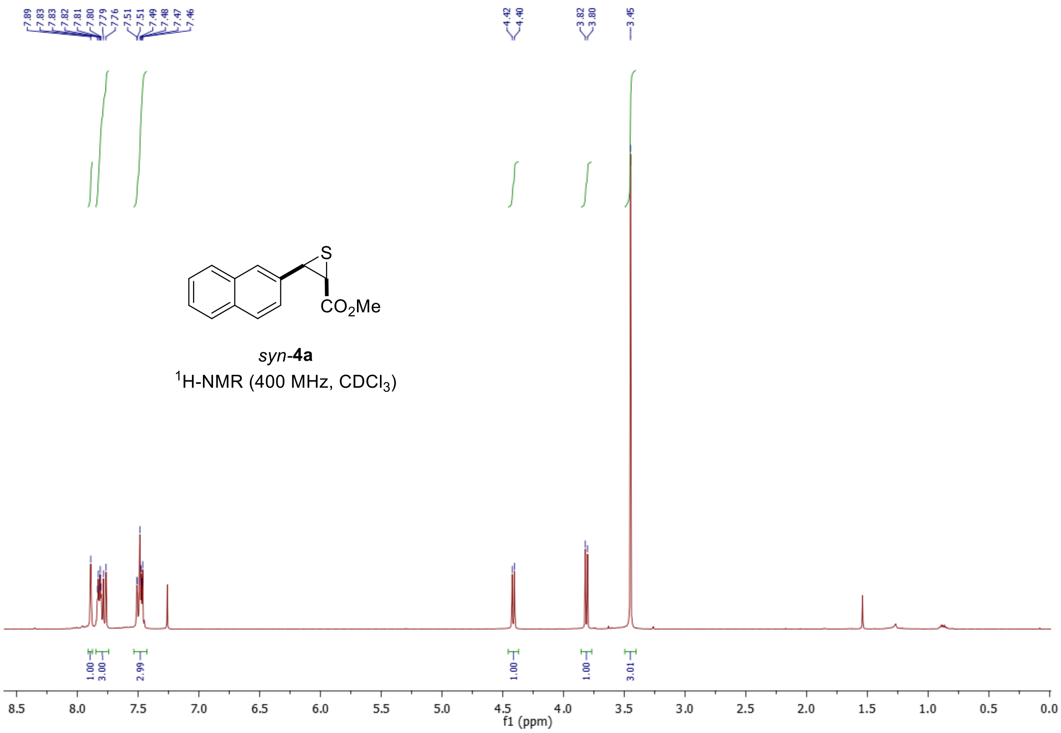

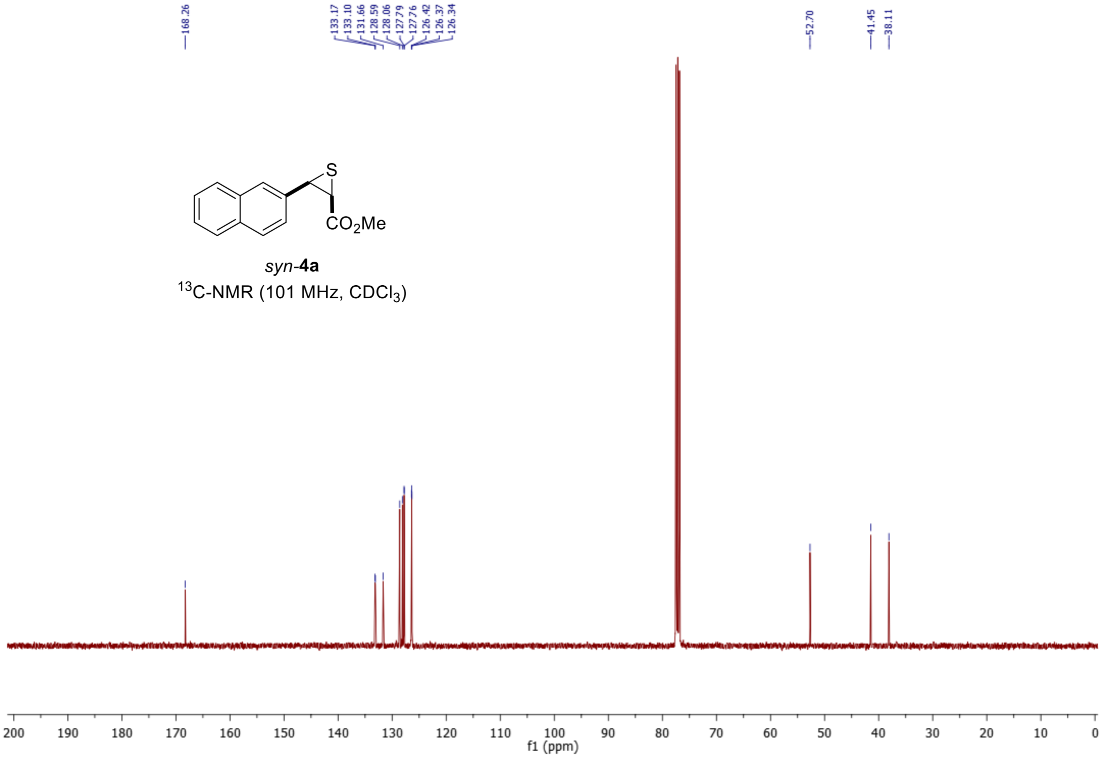

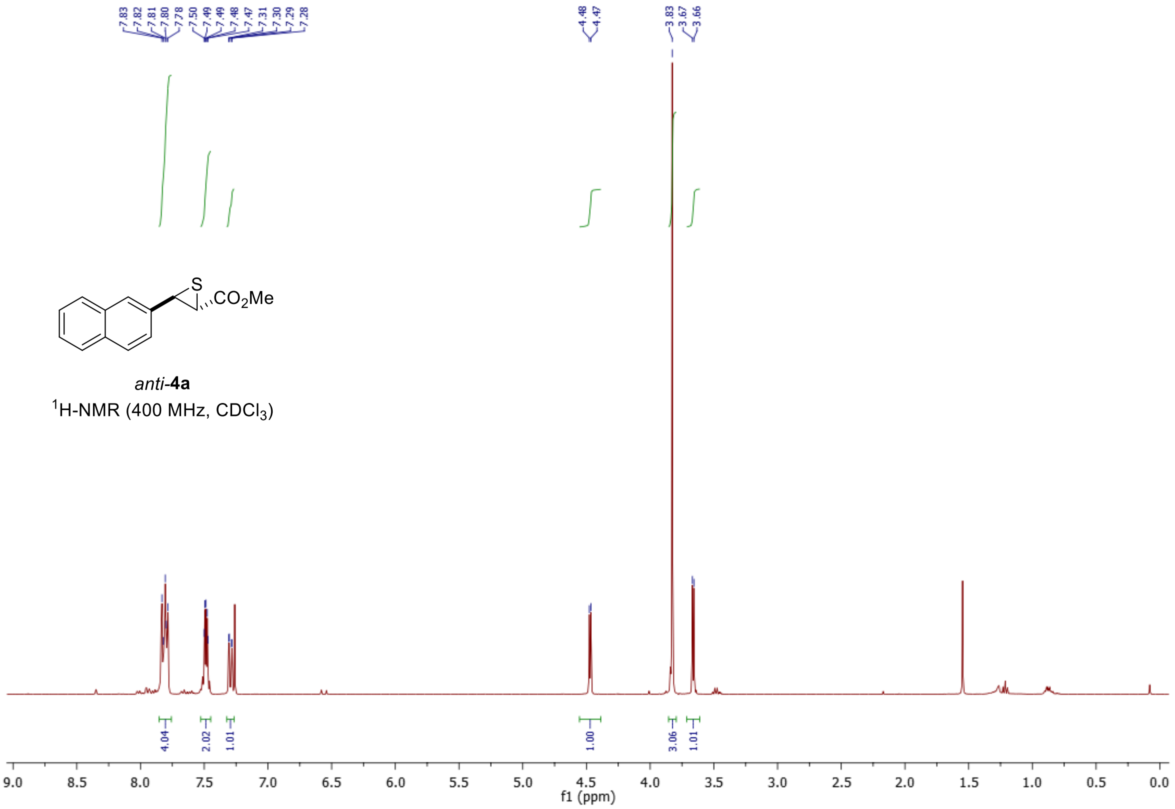

S
CO2Me
anti-4a
1H-NMR (400 MHz, CDCl3)
7.83 7.82 7.81 7.80 7.78 7.50 7.49 7.49 7.48 7.47 7.31 7.30 7.29 7.28
4.48 4.47
3.83 3.67 3.66
4.04 2.02 1.01 1.00 3.06 1.01
9.0 8.5 8.0 7.5 7.0 6.5 6.0 5.5 5.0 4.5 4.0 3.5 3.0 2.5 2.0 1.5 1.0 0.5 0.0
f1 (ppm)

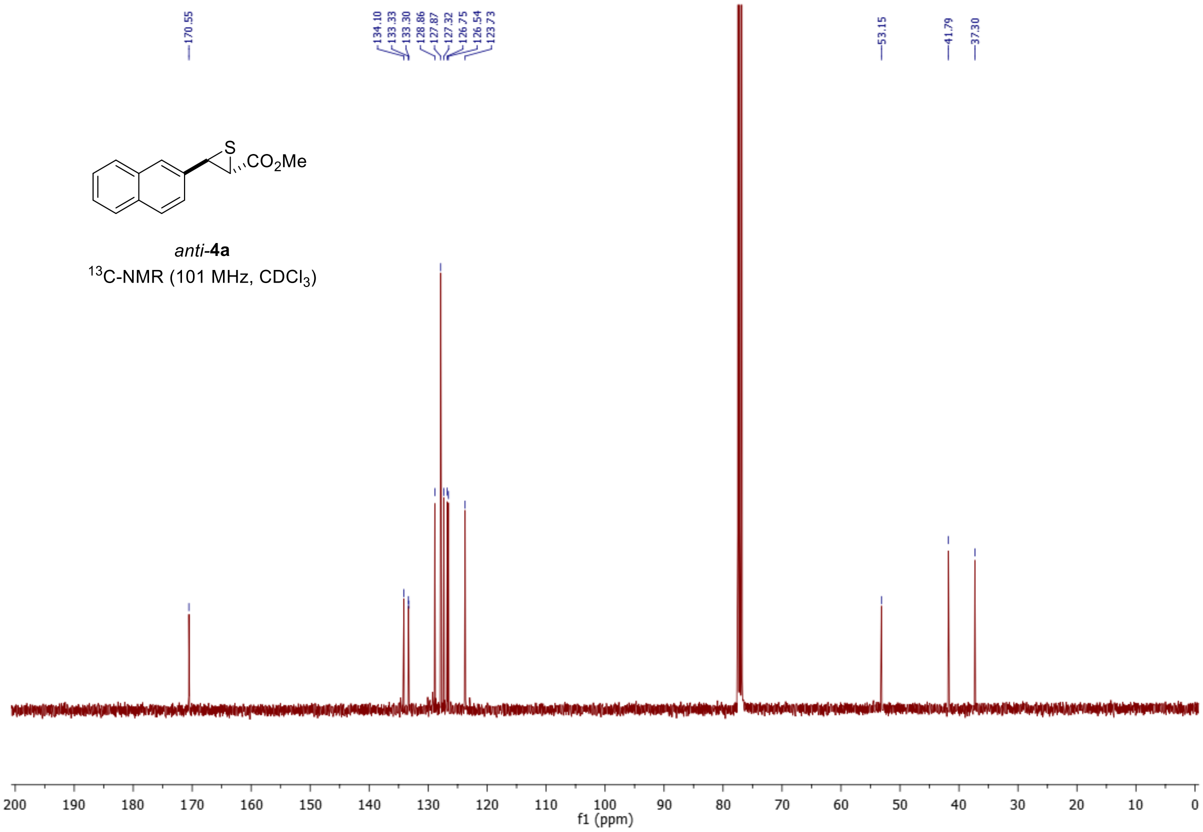

S
CO2Me
anti-4a
13C-NMR (101 MHz, CDCl3)
170.55
134.10 133.33 133.30 128.86 127.87 127.32 126.75 126.54 123.73
53.15
41.79
37.30
200 190 180 170 160 150 140 130 120 110 100 90 80 70 60 50 40 30 20 10 0
f1 (ppm)

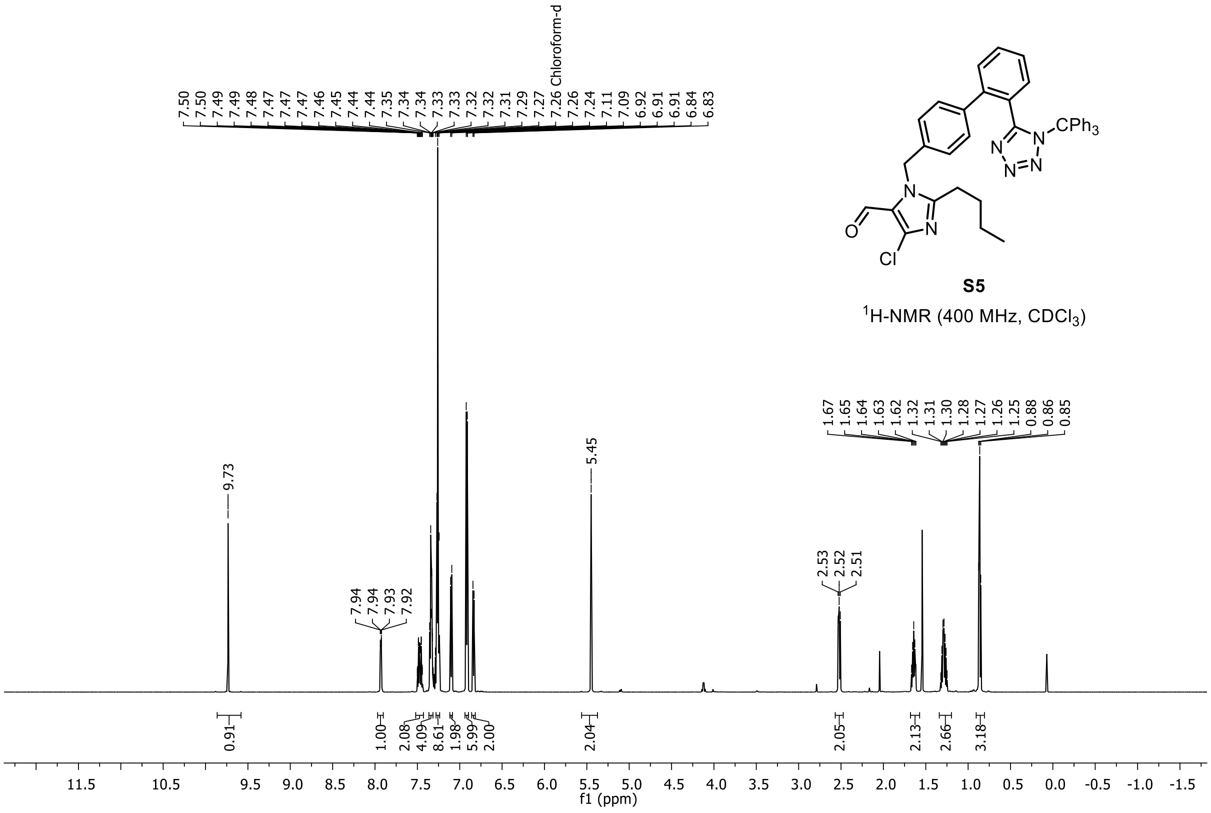
7.50 7.50 7.49 7.48 7.47 7.47 7.46 7.45 7.44 7.44 7.35 7.34 7.34 7.33 7.33 7.32 7.32 7.31 7.29 7.27 7.26 Chloroform-d 7.26 7.24 7.11 7.09 6.92 6.91 6.91 6.84 6.83
9.73
7.94 7.94 7.93 7.92
5.45
2.53 2.52 2.51
1.67 1.65 1.64 1.63 1.62 1.32 1.31 1.30 1.28 1.27 1.26 1.25 0.88 0.86 0.85
0.91 1.00 2.08 4.09 8.61 1.98 5.99 2.00 2.04 2.05 2.13 2.66 3.18
CPh3
N
O
Cl
S5
1H-NMR (400 MHz, CDCl3)
f1 (ppm)

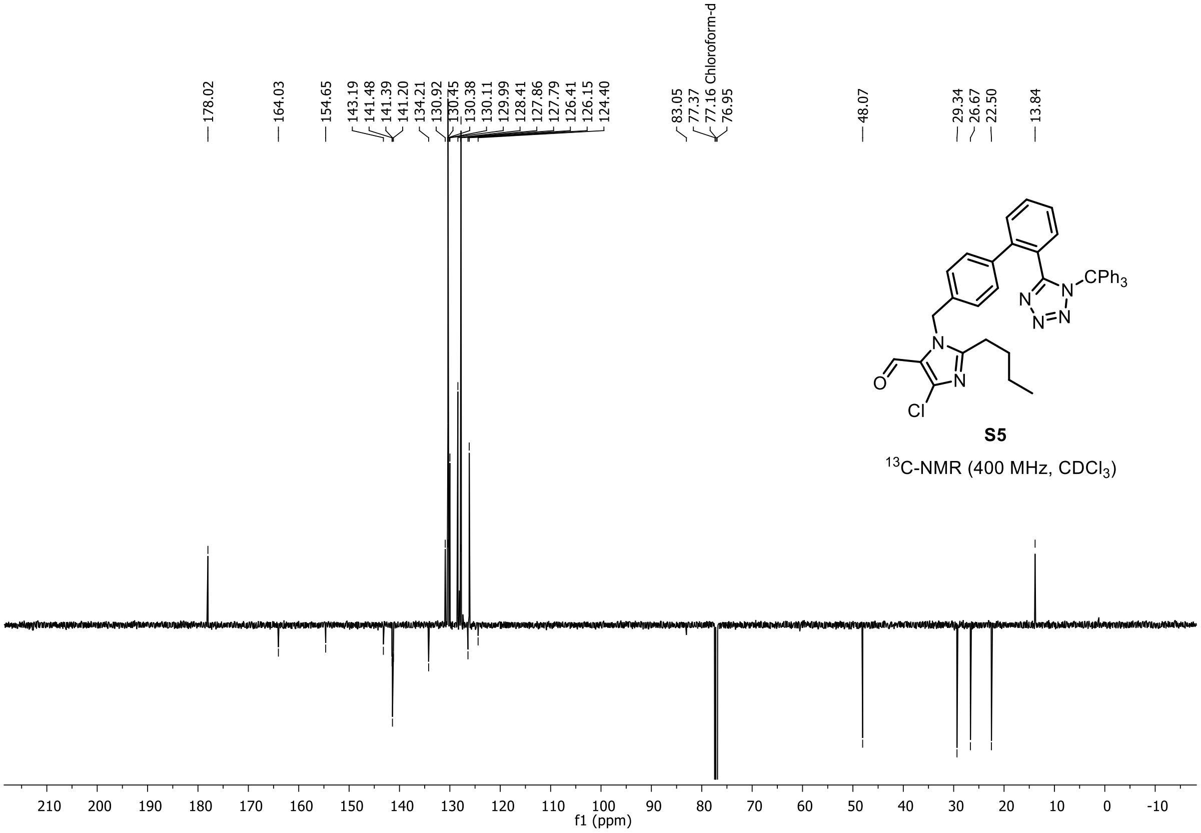
178.02
164.03
154.65
143.19 141.48 141.39 141.20 134.21 130.92 130.45 130.38 130.11 129.99 128.41 127.86 127.79 126.41 126.15 124.40
83.05 77.37 77.16 Chloroform-d 76.95
48.07
29.34 26.67 22.50
13.84
CPh3
N
O
Cl
S5
13C-NMR (400 MHz, CDCl3)
f1 (ppm)

8.1 Thioureas

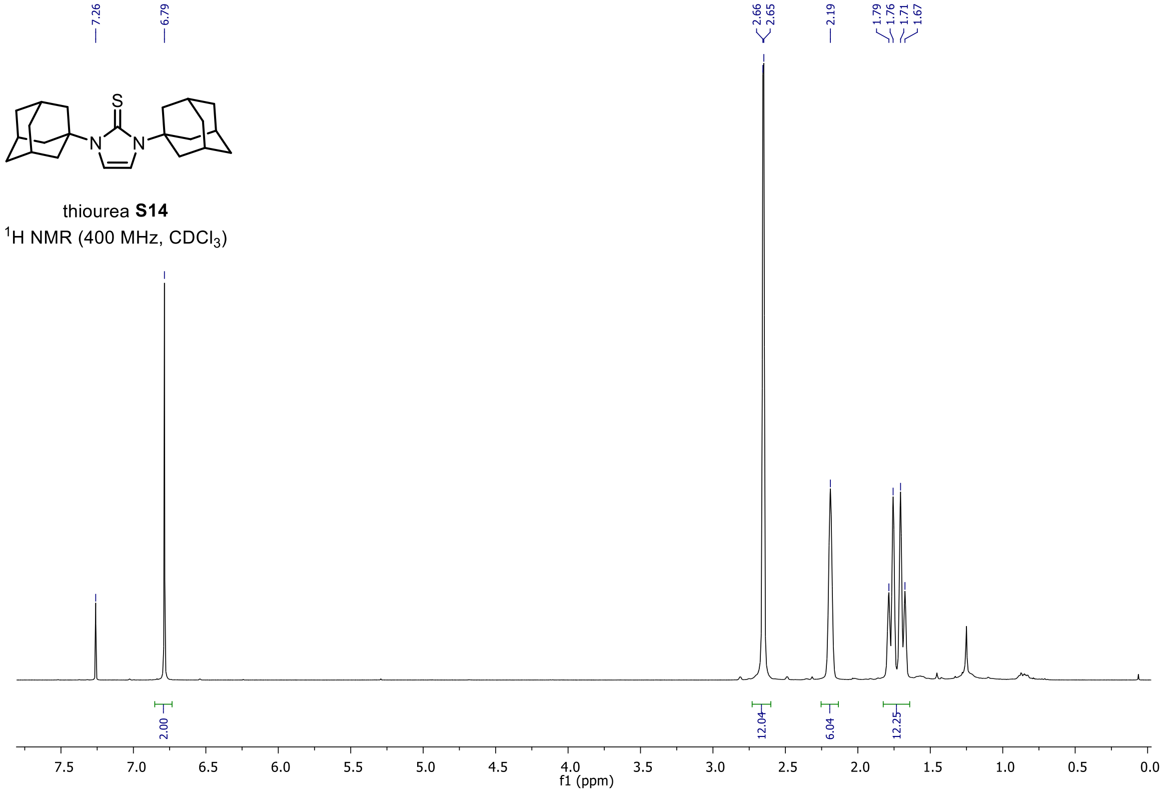

thiourea **S14**

^{1}H NMR (400 MHz, $CDCl_3$)

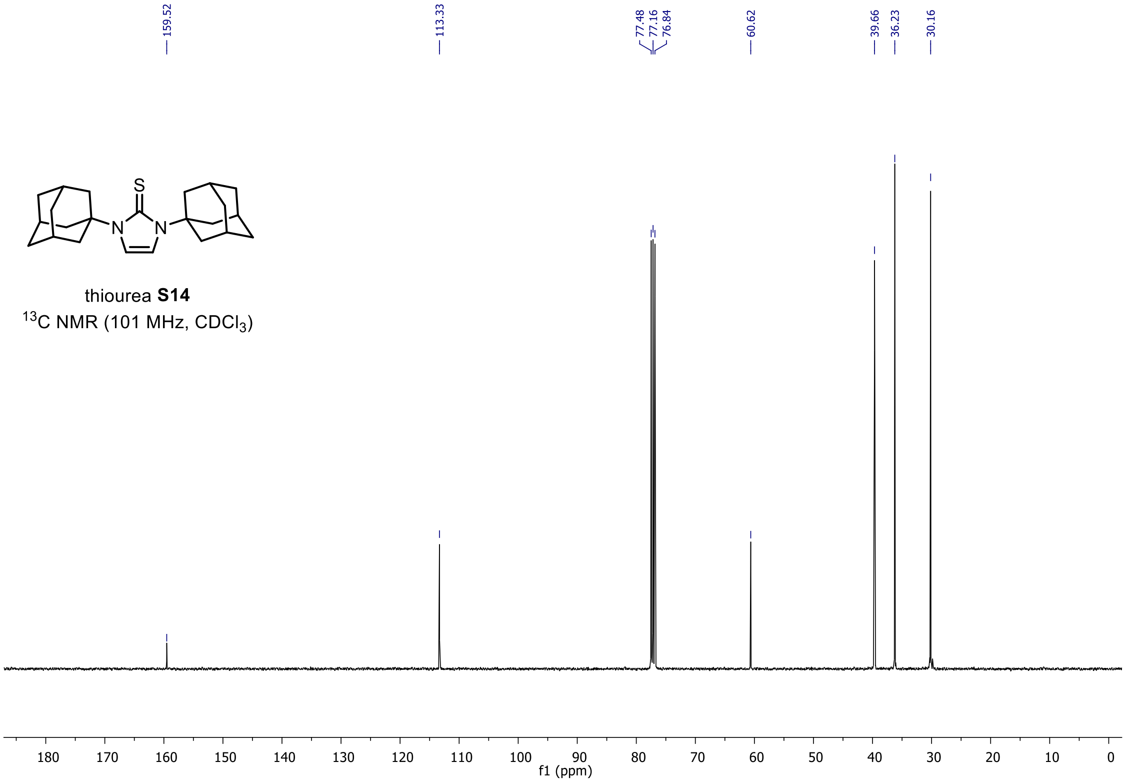

thiourea **S14**

^{13}C NMR (101 MHz, $CDCl_3$)

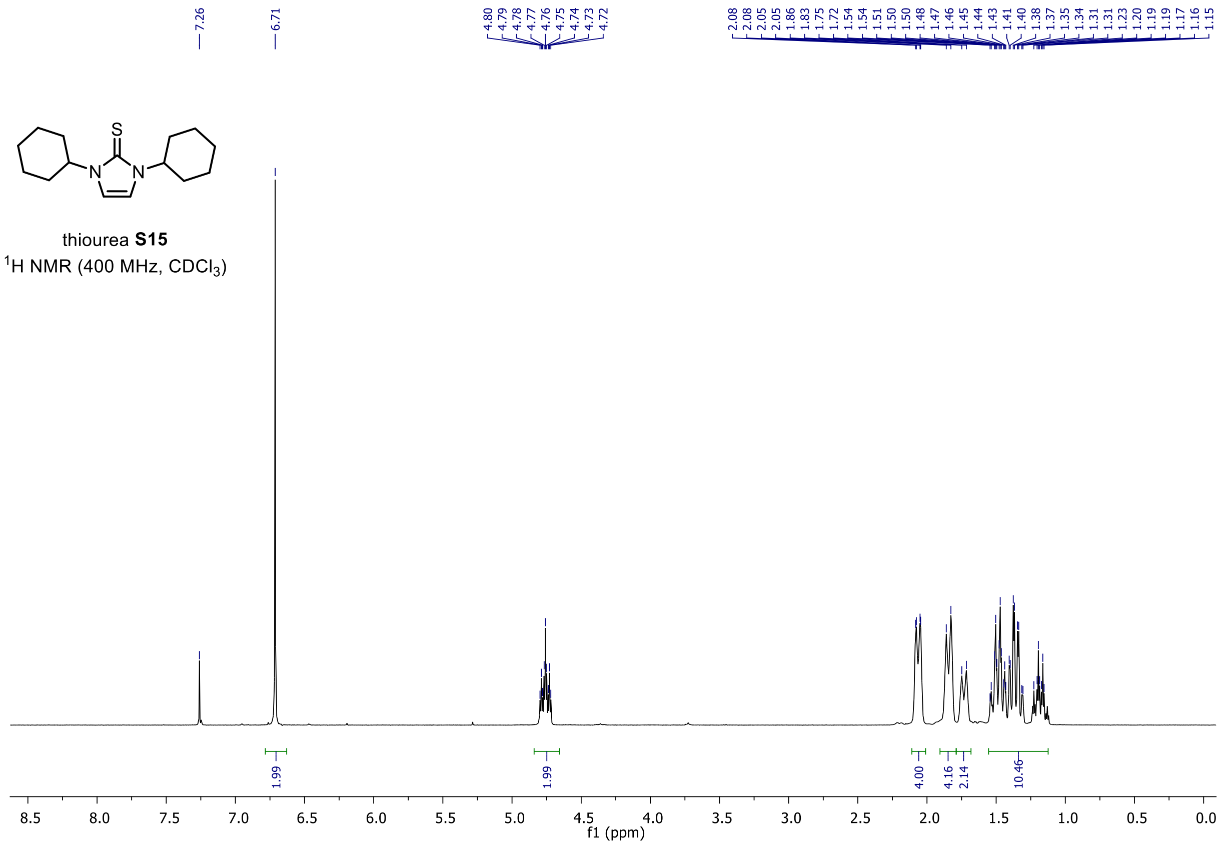

thiourea **S15**
^{1}H NMR (400 MHz, $CDCl_3$)
7.26
6.71
4.80 4.79 4.78 4.77 4.76 4.75 4.74 4.73 4.72
2.08 2.08 2.05 2.05 1.86 1.83 1.75 1.72 1.54 1.54 1.51 1.50 1.50 1.48 1.47 1.46 1.45 1.44 1.43 1.41 1.40 1.38 1.37 1.35 1.34 1.31 1.31 1.23 1.20 1.19 1.19 1.17 1.16 1.15
1.99
1.99
4.00
4.16
2.14
10.46
f1 (ppm)

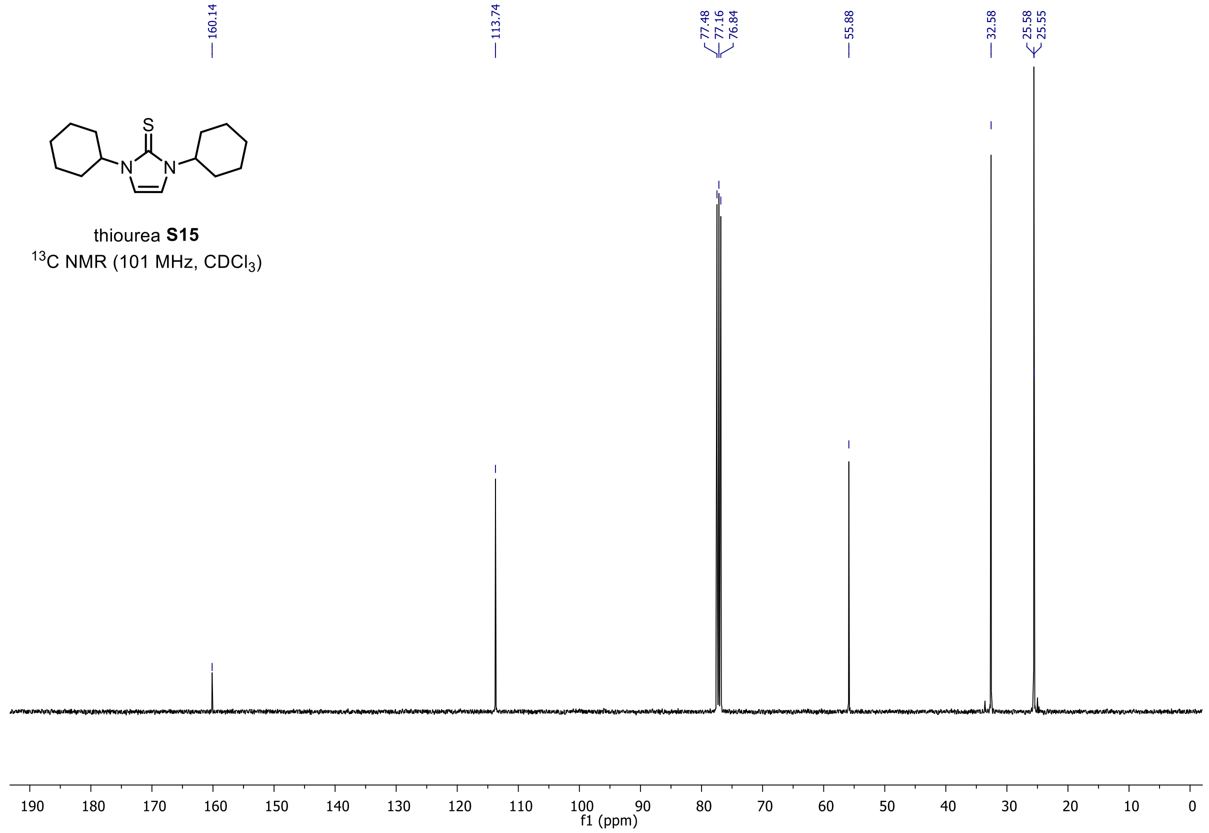

thiourea **S15**
^{13}C NMR (101 MHz, $CDCl_3$)
160.14
113.74
77.48 77.16 76.84
55.88
32.58
25.58 25.55
f1 (ppm)

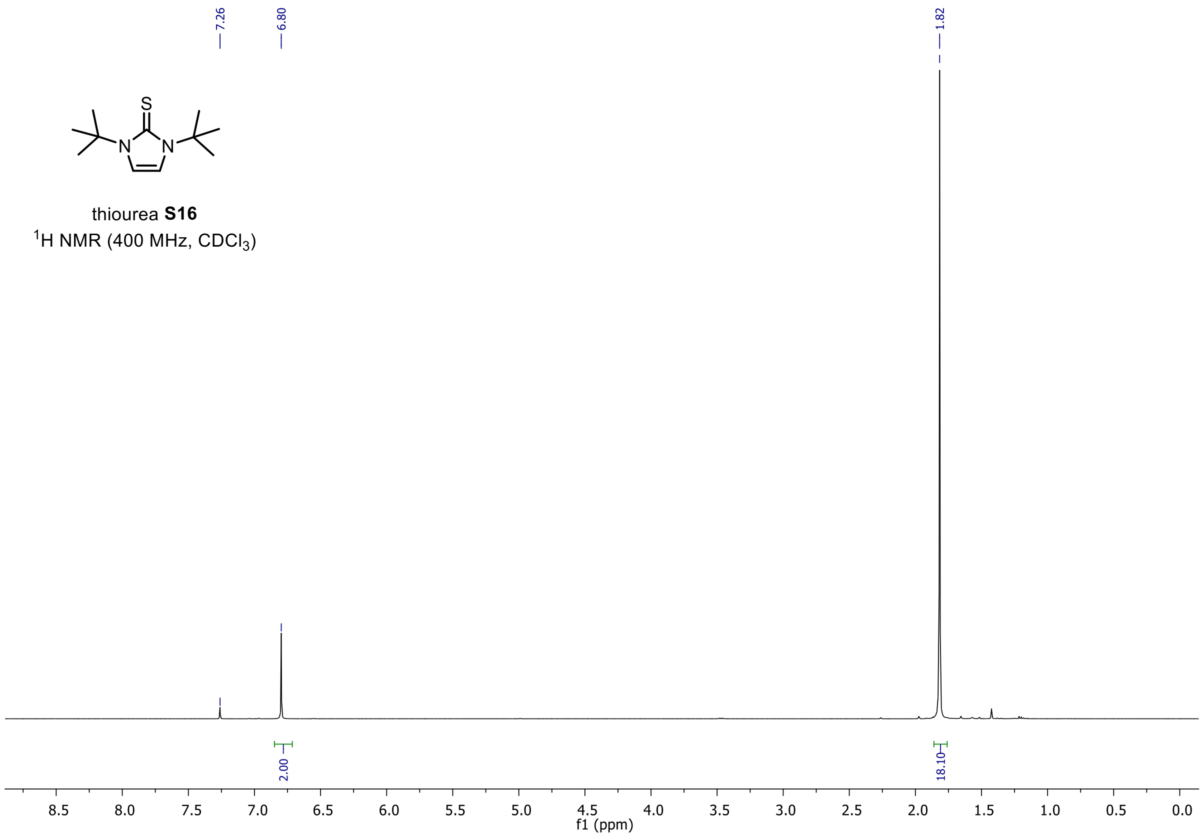
7.26
6.80
1.82
thiourea **S16**
^{1}H NMR (400 MHz, $CDCl_3$)
2.00
18.10
8.5 8.0 7.5 7.0 6.5 6.0 5.5 5.0 4.5 4.0 3.5 3.0 2.5 2.0 1.5 1.0 0.5 0.0
f1 (ppm)

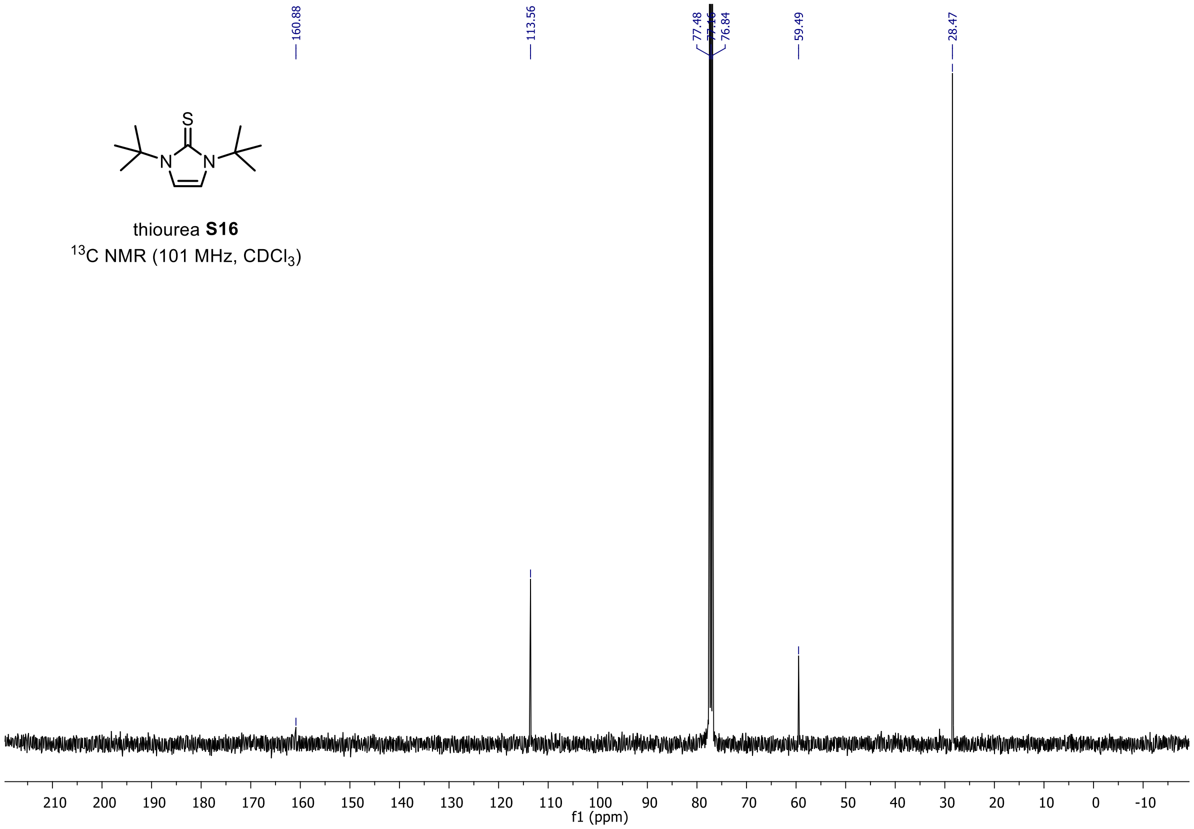
160.88
113.56
77.48
76.84
59.49
28.47
thiourea **S16**
^{13}C NMR (101 MHz, $CDCl_3$)
210 200 190 180 170 160 150 140 130 120 110 100 90 80 70 60 50 40 30 20 10 0 -10
f1 (ppm)

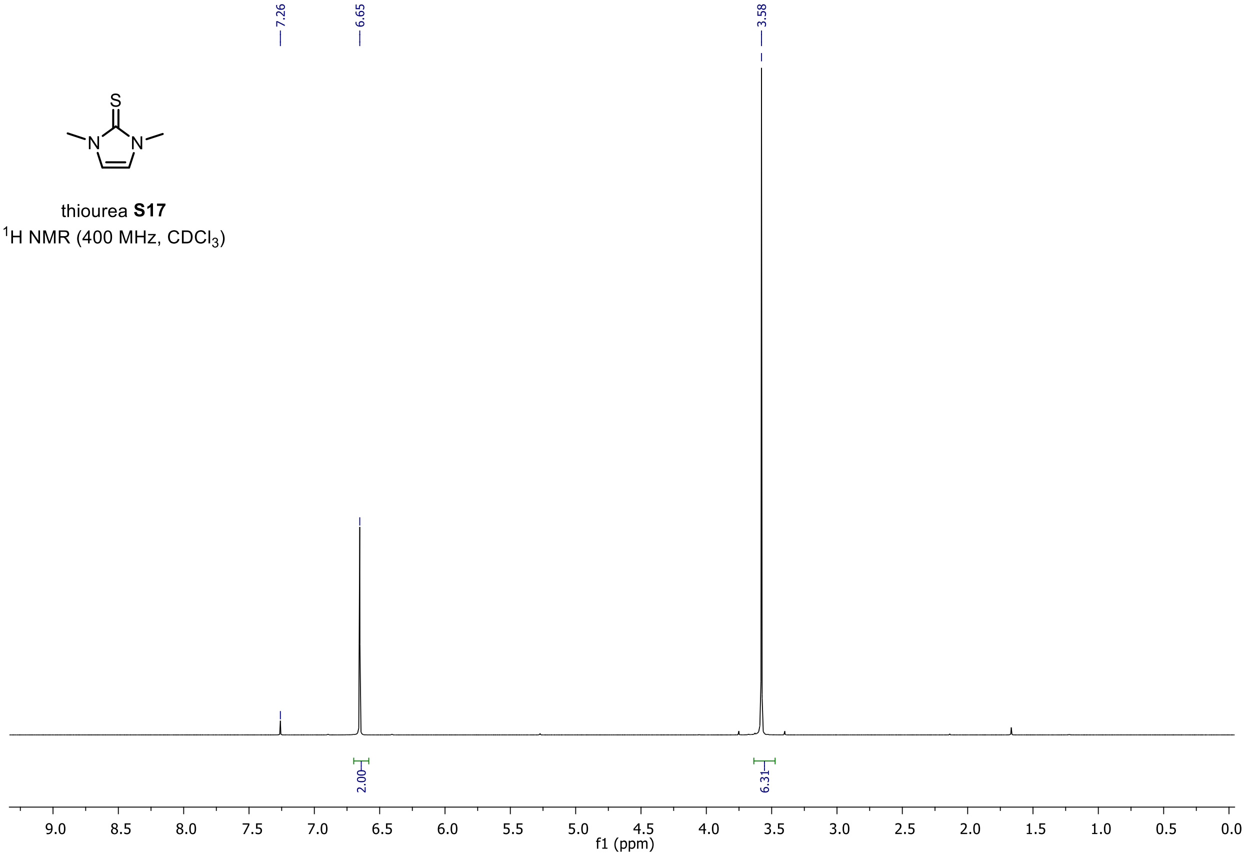

thiourea **S17**
^{1}H NMR (400 MHz, $CDCl_3$)

8.2 Thiouronium bromides

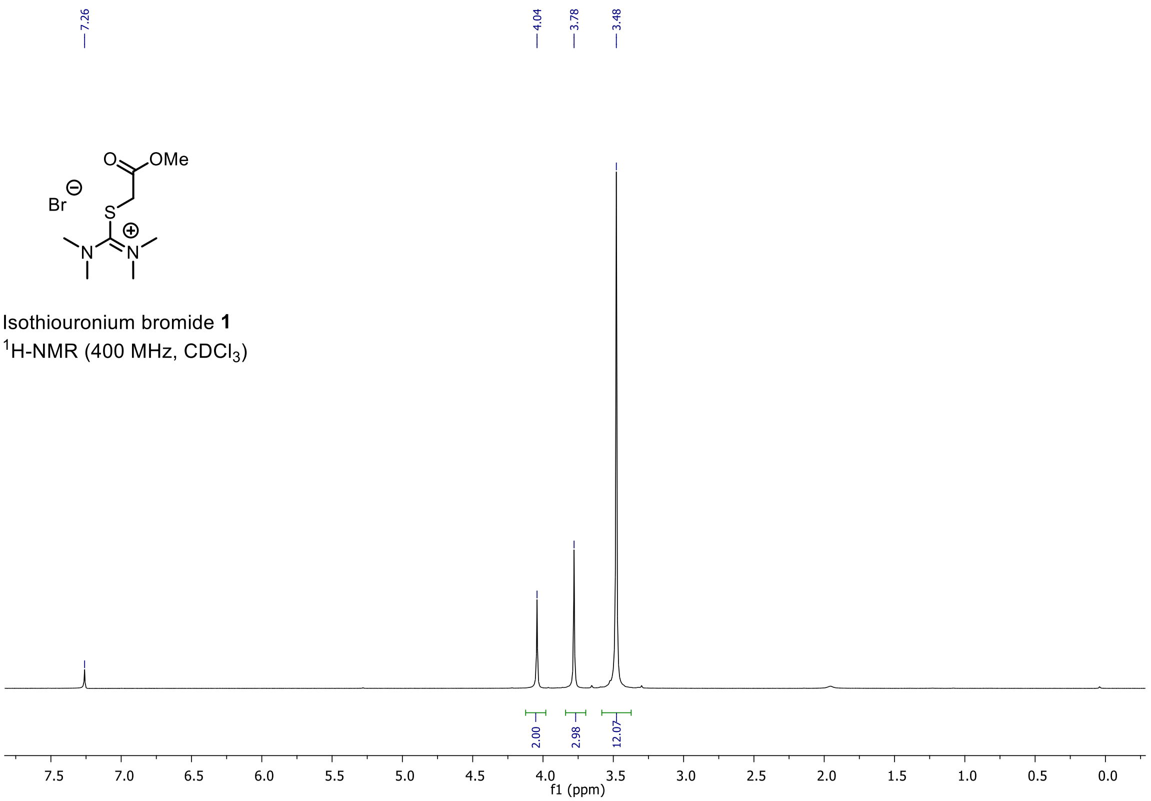

Isothiouronium bromide **1**
^{1}H-NMR (400 MHz, $CDCl_3$)

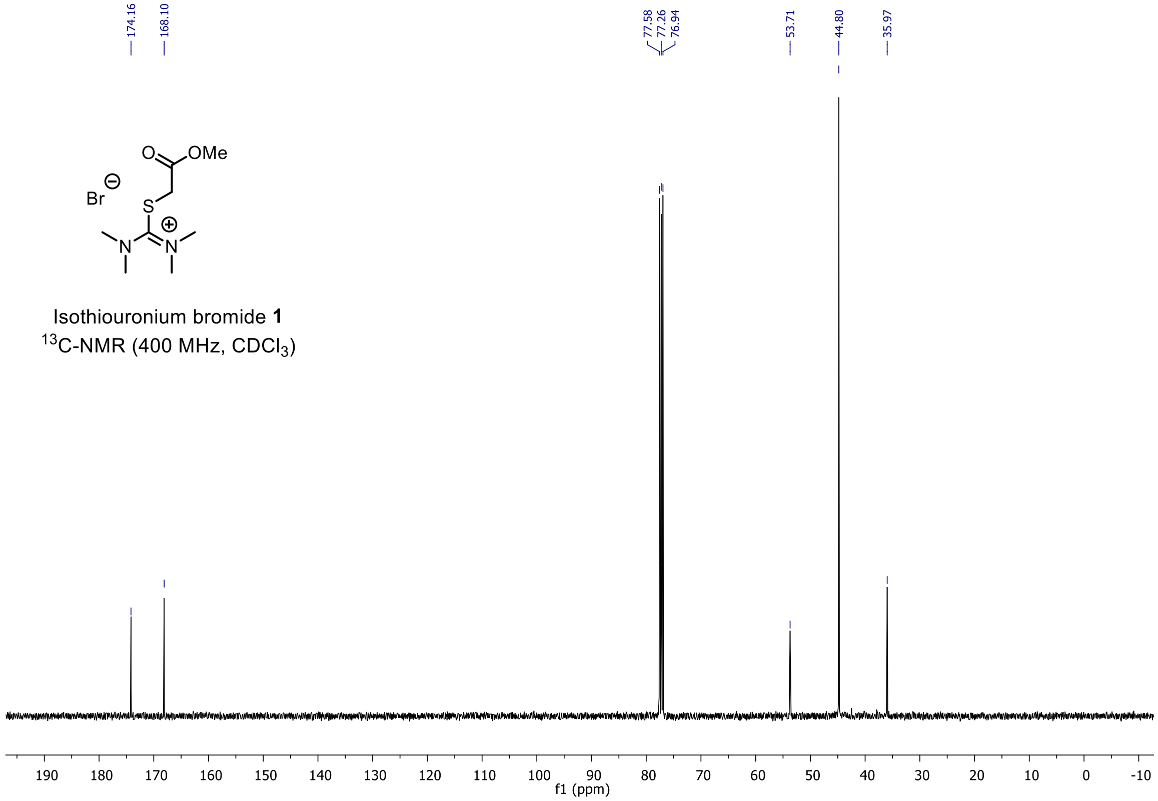

Isothiouronium bromide **1**
^{13}C-NMR (400 MHz, $CDCl_3$)

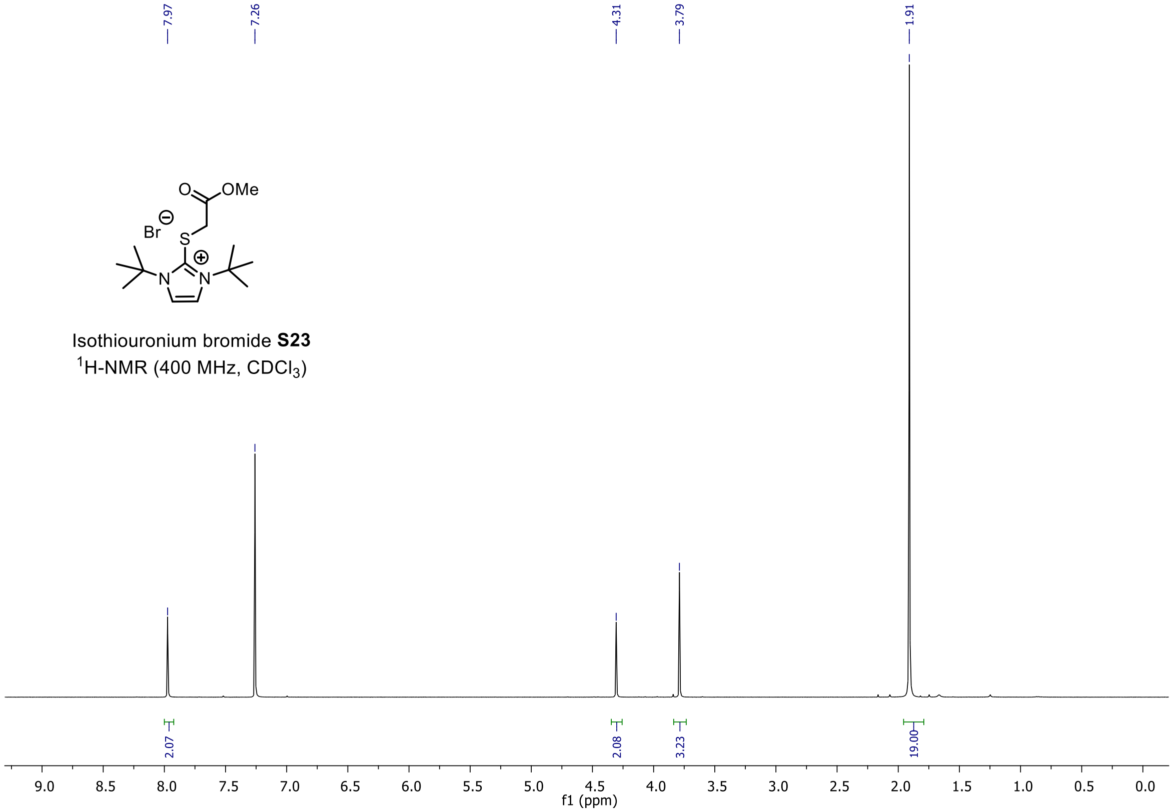

Isothiouronium bromide **S23**
^{1}H-NMR (400 MHz, $CDCl_3$)

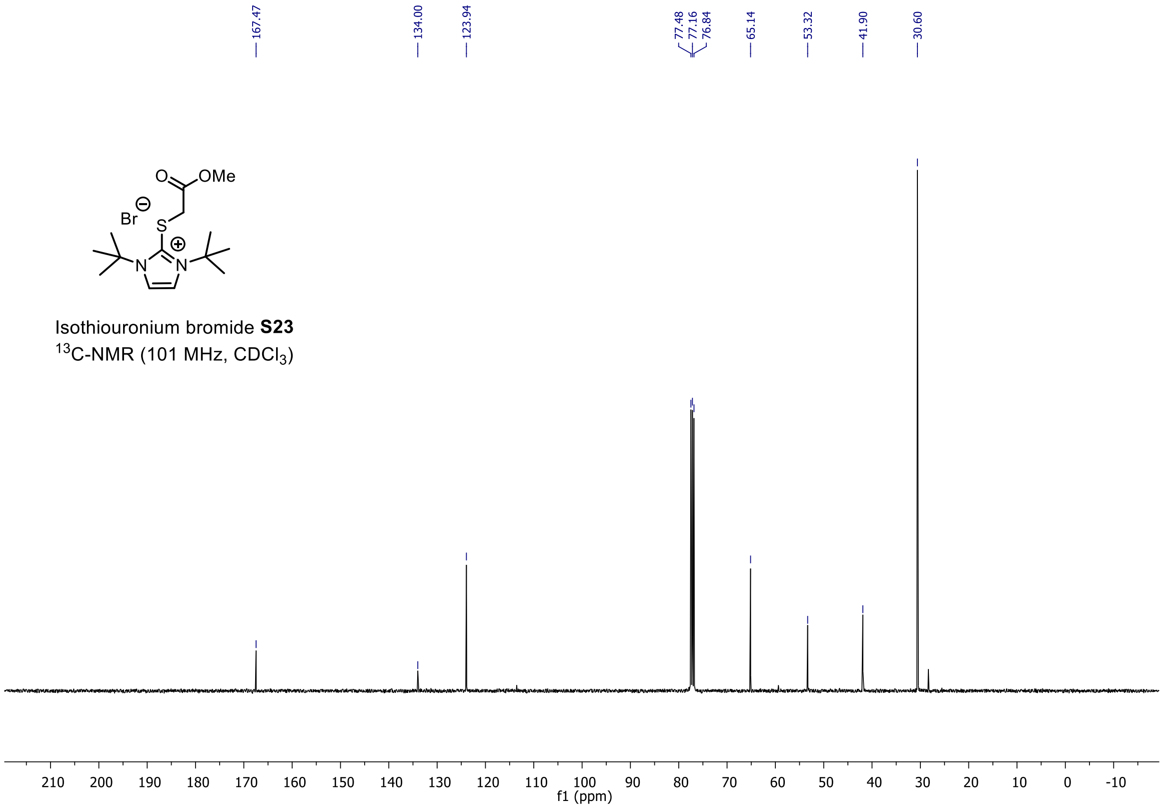

Isothiouronium bromide **S23**
^{13}C-NMR (101 MHz, $CDCl_3$)

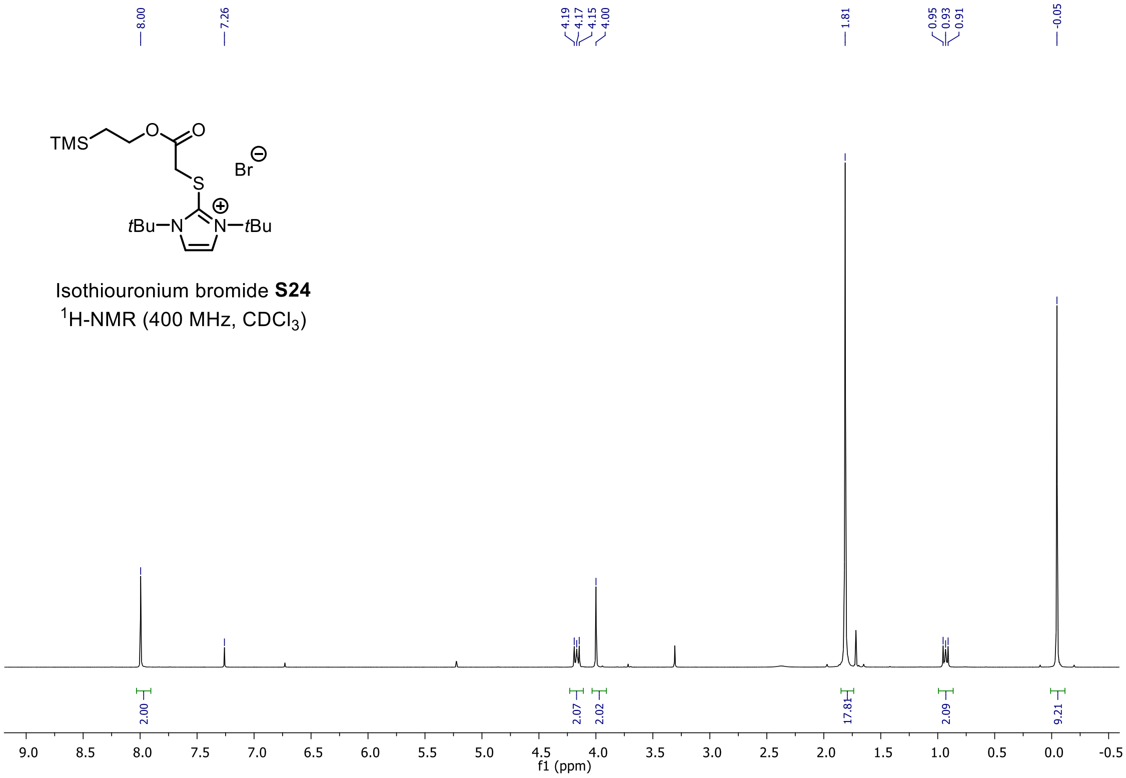
8.00
7.26
4.19
4.17
4.15
4.00
1.81
0.95
0.93
0.91
-0.05
TMS
O
O
Br
S
tBu
N
N
tBu
Isothiouronium bromide **S24**
^{1}H-NMR (400 MHz, $CDCl_3$)
2.00
2.07
2.02
17.81
2.09
9.21
9.0 8.5 8.0 7.5 7.0 6.5 6.0 5.5 5.0 4.5 4.0 3.5 3.0 2.5 2.0 1.5 1.0 0.5 0.0 -0.5
f1 (ppm)

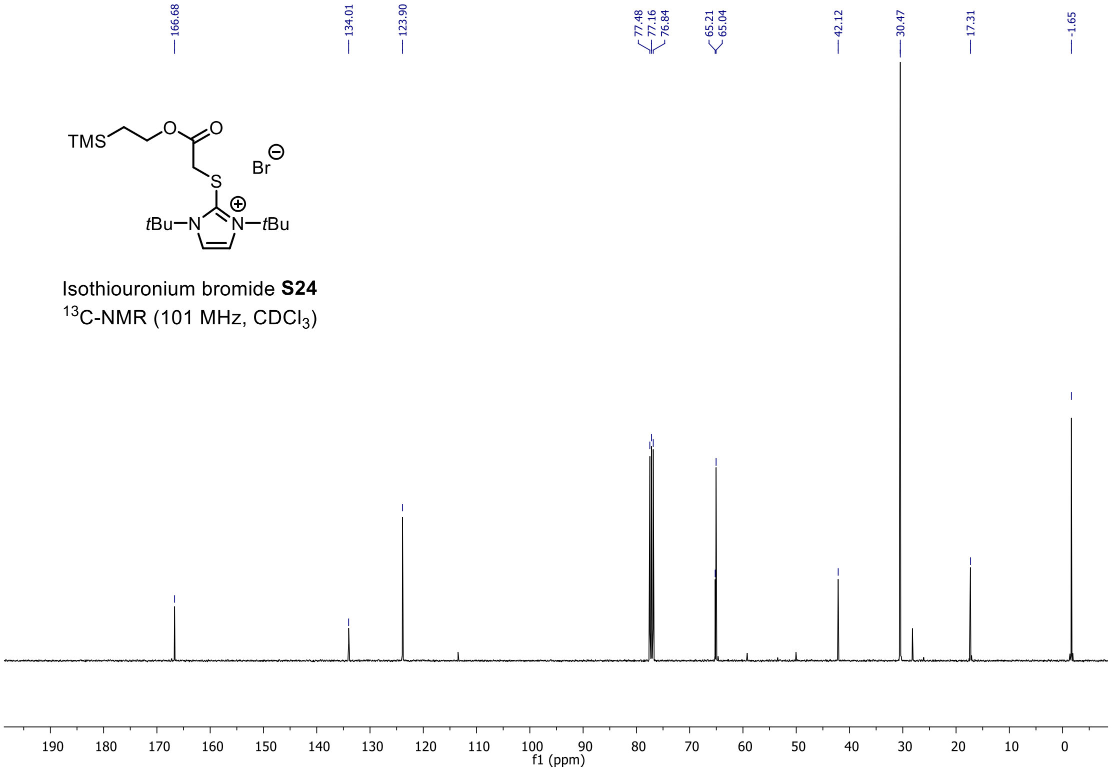
166.68
134.01
123.90
77.48
77.16
76.84
65.21
65.04
42.12
30.47
17.31
-1.65
TMS
O
O
Br
S
tBu
N
N
tBu
Isothiouronium bromide **S24**
^{13}C-NMR (101 MHz, $CDCl_3$)
190 180 170 160 150 140 130 120 110 100 90 80 70 60 50 40 30 20 10 0
f1 (ppm)

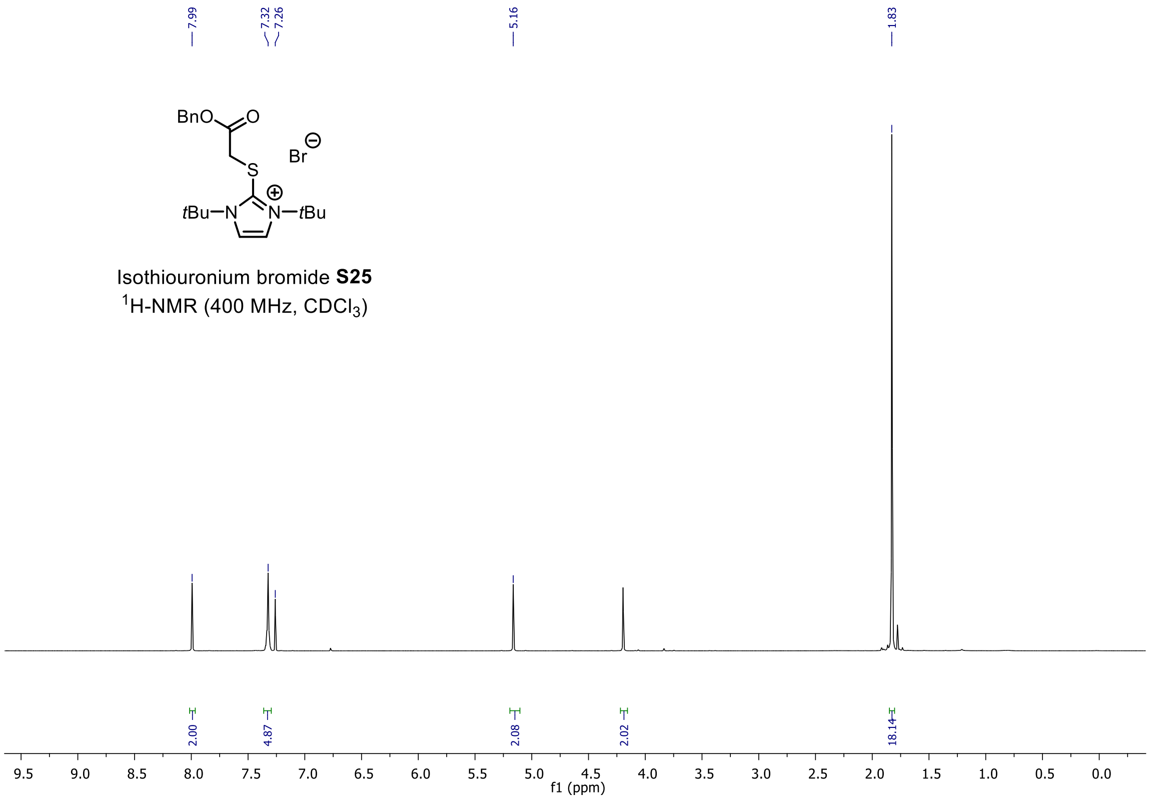

Isothiouronium bromide **S25**
^{1}H-NMR (400 MHz, $CDCl_3$)

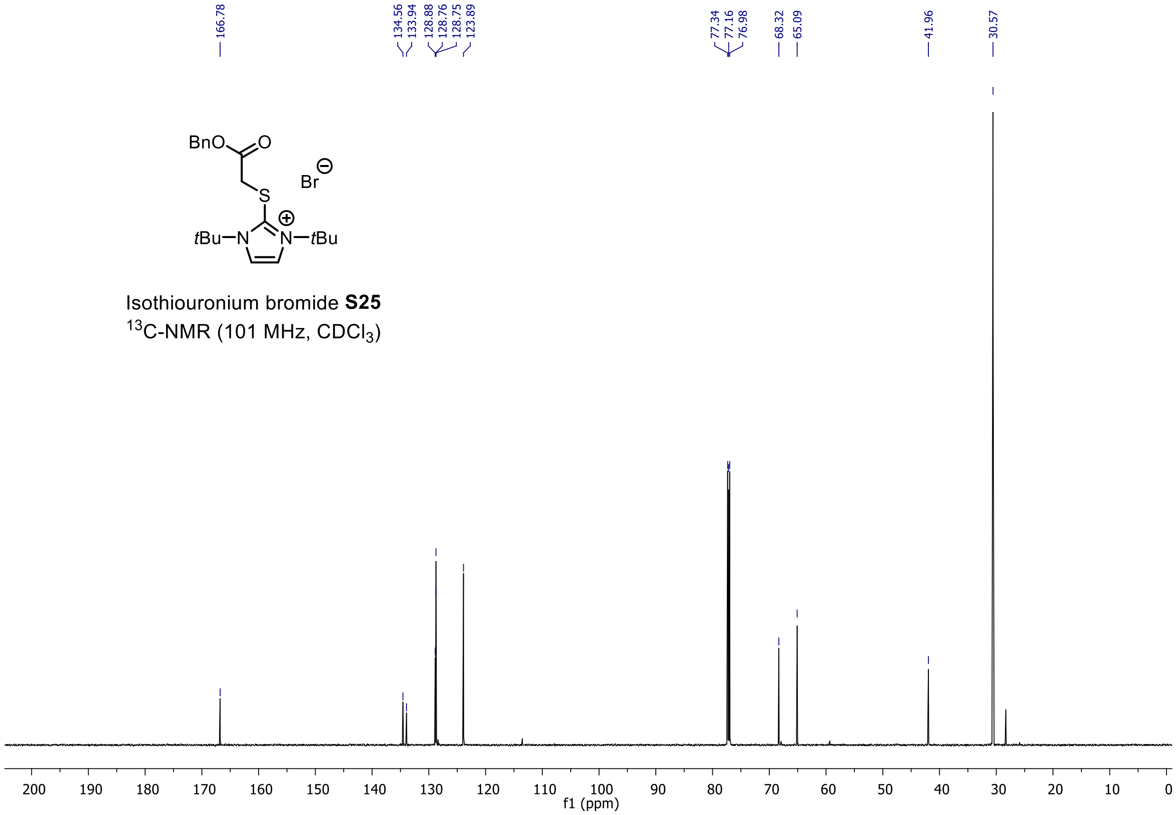

Isothiouronium bromide **S25**
^{13}C-NMR (101 MHz, $CDCl_3$)

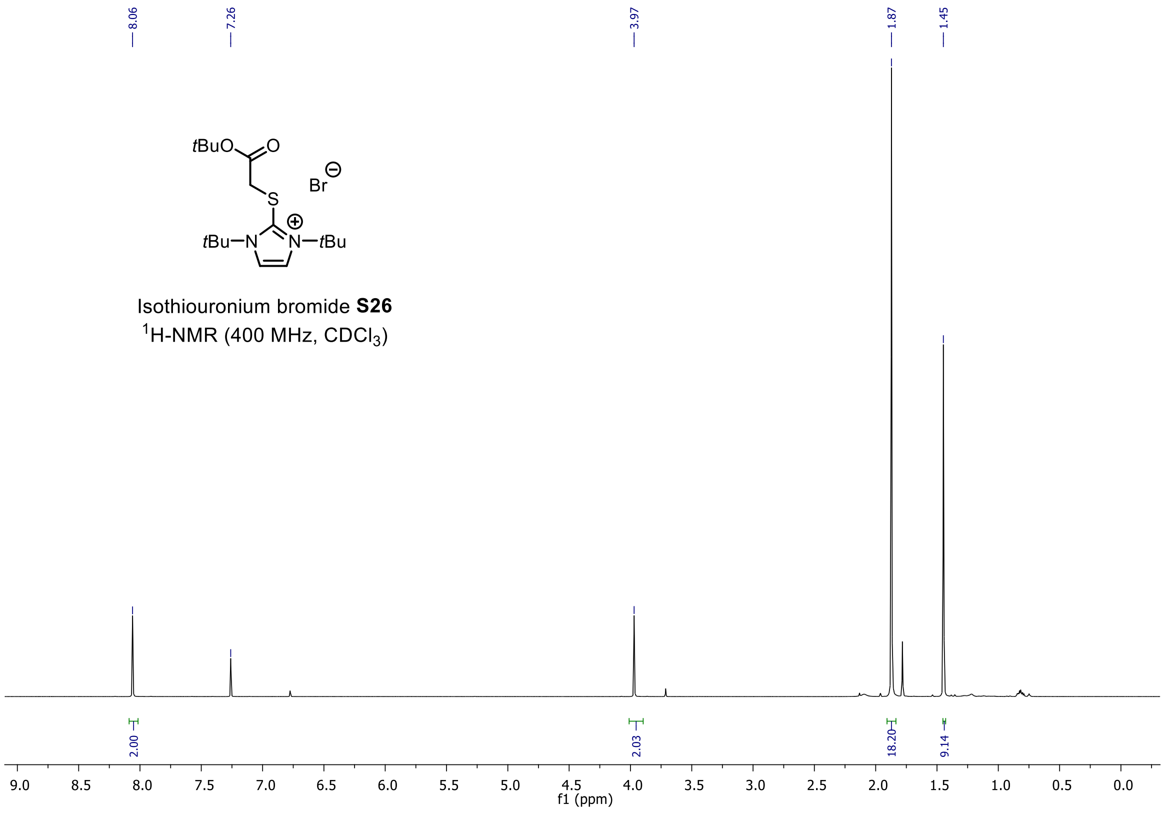
8.06
7.26
3.97
1.87
1.45
tBuO
O
S
Br
tBu–N
N–tBu
Isothiouronium bromide **S26**
^{1}H-NMR (400 MHz, $CDCl_3$)
2.00
2.03
18.20
9.14
9.0 8.5 8.0 7.5 7.0 6.5 6.0 5.5 5.0 4.5 4.0 3.5 3.0 2.5 2.0 1.5 1.0 0.5 0.0
f1 (ppm)

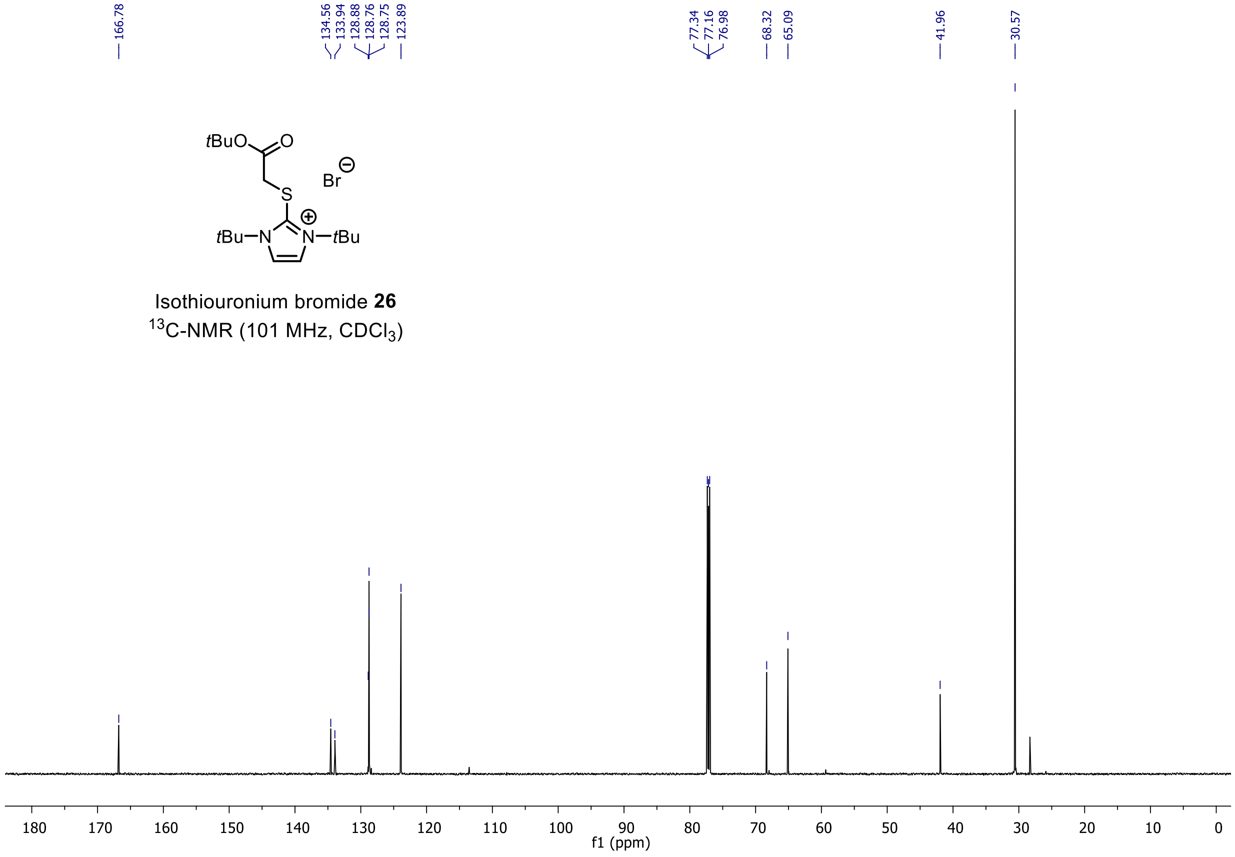
166.78
134.56
133.94
128.88
128.76
128.75
123.89
77.34
77.16
76.98
68.32
65.09
41.96
30.57
tBuO
O
S
Br
tBu–N
N–tBu
Isothiouronium bromide **26**
^{13}C-NMR (101 MHz, $CDCl_3$)
180 170 160 150 140 130 120 110 100 90 80 70 60 50 40 30 20 10 0
f1 (ppm)

Isothiouronium bromide **S1**
^{1}H-NMR (400 MHz, DMSO-d_6)

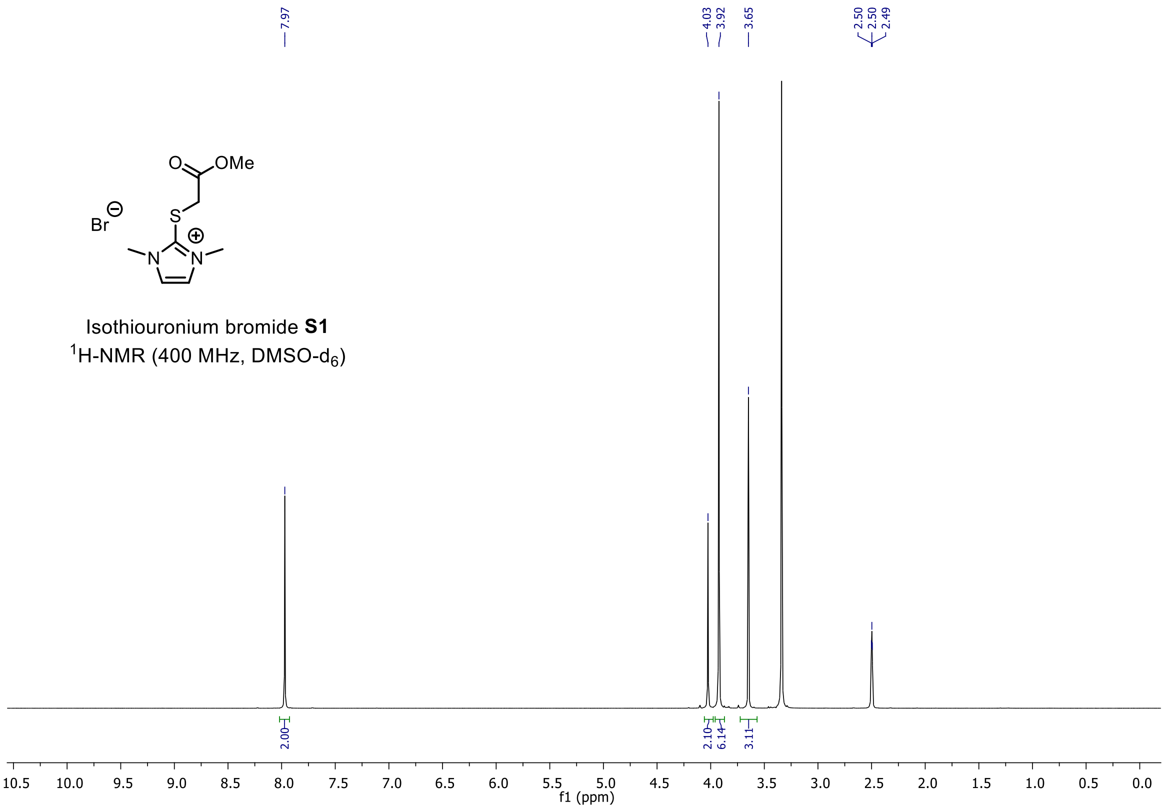

Isothiouronium bromide **S1**
^{13}C-NMR (400 MHz, DMSO-d_6)

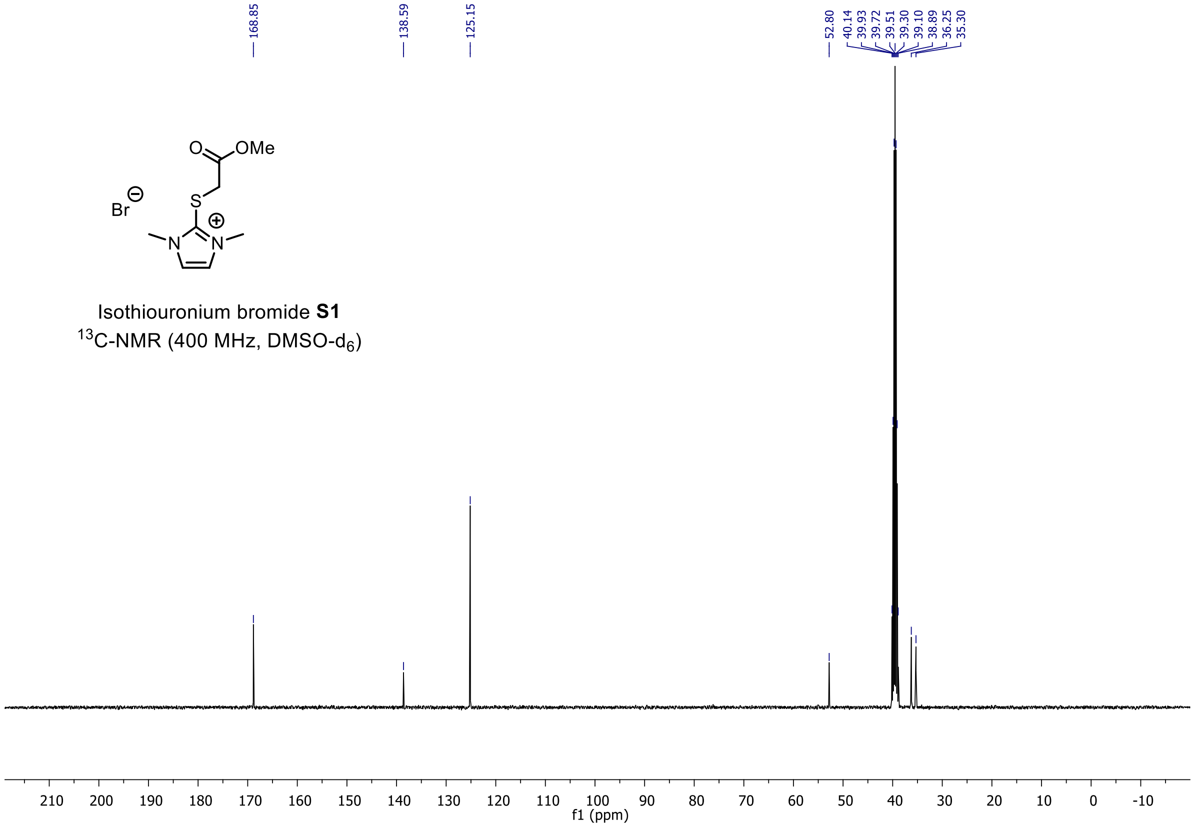

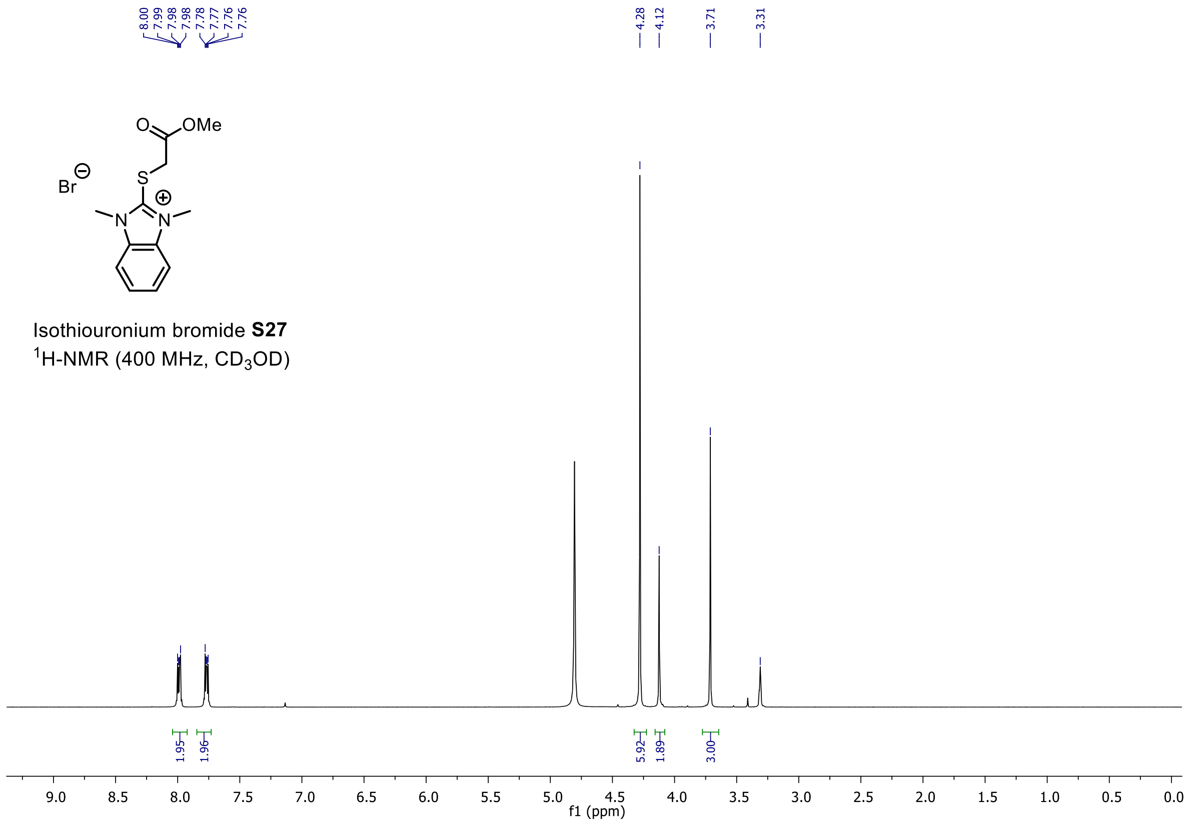

Isothiouronium bromide **S27**
^{1}H-NMR (400 MHz, CD_3OD)

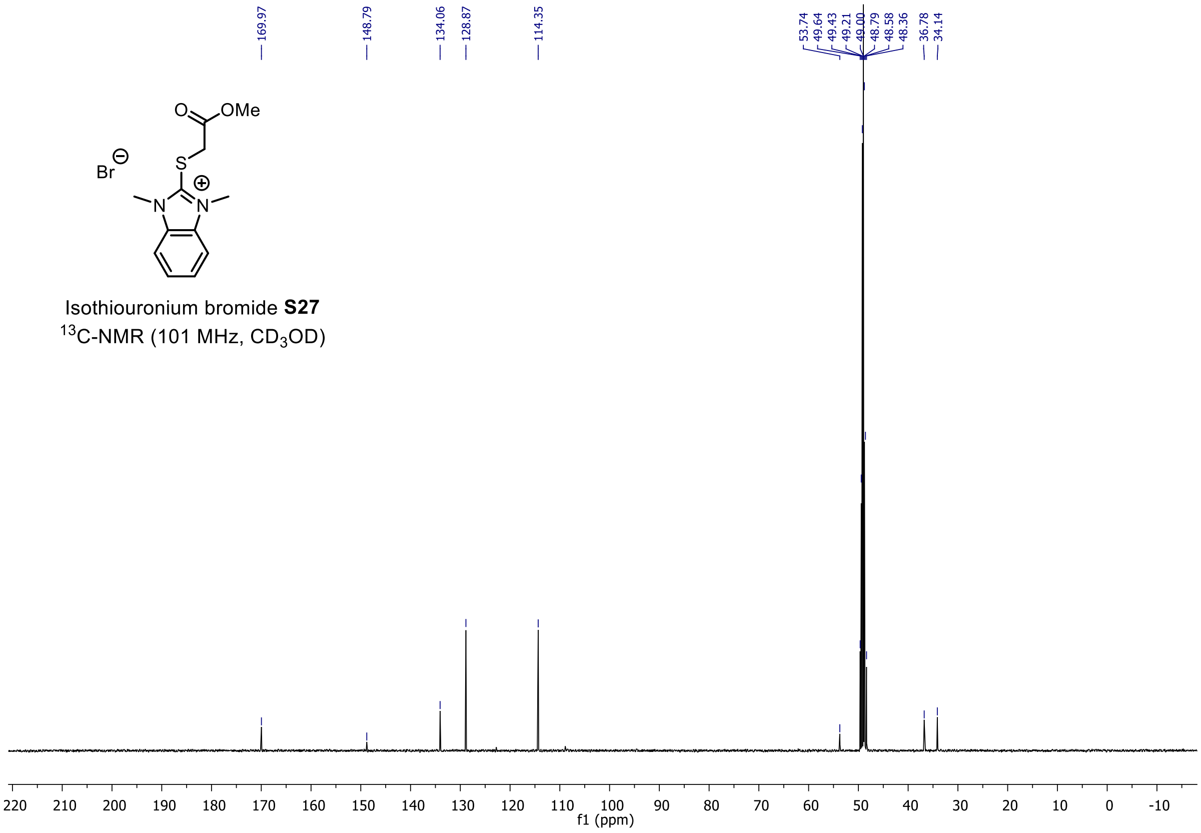

Isothiouronium bromide **S27**
^{13}C-NMR (101 MHz, CD_3OD)

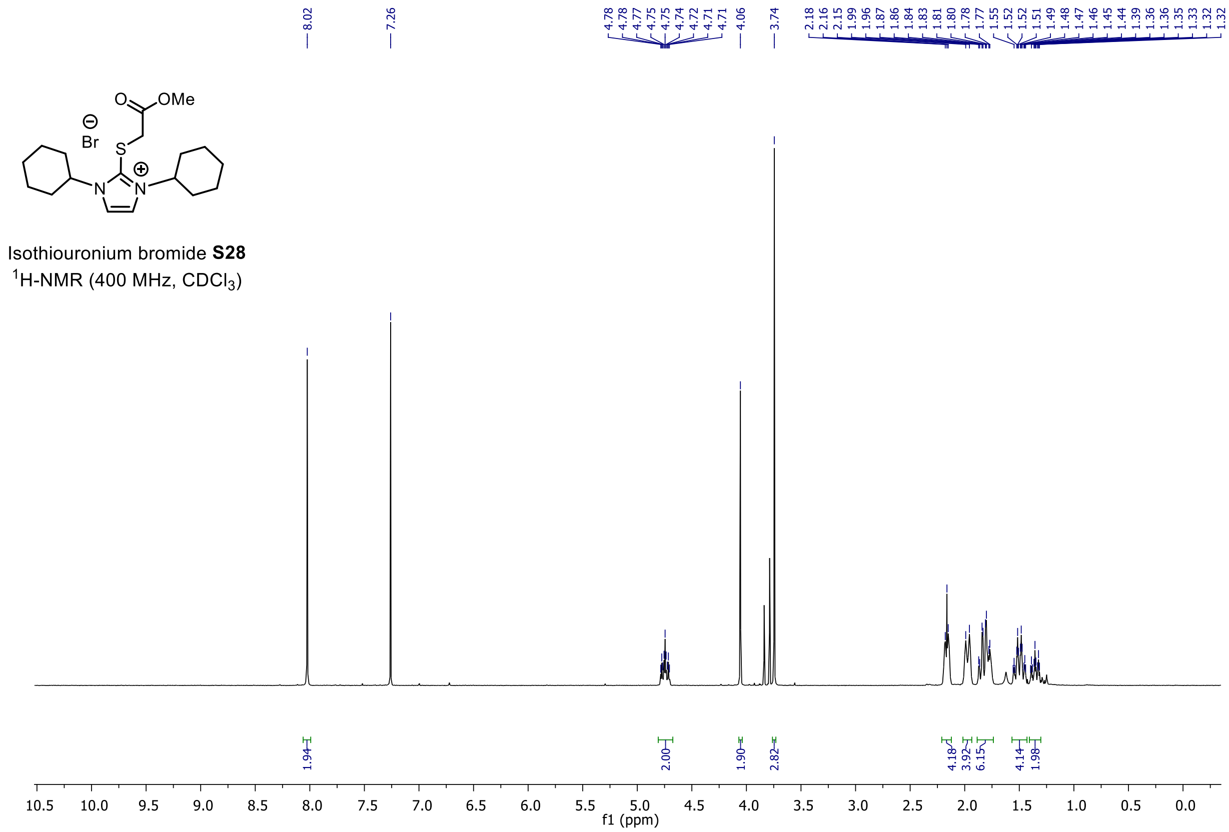

Isothiouronium bromide **S28**
^{1}H-NMR (400 MHz, $CDCl_3$)

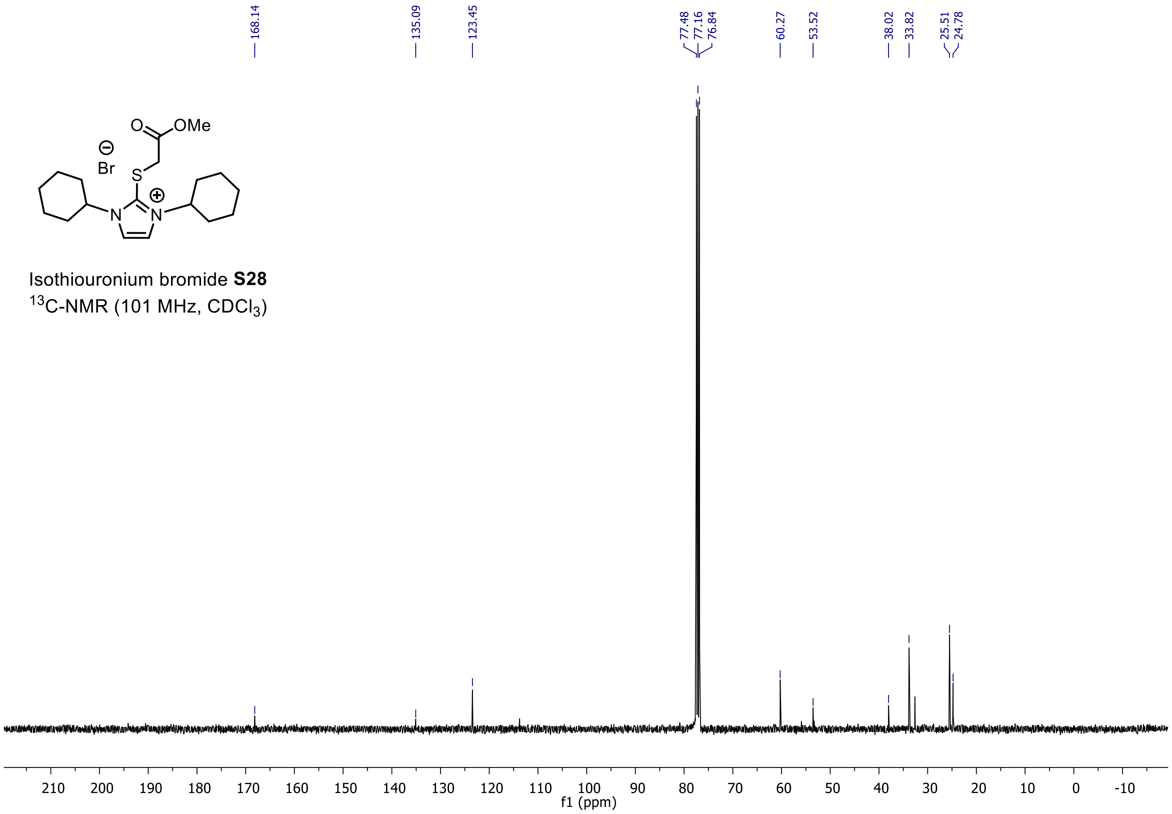

Isothiouronium bromide **S28**
^{13}C-NMR (101 MHz, $CDCl_3$)

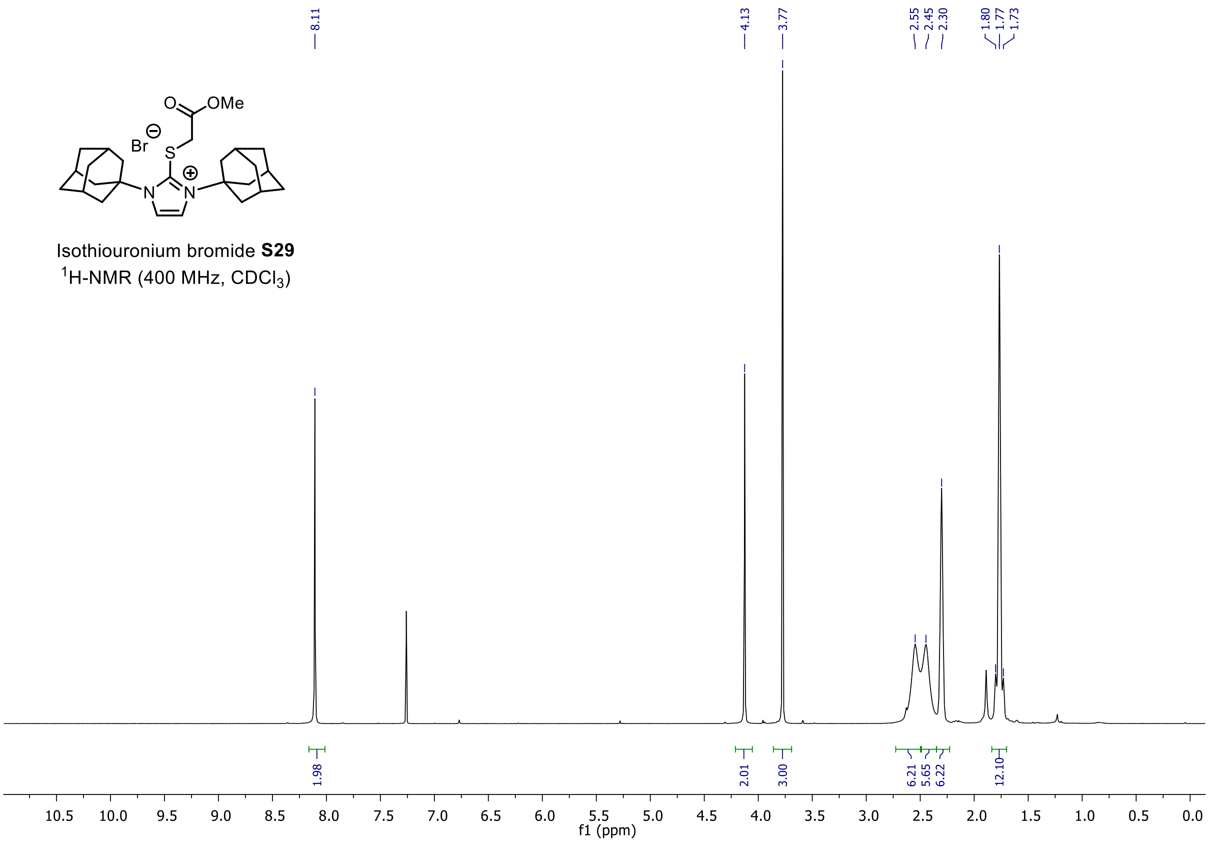
Isothiouronium bromide **S29**
^{1}H-NMR (400 MHz, $CDCl_3$)
8.11
4.13
3.77
2.55
2.45
2.30
1.80
1.77
1.73
1.98
2.01
3.00
6.21
5.65
6.22
12.10
f1 (ppm)

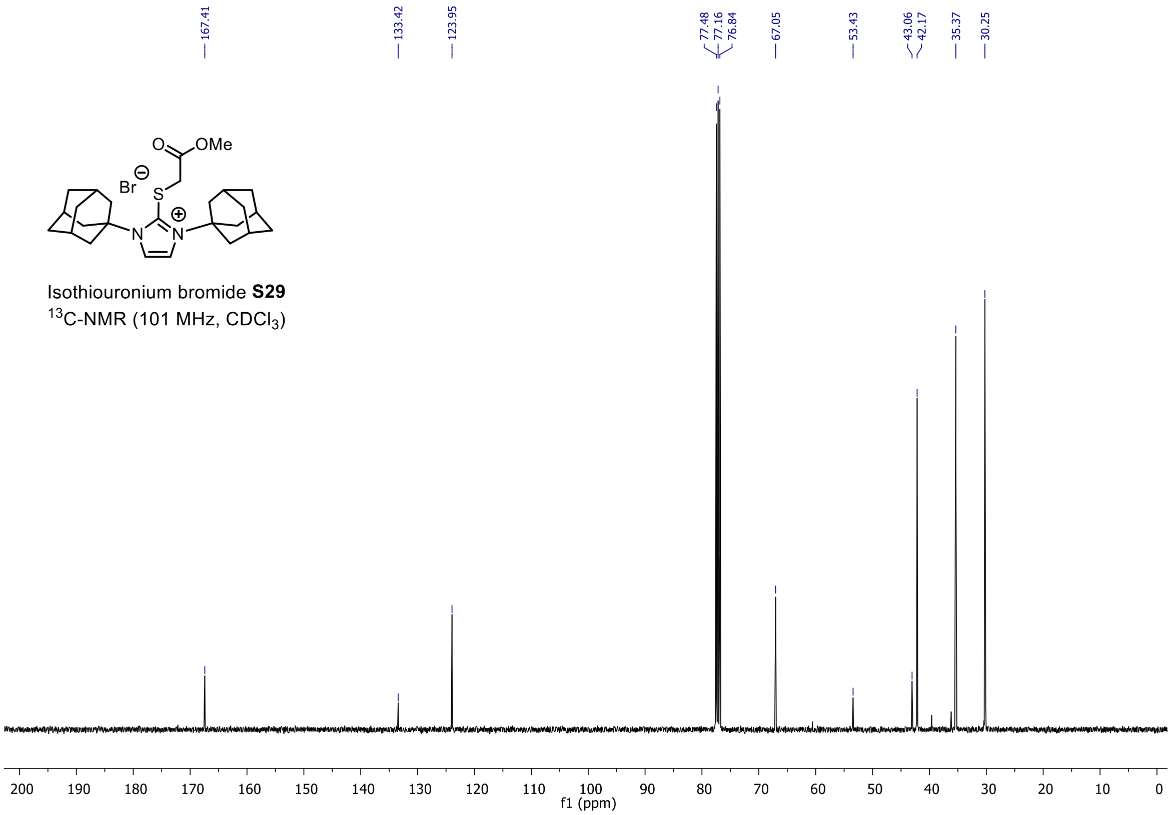
Isothiouronium bromide **S29**
^{13}C-NMR (101 MHz, $CDCl_3$)
167.41
133.42
123.95
77.48
77.16
76.84
67.05
53.43
43.06
42.17
35.37
30.25
f1 (ppm)

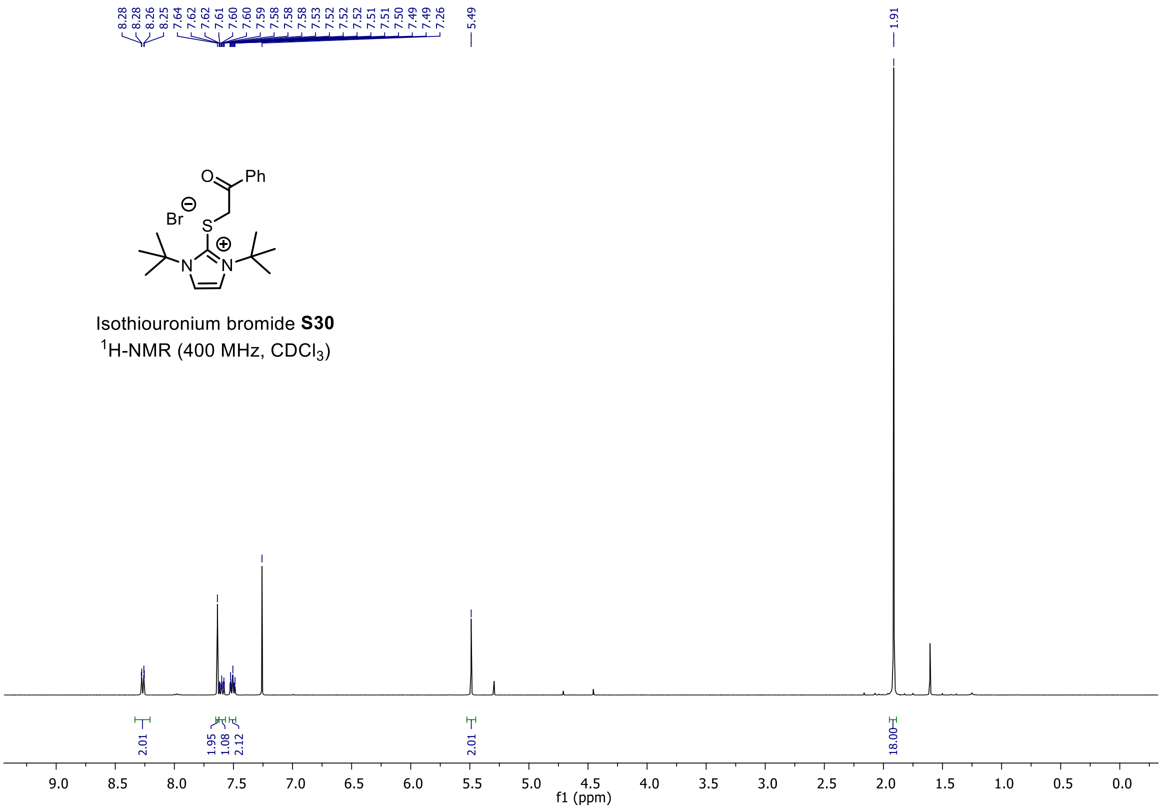

Isothiouronium bromide **S30**
^{1}H-NMR (400 MHz, $CDCl_3$)

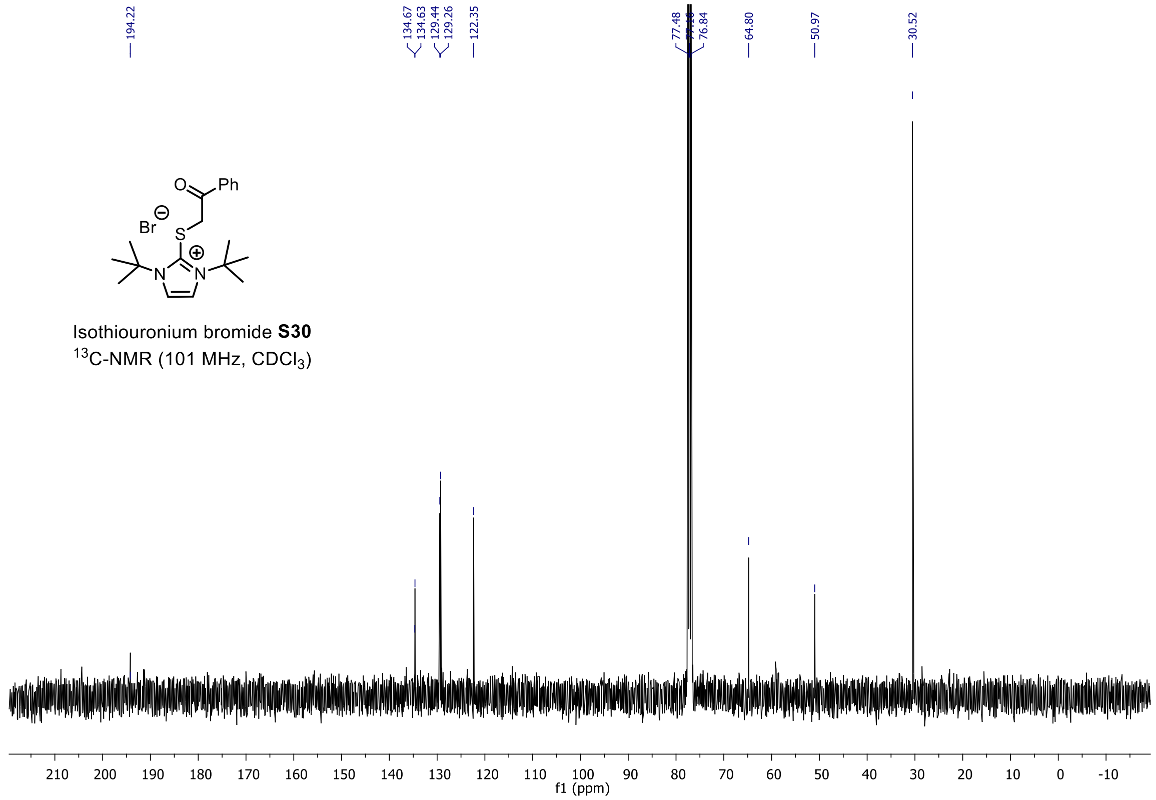

Isothiouronium bromide **S30**
^{13}C-NMR (101 MHz, $CDCl_3$)

Isothiouronium bromide **S31**
^{1}H-NMR (400 MHz, DMSO-d_6)

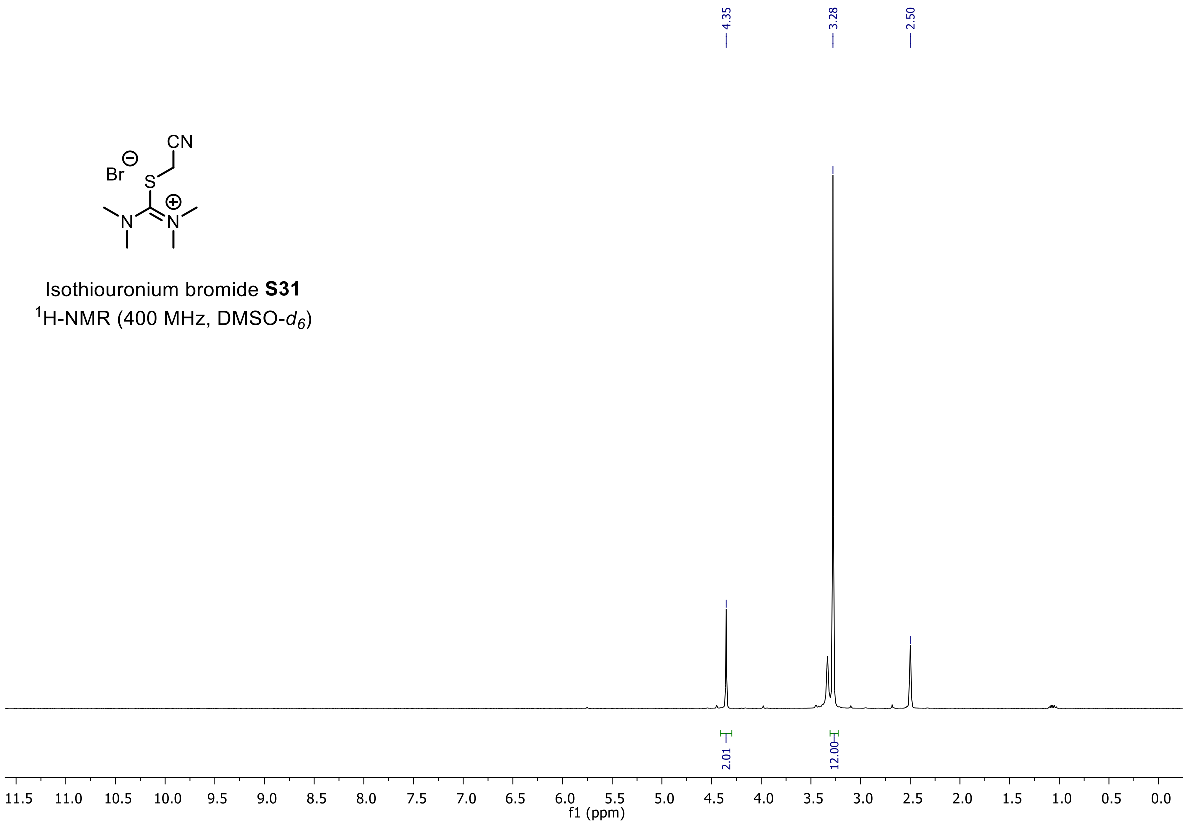

Isothiouronium bromide **S31**
^{13}C-NMR (101 MHz, DMSO-d_6)

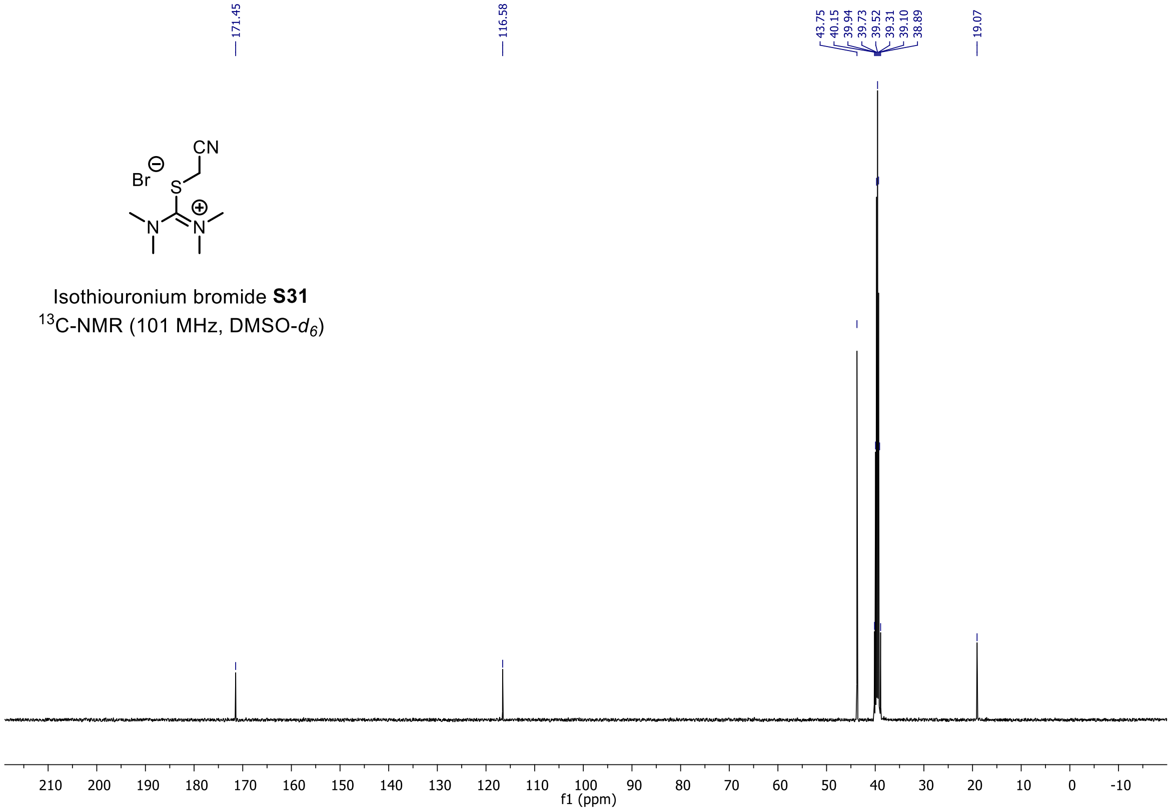

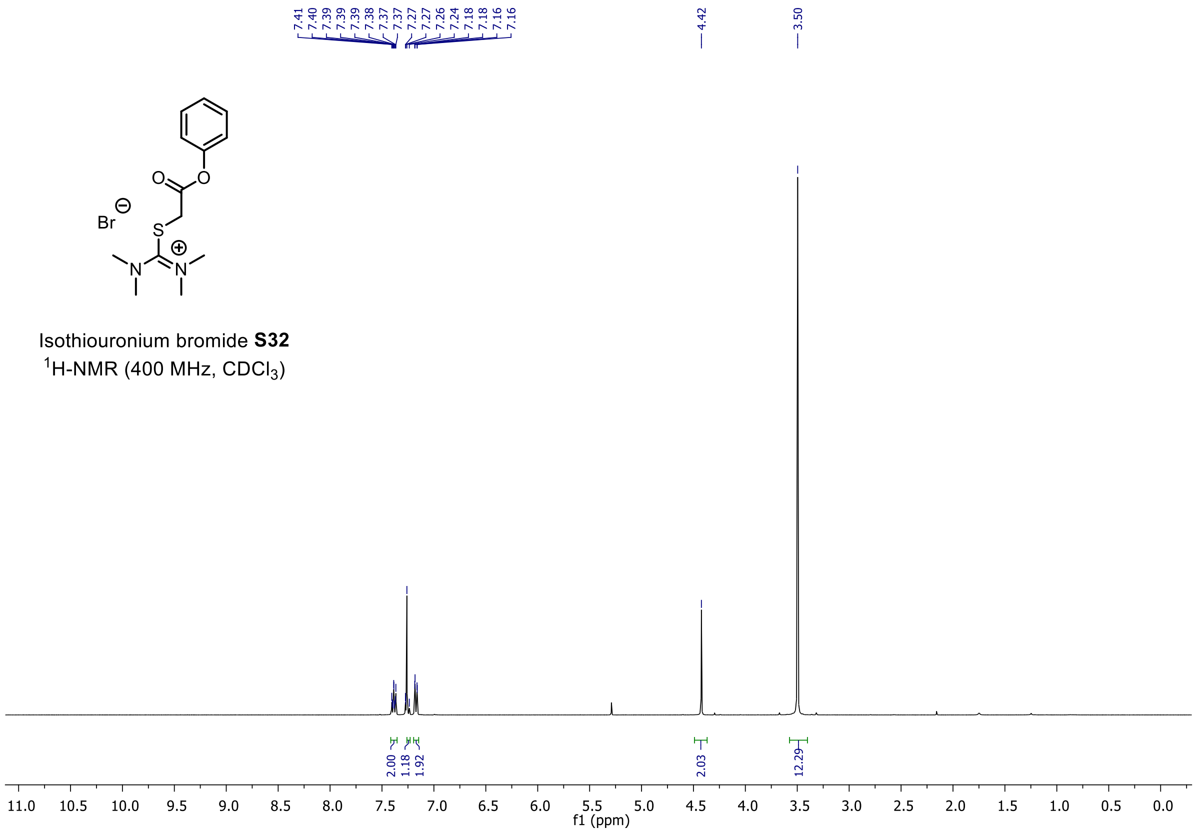

Isothiouronium bromide **S32**
^{1}H-NMR (400 MHz, $CDCl_3$)

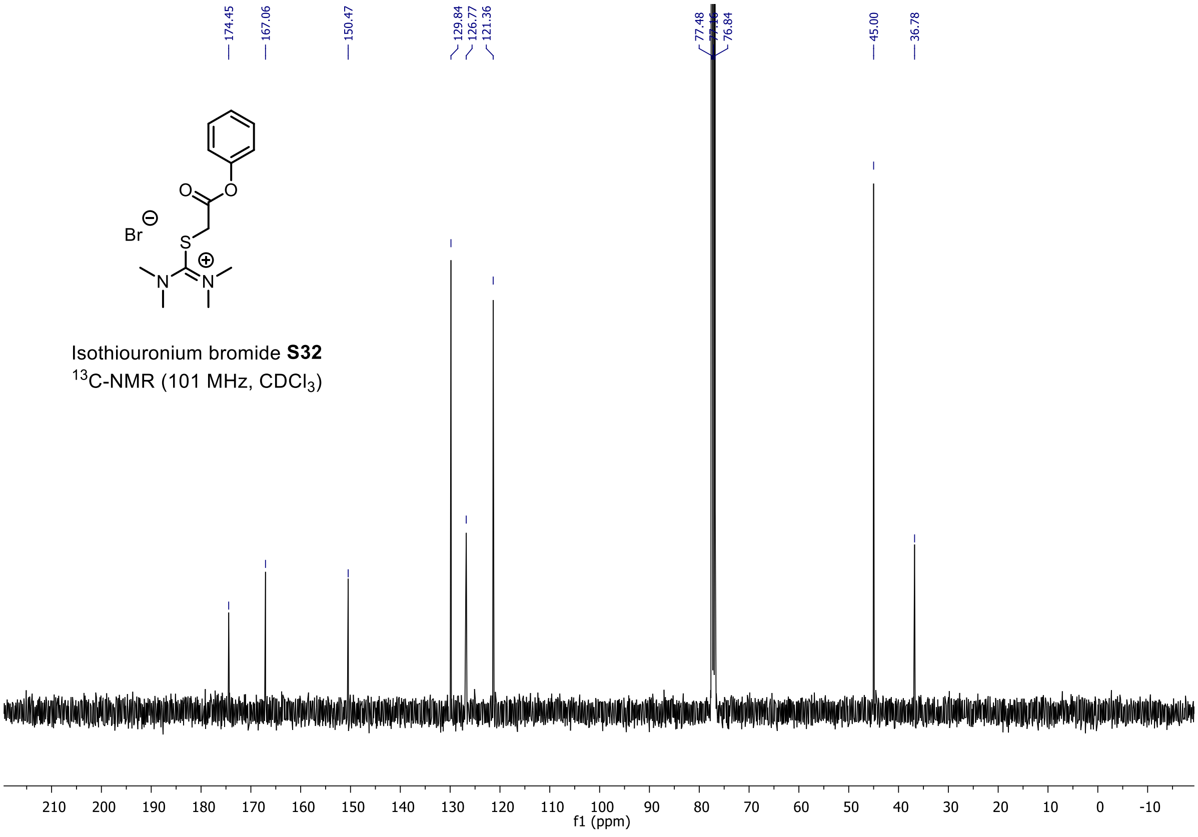

Isothiouronium bromide **S32**
^{13}C-NMR (101 MHz, $CDCl_3$)

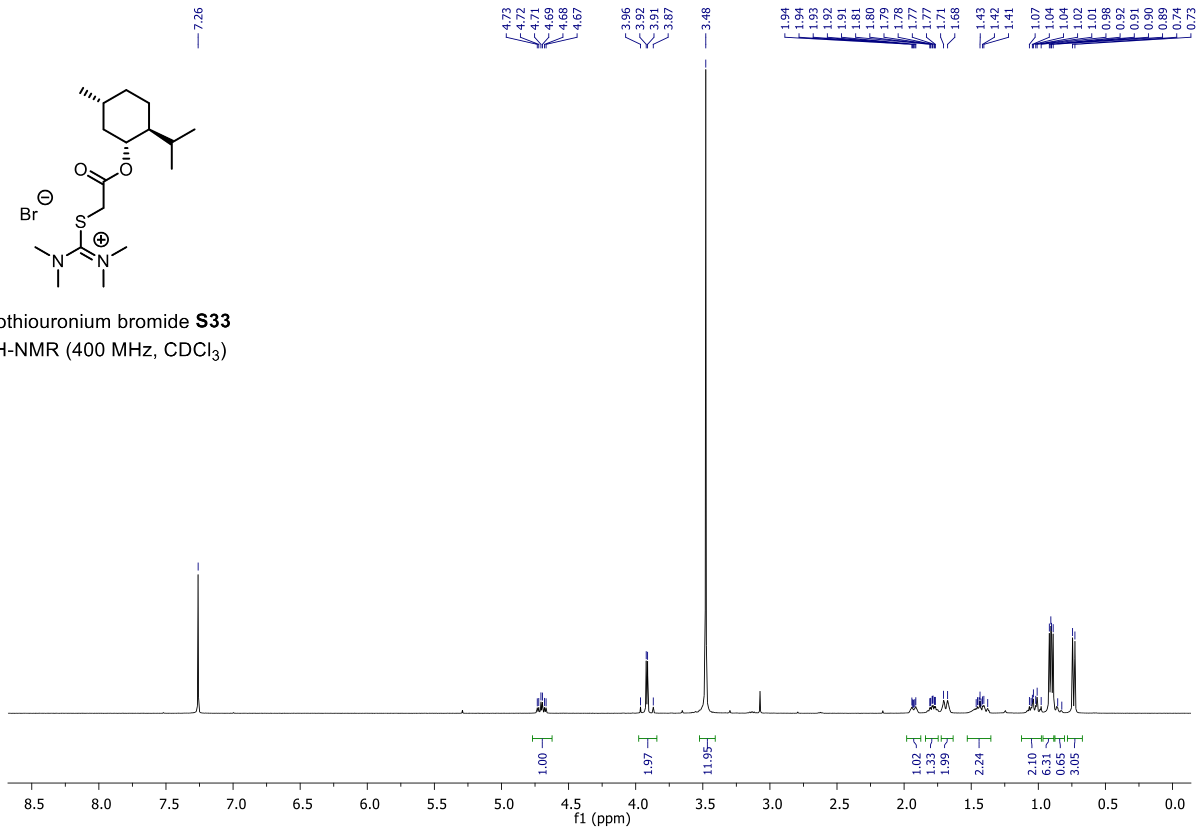

Isothiouronium bromide **S33**
^{1}H-NMR (400 MHz, $CDCl_3$)

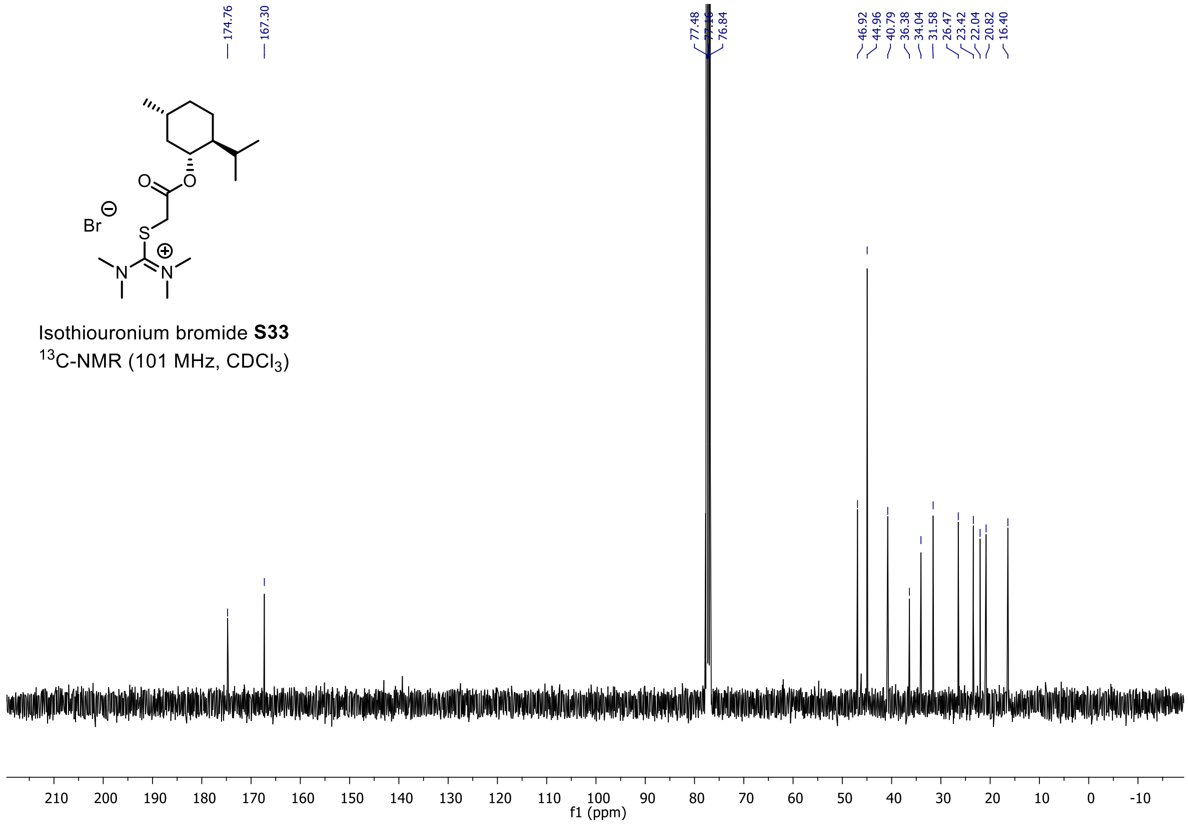

Isothiouronium bromide **S33**
^{13}C-NMR (101 MHz, $CDCl_3$)

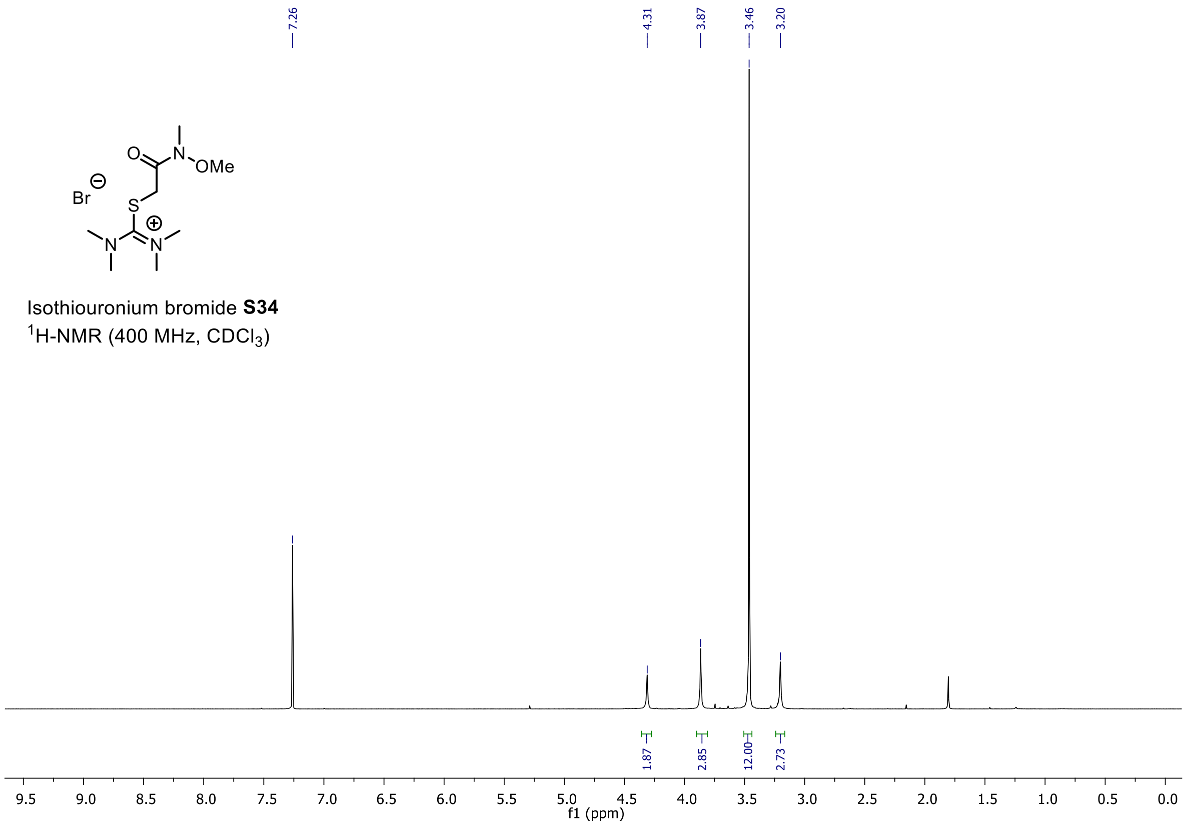

Isothiouronium bromide **S34**
^{1}H-NMR (400 MHz, $CDCl_3$)

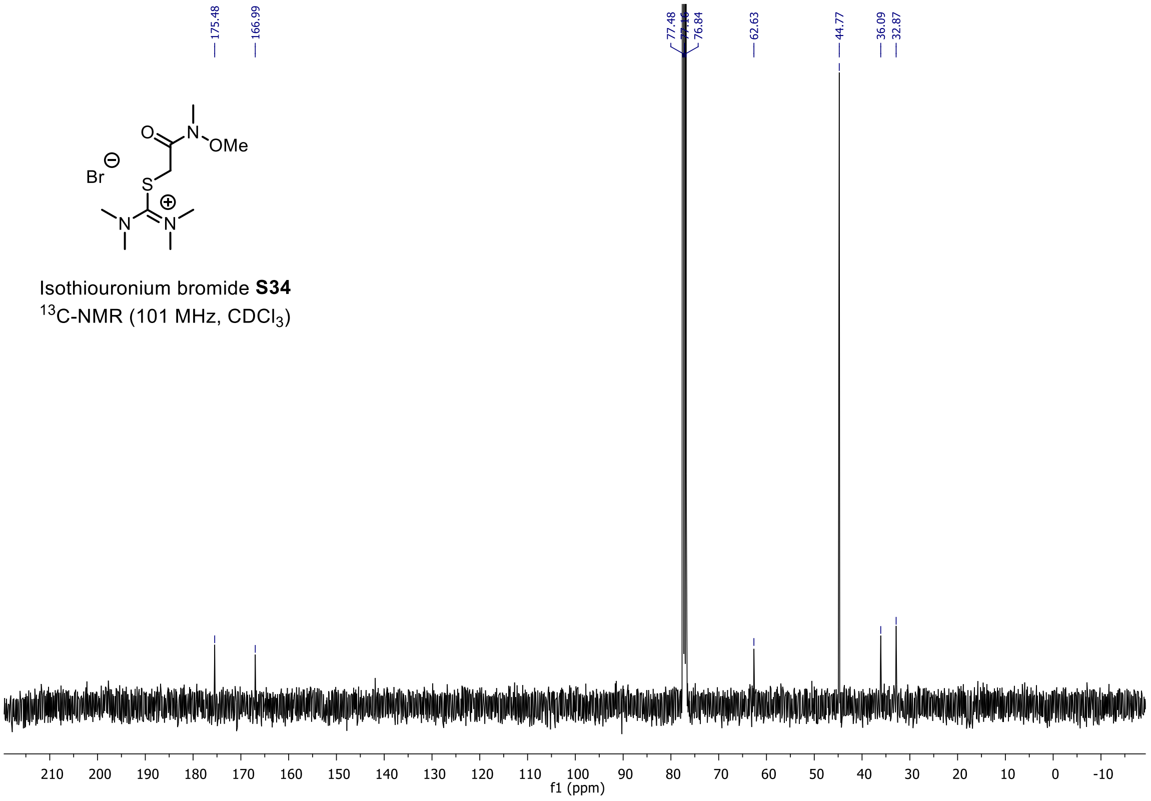

Isothiouronium bromide **S34**
^{13}C-NMR (101 MHz, $CDCl_3$)

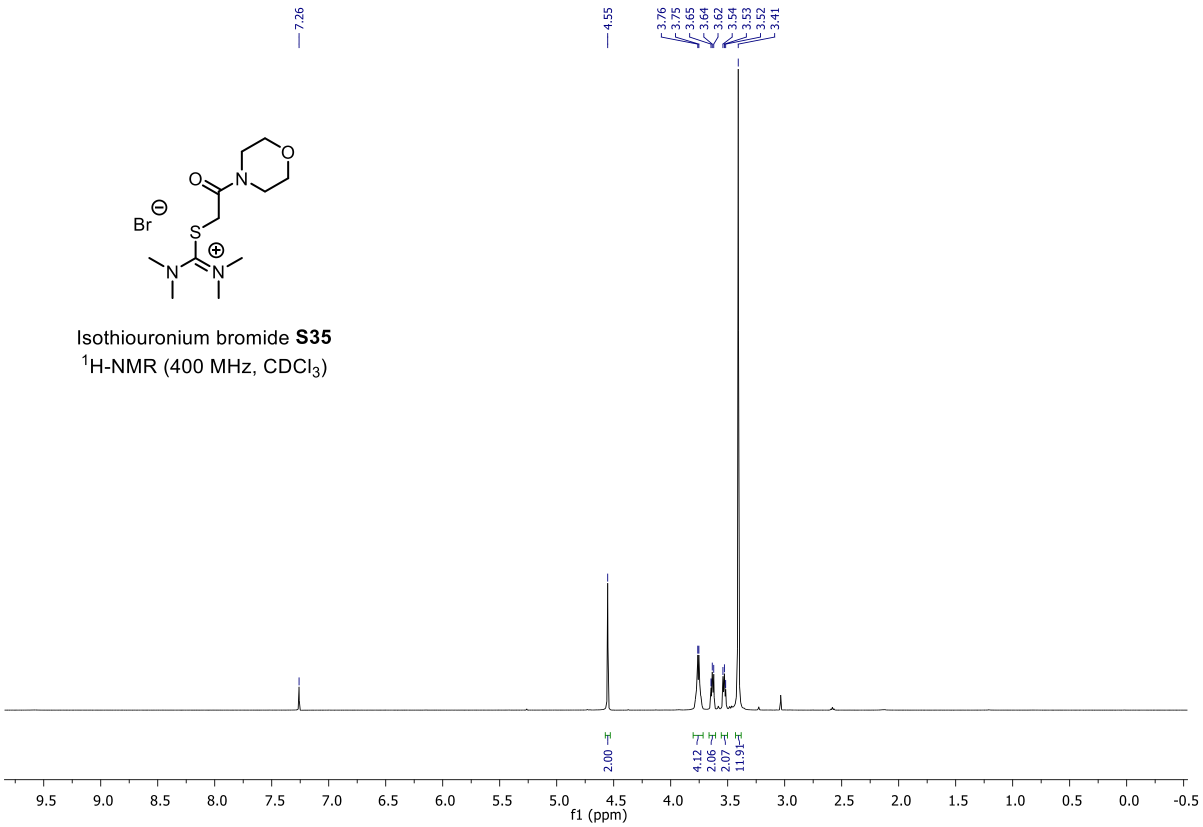

Isothiouronium bromide **S35**
^{1}H-NMR (400 MHz, $CDCl_3$)

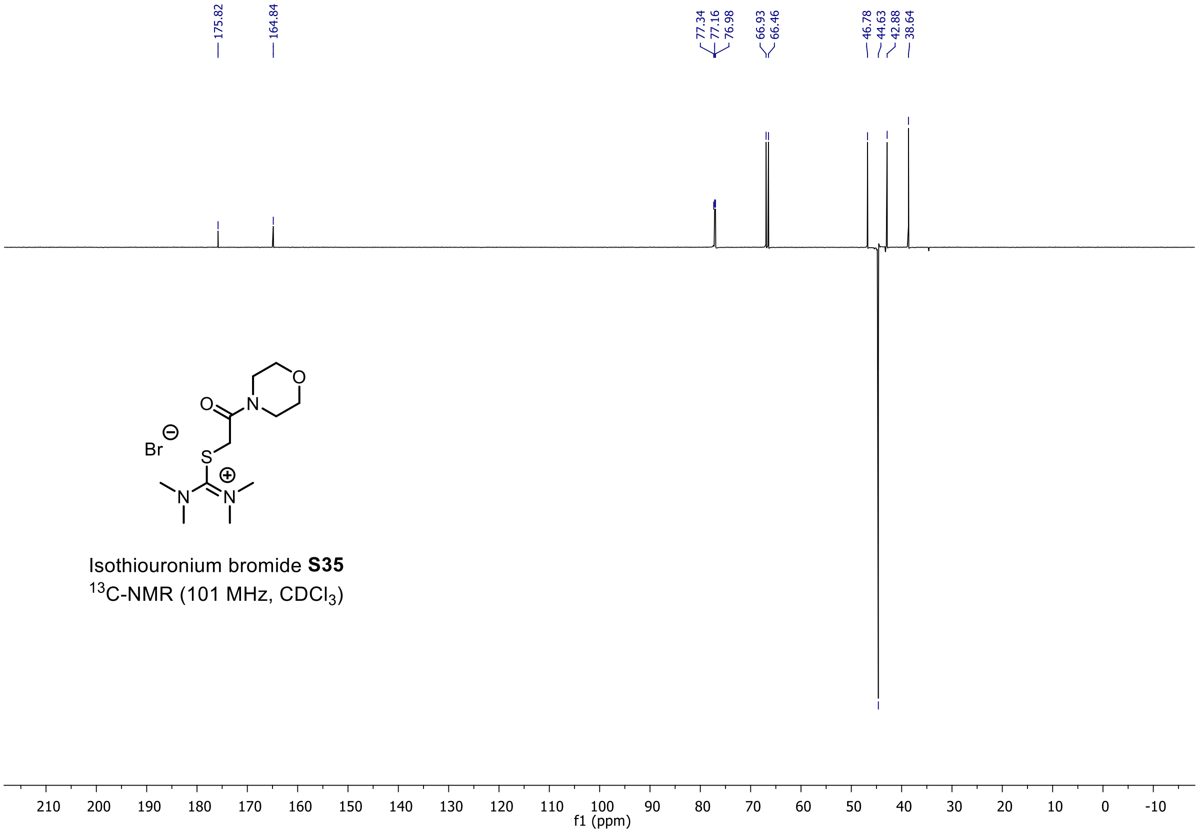

Isothiouronium bromide **S35**
^{13}C-NMR (101 MHz, $CDCl_3$)

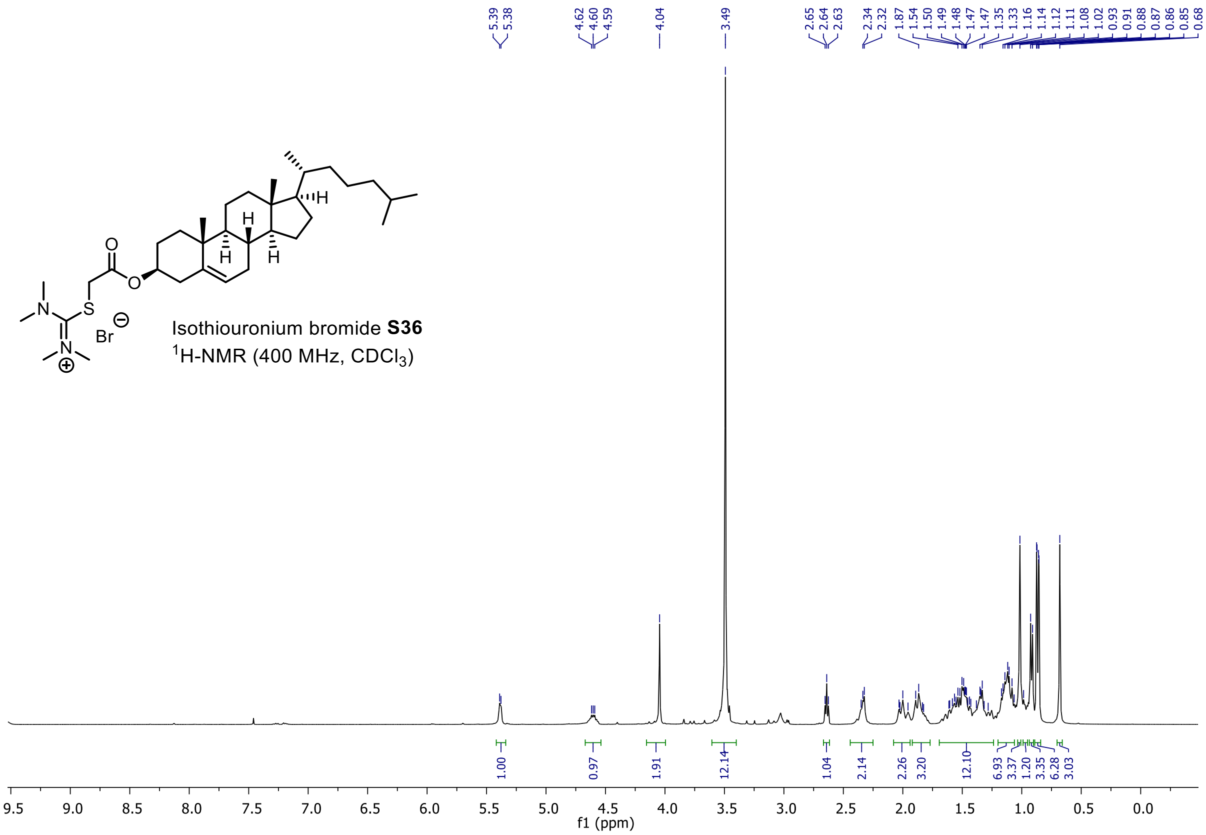

Isothiouronium bromide **S36**
^{1}H-NMR (400 MHz, $CDCl_3$)

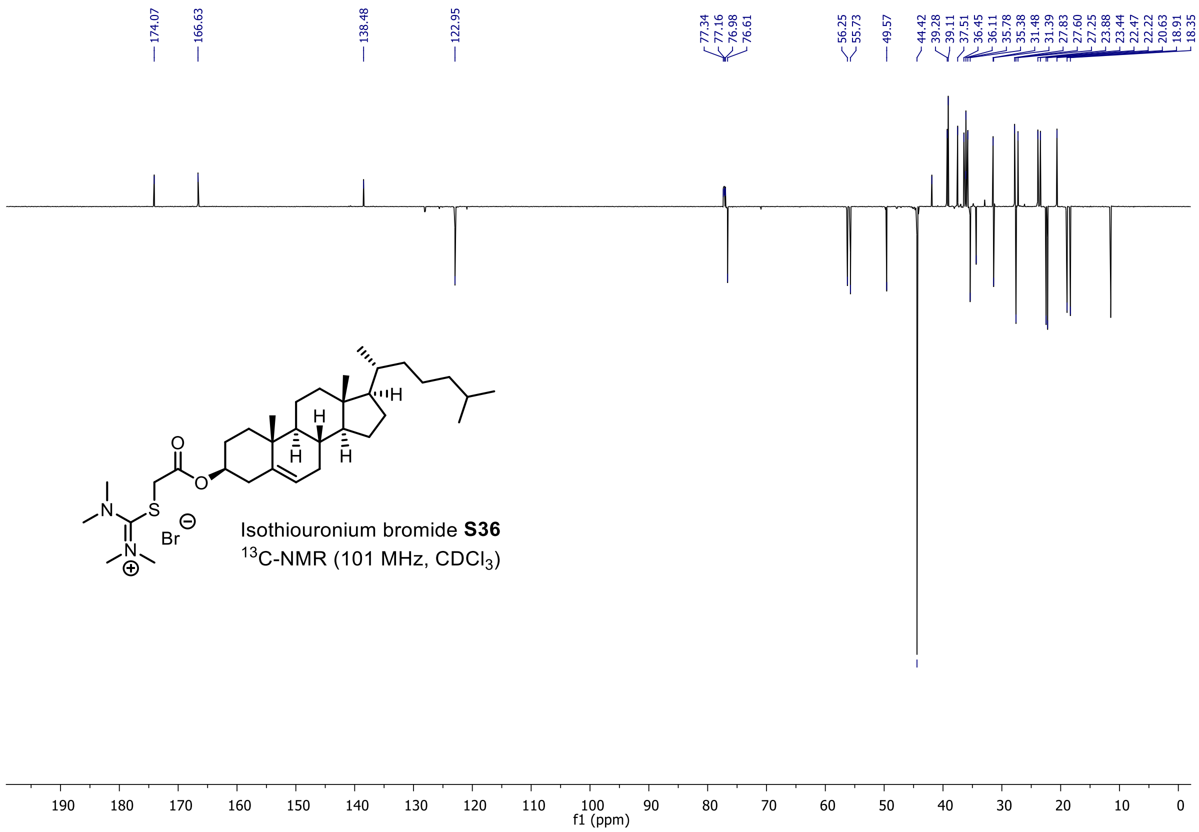

Isothiouronium bromide **S36**
^{13}C-NMR (101 MHz, $CDCl_3$)

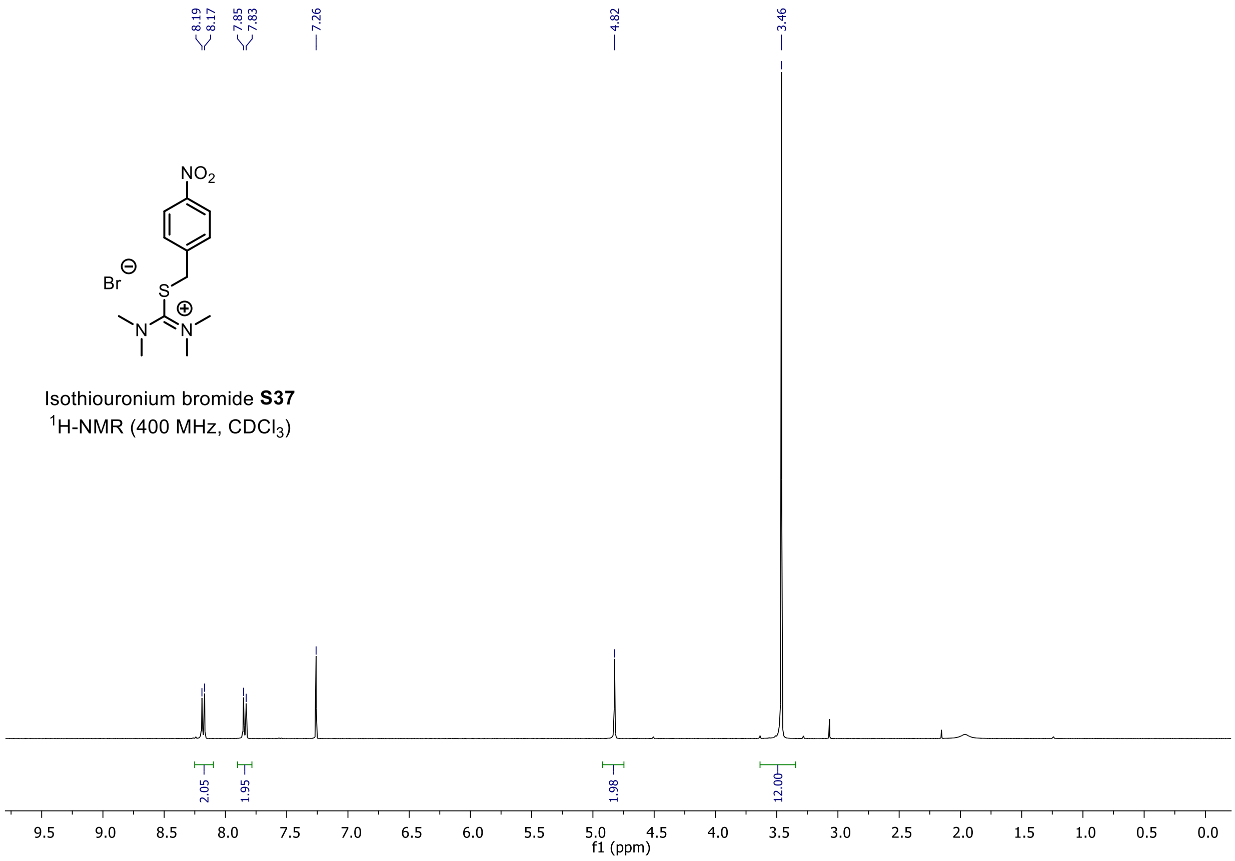

Isothiouronium bromide **S37**
^{1}H-NMR (400 MHz, $CDCl_3$)

8.3 Thiouronium bistriflimides

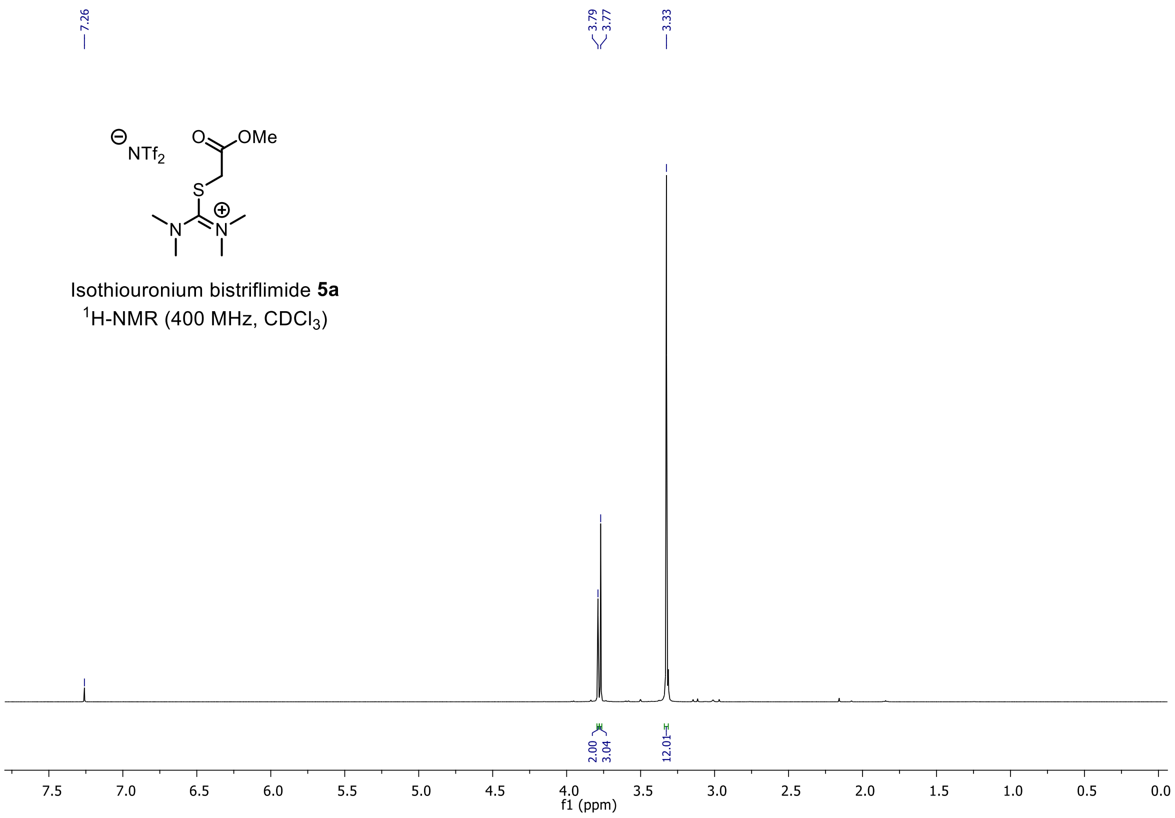

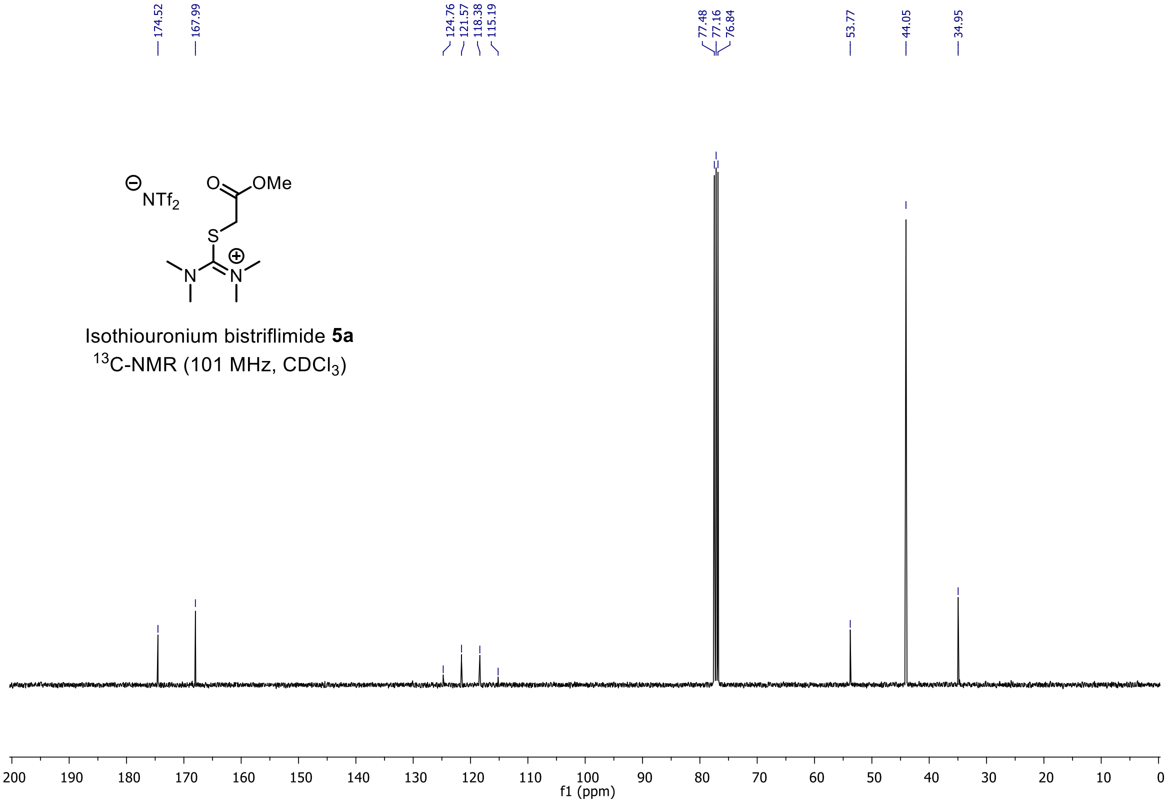

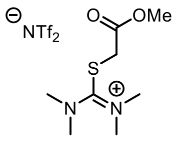

Isothiouronium bistriflimide **5a**
^{19}F-NMR (376 MHz, $CDCl_3$)

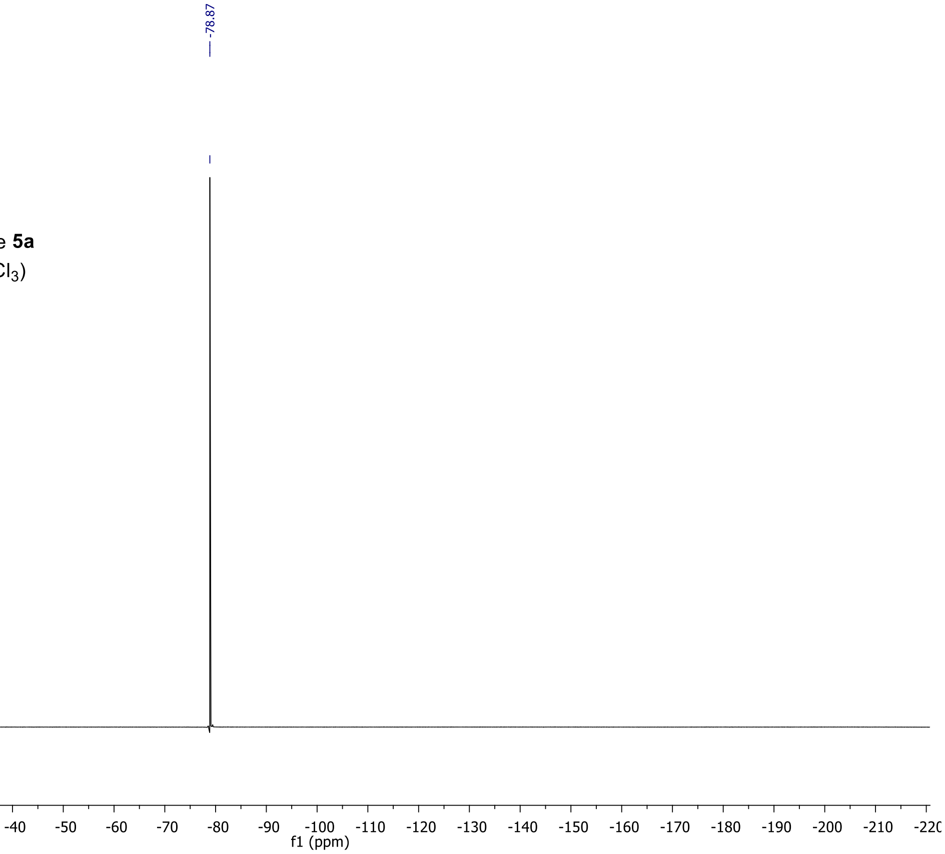

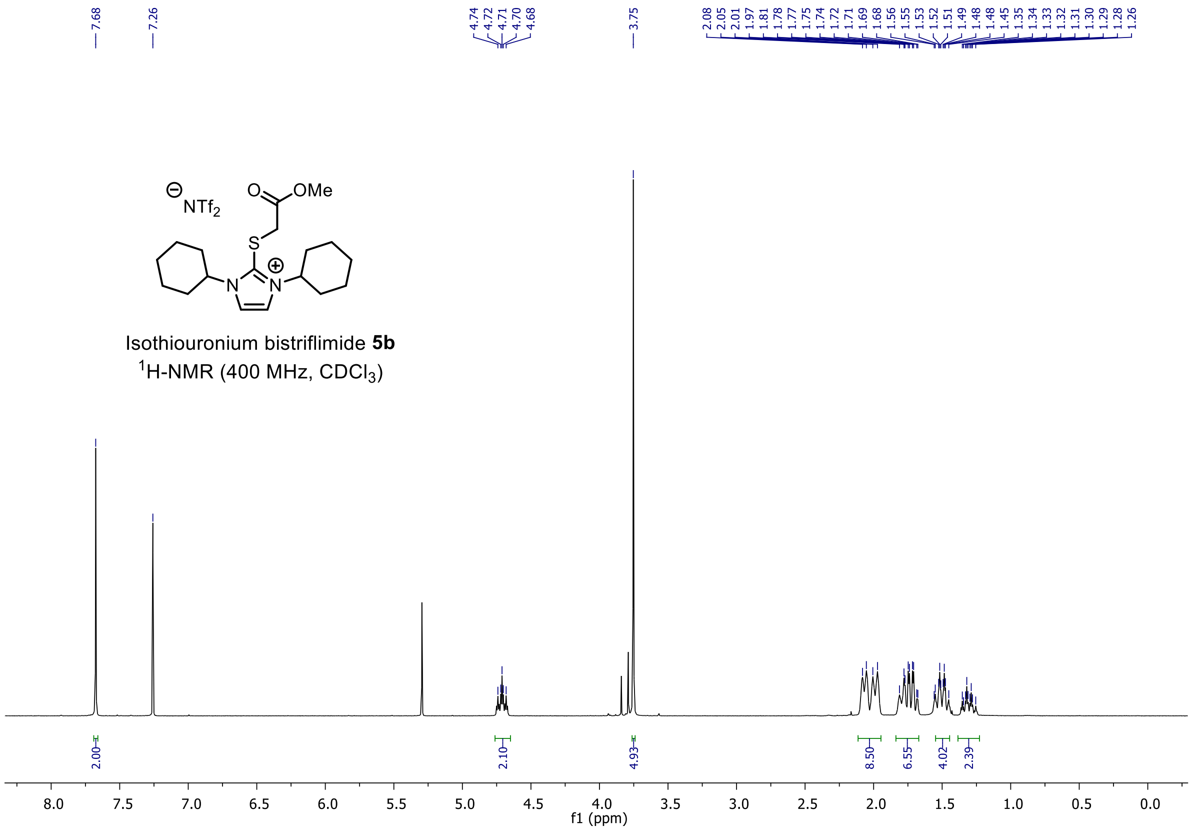
7.68
7.26
4.74
4.72
4.71
4.70
4.68
3.75
2.08
2.05
2.01
1.97
1.81
1.78
1.77
1.75
1.74
1.72
1.71
1.69
1.68
1.56
1.55
1.53
1.52
1.51
1.49
1.48
1.48
1.45
1.35
1.34
1.33
1.32
1.31
1.30
1.29
1.28
1.26
NTf2
O
OMe
S
N
N
Isothiouronium bistriflimide **5b**
^{1}H-NMR (400 MHz, $CDCl_3$)
2.00
2.10
4.93
8.50
6.55
4.02
2.39
8.0 7.5 7.0 6.5 6.0 5.5 5.0 4.5 4.0 3.5 3.0 2.5 2.0 1.5 1.0 0.5 0.0
f1 (ppm)

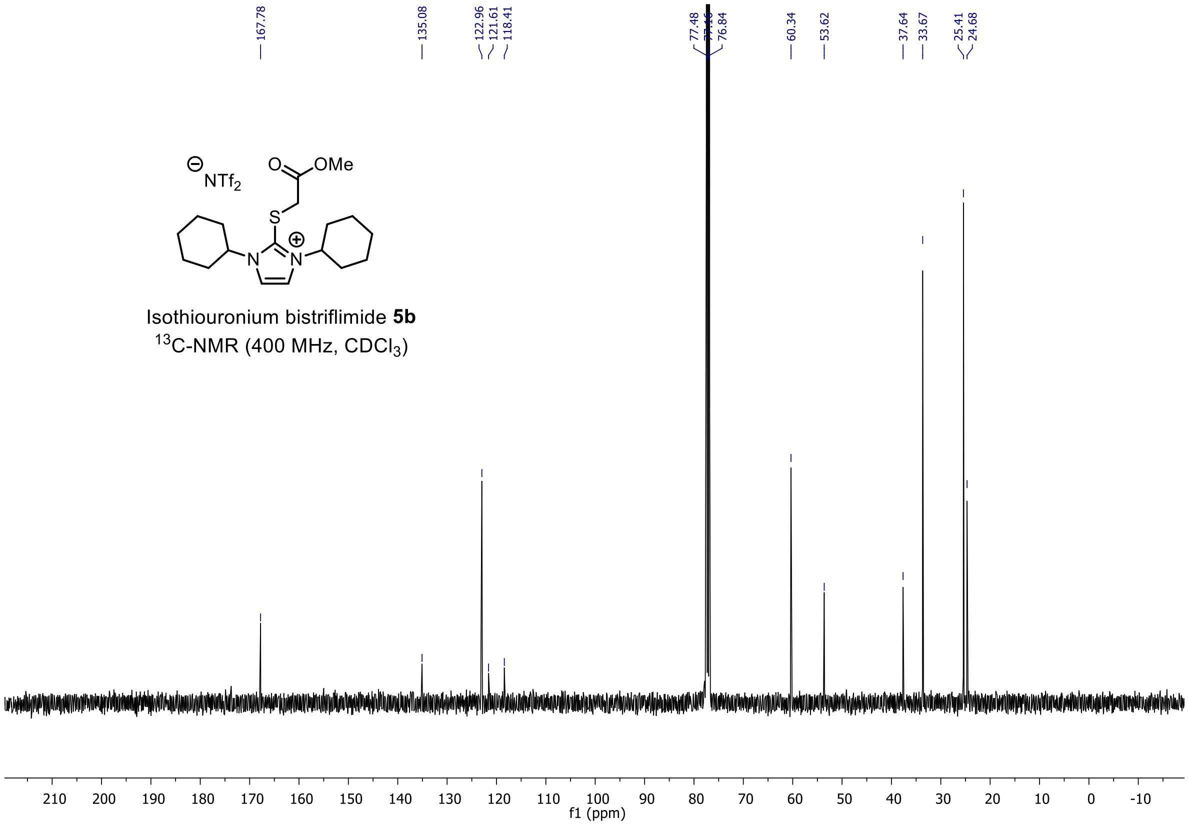
167.78
135.08
122.96
121.61
118.41
77.48
76.84
60.34
53.62
37.64
33.67
25.41
24.68
NTf2
O
OMe
S
N
N
Isothiouronium bistriflimide **5b**
^{13}C-NMR (400 MHz, $CDCl_3$)
210 200 190 180 170 160 150 140 130 120 110 100 90 80 70 60 50 40 30 20 10 0 -10
f1 (ppm)

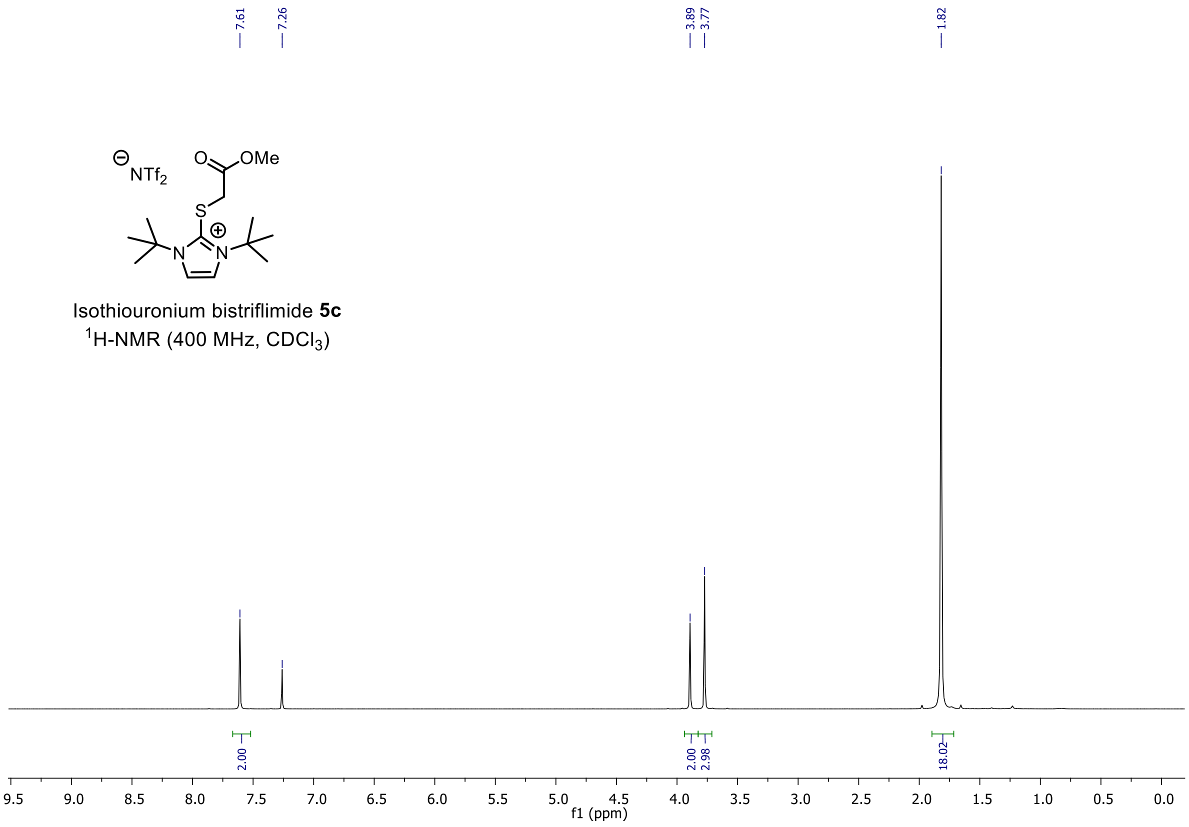
7.61
7.26
3.89
3.77
1.82
NTf2
O
OMe
S
N
N
Isothiouronium bistriflimide **5c**
^{1}H-NMR (400 MHz, $CDCl_3$)
2.00
2.00
2.98
18.02
9.5 9.0 8.5 8.0 7.5 7.0 6.5 6.0 5.5 5.0 4.5 4.0 3.5 3.0 2.5 2.0 1.5 1.0 0.5 0.0
f1 (ppm)

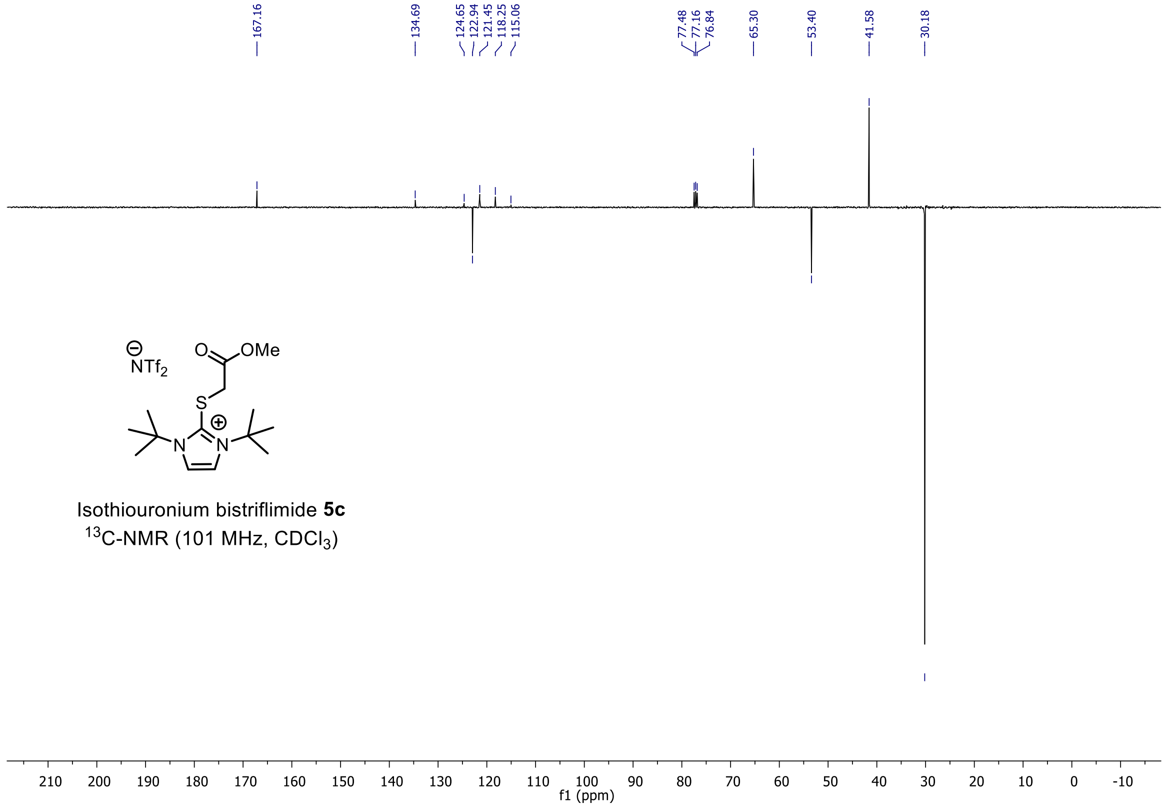
167.16
134.69
124.65
122.94
121.45
118.25
115.06
77.48
77.16
76.84
65.30
53.40
41.58
30.18
NTf2
O
OMe
S
N
N
Isothiouronium bistriflimide **5c**
^{13}C-NMR (101 MHz, $CDCl_3$)
210 200 190 180 170 160 150 140 130 120 110 100 90 80 70 60 50 40 30 20 10 0 -10
f1 (ppm)

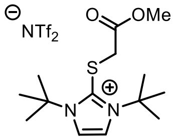

Isothiouronium bistriflimide **5c**
^{19}F-NMR (377 MHz, $CDCl_3$)

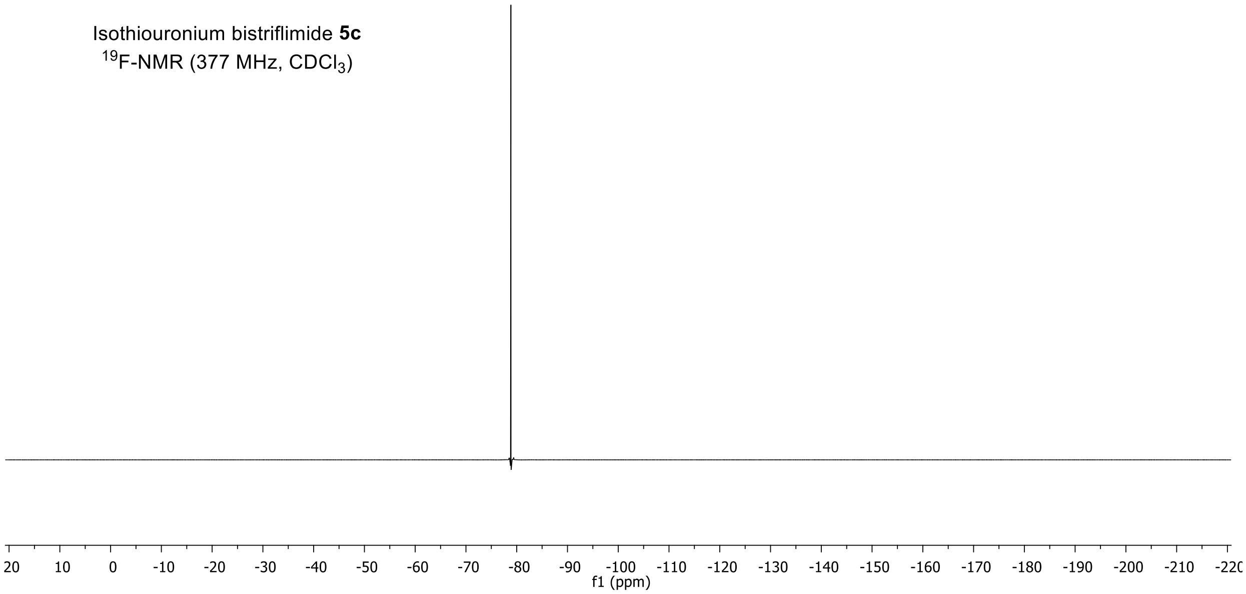

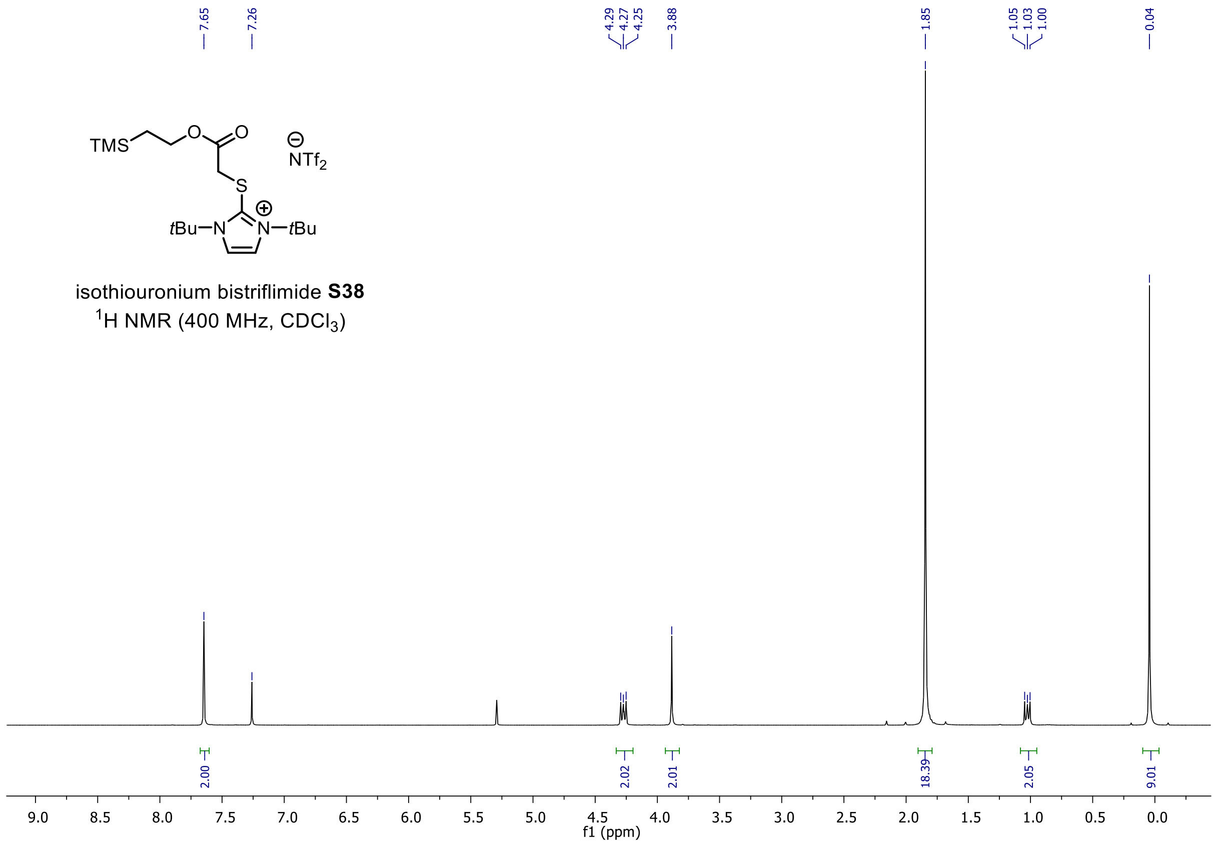
TMS
O
O
NTf2
S
tBu
N
N
tBu
isothiouronium bistriflimide S38
^{1}H NMR (400 MHz, CDCl$_3$)
7.65
7.26
4.29
4.27
4.25
3.88
1.85
1.05
1.03
1.00
0.04
2.00
2.02
2.01
18.39
2.05
9.01
f1 (ppm)

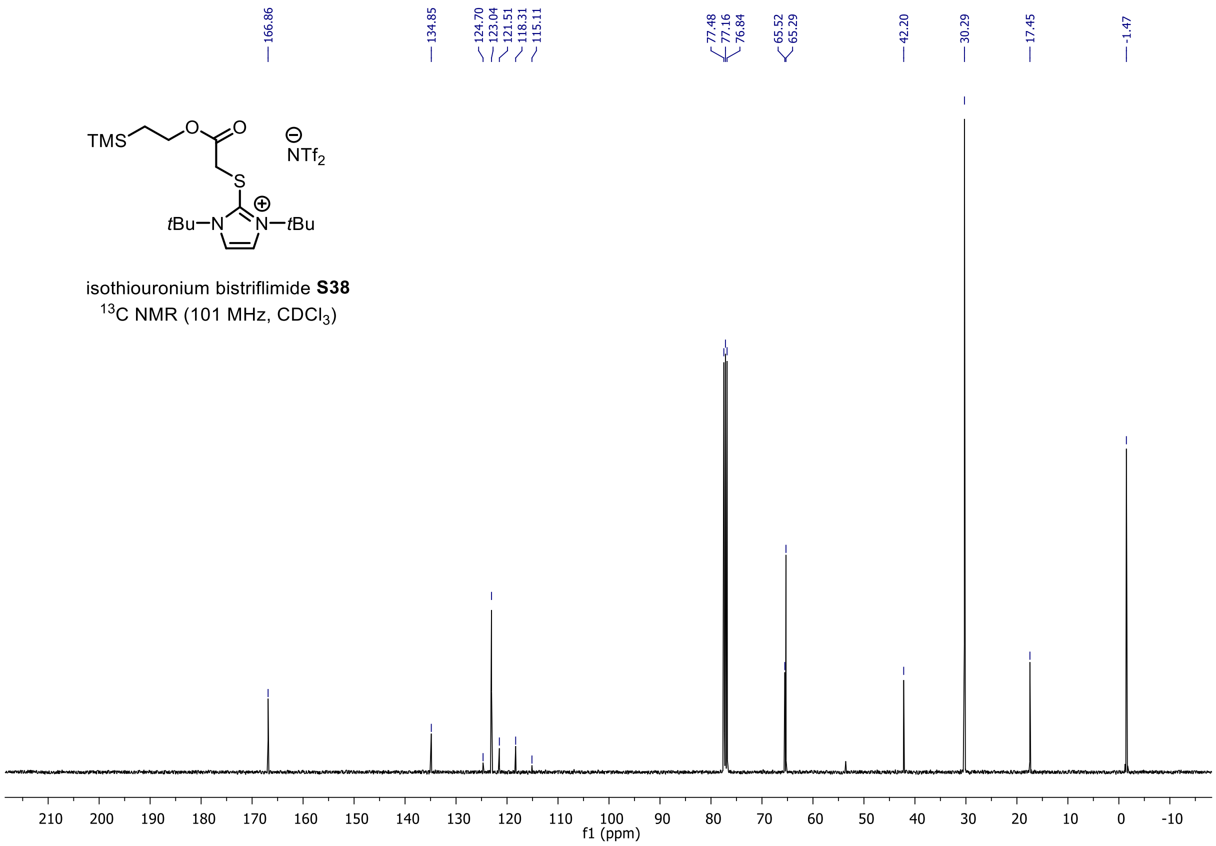
TMS
O
O
NTf2
S
tBu
N
N
tBu
isothiouronium bistriflimide S38
^{13}C NMR (101 MHz, CDCl$_3$)
166.86
134.85
124.70
123.04
121.51
118.31
115.11
77.48
77.16
76.84
65.52
65.29
42.20
30.29
17.45
-1.47
f1 (ppm)

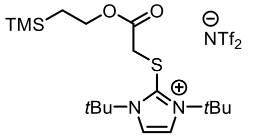

isothiouronium bistriflimide **S38**
^{19}F NMR (377 MHz, $CDCl_3$)

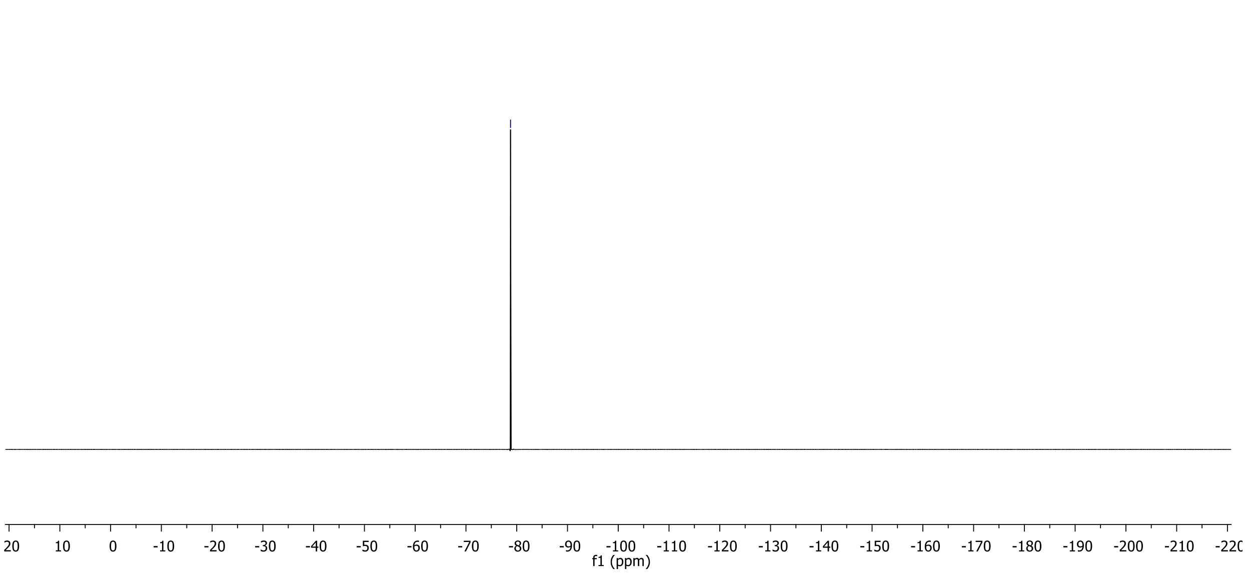

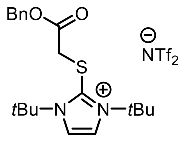

isothiouronium bistriflimide **S39**

^{1}H NMR (400 MHz, $CDCl_3$)

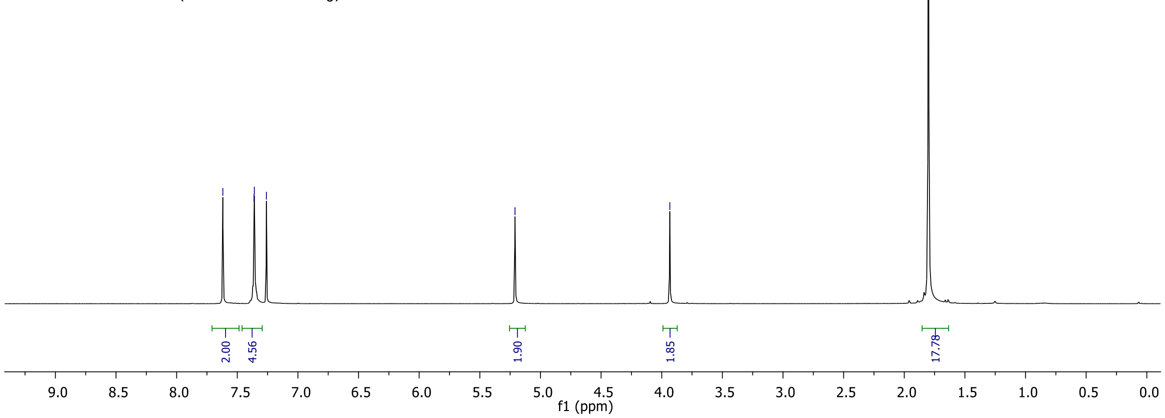

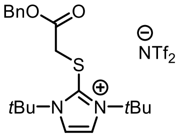

isothiouronium bistriflimide **S39**

^{13}C NMR (101 MHz, $CDCl_3$)

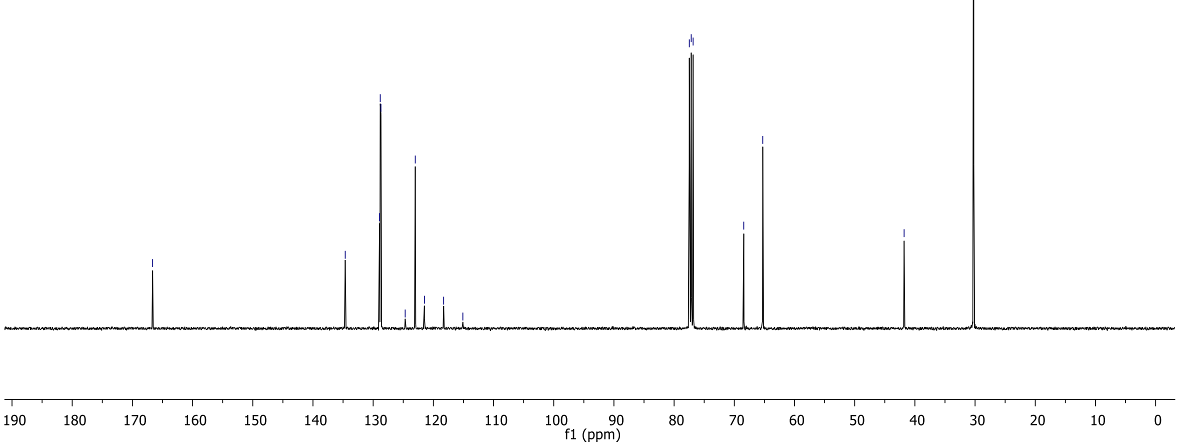

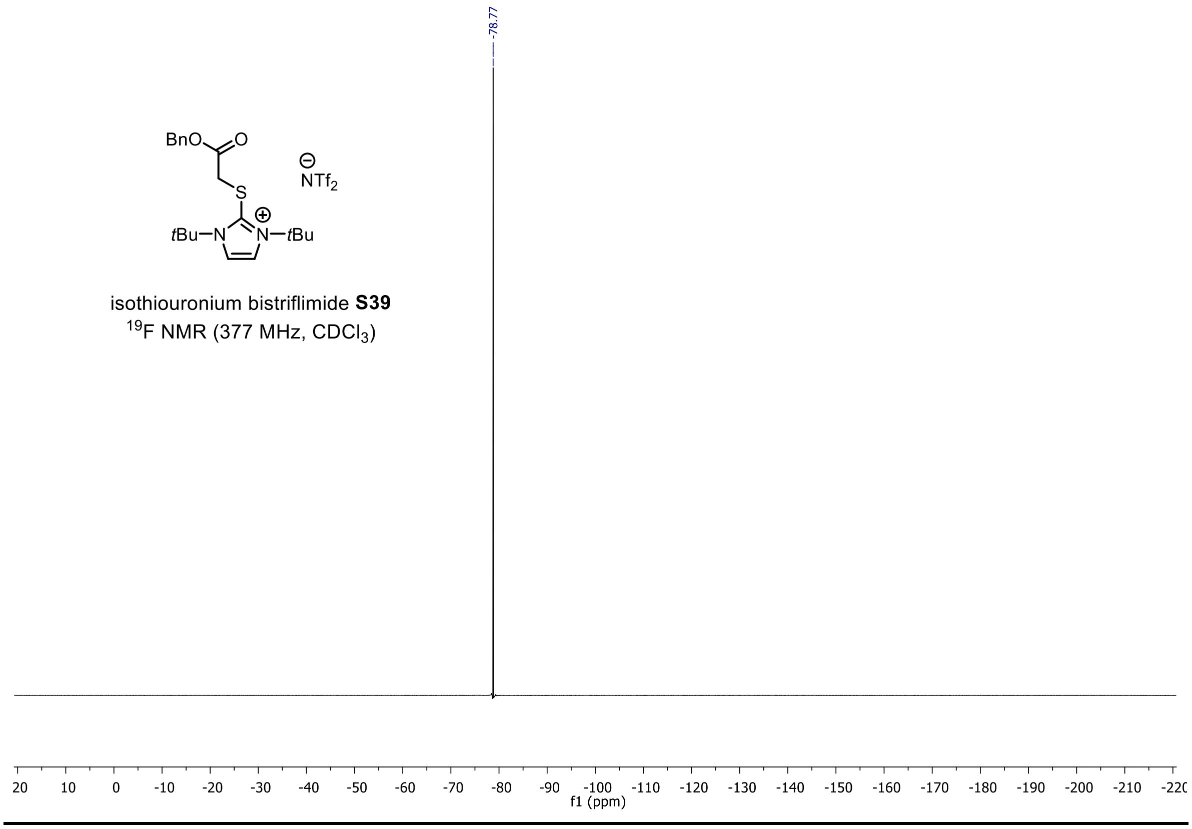

isothiouronium bistriflimide **S39**
^{19}F NMR (377 MHz, $CDCl_3$)

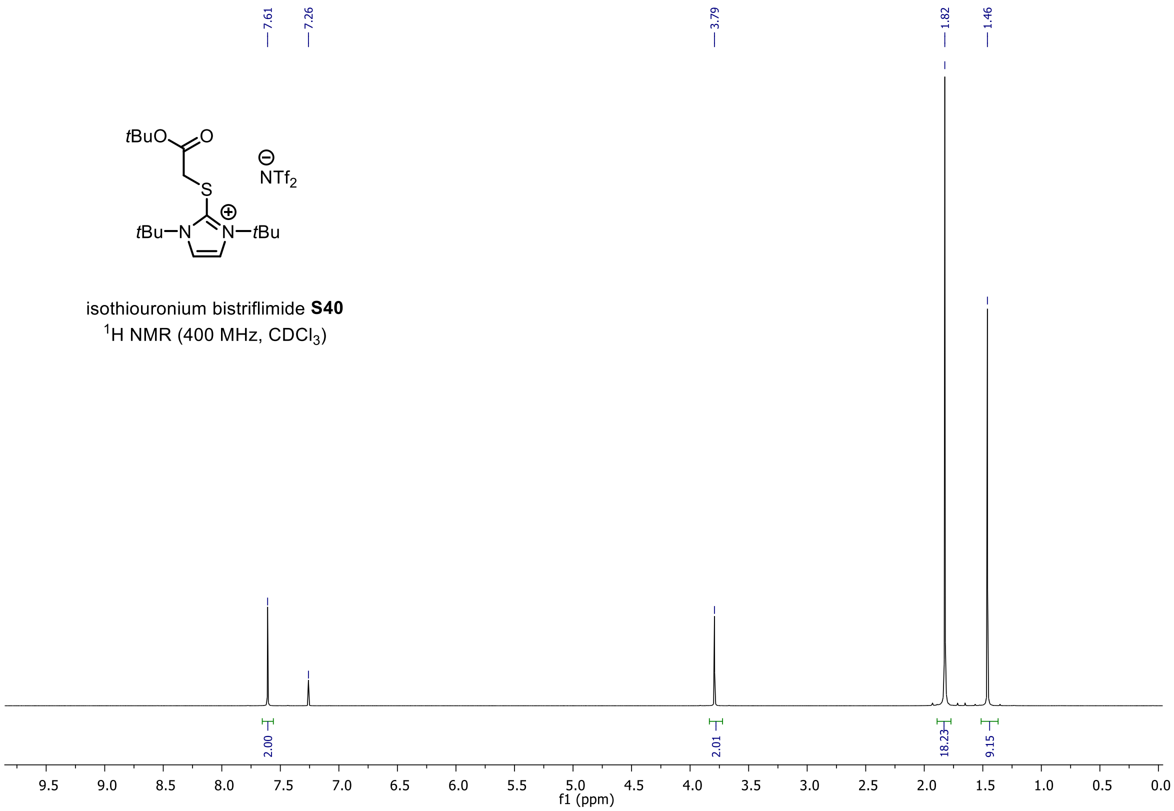

isothiouronium bistriflimide **S40**
^{1}H NMR (400 MHz, $CDCl_3$)

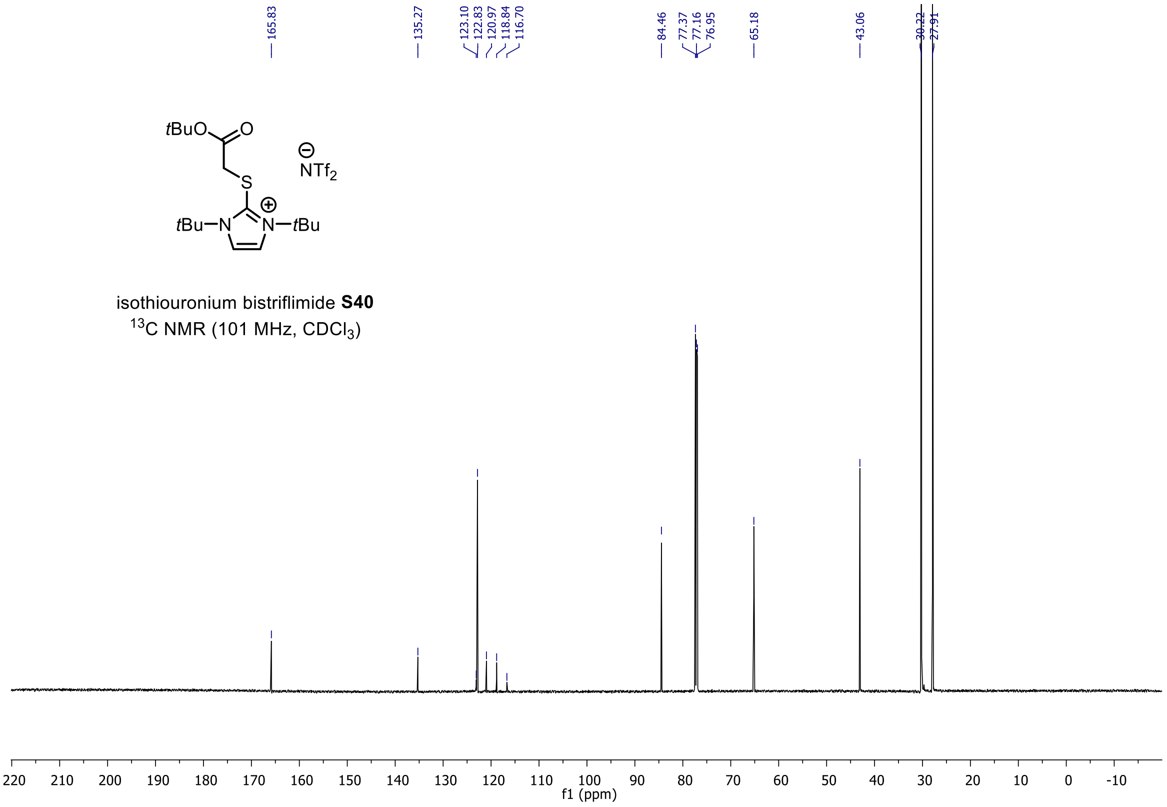

isothiouronium bistriflimide **S40**
^{13}C NMR (101 MHz, $CDCl_3$)

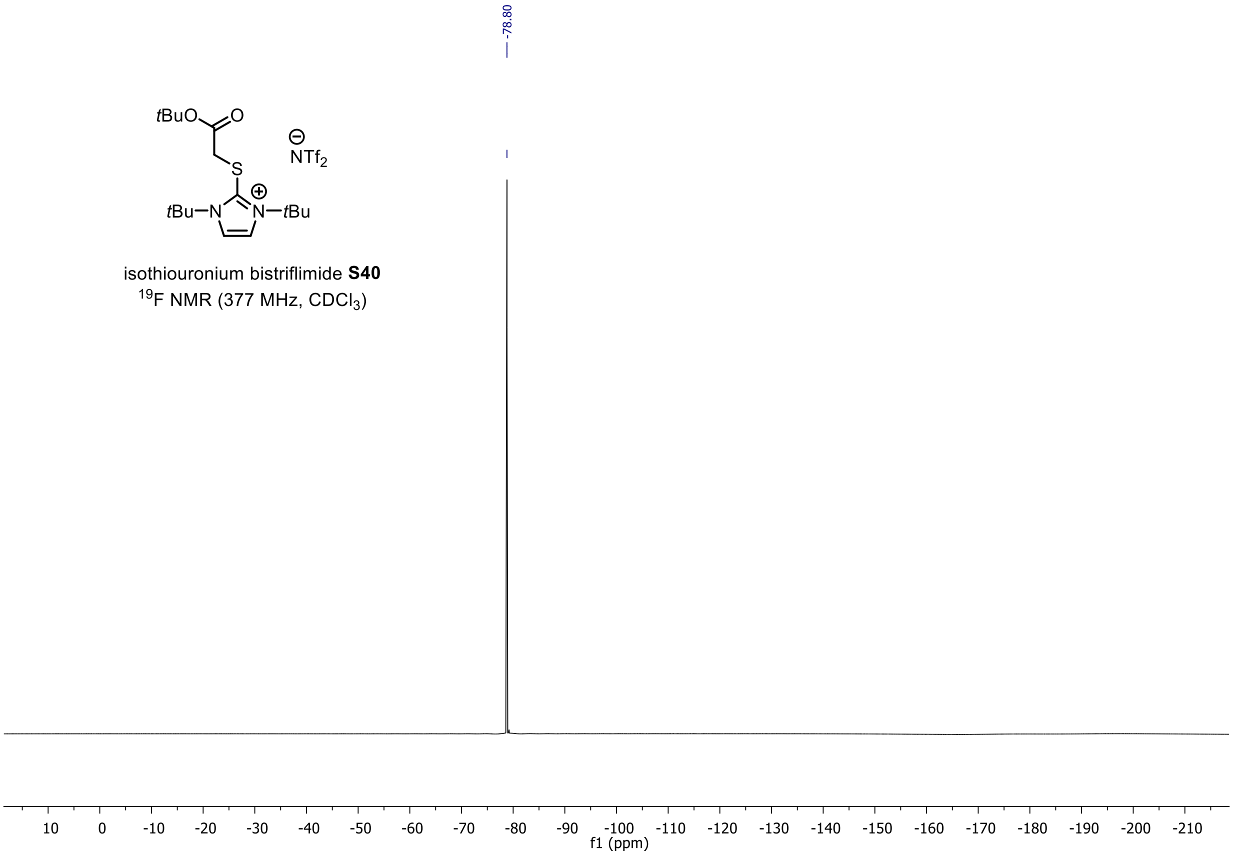

isothiouronium bistriflimide **S40**
^{19}F NMR (377 MHz, $CDCl_3$)

8.4 *Z*-olefins

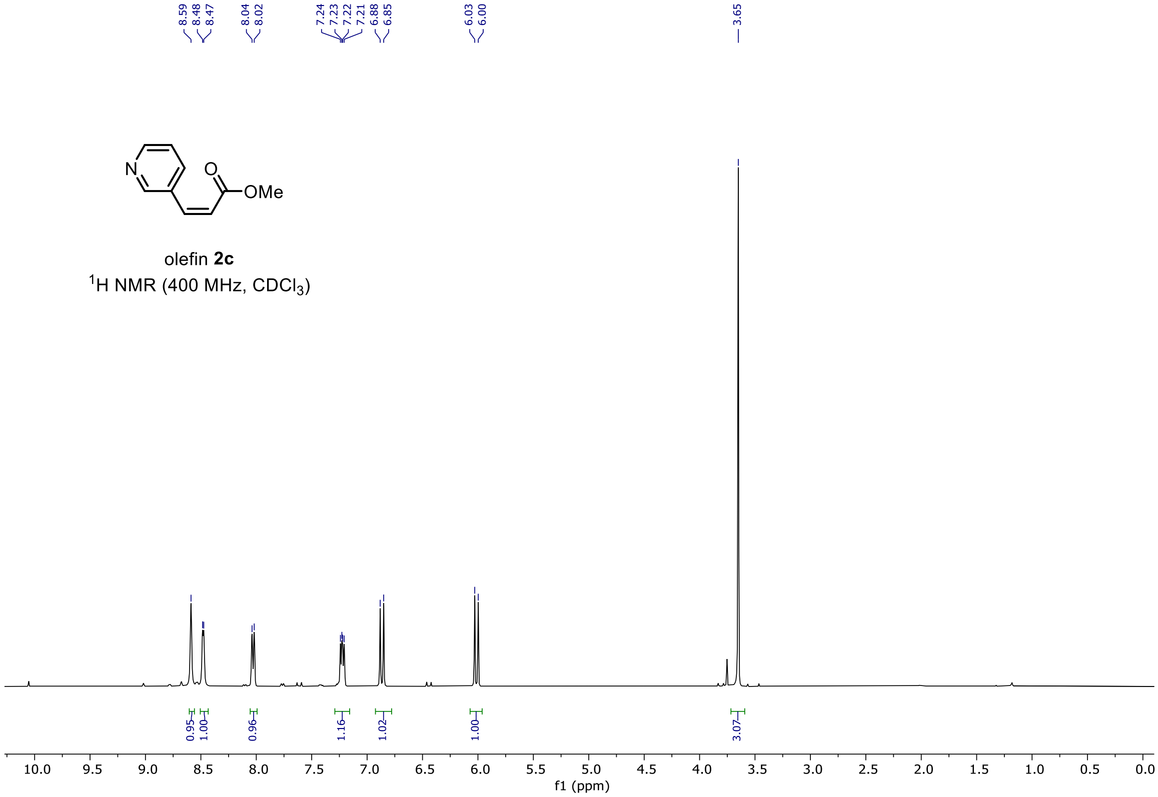

olefin **2c**
^{1}H NMR (400 MHz, $CDCl_3$)

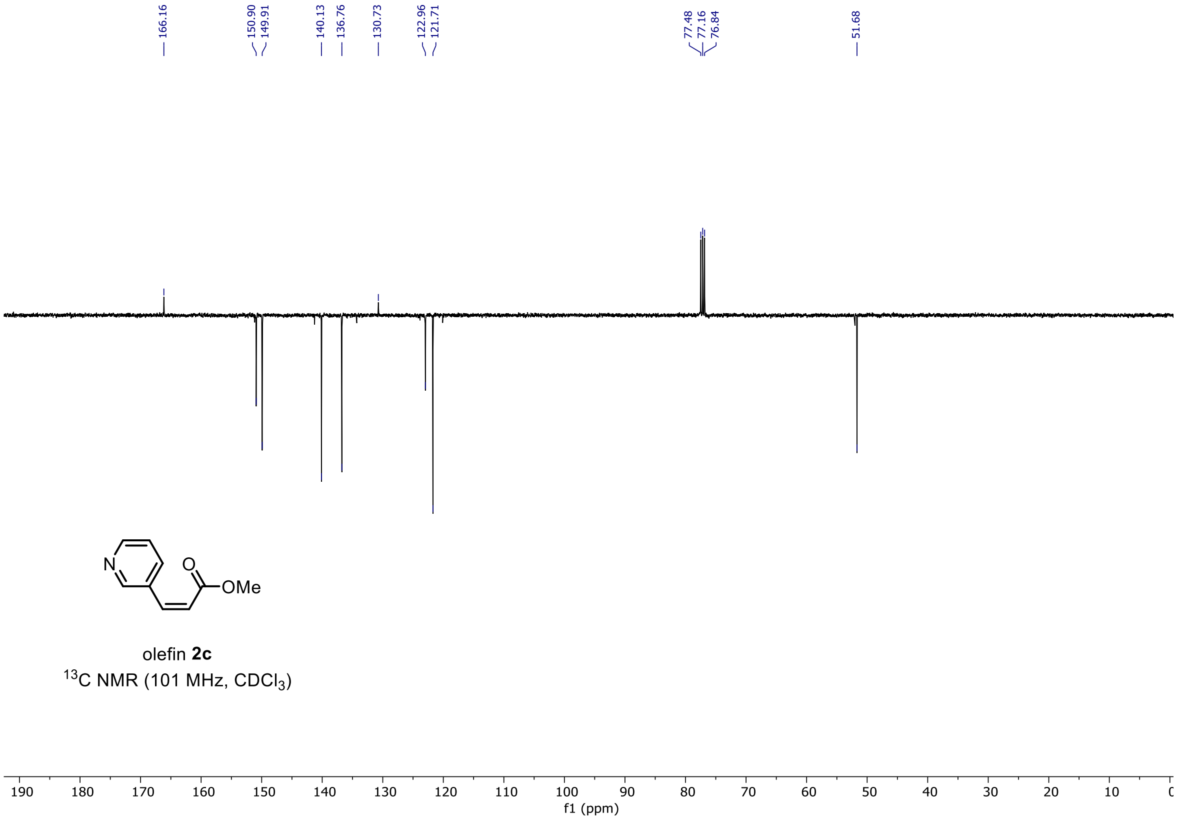

olefin **2c**
^{13}C NMR (101 MHz, $CDCl_3$)

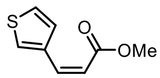

olefin **2d**

^{1}H NMR (400 MHz, $CDCl_3$)

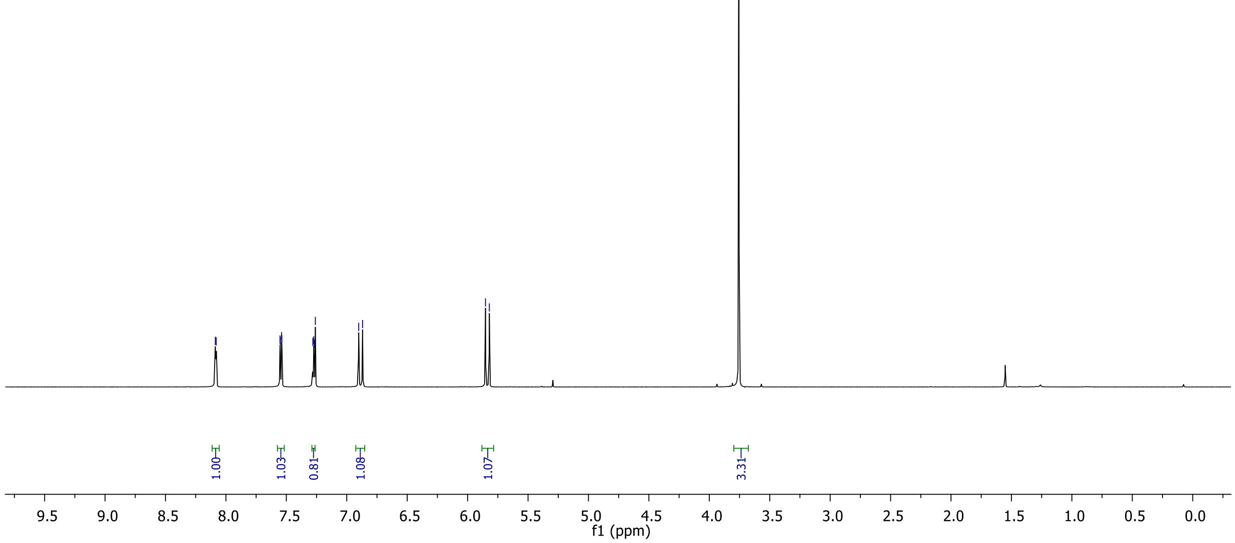

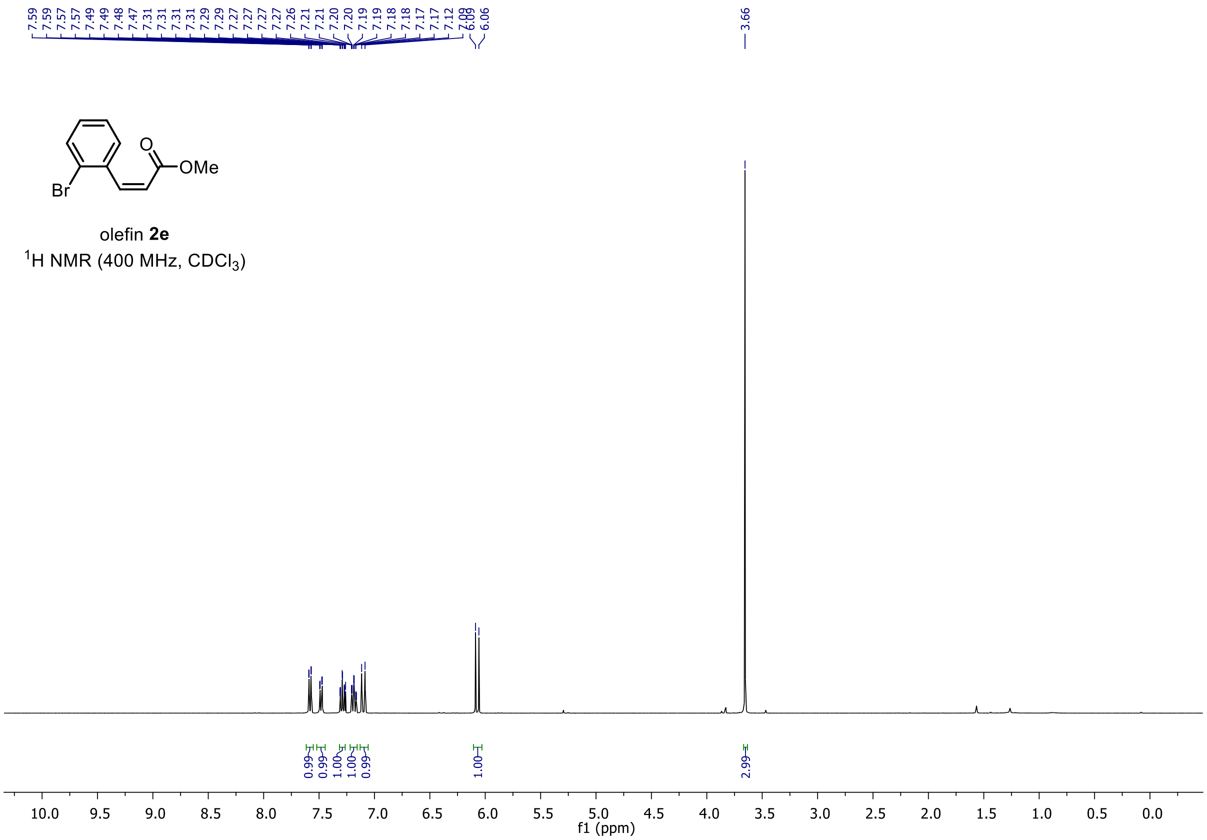
7.59
7.59
7.57
7.57
7.49
7.49
7.48
7.47
7.31
7.31
7.31
7.31
7.29
7.29
7.27
7.27
7.27
7.27
7.26
7.21
7.21
7.20
7.20
7.19
7.19
7.18
7.18
7.17
7.17
7.12
7.09
6.09
6.06
3.66
O
OMe
Br
olefin **2e**
^{1}H NMR (400 MHz, $CDCl_3$)
0.99
0.99
1.00
1.00
0.99
1.00
2.99
10.0 9.5 9.0 8.5 8.0 7.5 7.0 6.5 6.0 5.5 5.0 4.5 4.0 3.5 3.0 2.5 2.0 1.5 1.0 0.5 0.0
f1 (ppm)

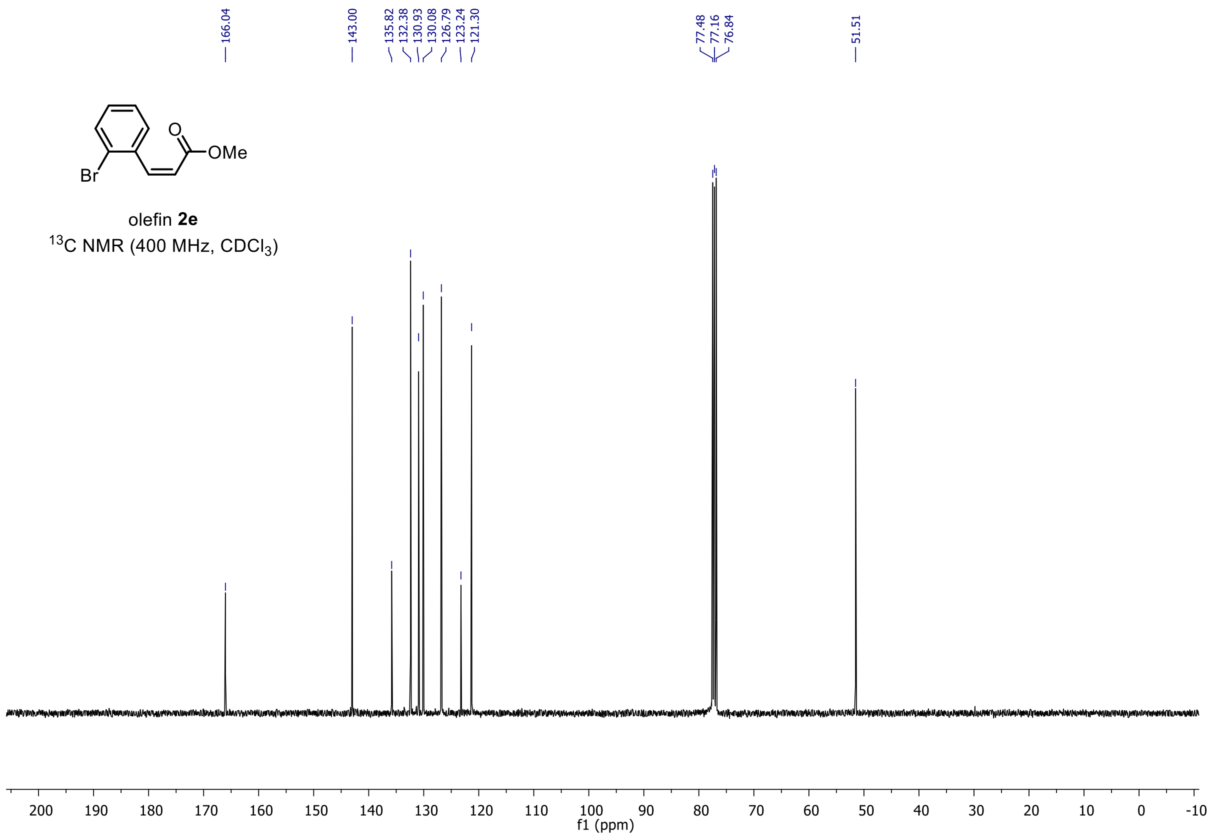
166.04
143.00
135.82
132.38
130.93
130.08
126.79
123.24
121.30
77.48
77.16
76.84
51.51
O
OMe
Br
olefin **2e**
^{13}C NMR (400 MHz, $CDCl_3$)
200 190 180 170 160 150 140 130 120 110 100 90 80 70 60 50 40 30 20 10 0 -10
f1 (ppm)

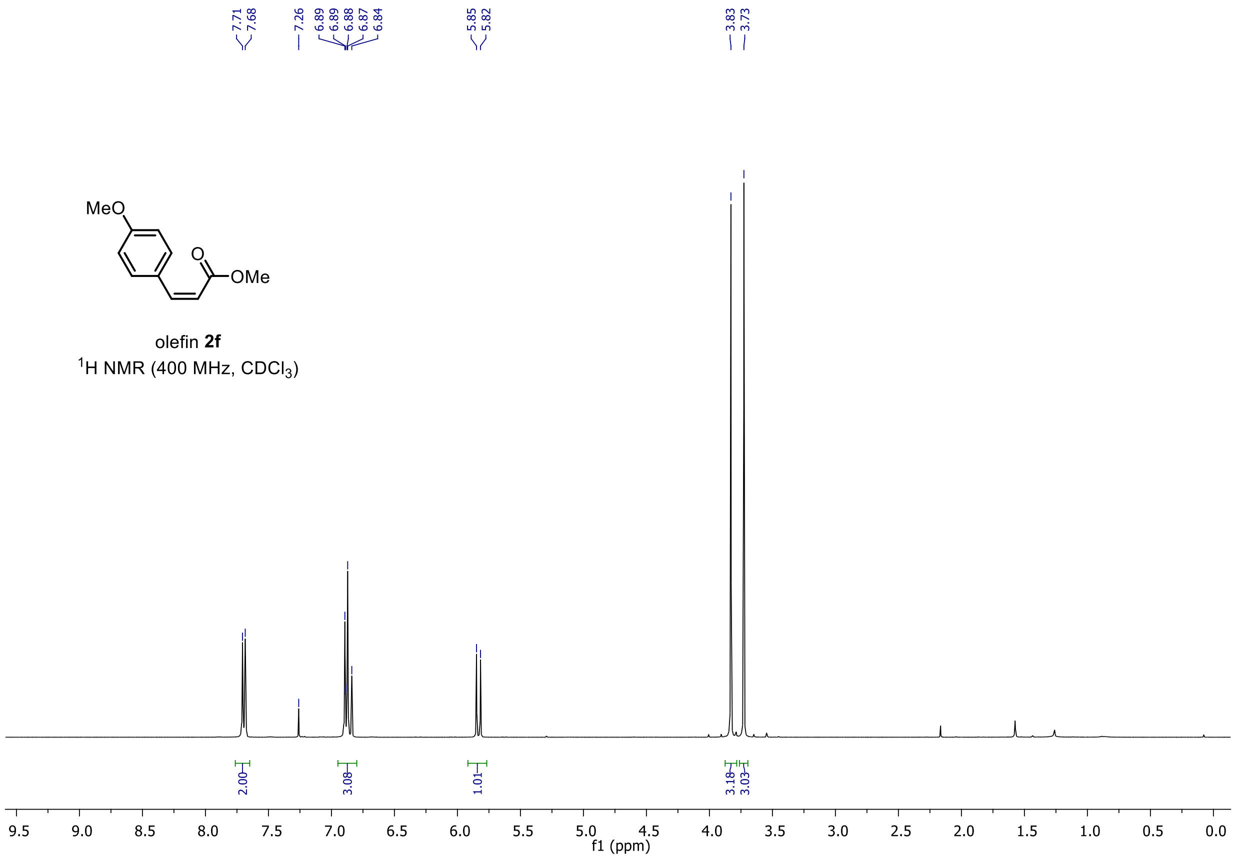

olefin **2f**

^{1}H NMR (400 MHz, $CDCl_3$)

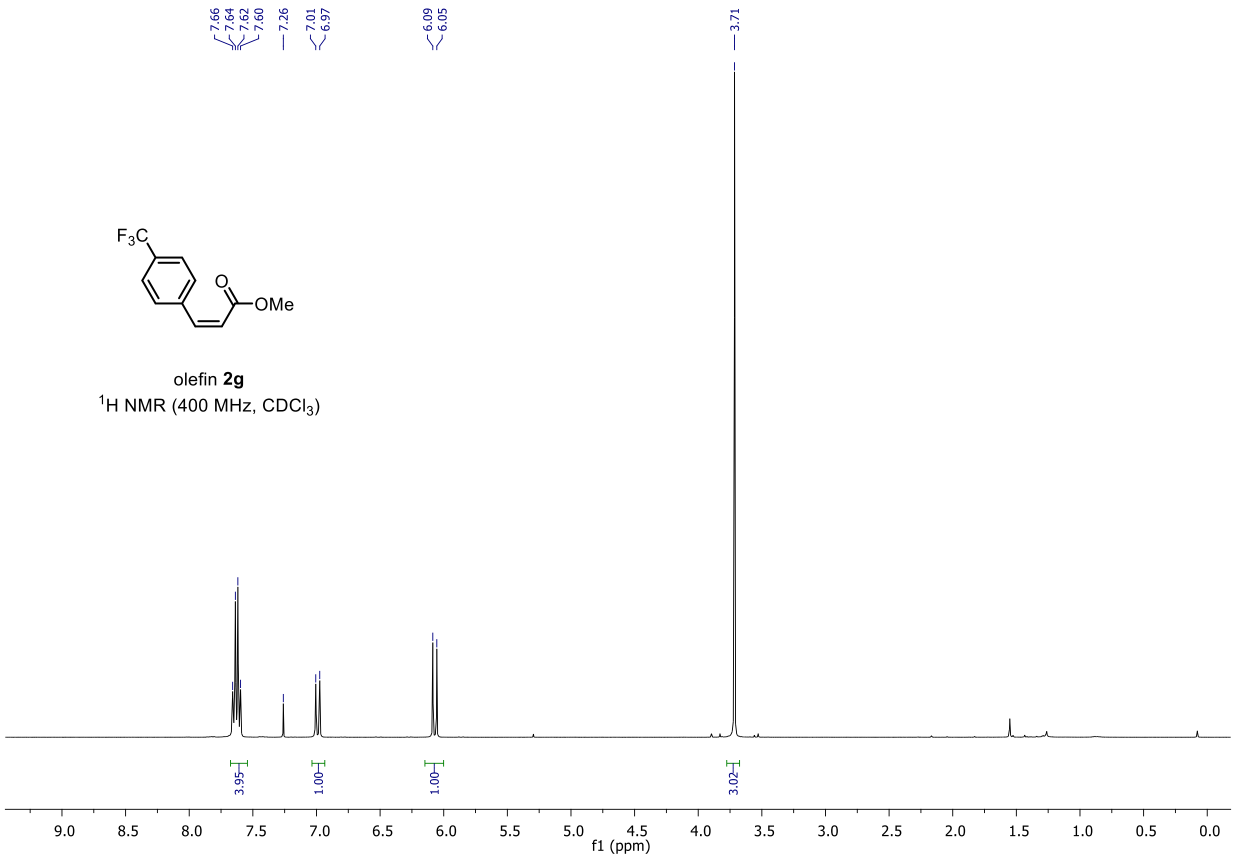

olefin **2g**

^{1}H NMR (400 MHz, $CDCl_3$)

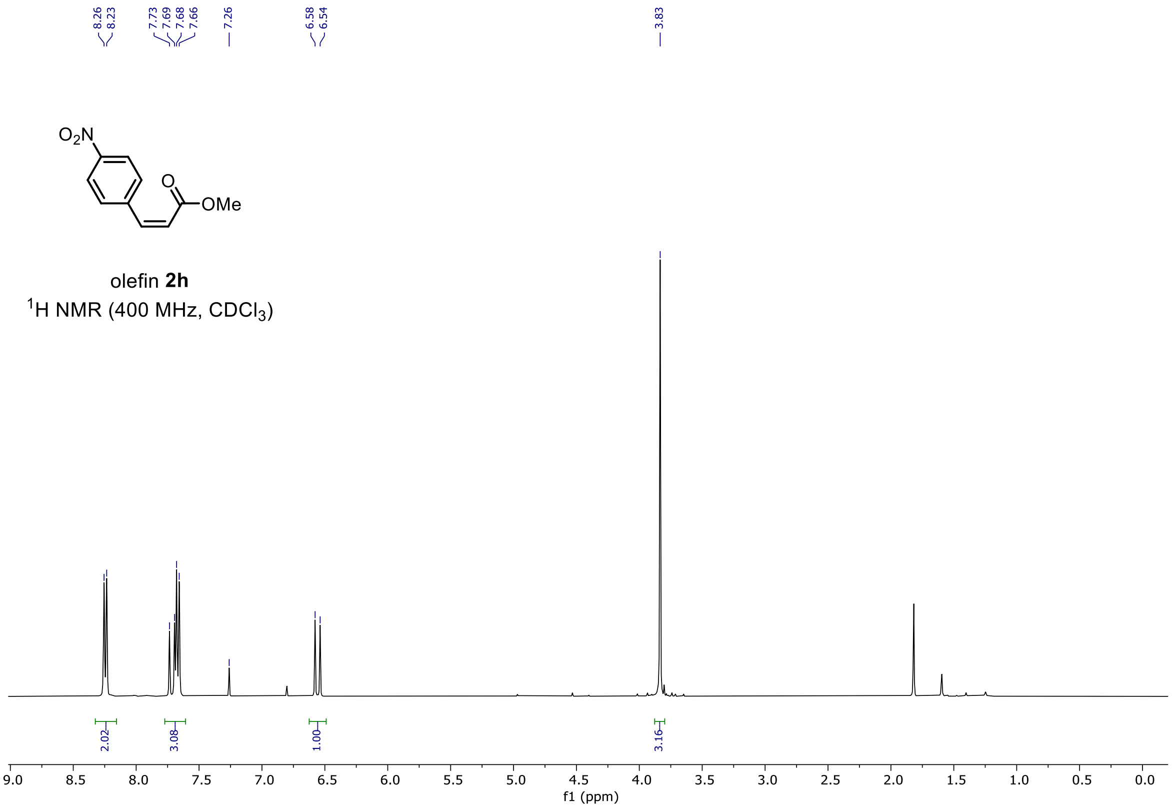

8.26
8.23
7.73
7.69
7.68
7.66
7.26
6.58
6.54
3.83
O2N
O
OMe
olefin 2h
1H NMR (400 MHz, CDCl3)
2.02
3.08
1.00
3.16
9.0 8.5 8.0 7.5 7.0 6.5 6.0 5.5 5.0 4.5 4.0 3.5 3.0 2.5 2.0 1.5 1.0 0.5 0.0
f1 (ppm)

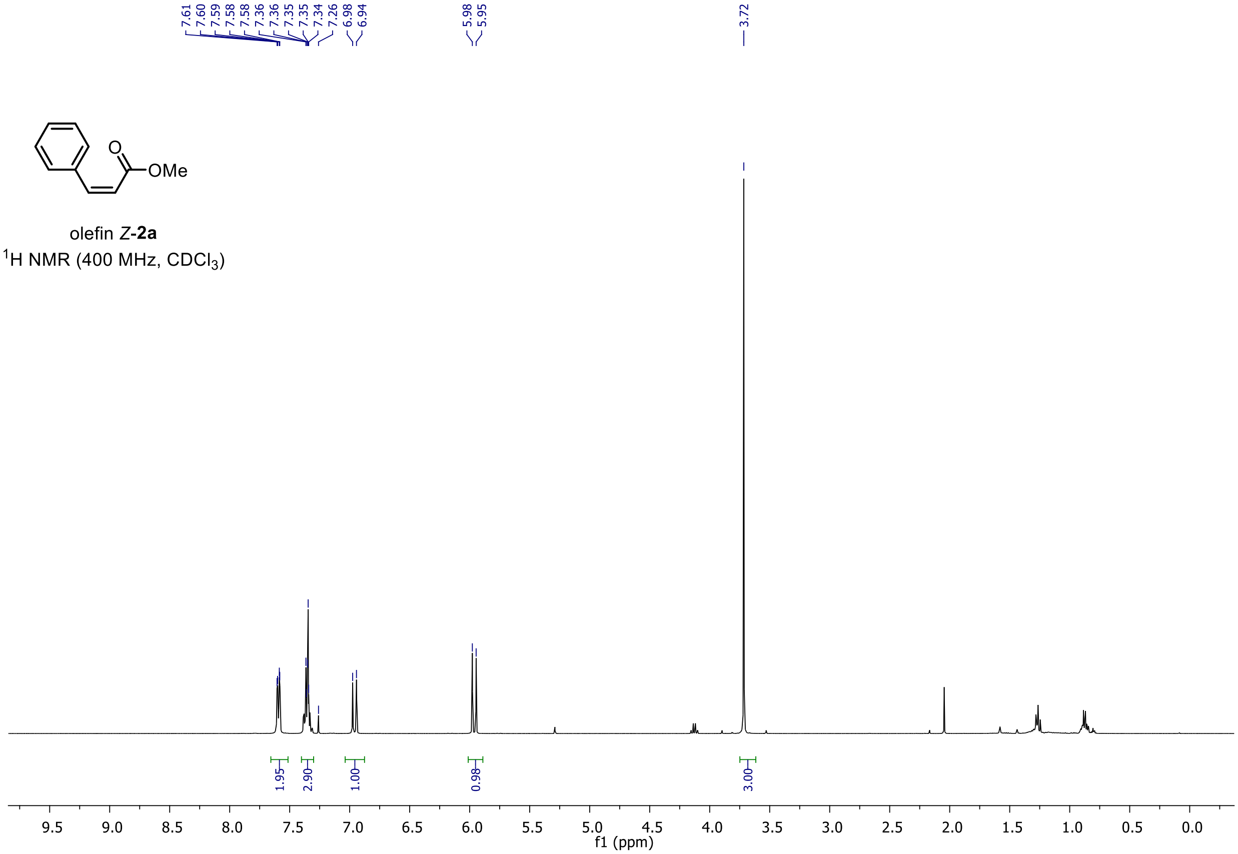

7.61
7.60
7.59
7.58
7.58
7.36
7.36
7.35
7.35
7.34
7.26
6.98
6.94
5.98
5.95
3.72
O
OMe
olefin Z-2a
1H NMR (400 MHz, CDCl3)
1.95
2.90
1.00
0.98
3.00
9.5 9.0 8.5 8.0 7.5 7.0 6.5 6.0 5.5 5.0 4.5 4.0 3.5 3.0 2.5 2.0 1.5 1.0 0.5 0.0
f1 (ppm)

olefin **2i**
^{1}H NMR (600 MHz, $CDCl_3$)

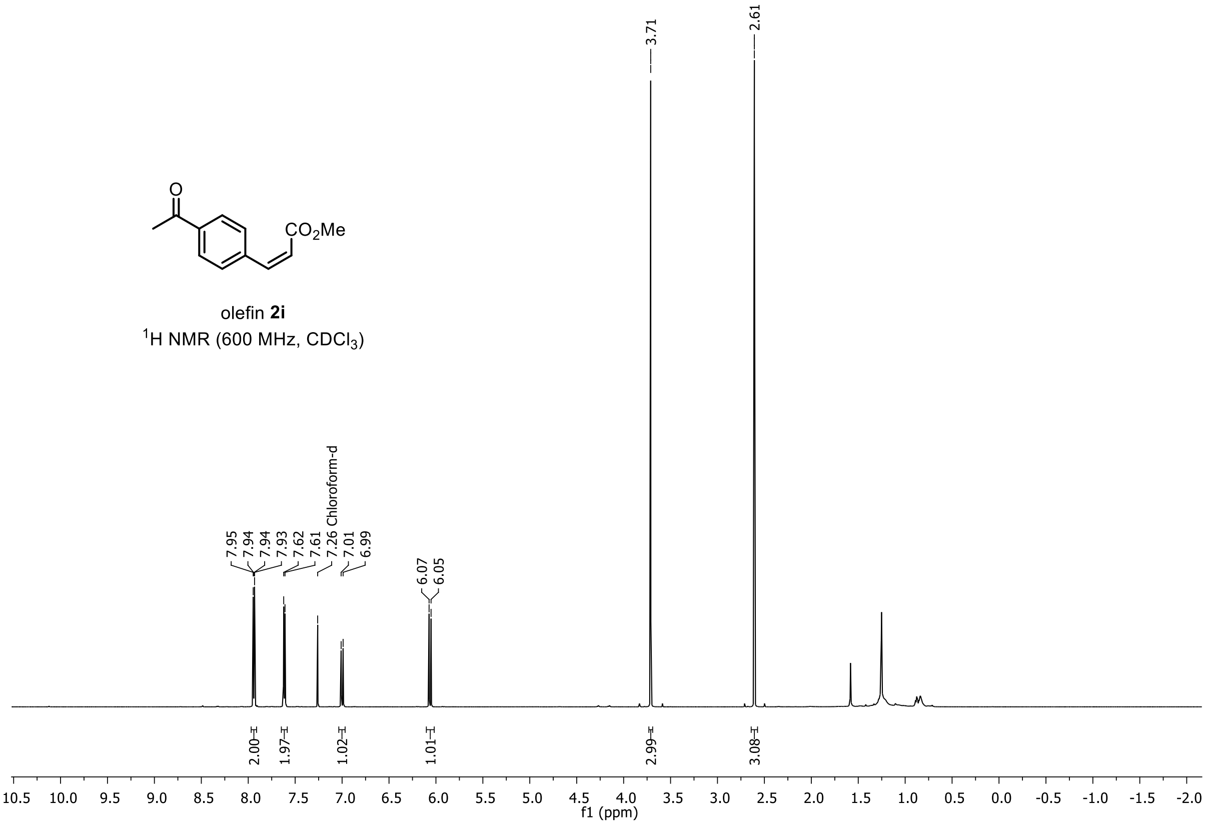

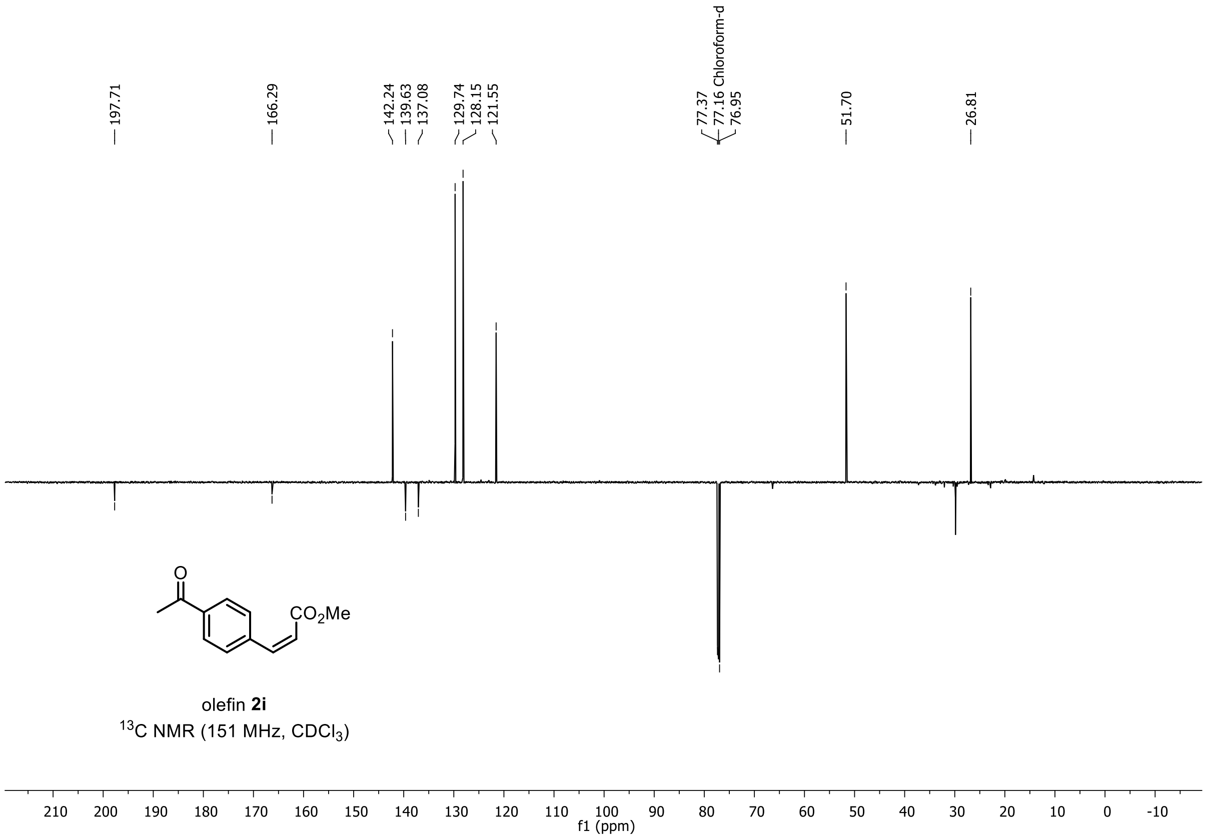

olefin **2i**
^{13}C NMR (151 MHz, $CDCl_3$)

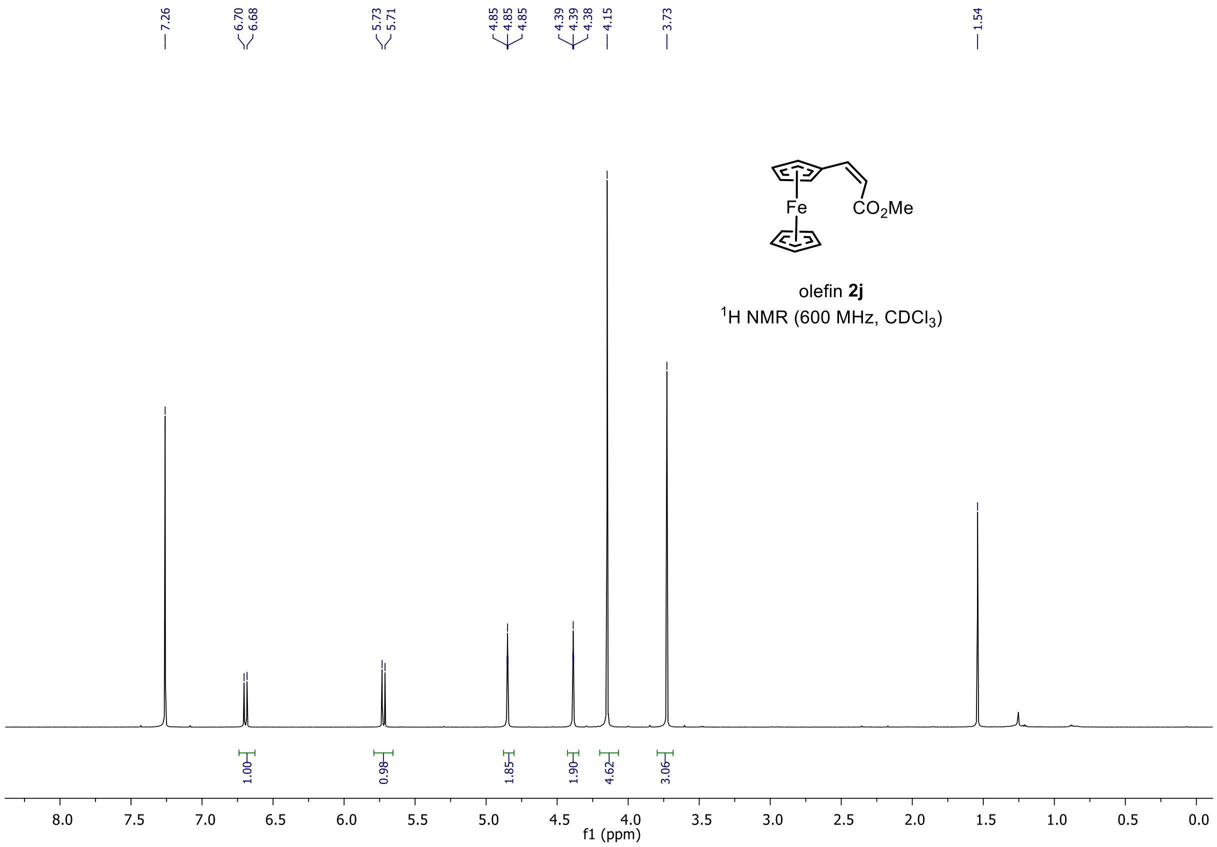
7.26
6.70
6.68
5.73
5.71
4.85
4.85
4.85
4.39
4.39
4.38
4.15
3.73
1.54
Fe
CO2Me
olefin 2j
1H NMR (600 MHz, CDCl3)
1.00
0.98
1.85
1.90
4.62
3.06
8.0
7.5
7.0
6.5
6.0
5.5
5.0
4.5
4.0
3.5
3.0
2.5
2.0
1.5
1.0
0.5
0.0
f1 (ppm)

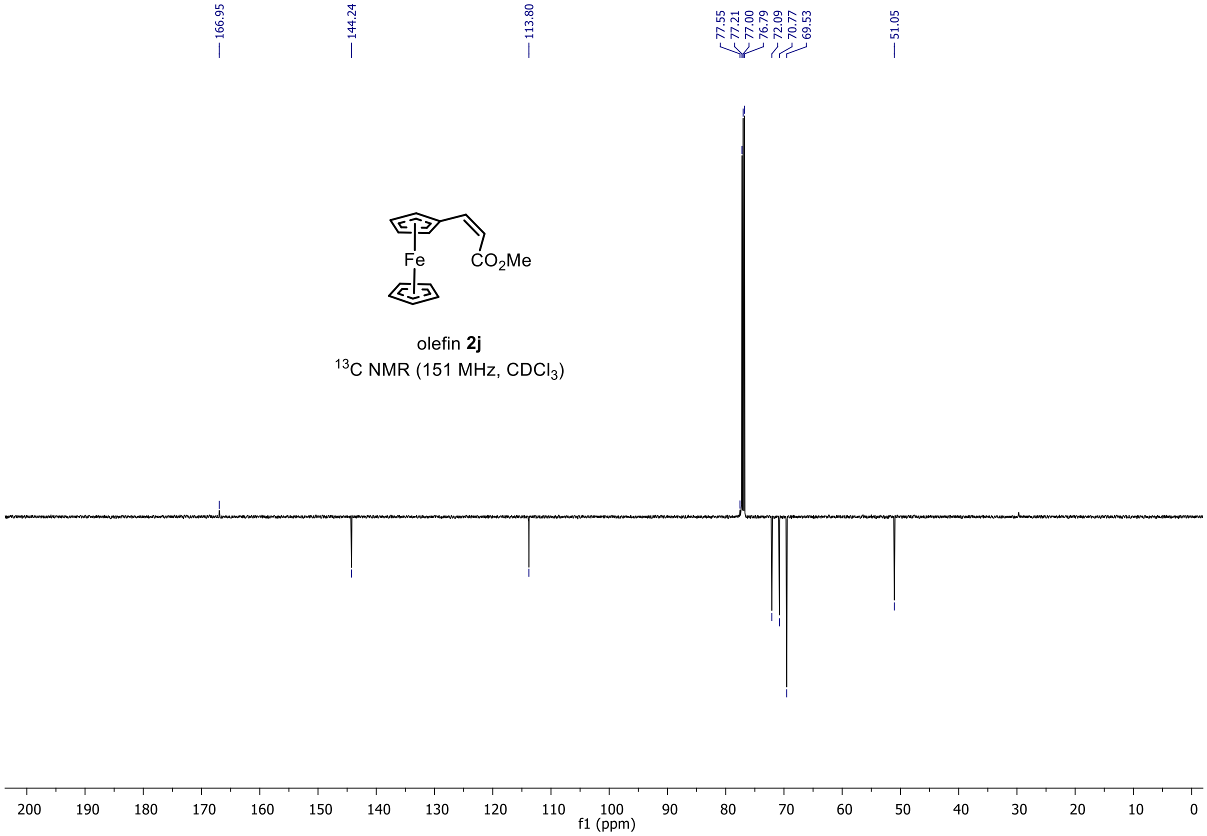
166.95
144.24
113.80
77.55
77.21
77.00
76.79
72.09
70.77
69.53
51.05
Fe
CO2Me
olefin 2j
13C NMR (151 MHz, CDCl3)
200
190
180
170
160
150
140
130
120
110
100
90
80
70
60
50
40
30
20
10
0
f1 (ppm)

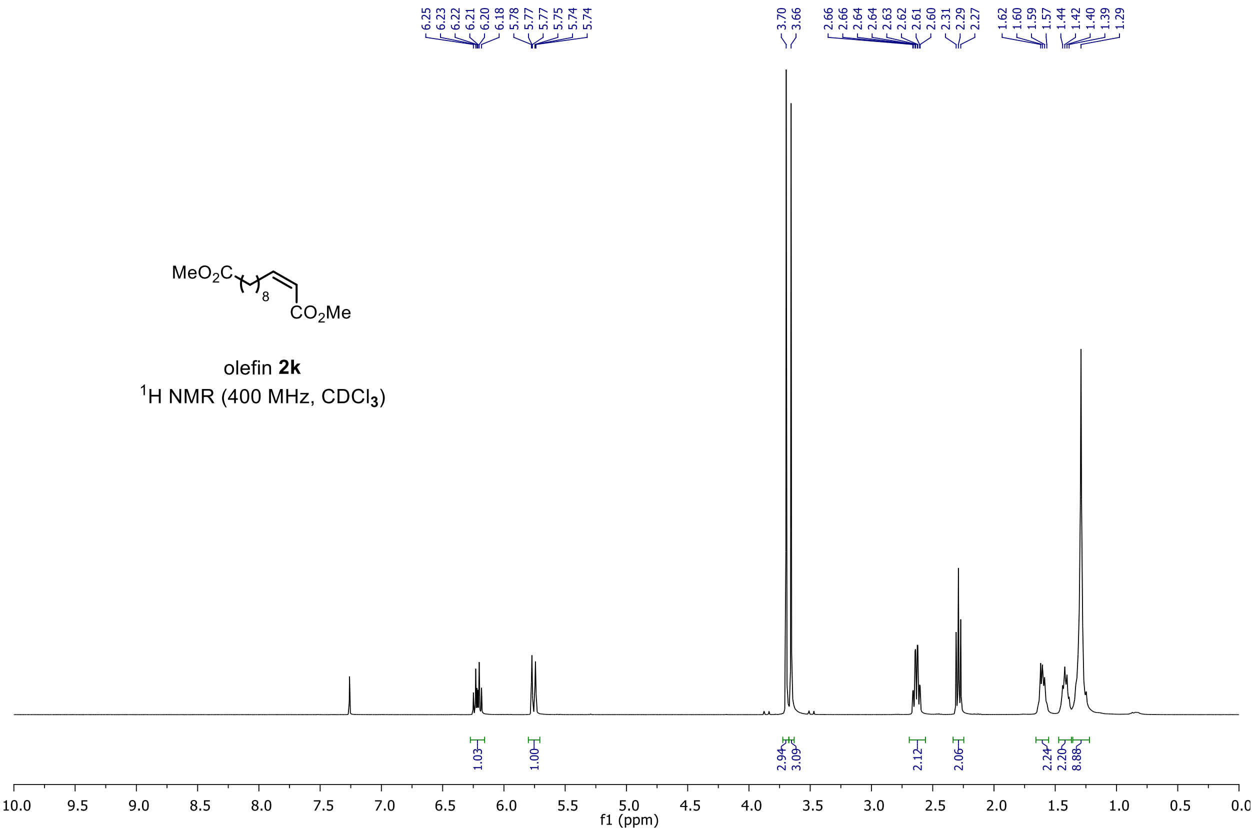

olefin **2k**

^{1}H NMR (400 MHz, $CDCl_3$)

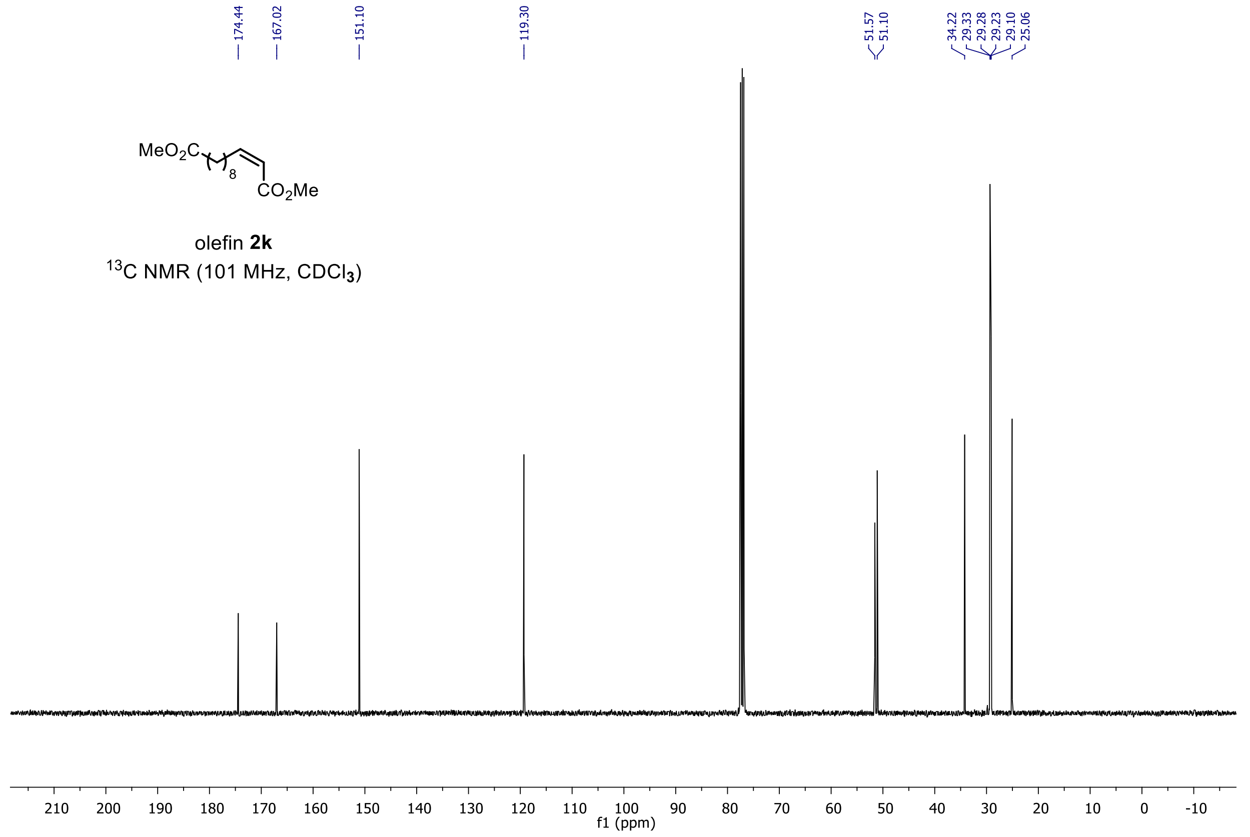

olefin **2k**

^{13}C NMR (101 MHz, $CDCl_3$)

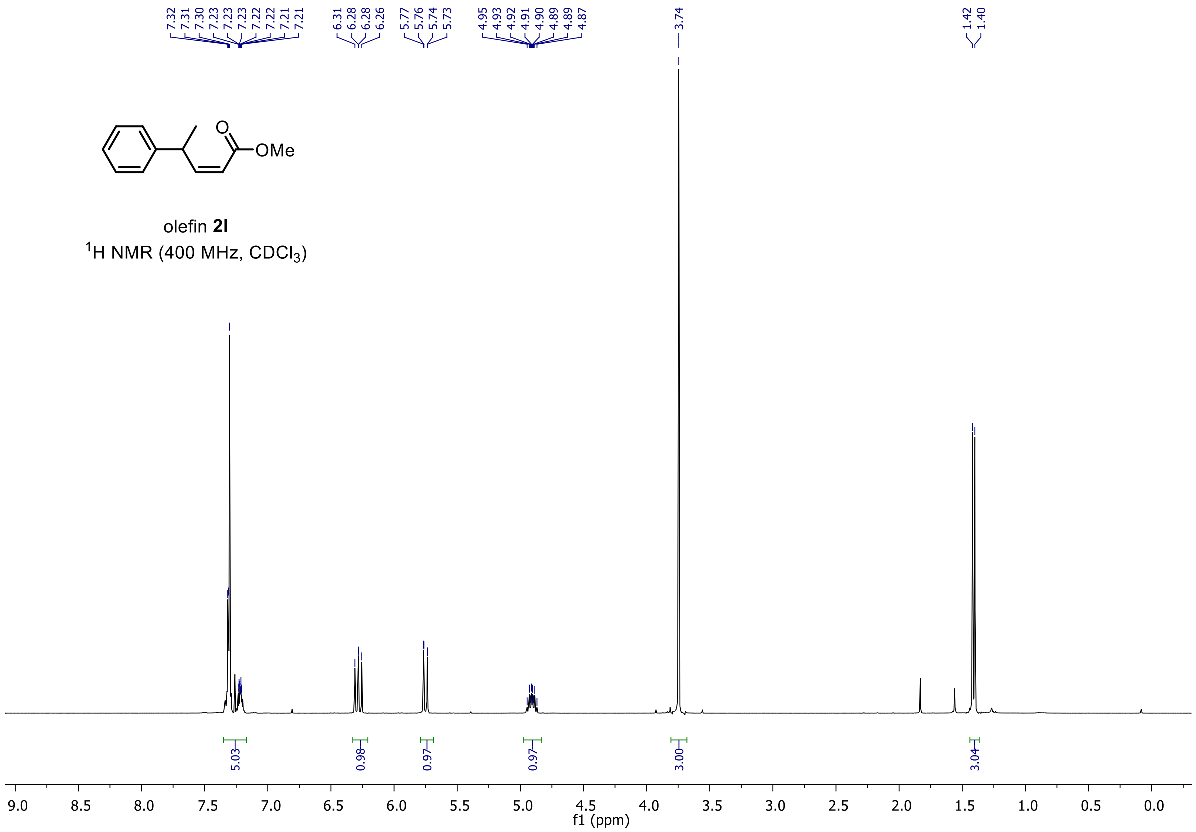
OMe
olefin 2l
^{1}H NMR (400 MHz, CDCl$_3$)
7.32 7.31 7.30 7.23 7.23 7.23 7.22 7.22 7.21 7.21
6.31 6.28 6.28 6.26
5.77 5.76 5.74 5.73
4.95 4.93 4.92 4.91 4.90 4.89 4.89 4.87
3.74
1.42 1.40
5.03 0.98 0.97 0.97 3.00 3.04
f1 (ppm)

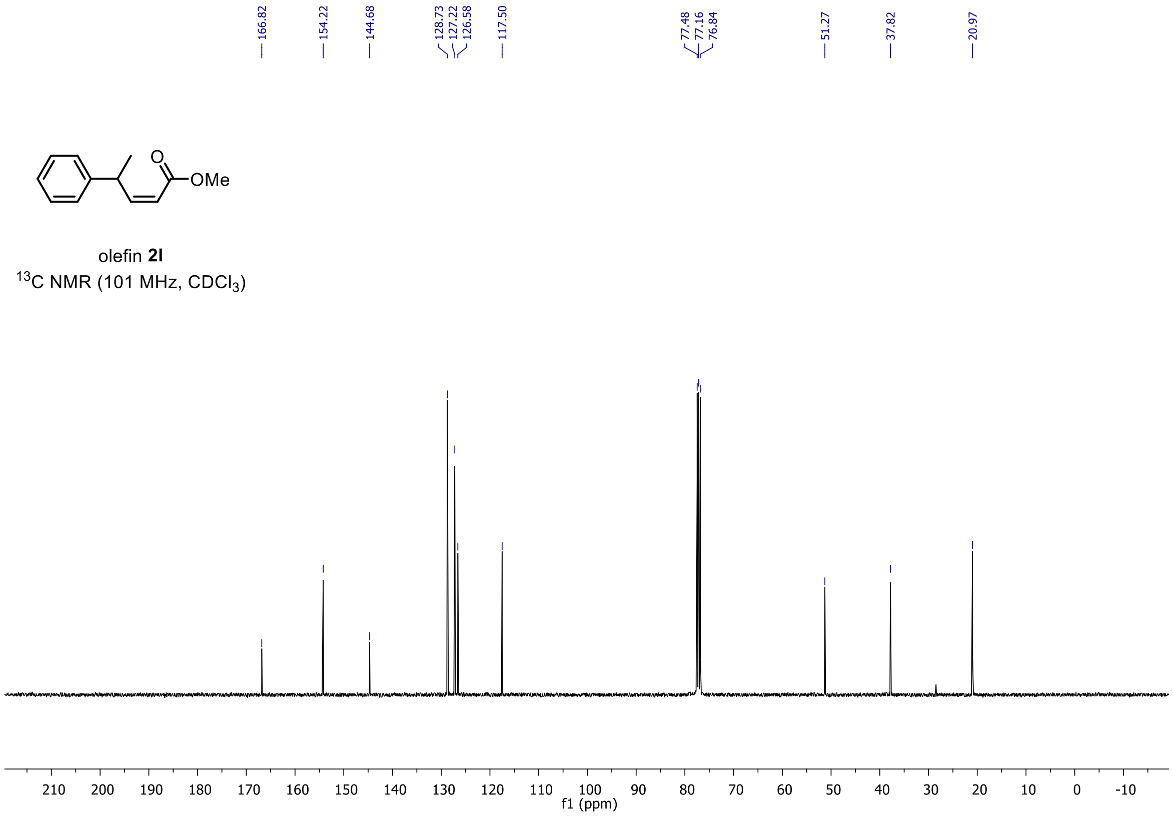
OMe
olefin 2l
^{13}C NMR (101 MHz, CDCl$_3$)
166.82 154.22 144.68 128.73 127.22 126.58 117.50 77.48 77.16 76.84 51.27 37.82 20.97
f1 (ppm)

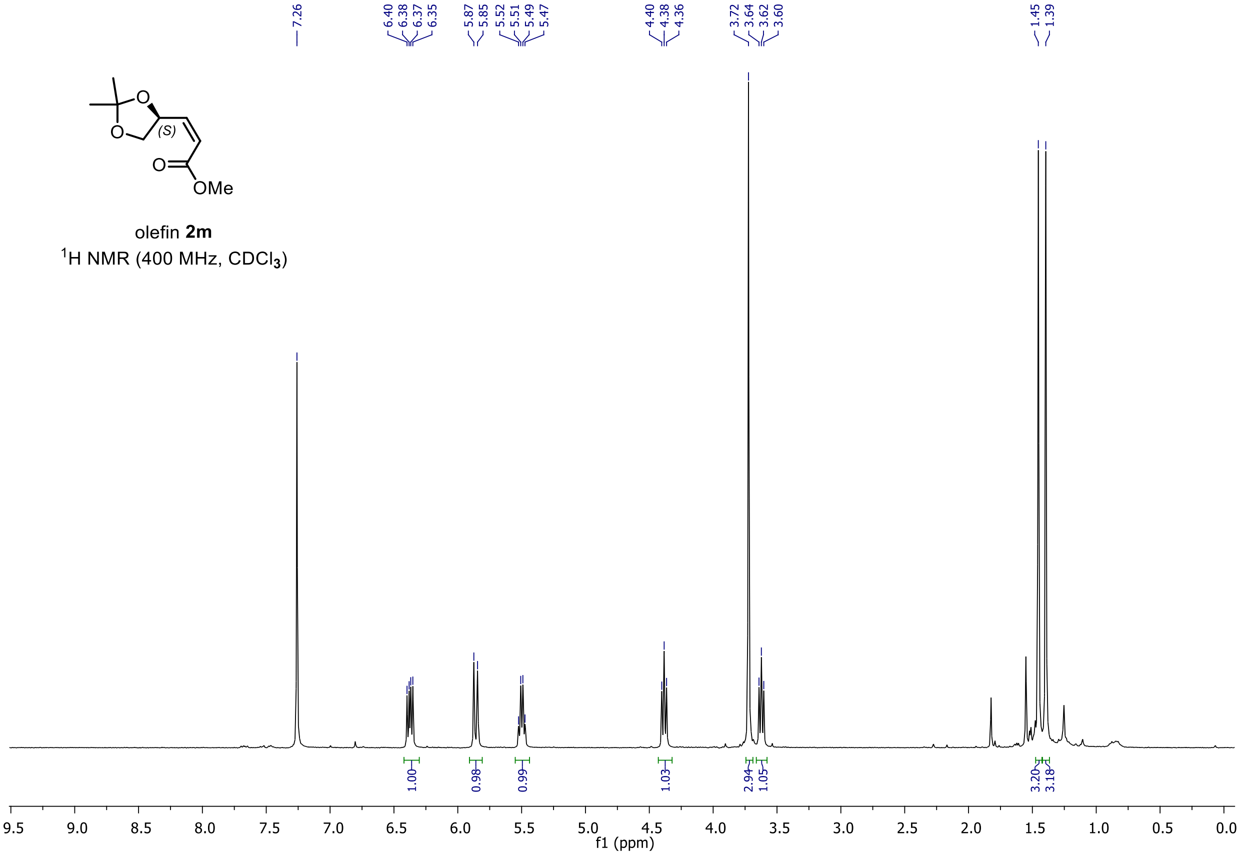

olefin **2m**

^{1}H NMR (400 MHz, $CDCl_3$)

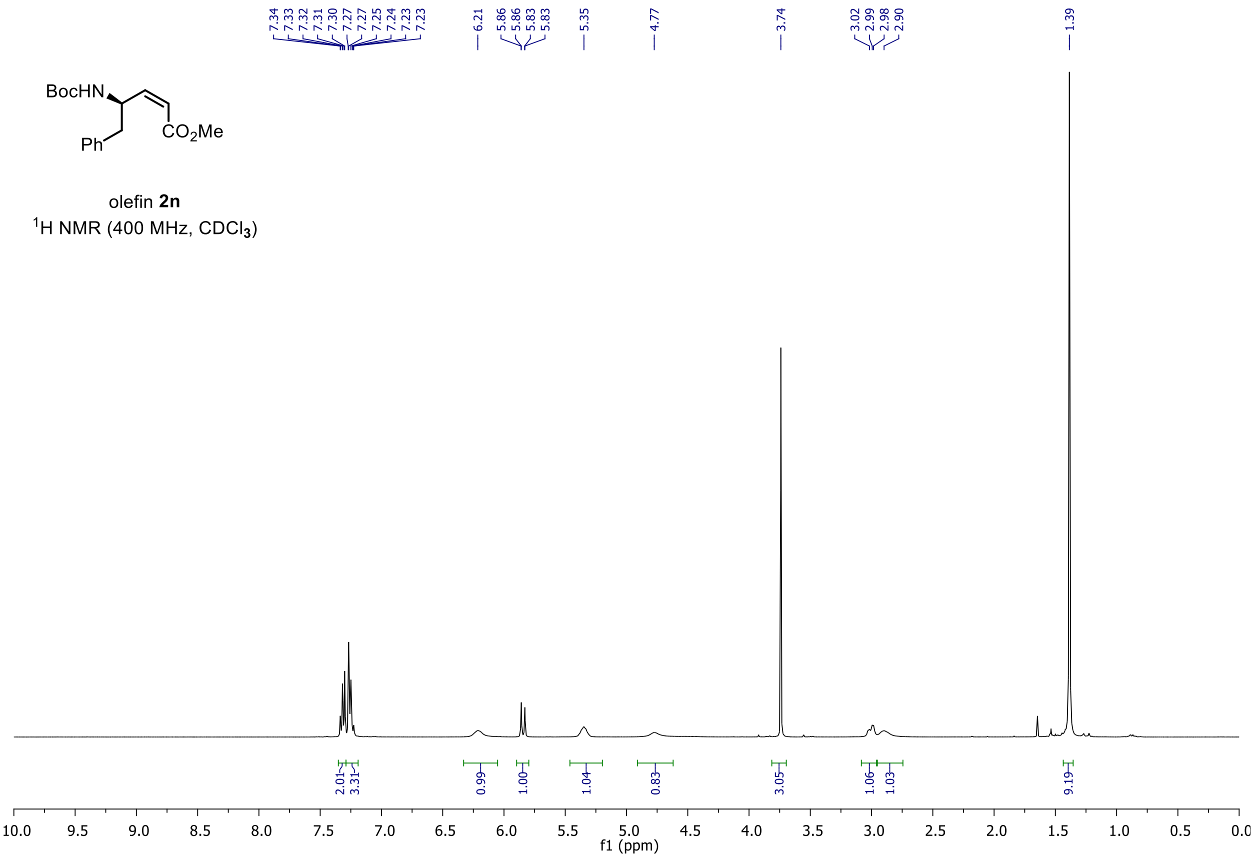

olefin **2n**
^{1}H NMR (400 MHz, $CDCl_3$)

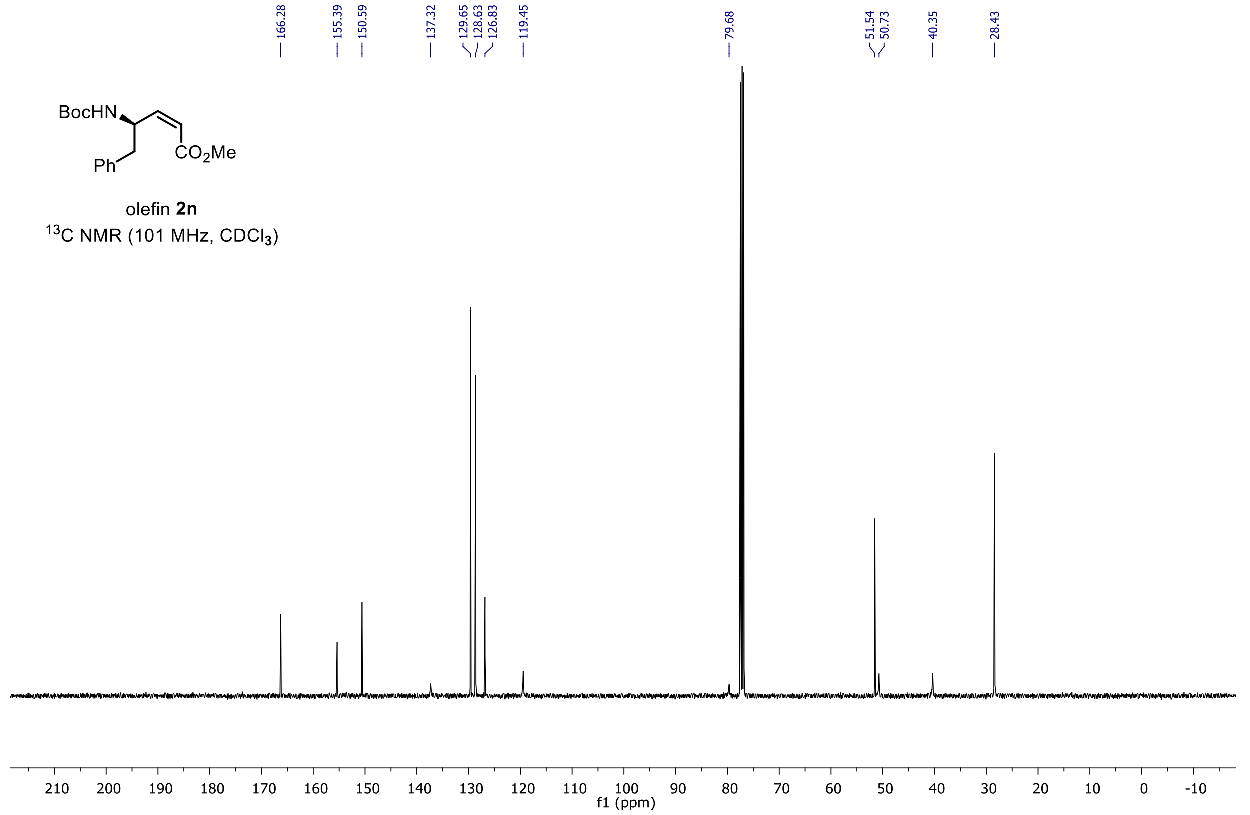

olefin **2n**
^{13}C NMR (101 MHz, $CDCl_3$)

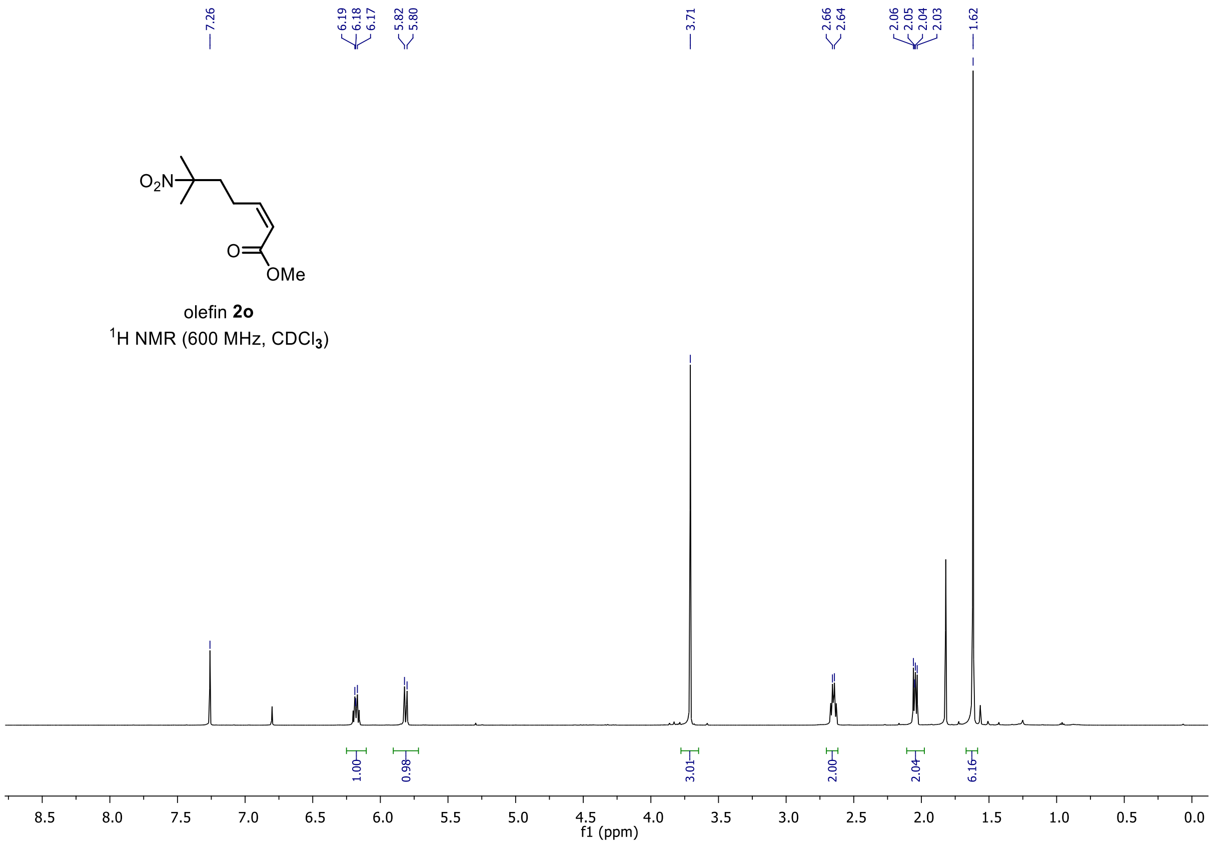

7.26
6.19
6.18
6.17
5.82
5.80
3.71
2.66
2.64
2.06
2.05
2.04
2.03
1.62
O_2N
O
OMe
olefin **2o**
^{1}H NMR (600 MHz, $CDCl_3$)
1.00
0.98
3.01
2.00
2.04
6.16
f1 (ppm)

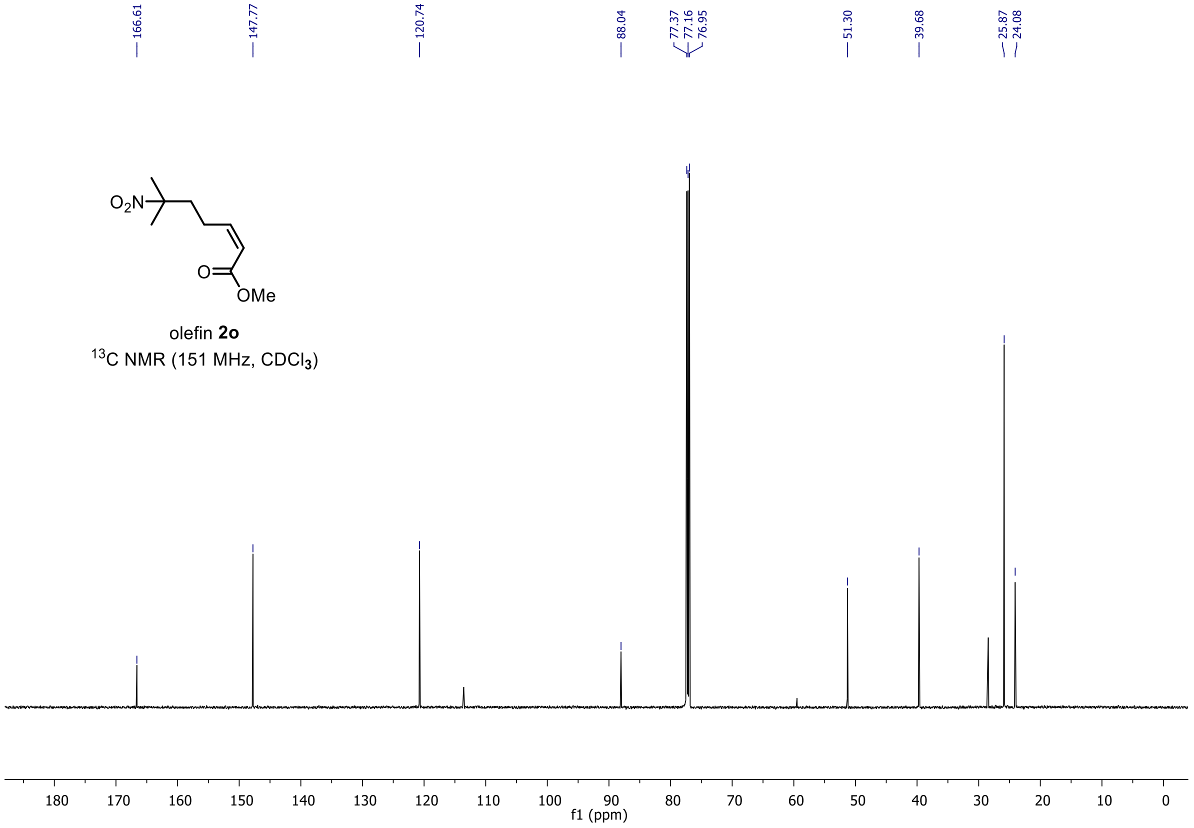

166.61
147.77
120.74
88.04
77.37
77.16
76.95
51.30
39.68
25.87
24.08
O_2N
O
OMe
olefin **2o**
^{13}C NMR (151 MHz, $CDCl_3$)
f1 (ppm)

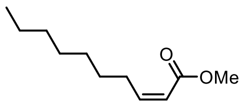

olefin **2p**

^{1}H NMR (400 MHz, $CDCl_3$)

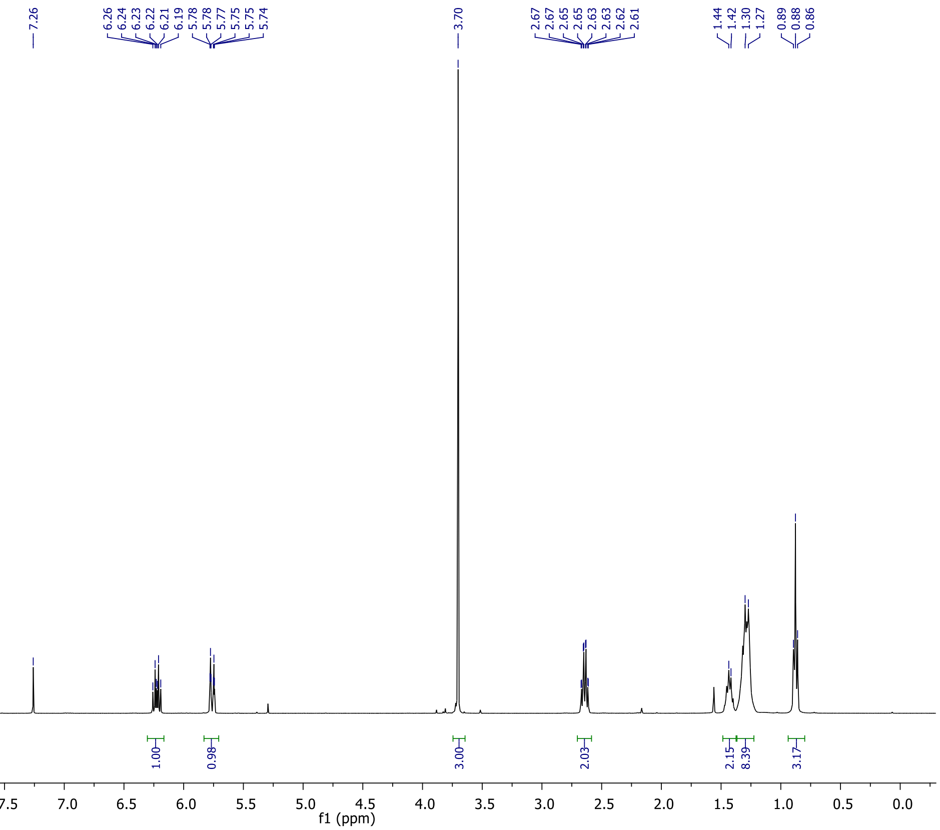

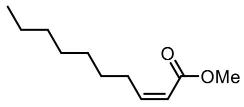

olefin **2p**

^{13}C NMR (101 MHz, $CDCl_3$)

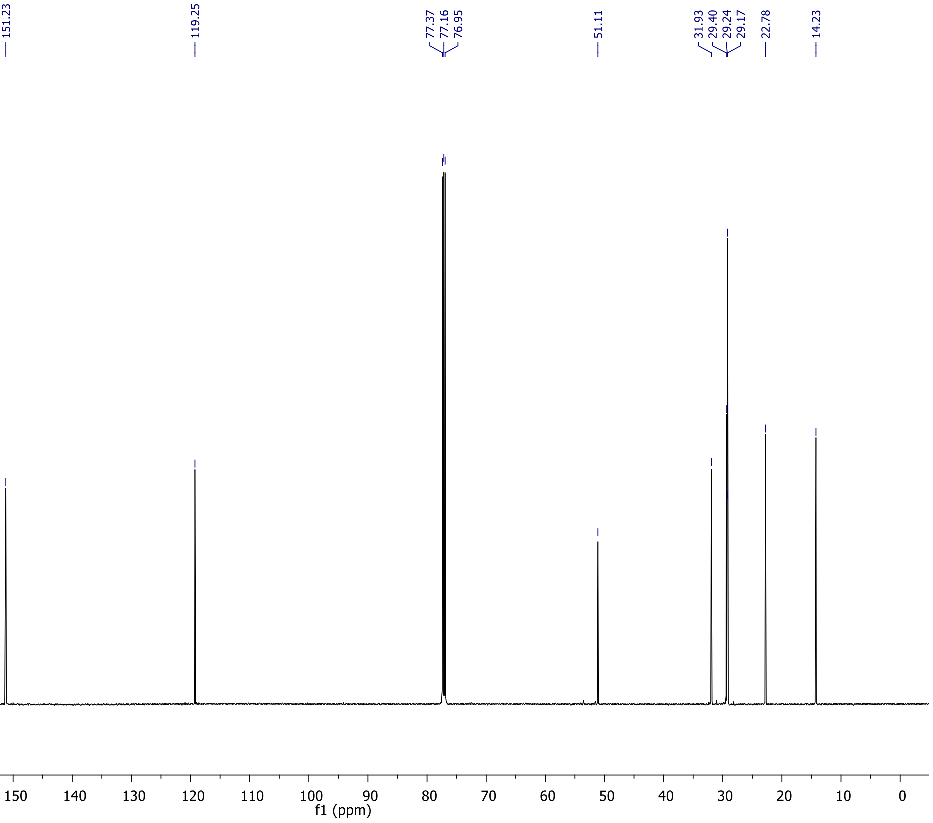

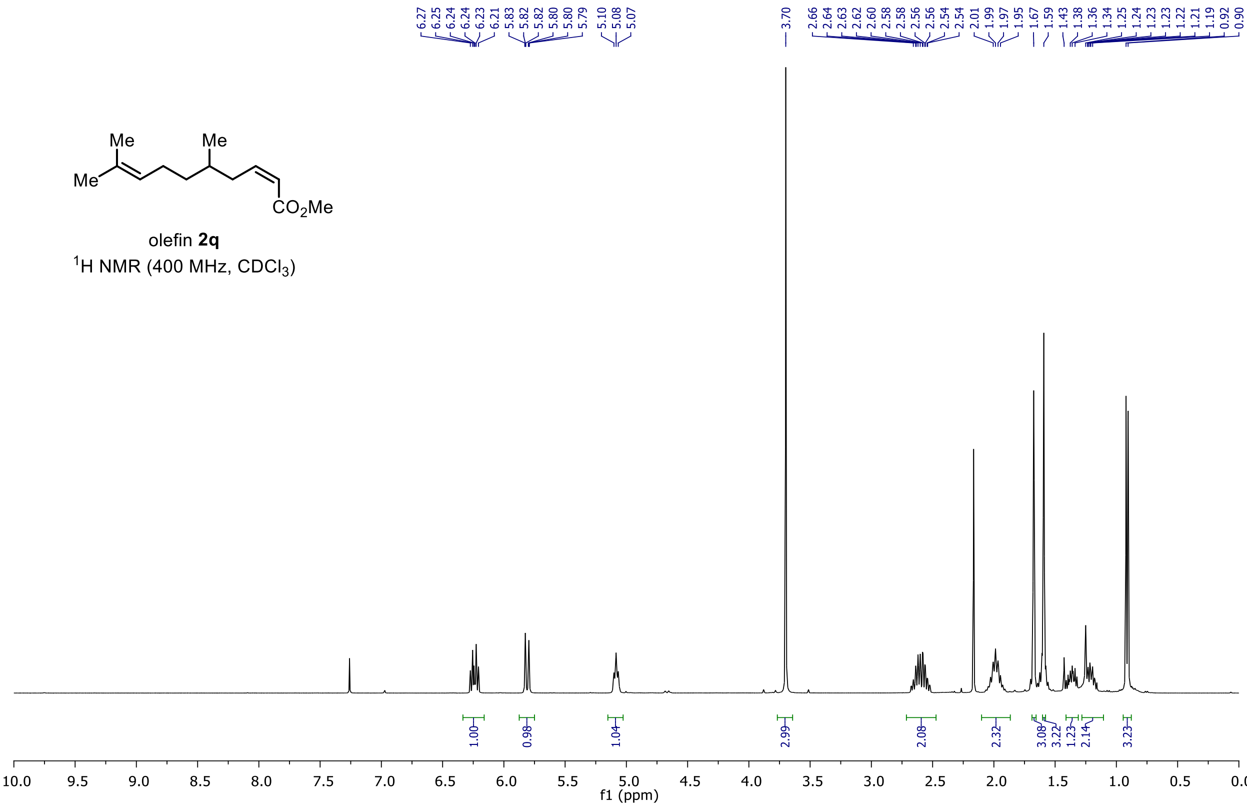
Me
Me
Me
CO2Me
olefin 2q
1H NMR (400 MHz, CDCl3)
6.27
6.25
6.24
6.24
6.23
6.21
5.83
5.82
5.82
5.80
5.80
5.79
5.10
5.08
5.07
3.70
2.66
2.64
2.63
2.62
2.60
2.58
2.58
2.56
2.56
2.54
2.54
2.01
1.99
1.97
1.95
1.67
1.59
1.43
1.38
1.36
1.34
1.25
1.24
1.23
1.23
1.22
1.21
1.19
0.92
0.90
1.00
0.98
1.04
2.99
2.08
2.32
3.08
3.22
1.23
2.14
3.23
f1 (ppm)

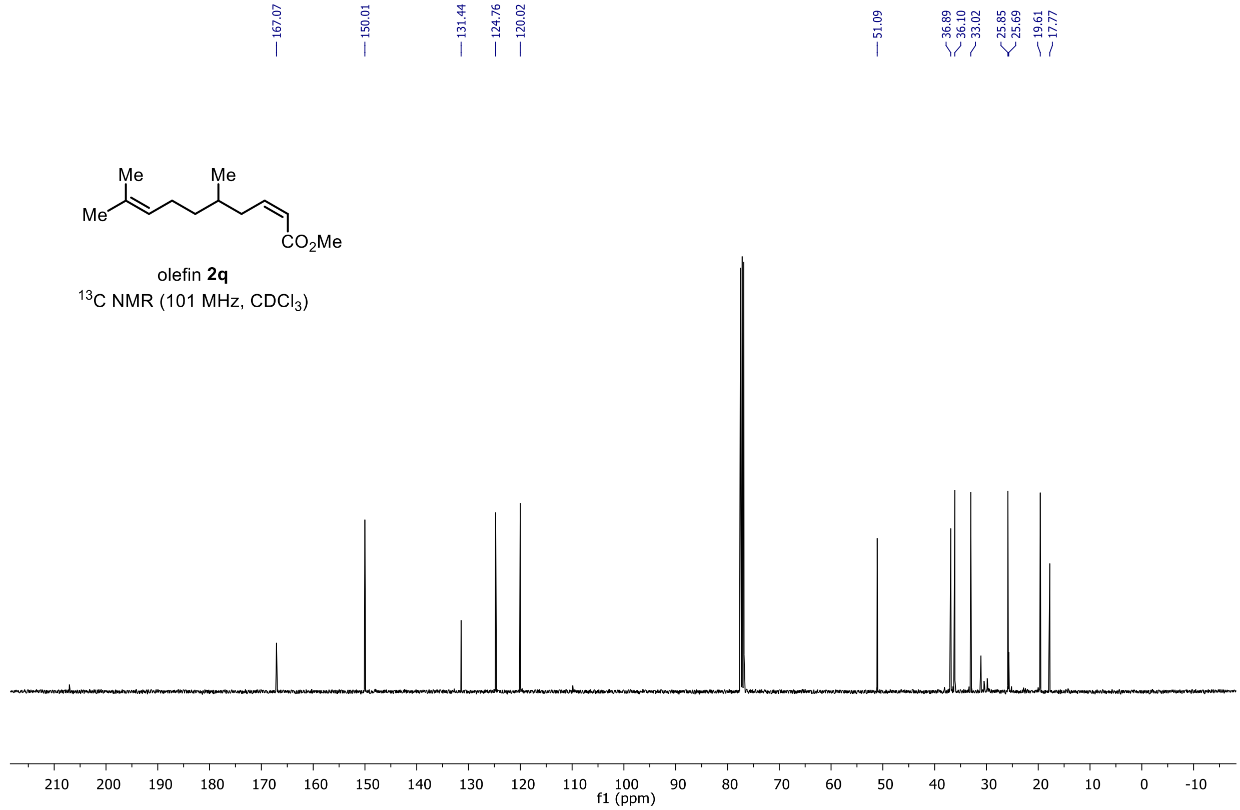
Me
Me
Me
CO2Me
olefin 2q
13C NMR (101 MHz, CDCl3)
167.07
150.01
131.44
124.76
120.02
51.09
36.89
36.10
33.02
25.85
25.69
19.61
17.77
f1 (ppm)

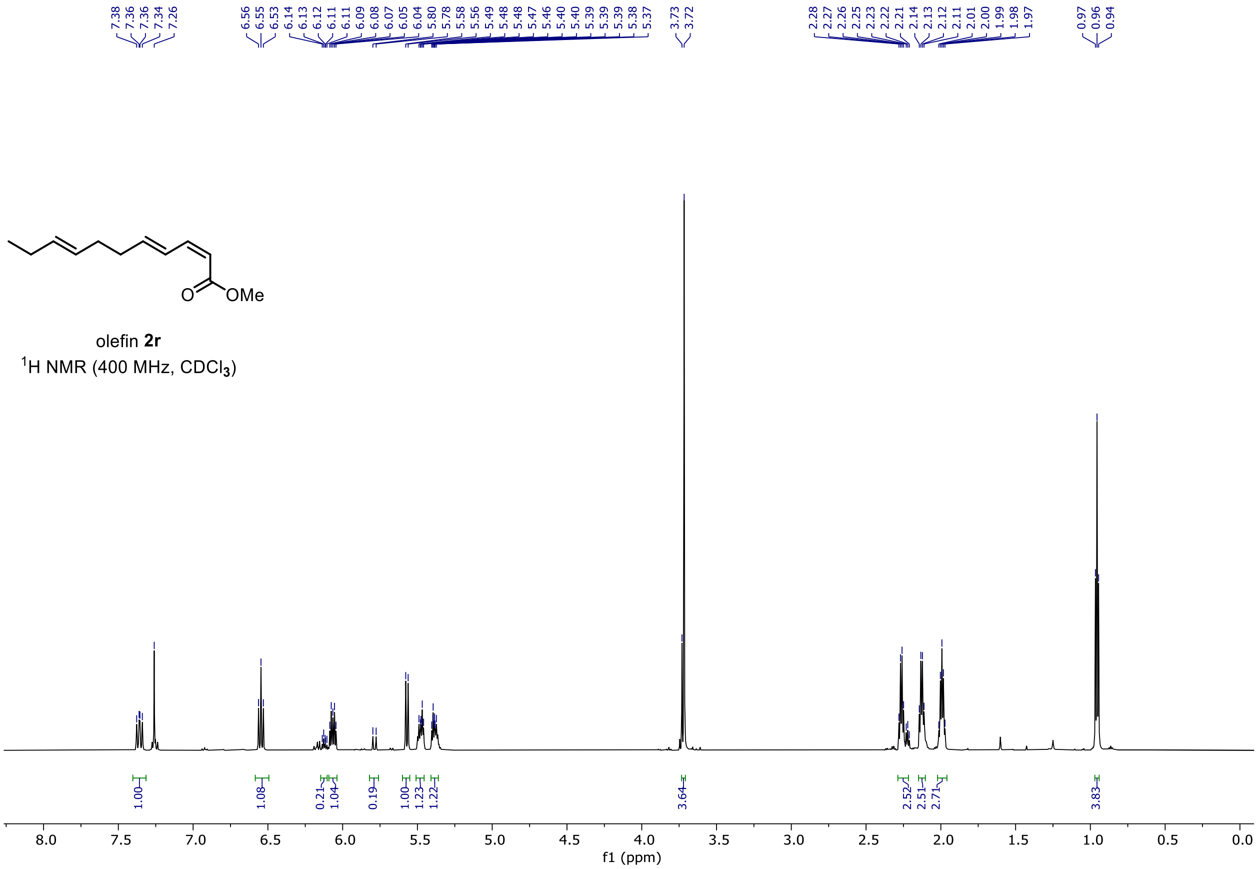
7.38 7.36 7.36 7.34 7.26
6.56 6.55 6.53 6.14 6.13 6.12 6.11 6.11 6.09 6.08 6.07 6.05 6.04 5.80 5.78 5.58 5.56 5.49 5.48 5.48 5.47 5.46 5.40 5.39 5.39 5.39 5.38 5.37
3.73 3.72
2.28 2.27 2.26 2.25 2.23 2.22 2.21 2.14 2.13 2.12 2.11 2.01 2.00 1.99 1.98 1.97
0.97 0.96 0.94
O
OMe
olefin 2r
1H NMR (400 MHz, CDCl3)
1.00 1.08 0.21 1.04 0.19 1.00 1.23 1.22 3.64 2.52 2.51 2.71 3.83
8.0 7.5 7.0 6.5 6.0 5.5 5.0 4.5 4.0 3.5 3.0 2.5 2.0 1.5 1.0 0.5 0.0
f1 (ppm)

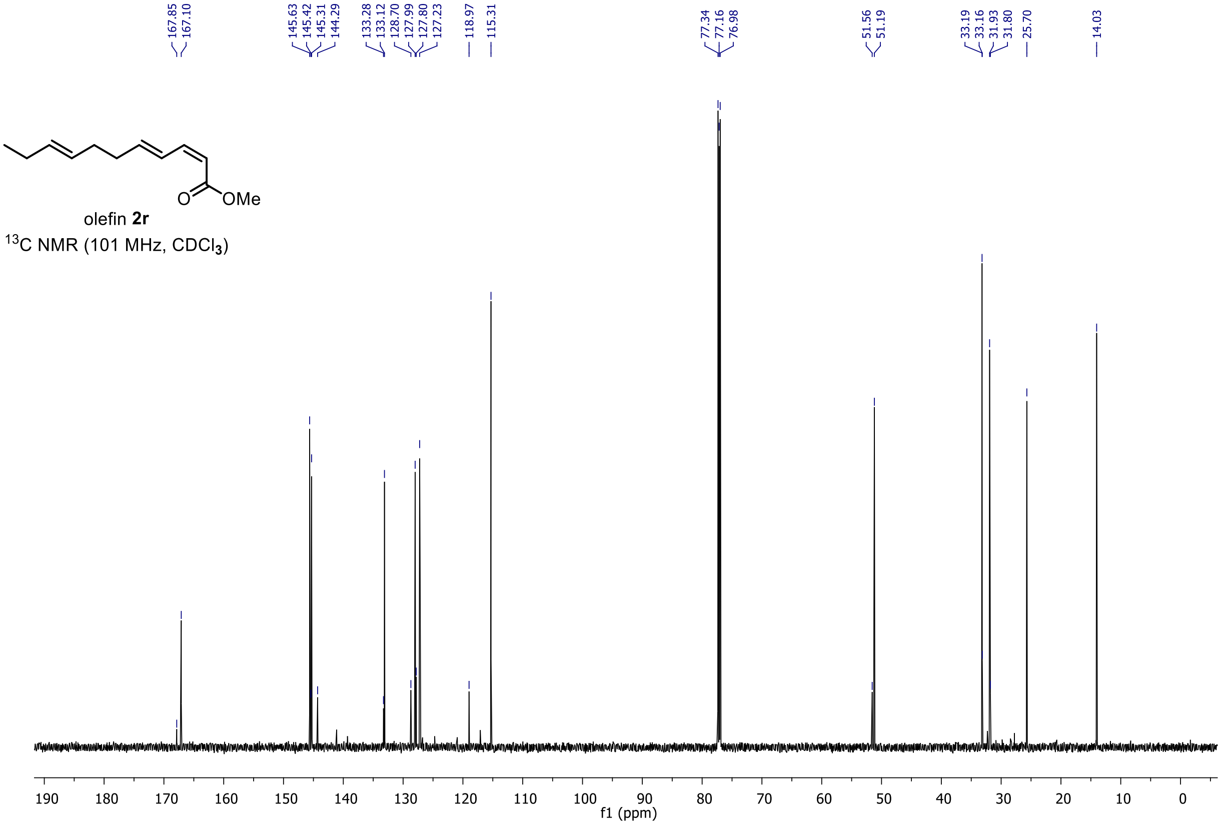
167.85 167.10
145.63 145.42 145.31 144.29
133.28 133.12 128.70 127.99 127.80 127.23
118.97
115.31
77.34 77.16 76.98
51.56 51.19
33.19 33.16 31.93 31.80
25.70
14.03
O
OMe
olefin 2r
13C NMR (101 MHz, CDCl3)
190 180 170 160 150 140 130 120 110 100 90 80 70 60 50 40 30 20 10 0
f1 (ppm)

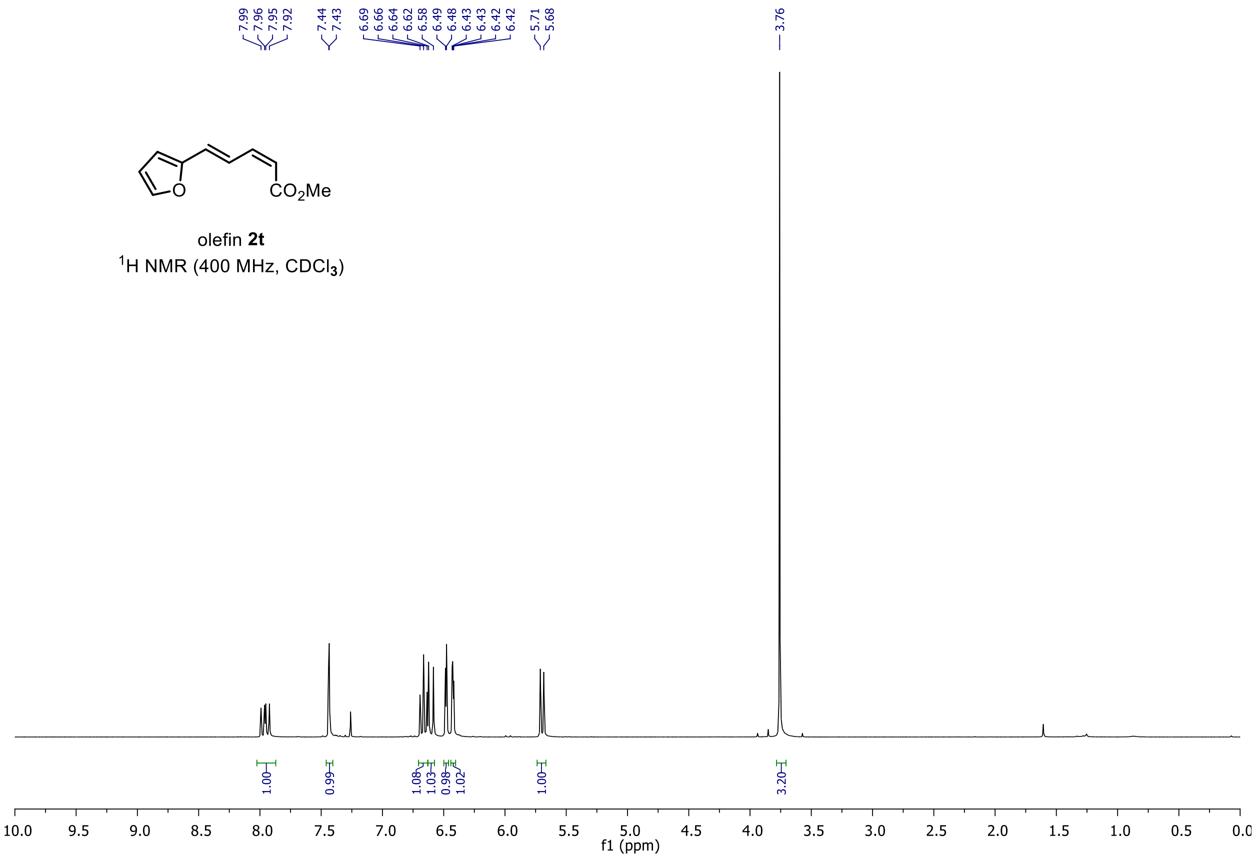
7.99
7.96
7.95
7.92
7.44
7.43
6.69
6.66
6.64
6.62
6.58
6.49
6.48
6.43
6.43
6.42
6.42
5.71
5.68
3.76
CO2Me
O
olefin 2t
1H NMR (400 MHz, CDCl3)
1.00
0.99
1.08
1.03
0.98
1.02
1.00
3.20
10.0 9.5 9.0 8.5 8.0 7.5 7.0 6.5 6.0 5.5 5.0 4.5 4.0 3.5 3.0 2.5 2.0 1.5 1.0 0.5 0.0
f1 (ppm)

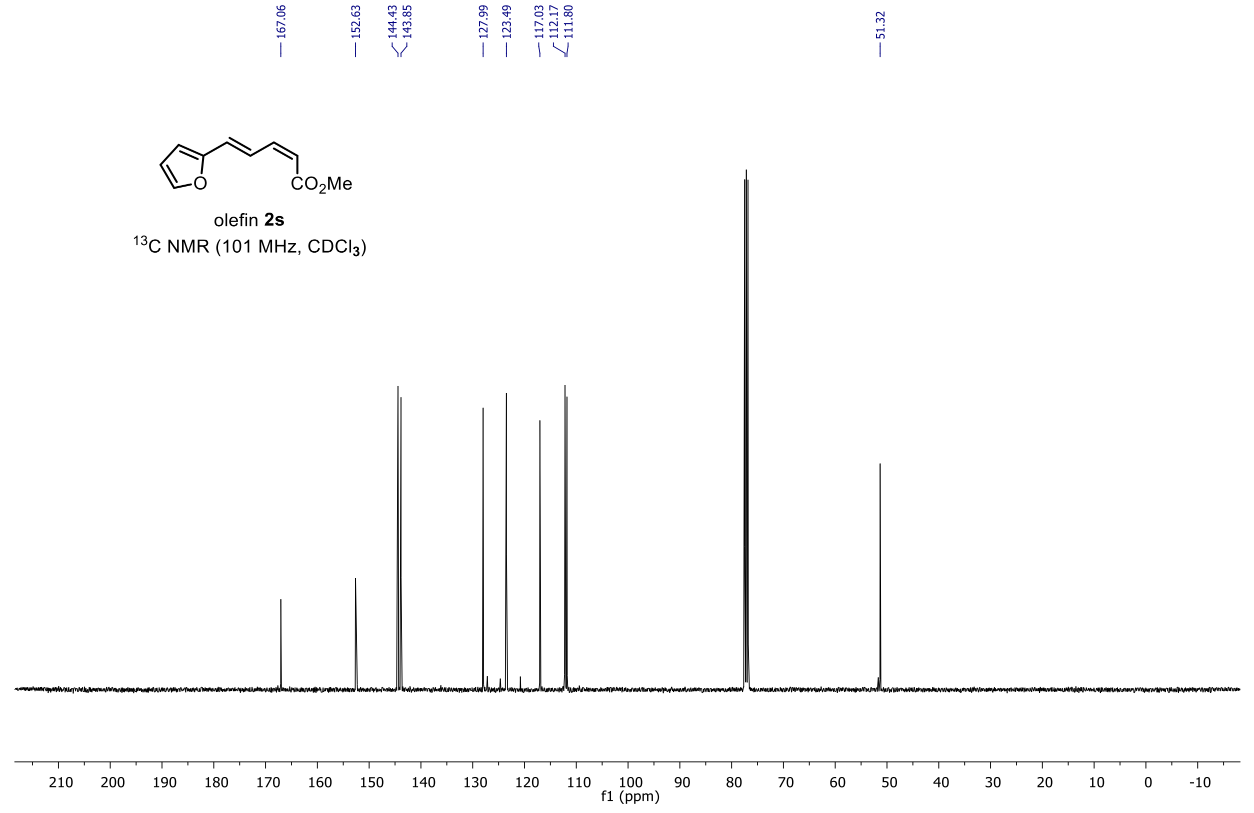
167.06
152.63
144.43
143.85
127.99
123.49
117.03
112.17
111.80
51.32
CO2Me
O
olefin 2s
13C NMR (101 MHz, CDCl3)
210 200 190 180 170 160 150 140 130 120 110 100 90 80 70 60 50 40 30 20 10 0 -10
f1 (ppm)

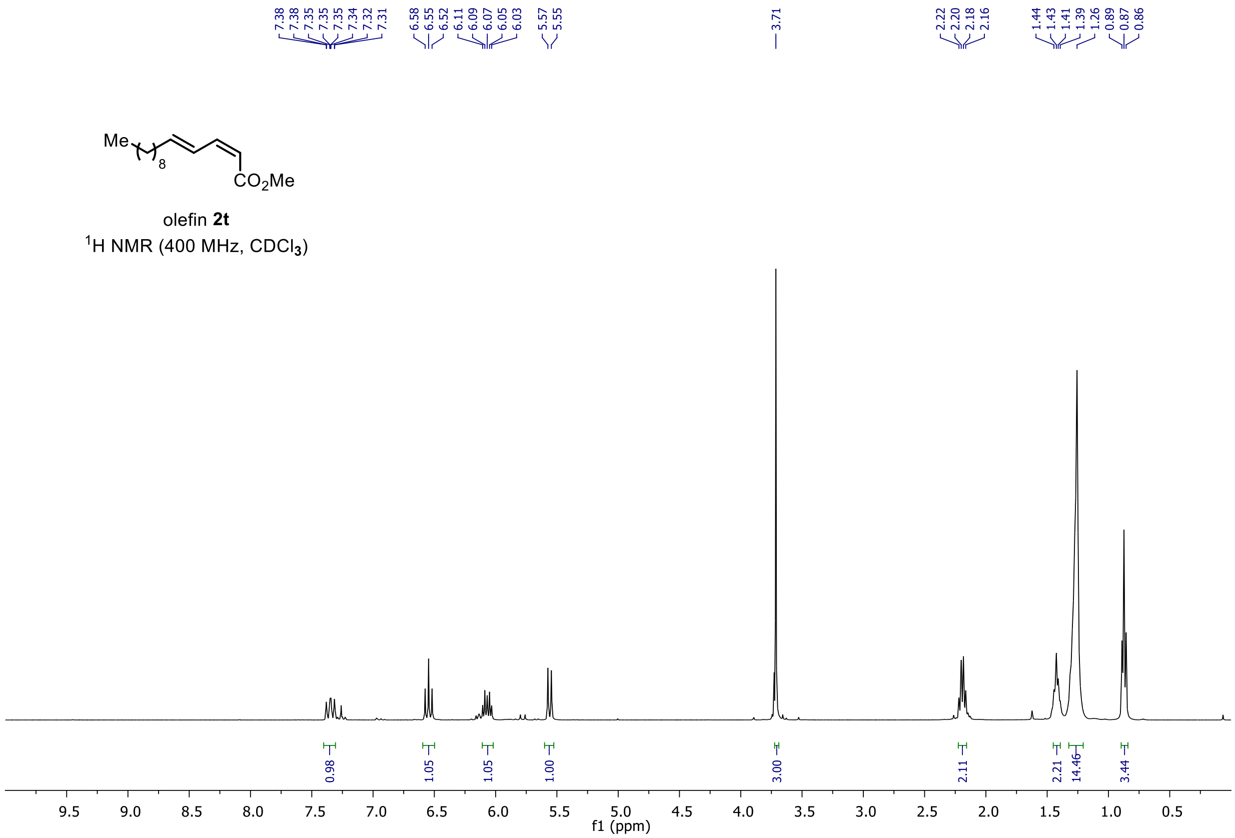

Me
8
CO2Me
olefin 2t
1H NMR (400 MHz, CDCl3)
7.38 7.38 7.35 7.35 7.35 7.34 7.32 7.31
6.58 6.55 6.52 6.11 6.09 6.07 6.05 6.03
5.57 5.55
3.71
2.22 2.20 2.18 2.16
1.44 1.43 1.41 1.39 1.26 0.89 0.87 0.86
0.98 1.05 1.05 1.00 3.00 2.11 2.21 14.46 3.44
f1 (ppm)

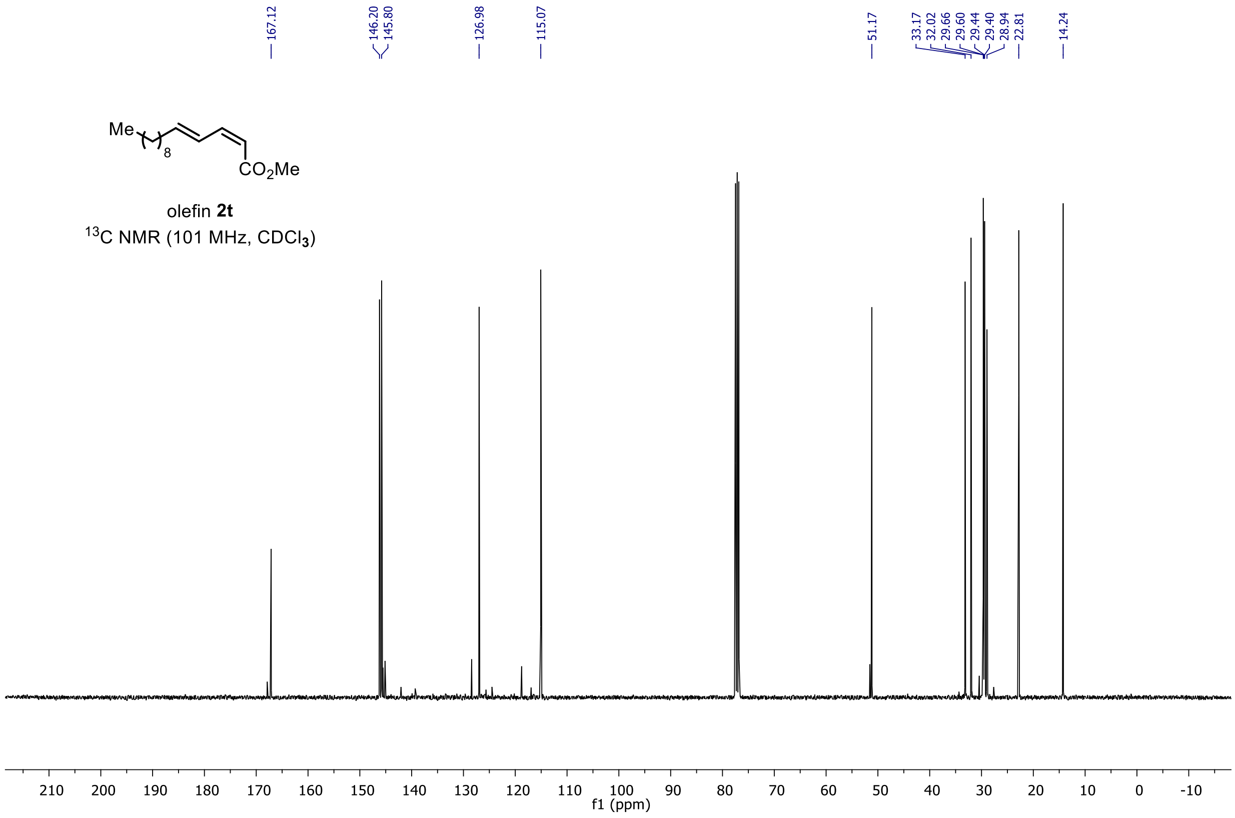

Me
8
CO2Me
olefin 2t
13C NMR (101 MHz, CDCl3)
167.12
146.20 145.80
126.98
115.07
51.17
33.17 32.02 29.66 29.60 29.44 29.40 28.94 22.81
14.24
f1 (ppm)

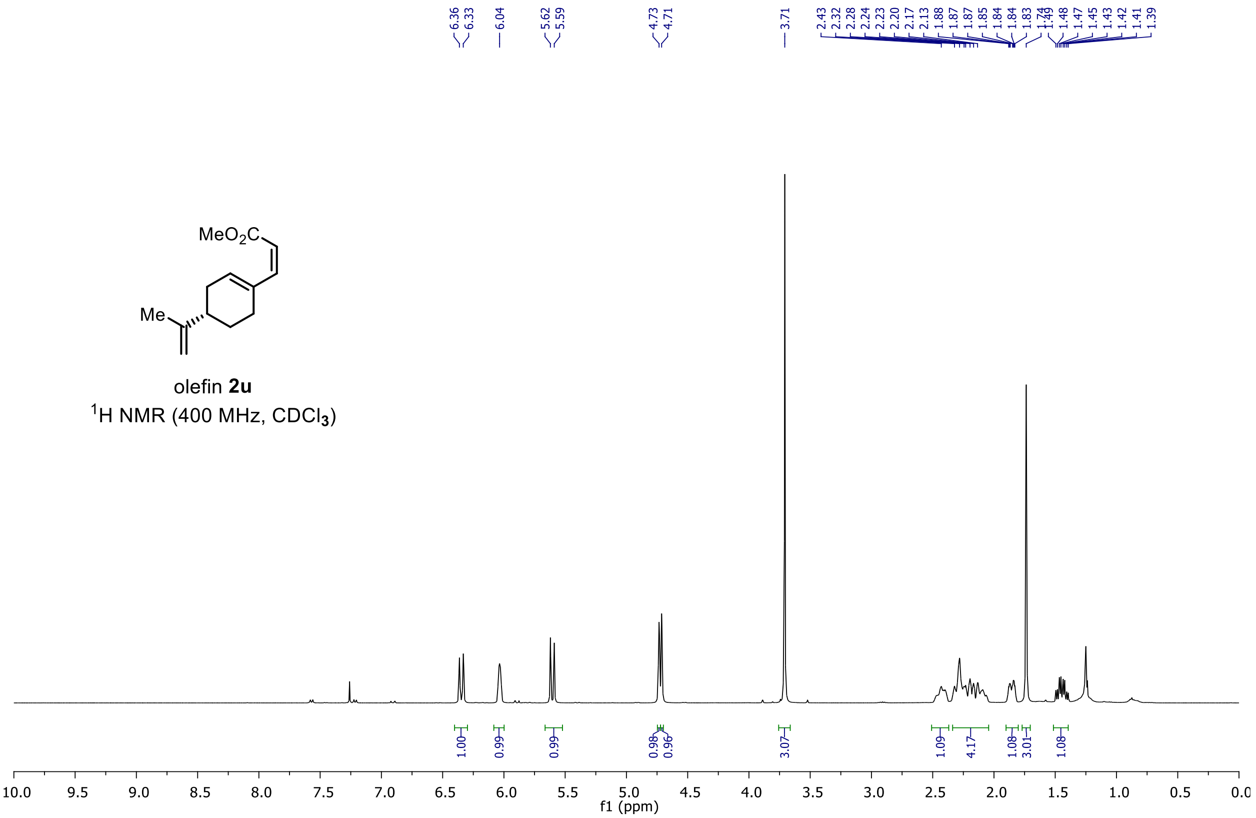

olefin **2u**

^1H NMR (400 MHz, $CDCl_3$)

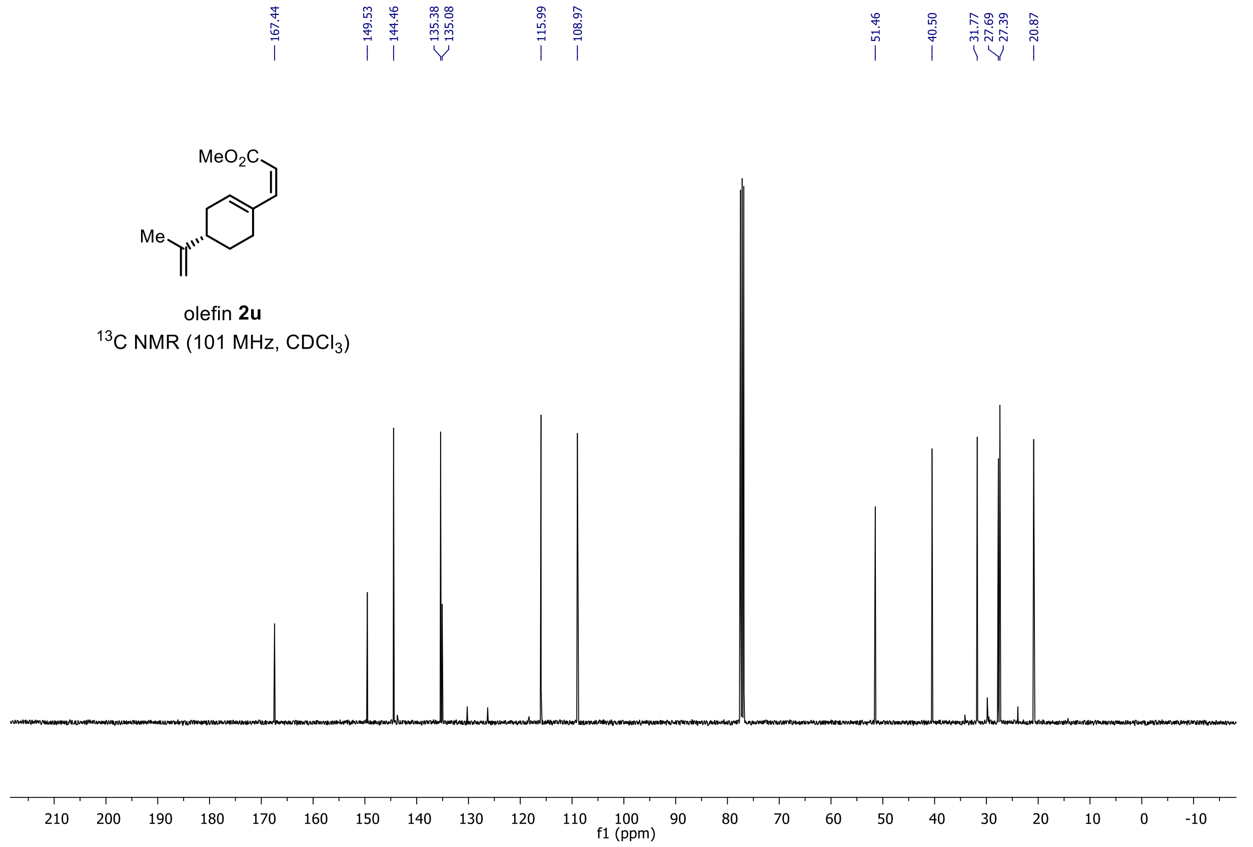

olefin **2u**

^{13}C NMR (101 MHz, $CDCl_3$)

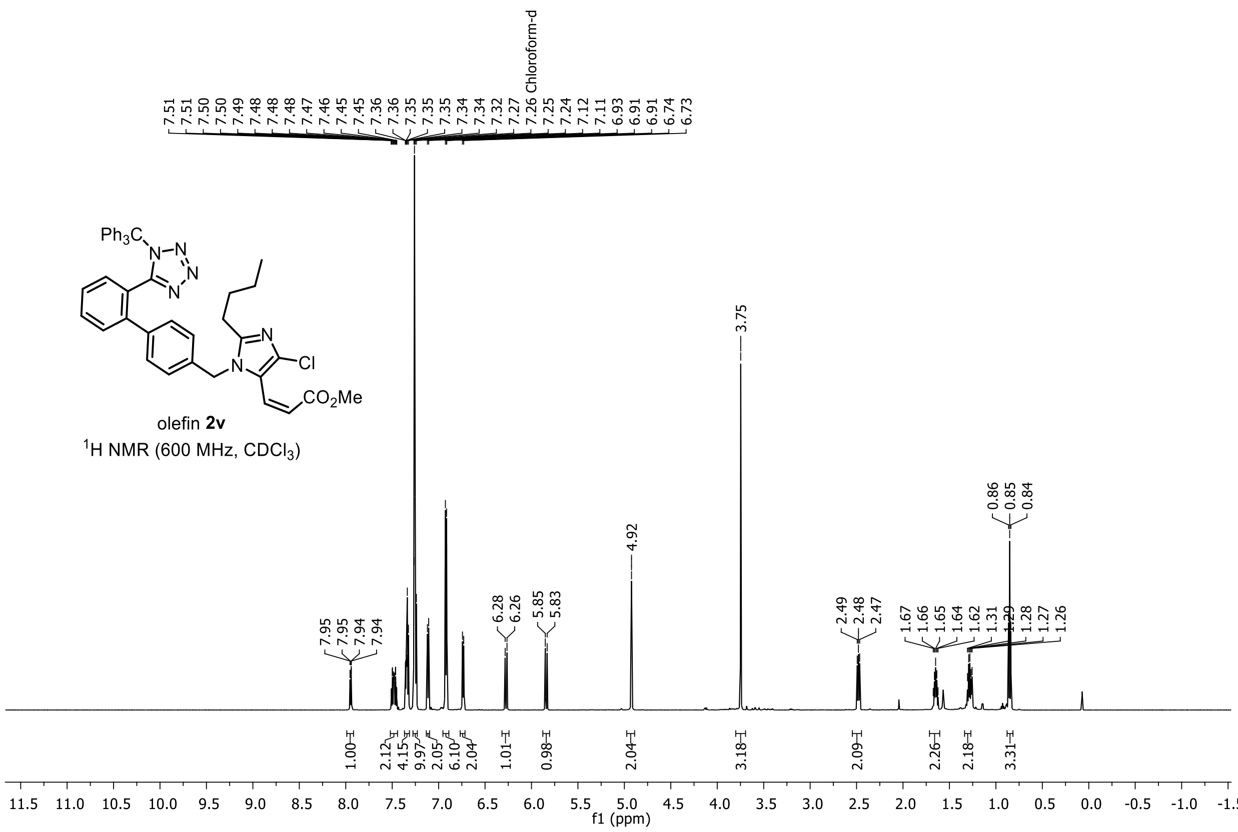
olefin **2v**
^1H NMR (600 MHz, $CDCl_3$)

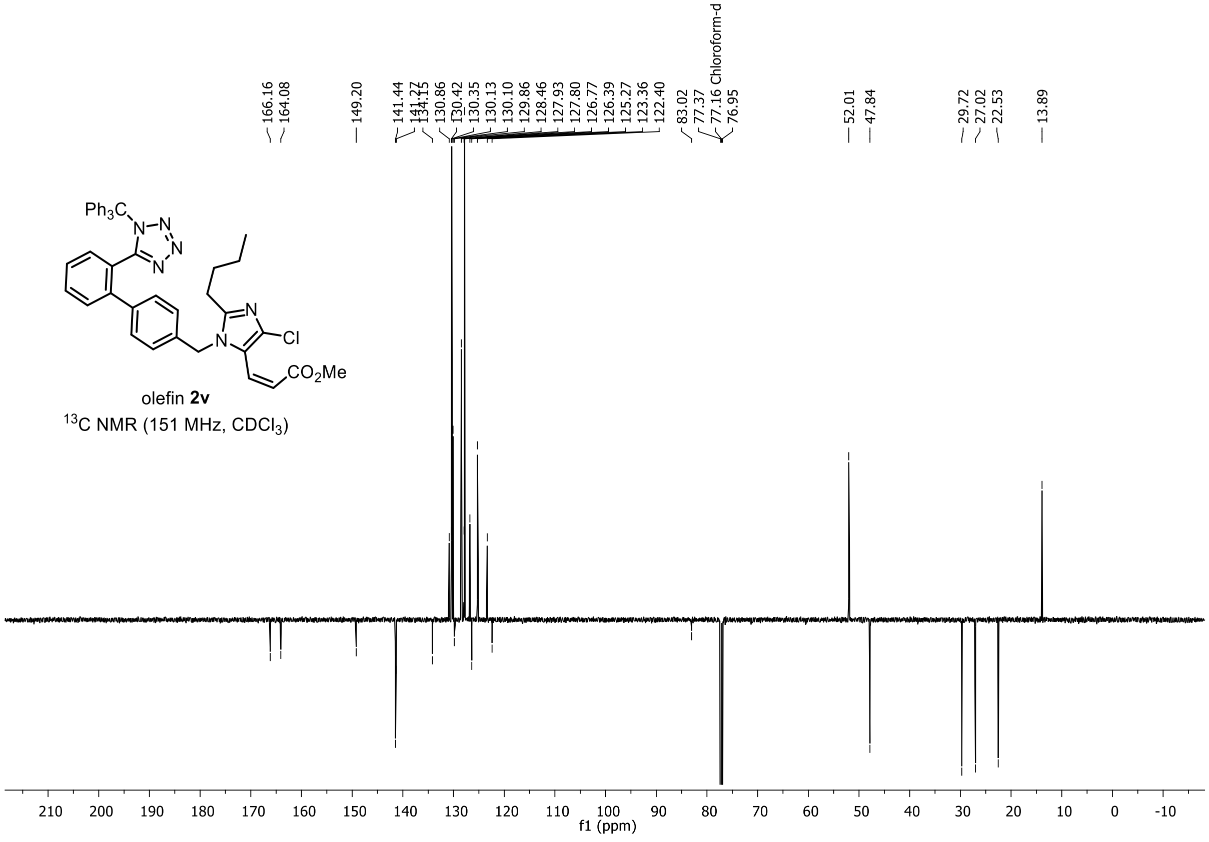
olefin **2v**
^{13}C NMR (151 MHz, $CDCl_3$)

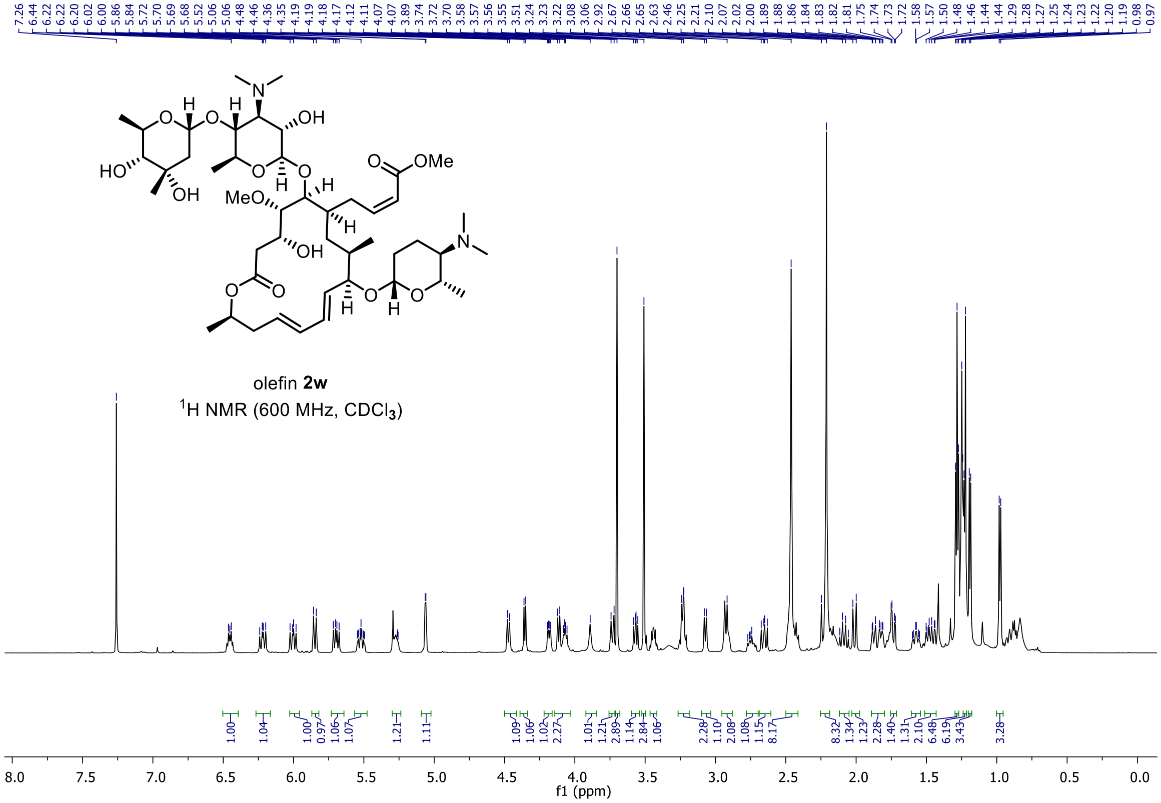
olefin **2w**
^{1}H NMR (600 MHz, $CDCl_3$)
f1 (ppm)

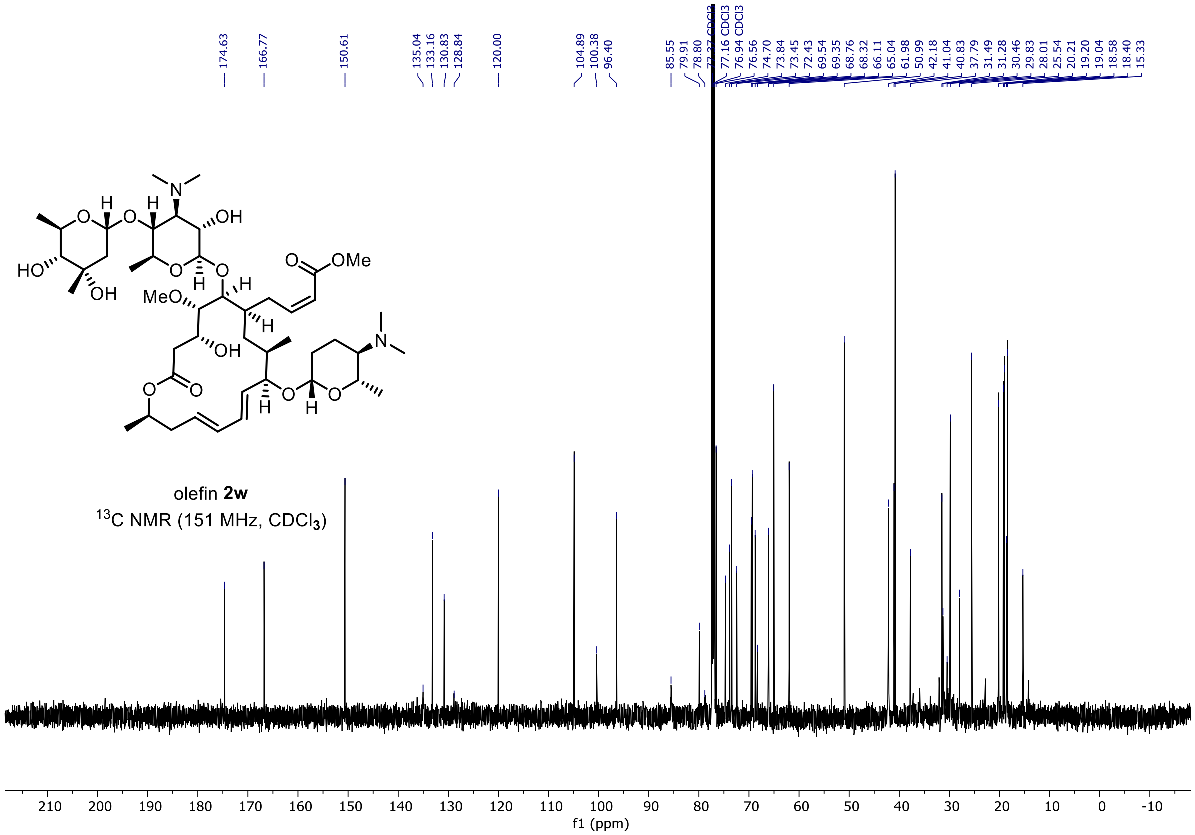
olefin **2w**
^{13}C NMR (151 MHz, $CDCl_3$)
f1 (ppm)

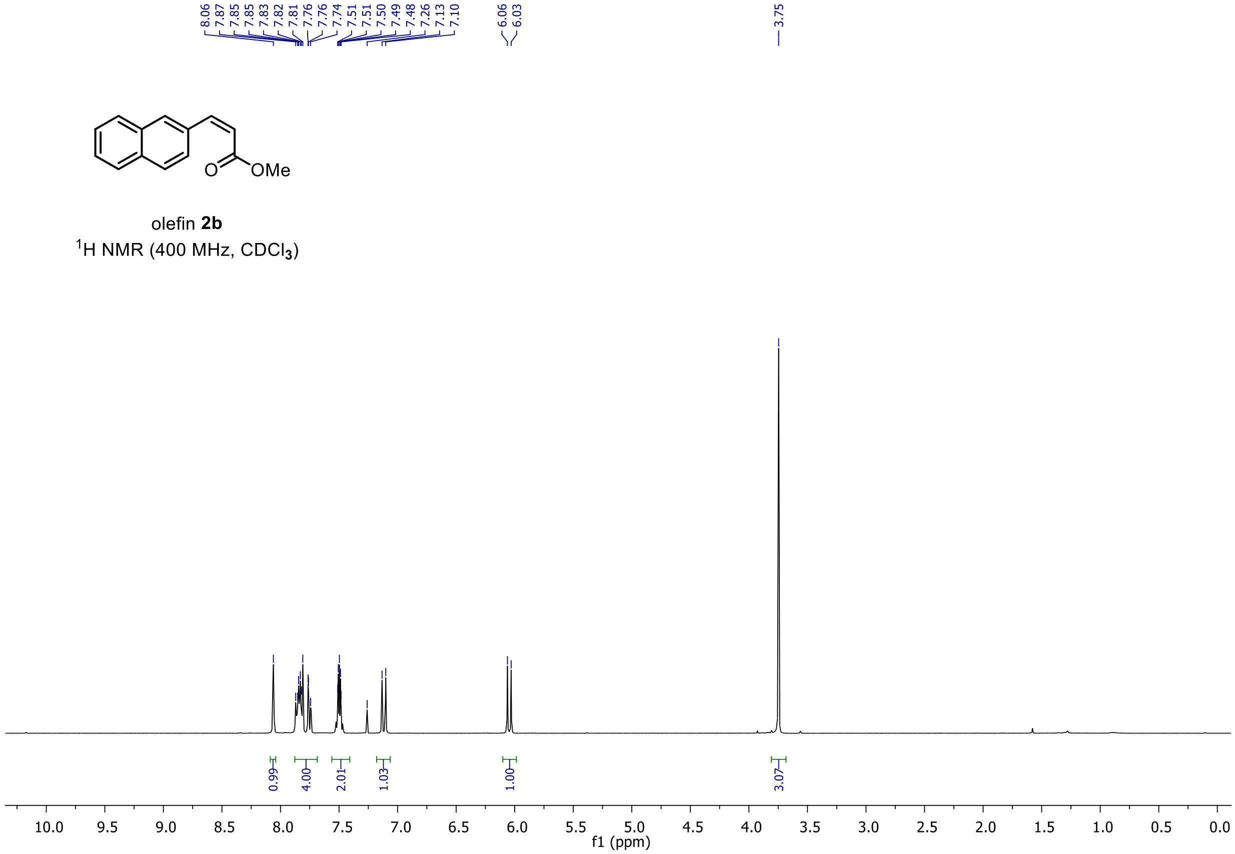

olefin **2b**

^{1}H NMR (400 MHz, $CDCl_3$)

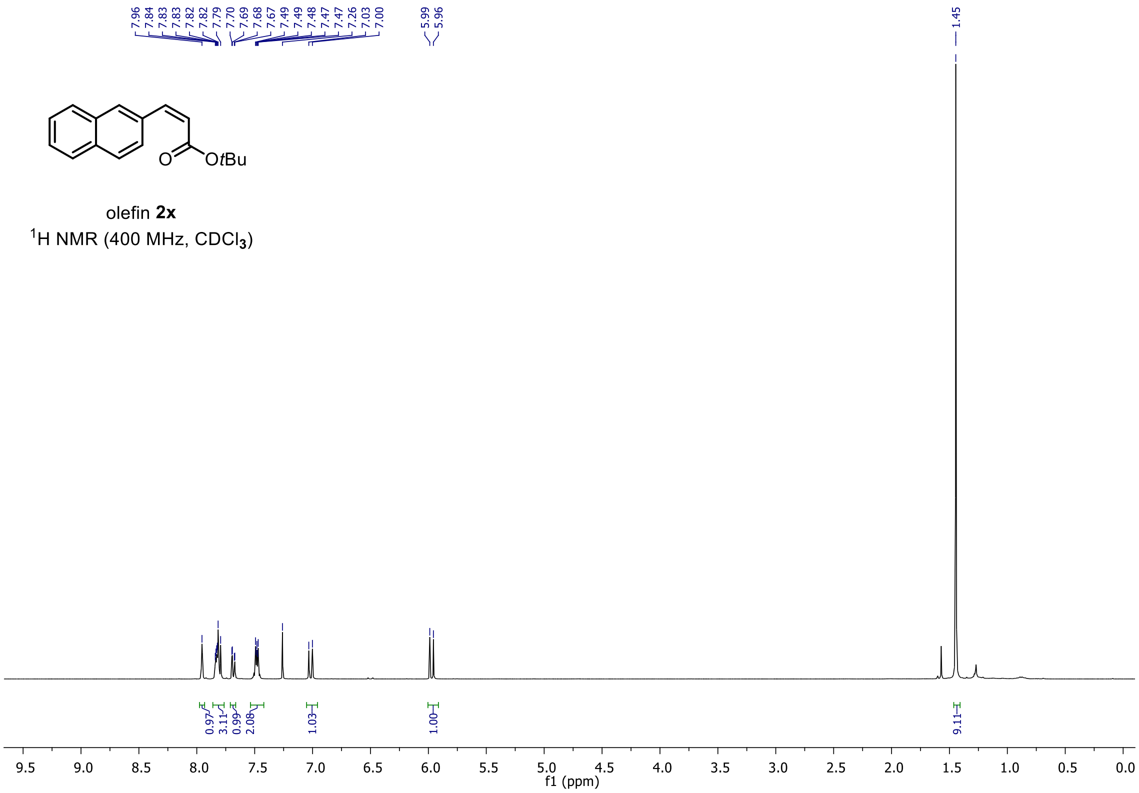

olefin **2x**
^{1}H NMR (400 MHz, $CDCl_3$)

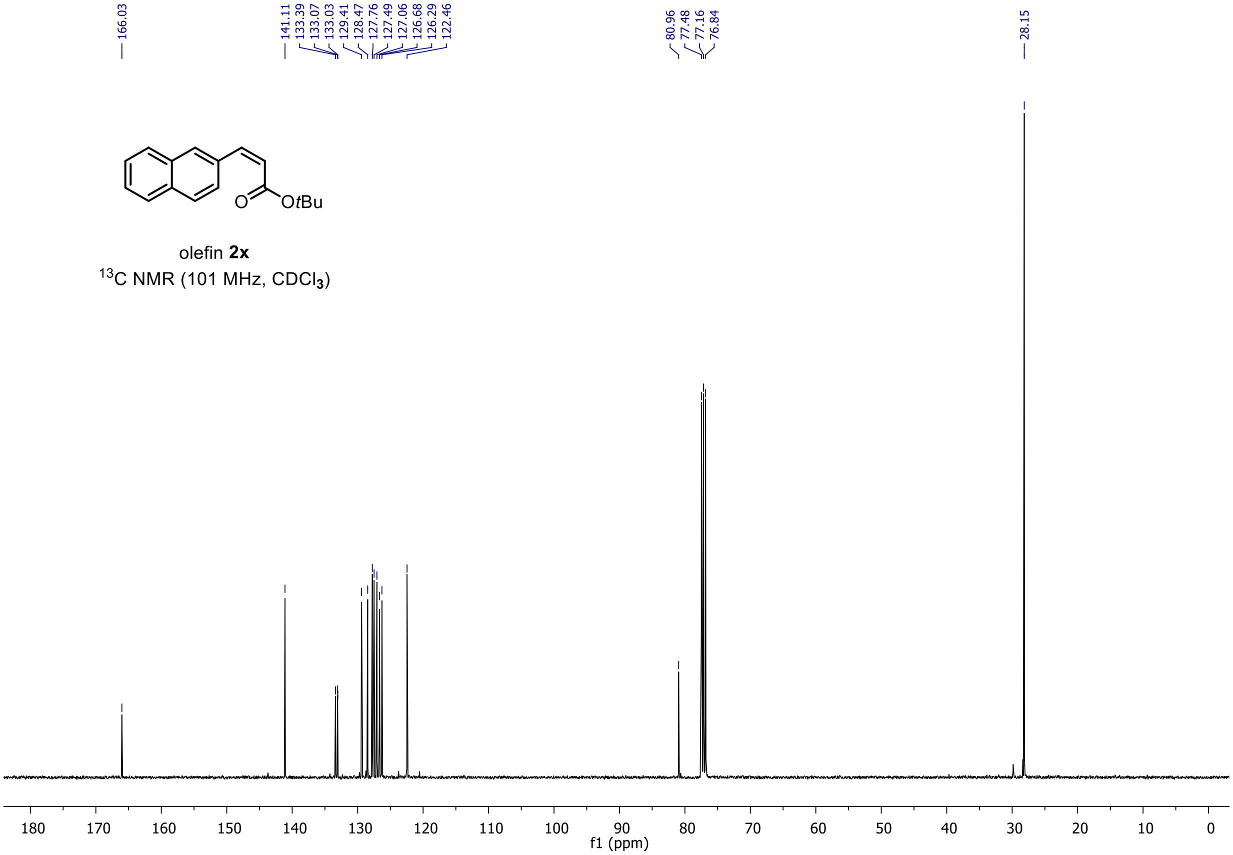

olefin **2x**
^{13}C NMR (101 MHz, $CDCl_3$)

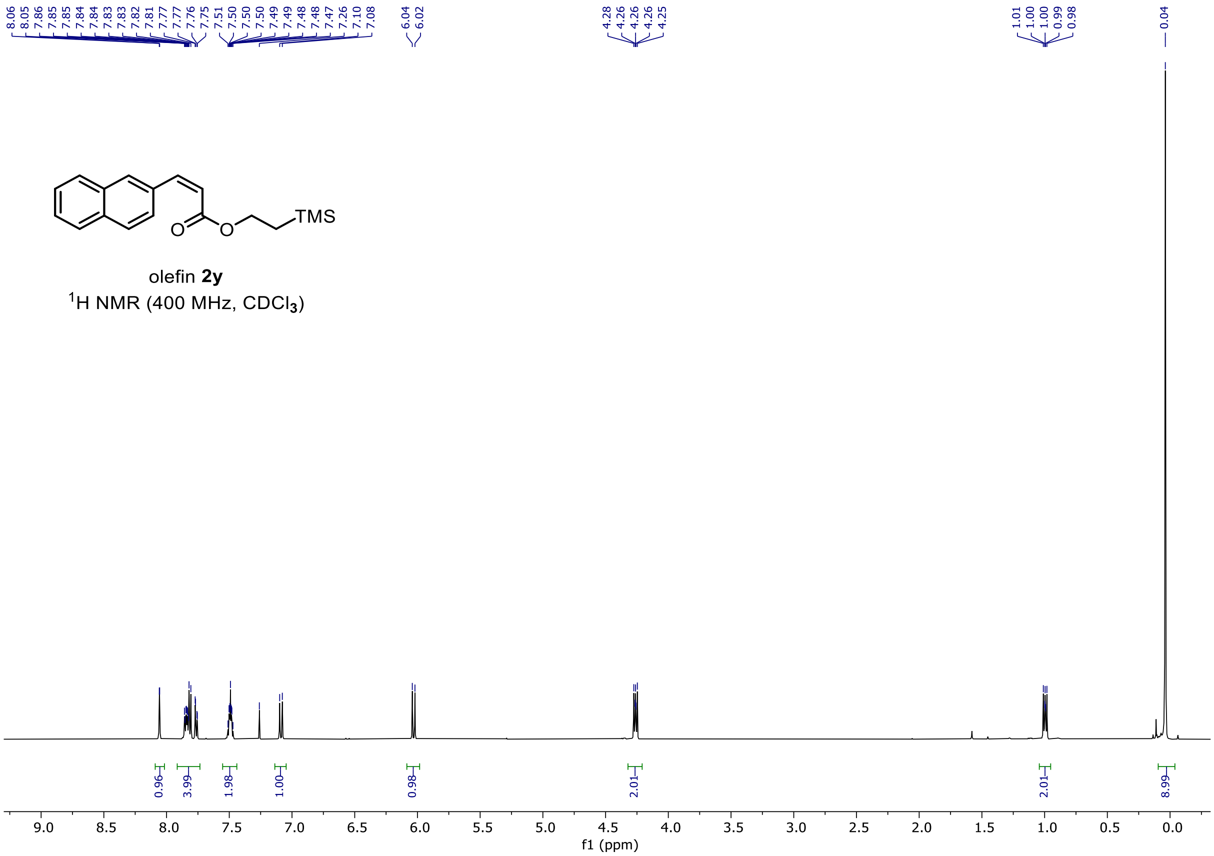
8.06 8.05 7.86 7.85 7.85 7.84 7.84 7.83 7.83 7.82 7.81 7.77 7.77 7.76 7.75 7.51 7.50 7.50 7.50 7.49 7.49 7.48 7.48 7.47 7.26 7.10 7.08
6.04 6.02
4.28 4.26 4.26 4.26 4.25
1.01 1.00 1.00 0.99 0.98
0.04
TMS
O
O
olefin **2y**
^{1}H NMR (400 MHz, $CDCl_3$)
0.96 3.99 1.98 1.00 0.98 2.01 2.01 8.99
9.0 8.5 8.0 7.5 7.0 6.5 6.0 5.5 5.0 4.5 4.0 3.5 3.0 2.5 2.0 1.5 1.0 0.5 0.0
f1 (ppm)

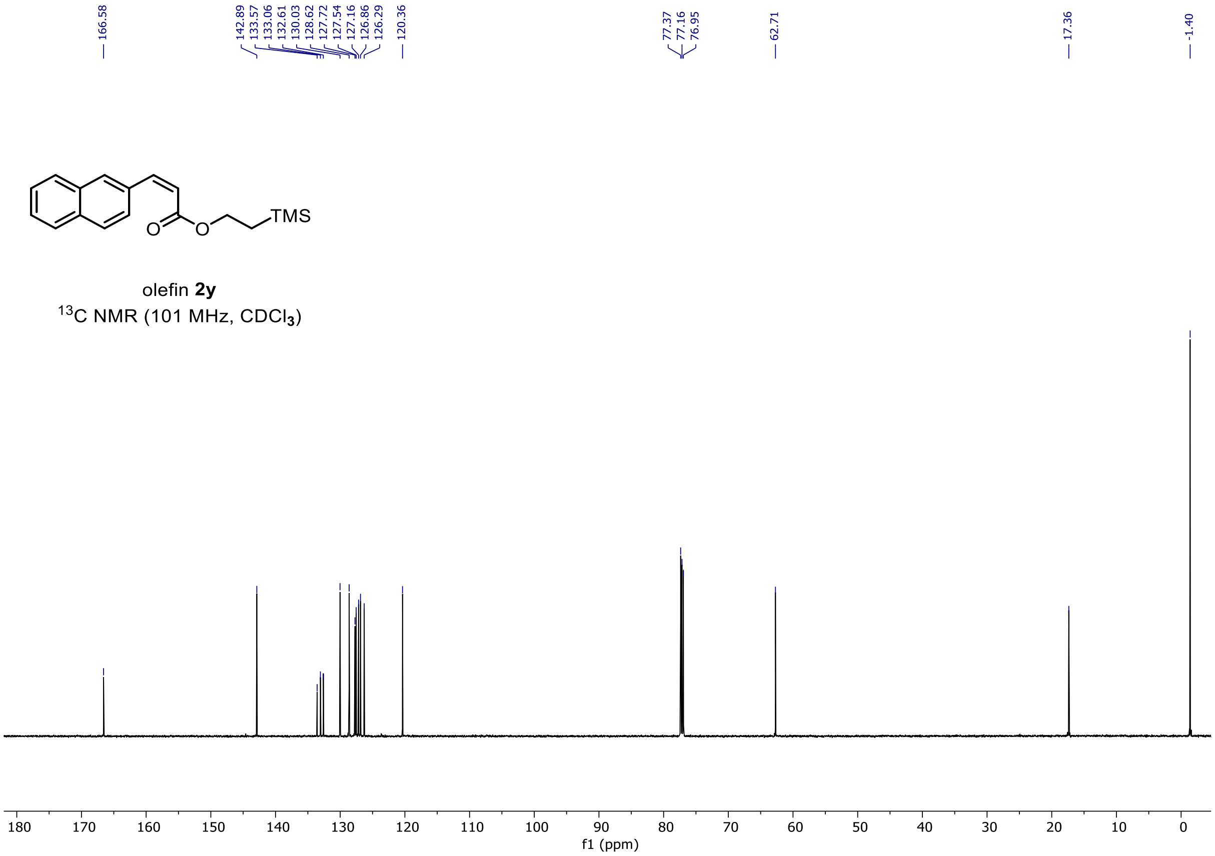
166.58
142.89 133.57 133.06 132.61 130.03 128.62 127.72 127.54 127.16 126.86 126.29 120.36
77.37 77.16 76.95
62.71
17.36
-1.40
TMS
O
O
olefin **2y**
^{13}C NMR (101 MHz, $CDCl_3$)
180 170 160 150 140 130 120 110 100 90 80 70 60 50 40 30 20 10 0
f1 (ppm)

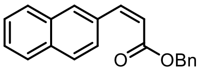

olefin **2z**

^{1}H NMR (600 MHz, $CDCl_3$)

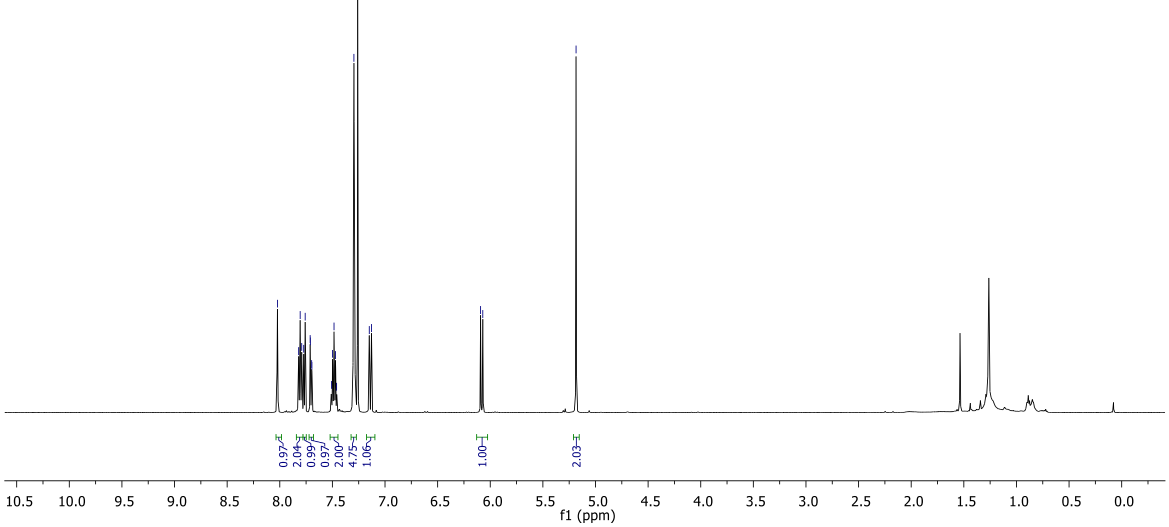

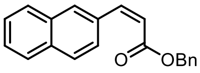

olefin **2z**

^{13}C NMR (151 MHz, $CDCl_3$)

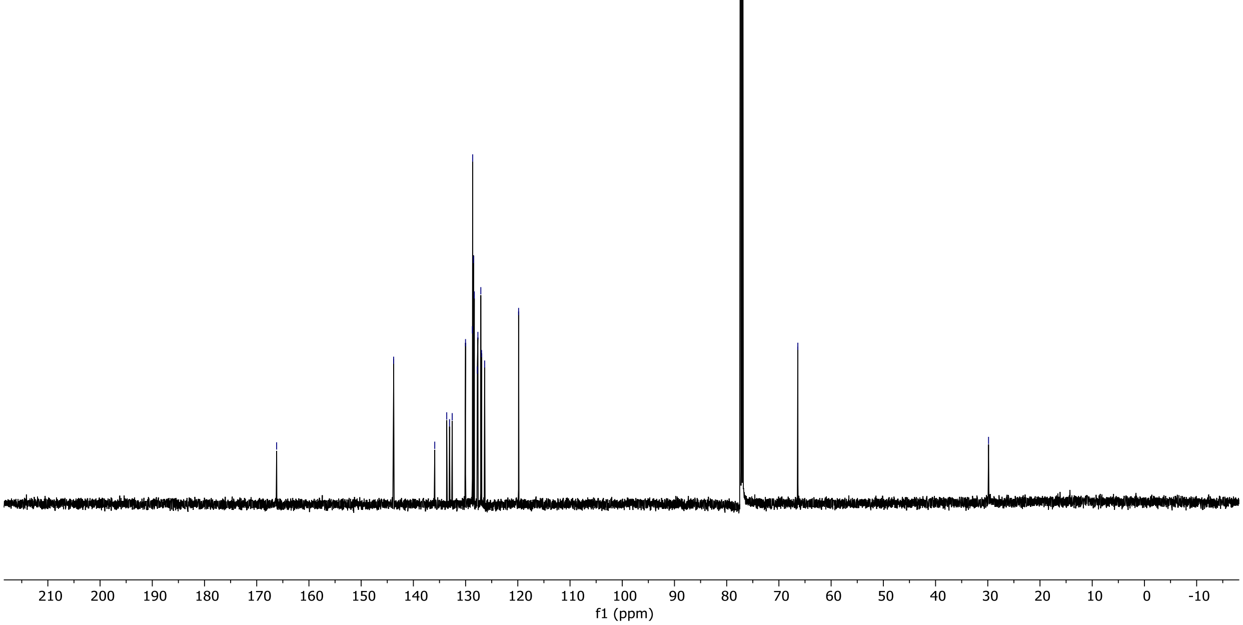

8.5 *E*-olefins

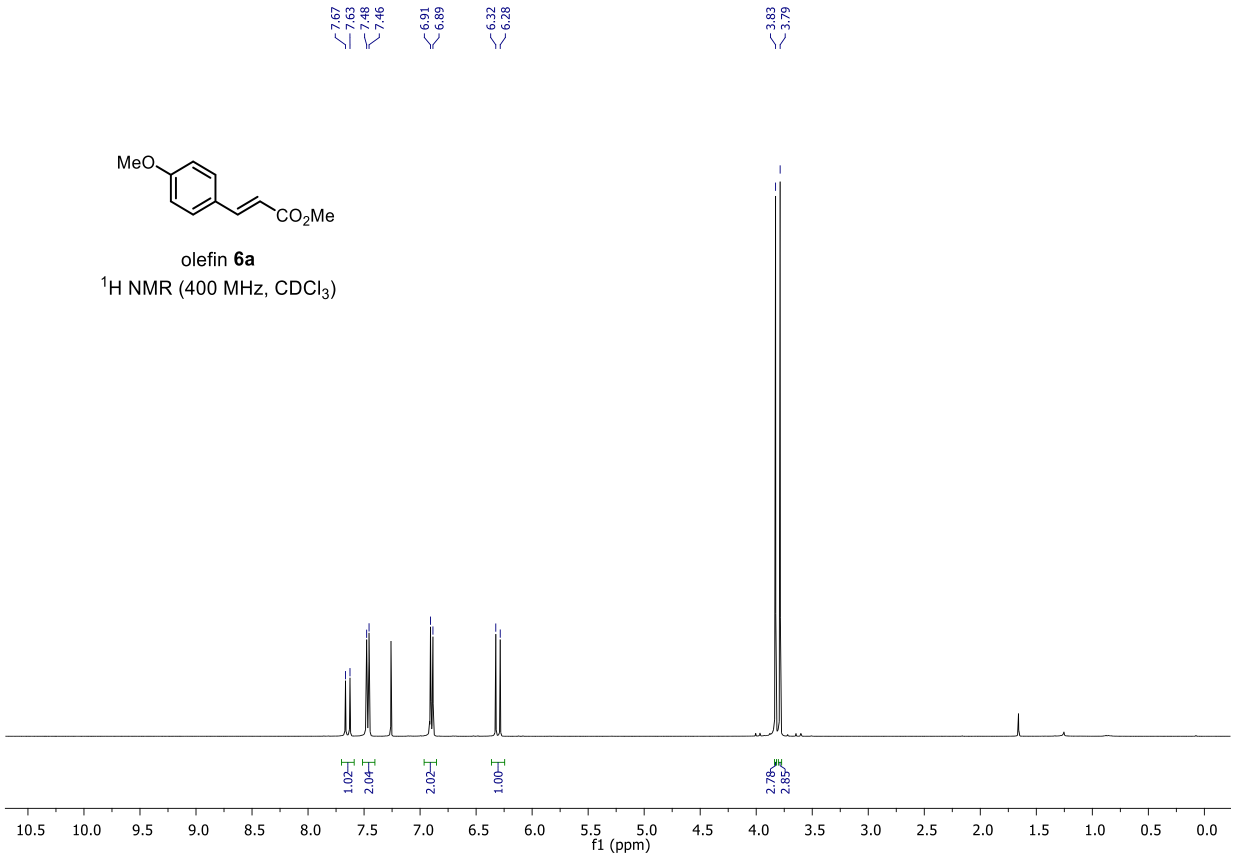

7.67
7.63
7.48
7.46
6.91
6.89
6.32
6.28
3.83
3.79
MeO
CO2Me
olefin 6a
1H NMR (400 MHz, CDCl3)
1.02
2.04
2.02
1.00
2.78
2.85
10.5 10.0 9.5 9.0 8.5 8.0 7.5 7.0 6.5 6.0 5.5 5.0 4.5 4.0 3.5 3.0 2.5 2.0 1.5 1.0 0.5 0.0
f1 (ppm)

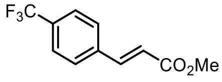

olefin **6b**

^{1}H NMR (400 MHz, $CDCl_3$)

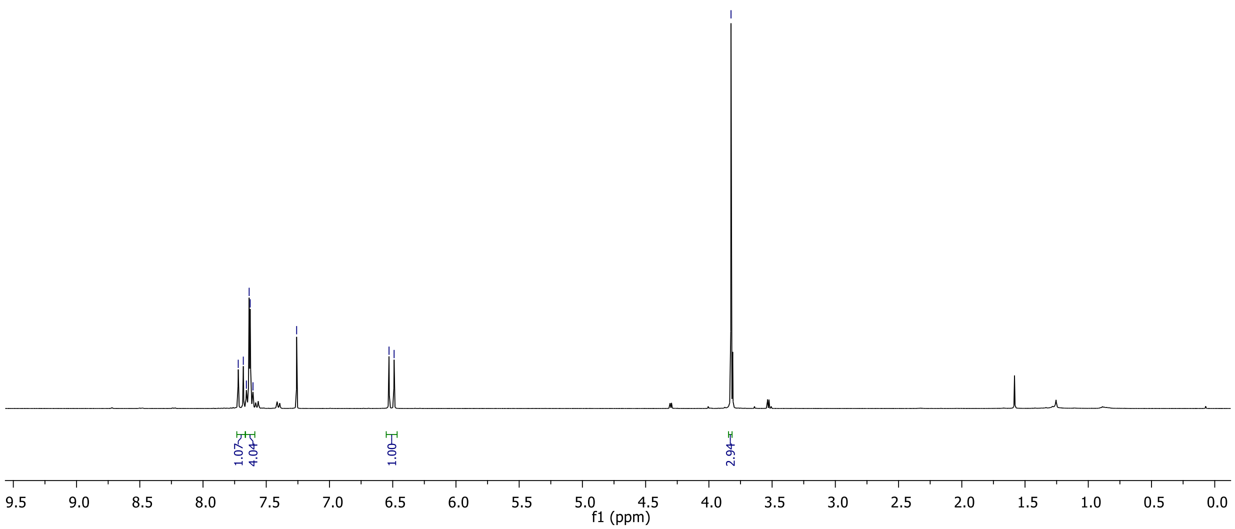

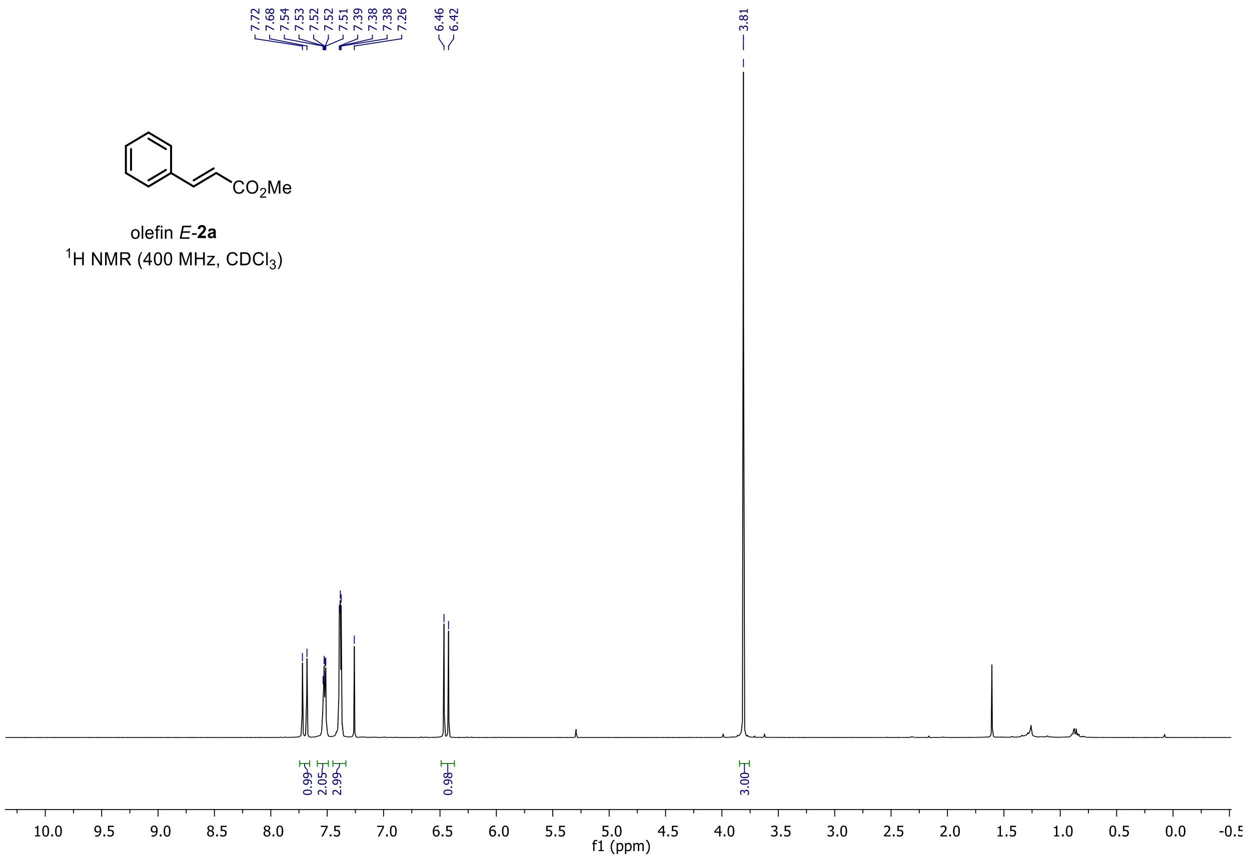

7.72
7.68
7.54
7.53
7.52
7.52
7.51
7.39
7.38
7.38
7.26
6.46
6.42
3.81
CO_2Me
olefin *E*-**2a**
^{1}H NMR (400 MHz, $CDCl_3$)
0.99
2.05
2.99
0.98
3.00
10.0
9.5
9.0
8.5
8.0
7.5
7.0
6.5
6.0
5.5
5.0
4.5
4.0
3.5
3.0
2.5
2.0
1.5
1.0
0.5
0.0
-0.5
f1 (ppm)

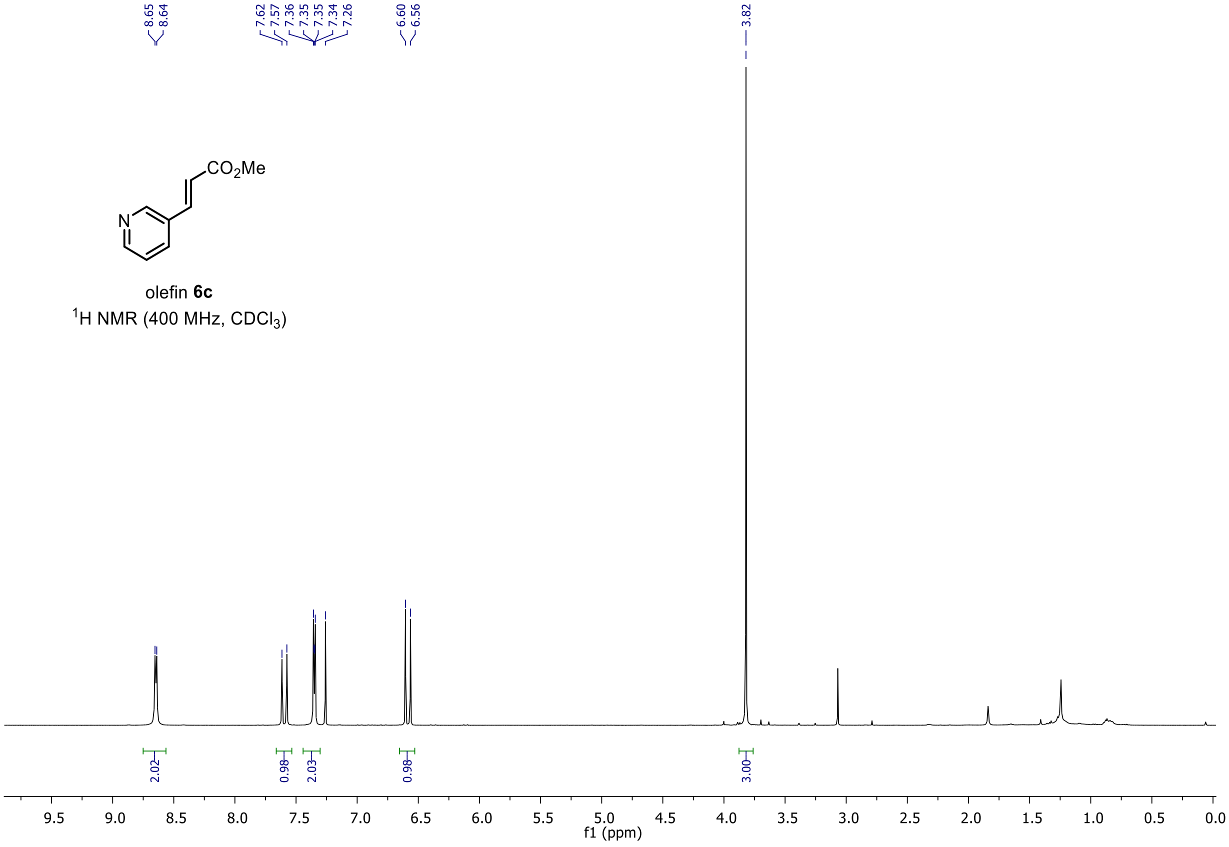
8.65
8.64
7.62
7.57
7.36
7.35
7.35
7.34
7.26
6.60
6.56
3.82
CO2Me
N
olefin 6c
1H NMR (400 MHz, CDCl3)
2.02
0.98
2.03
0.98
3.00
9.5 9.0 8.5 8.0 7.5 7.0 6.5 6.0 5.5 5.0 4.5 4.0 3.5 3.0 2.5 2.0 1.5 1.0 0.5 0.0
f1 (ppm)

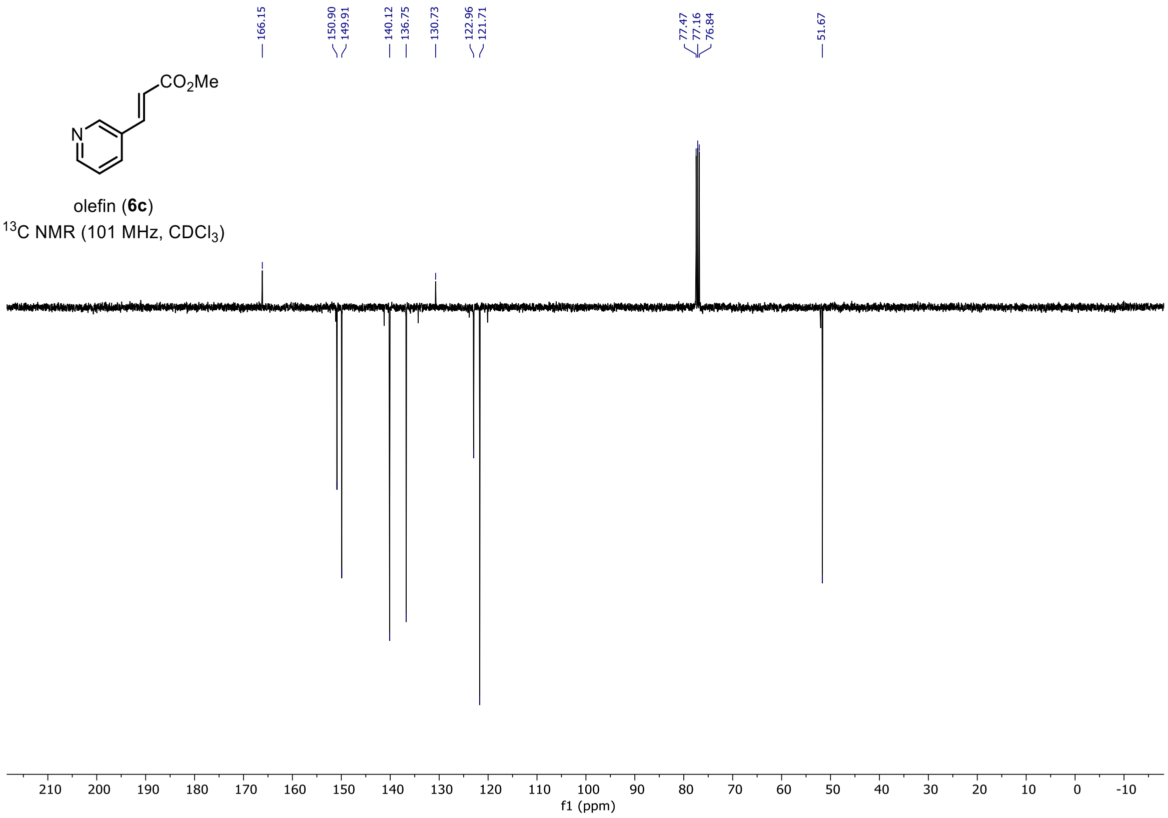
166.15
150.90
149.91
140.12
136.75
130.73
122.96
121.71
77.47
77.16
76.84
51.67
CO2Me
N
olefin (6c)
13C NMR (101 MHz, CDCl3)
210 200 190 180 170 160 150 140 130 120 110 100 90 80 70 60 50 40 30 20 10 0 -10
f1 (ppm)

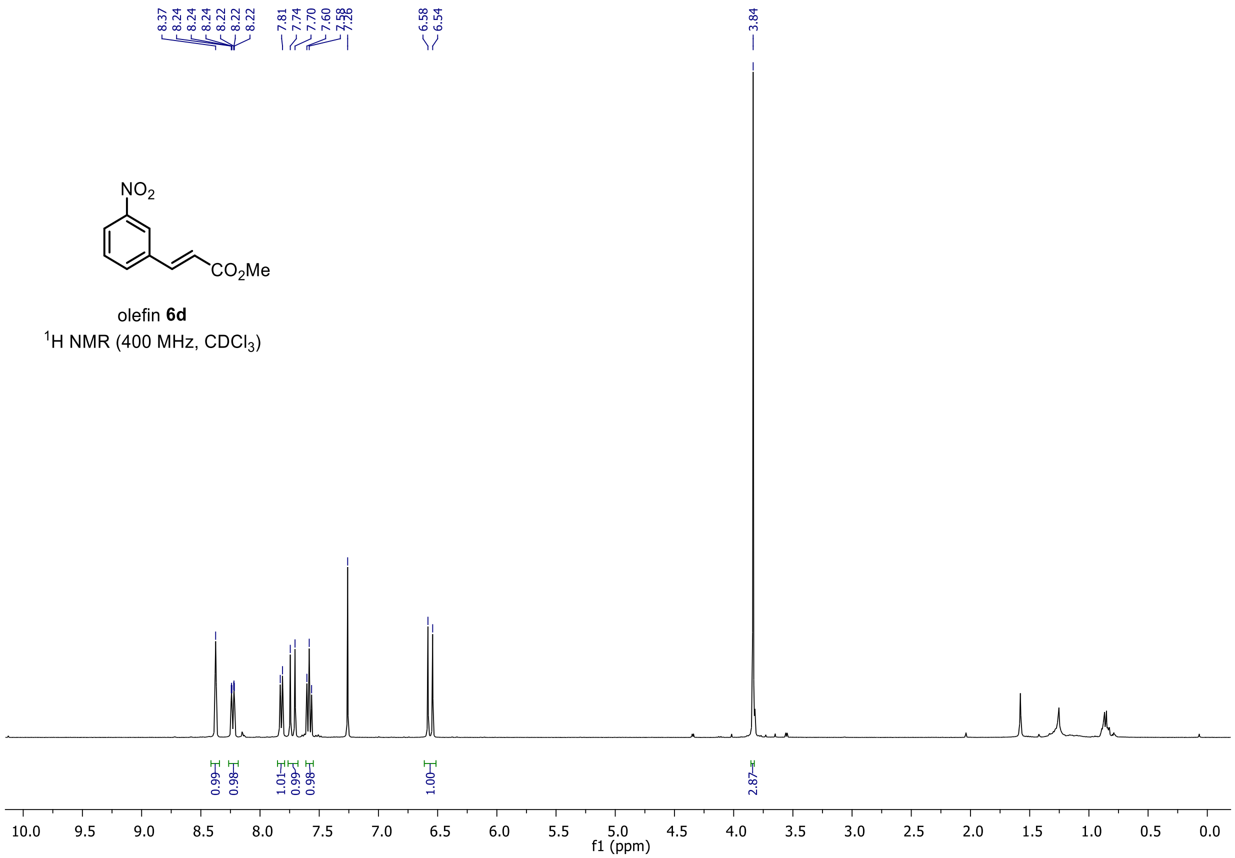
8.37
8.24
8.24
8.24
8.22
8.22
8.22
7.81
7.74
7.70
7.60
7.58
7.26
6.58
6.54
3.84
NO2
CO2Me
olefin 6d
1H NMR (400 MHz, CDCl3)
0.99
0.98
1.01
0.99
0.98
1.00
2.87
10.0
9.5
9.0
8.5
8.0
7.5
7.0
6.5
6.0
5.5
5.0
4.5
4.0
3.5
3.0
2.5
2.0
1.5
1.0
0.5
0.0
f1 (ppm)

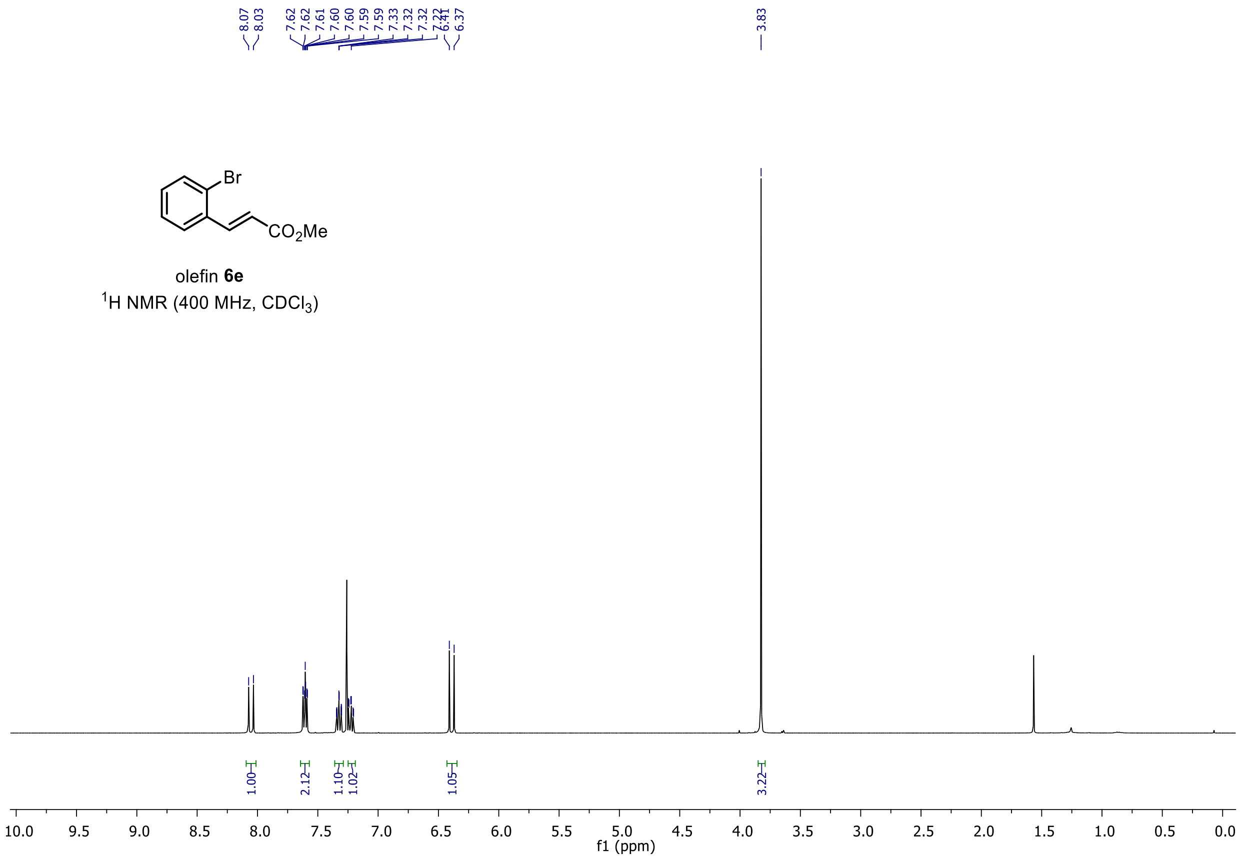
8.07
8.03
7.62
7.62
7.61
7.60
7.60
7.59
7.59
7.33
7.32
7.32
7.22
6.41
6.37
3.83
Br
CO2Me
olefin 6e
1H NMR (400 MHz, CDCl3)
1.00
2.12
1.10
1.02
1.05
3.22
f1 (ppm)

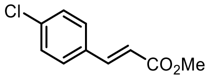

olefin **6f**

^1H NMR (400 MHz, $CDCl_3$)

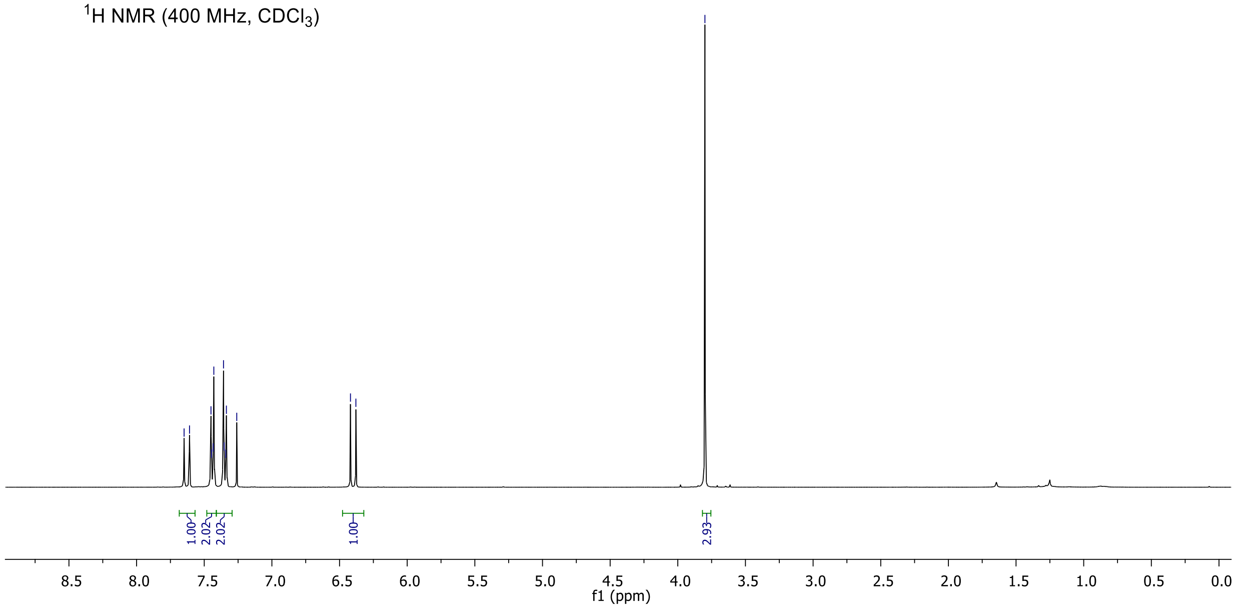

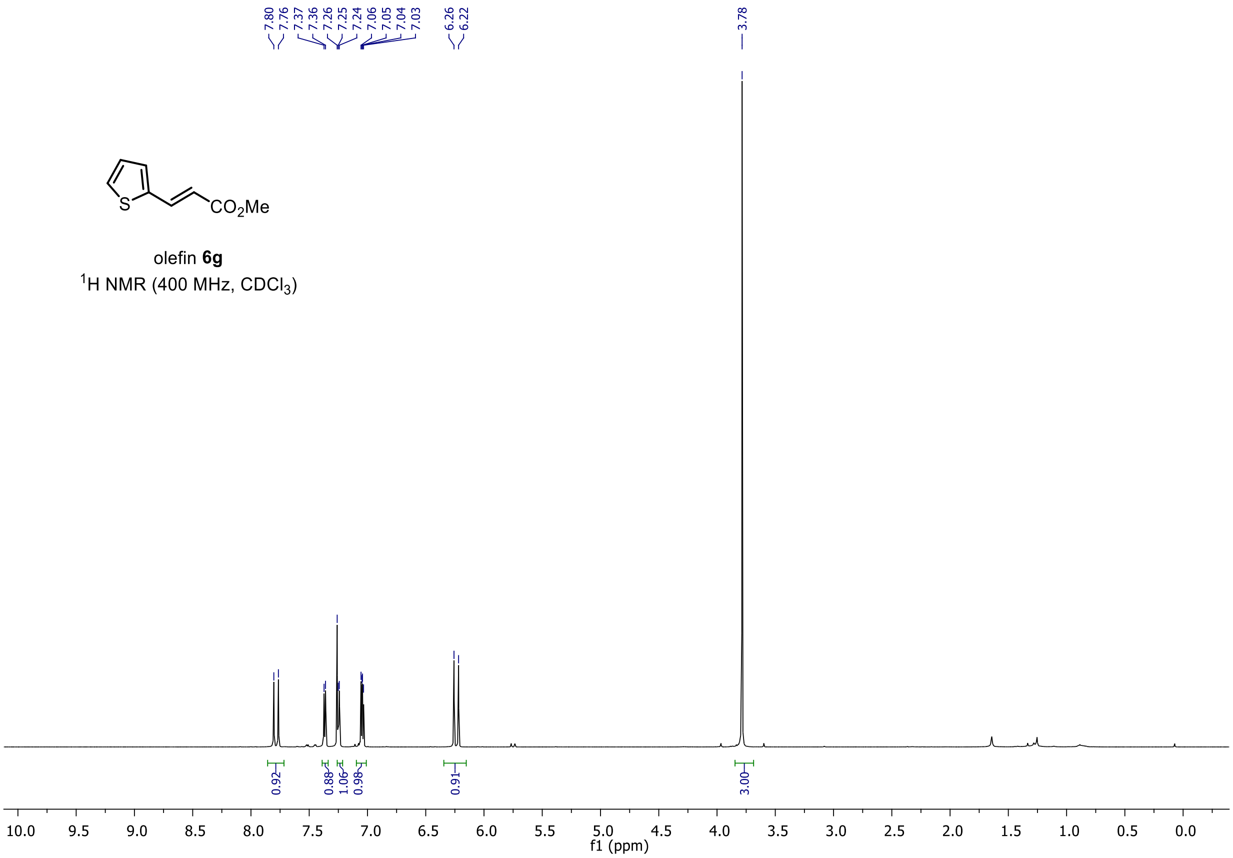

olefin **6g**
^{1}H NMR (400 MHz, $CDCl_3$)
7.80
7.76
7.37
7.36
7.26
7.25
7.24
7.06
7.05
7.04
7.03
6.26
6.22
3.78
0.92
0.88
1.06
0.98
0.91
3.00
10.0 9.5 9.0 8.5 8.0 7.5 7.0 6.5 6.0 5.5 5.0 4.5 4.0 3.5 3.0 2.5 2.0 1.5 1.0 0.5 0.0
f1 (ppm)

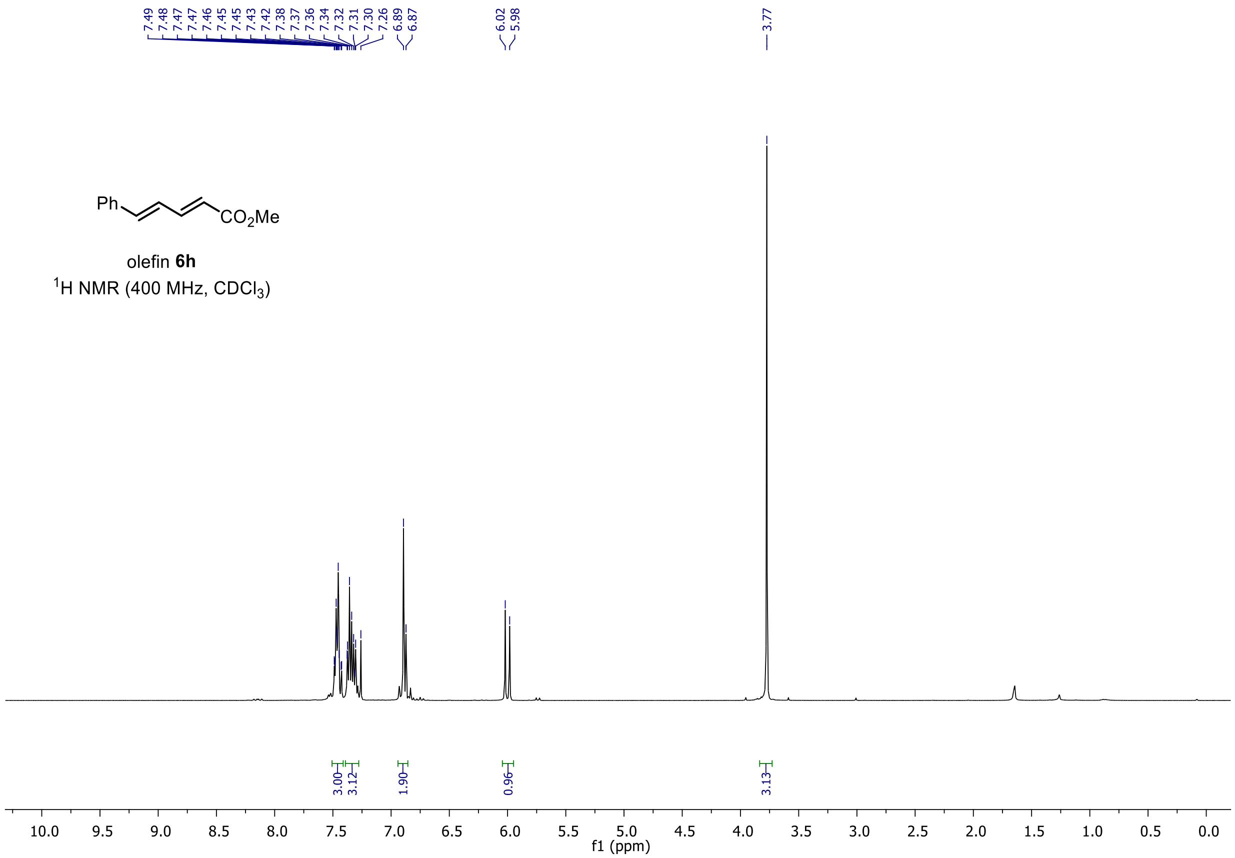
7.49
7.48
7.47
7.47
7.46
7.45
7.45
7.43
7.42
7.38
7.37
7.36
7.34
7.32
7.31
7.30
7.26
6.89
6.87
6.02
5.98
3.77
Ph
CO2Me
olefin 6h
1H NMR (400 MHz, CDCl3)
3.00
3.12
1.90
0.96
3.13
10.0
9.5
9.0
8.5
8.0
7.5
7.0
6.5
6.0
5.5
5.0
4.5
4.0
3.5
3.0
2.5
2.0
1.5
1.0
0.5
0.0
f1 (ppm)

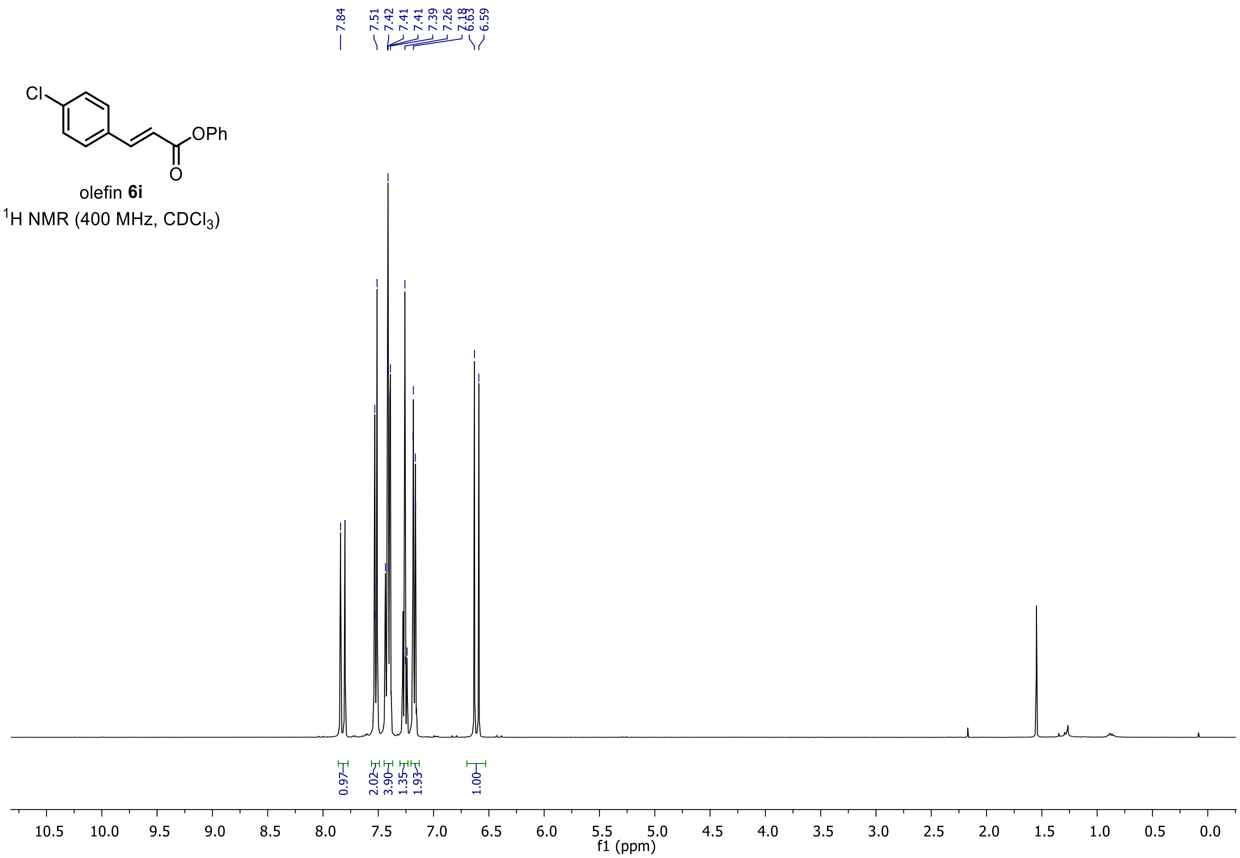

Cl
OPh
O
olefin 6i
1H NMR (400 MHz, CDCl3)
7.84
7.51
7.42
7.41
7.41
7.39
7.26
7.18
6.63
6.59
0.97
2.02
3.90
1.35
1.93
1.00
10.5
10.0
9.5
9.0
8.5
8.0
7.5
7.0
6.5
6.0
5.5
5.0
4.5
4.0
3.5
3.0
2.5
2.0
1.5
1.0
0.5
0.0
f1 (ppm)

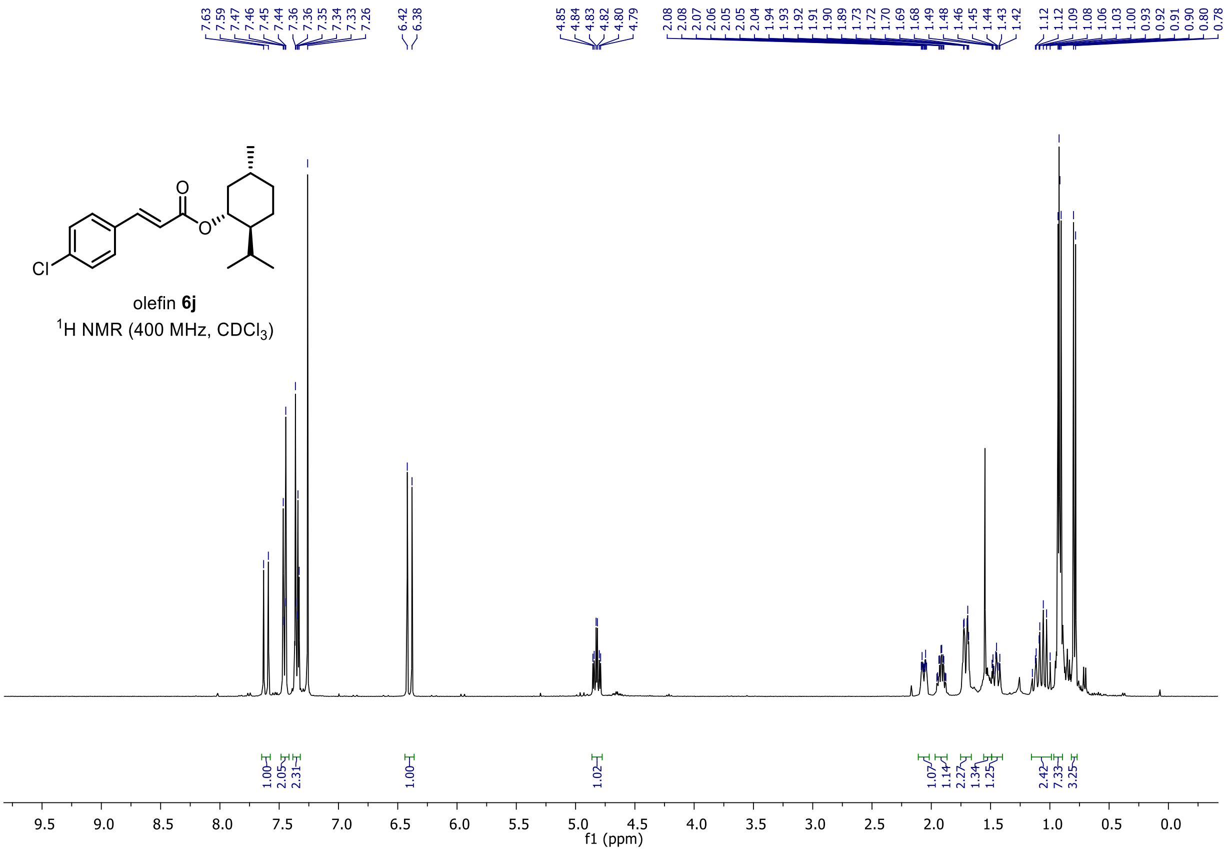
Cl
O
O
olefin 6j
1H NMR (400 MHz, CDCl3)
f1 (ppm)

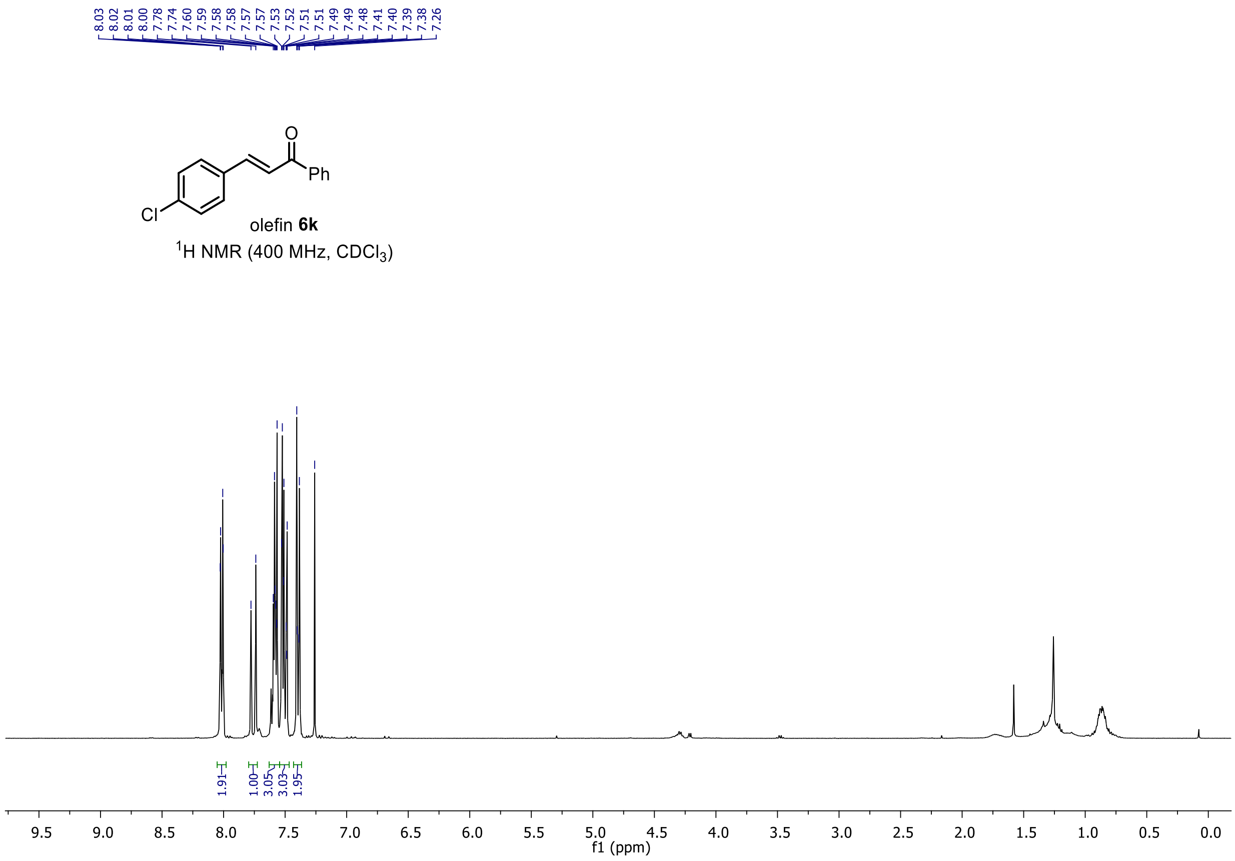

olefin **6k**

^{1}H NMR (400 MHz, $CDCl_3$)

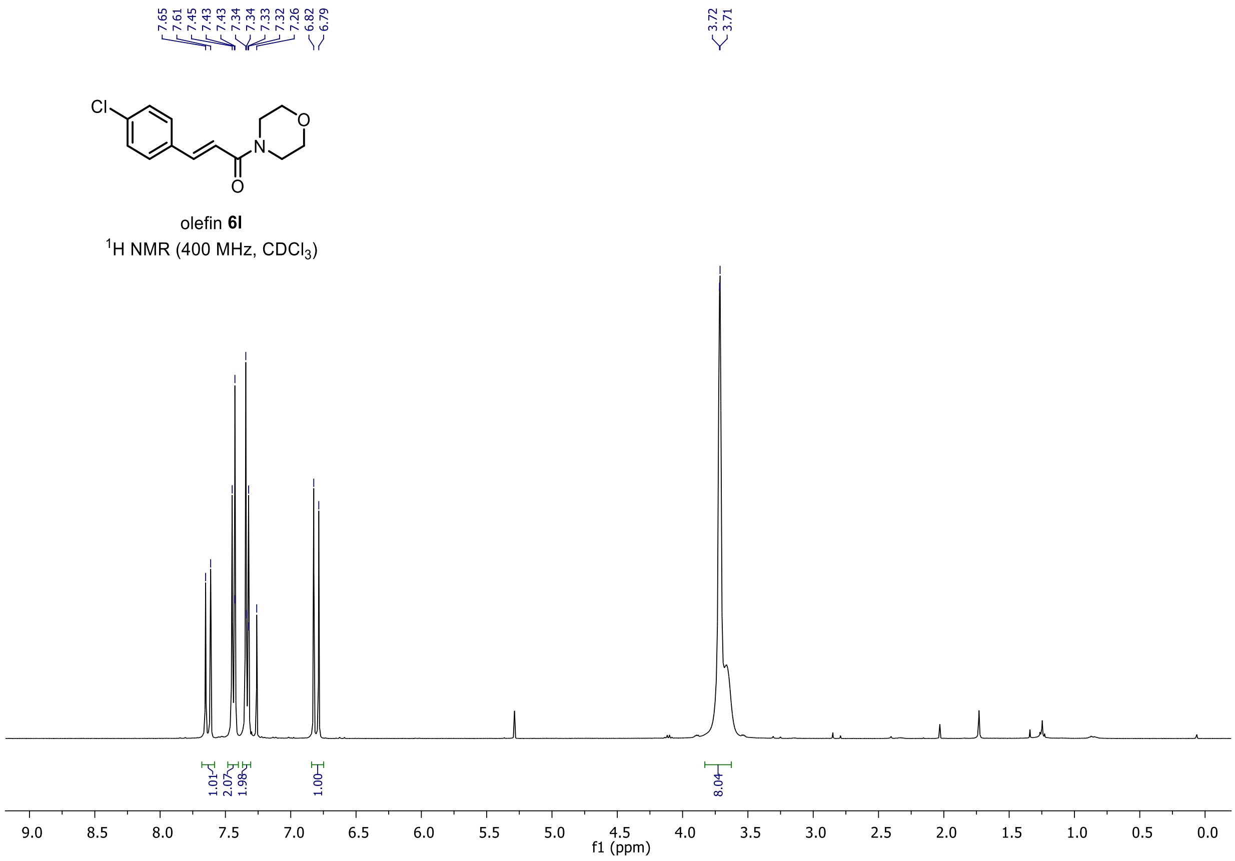
7.65
7.61
7.45
7.43
7.43
7.34
7.34
7.33
7.32
7.26
6.82
6.79
3.72
3.71
Cl
N
O
O
olefin **6l**
^{1}H NMR (400 MHz, $CDCl_3$)
1.01
2.07
1.98
1.00
8.04
9.0
8.5
8.0
7.5
7.0
6.5
6.0
5.5
5.0
4.5
4.0
3.5
3.0
2.5
2.0
1.5
1.0
0.5
0.0
f1 (ppm)

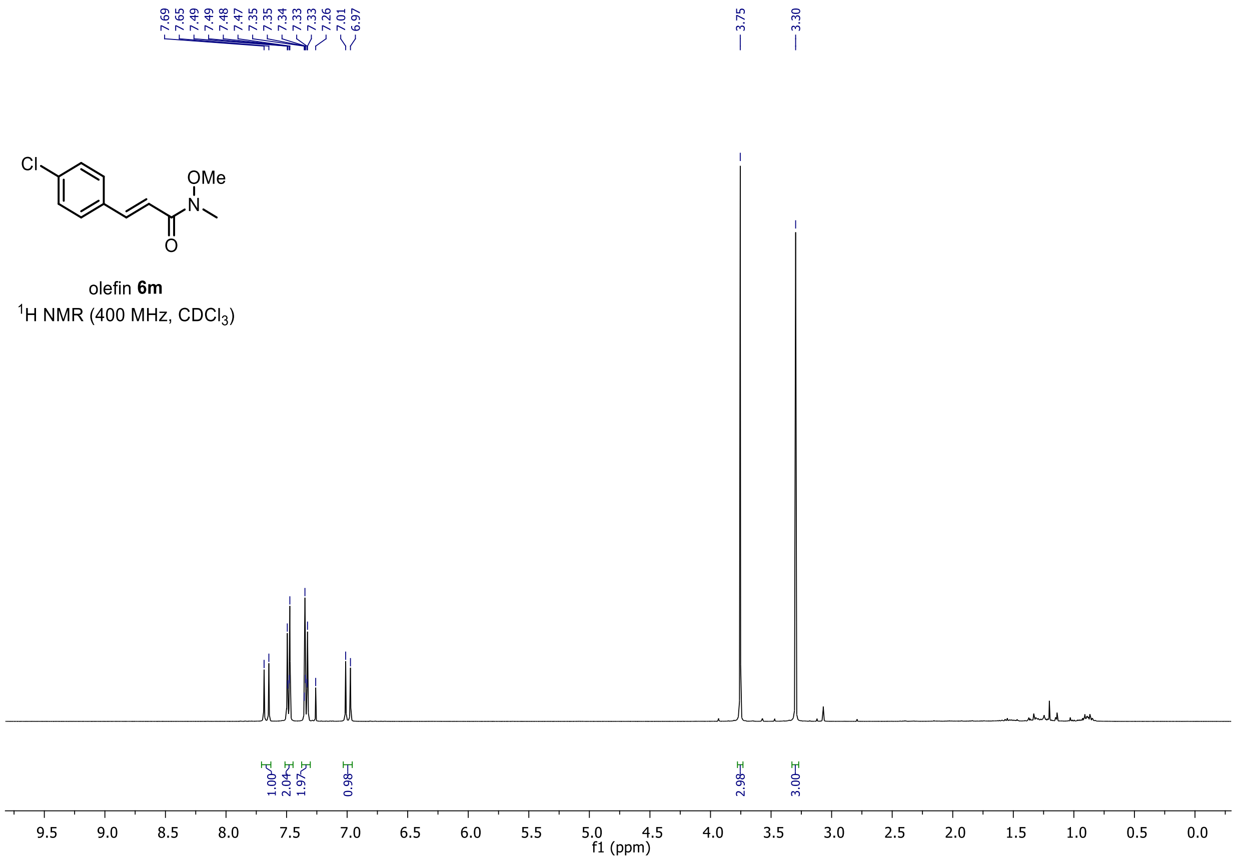

olefin **6m**

^{1}H NMR (400 MHz, $CDCl_3$)

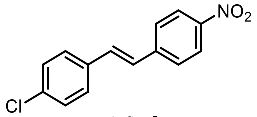

olefin **6n**

^{1}H NMR (400 MHz, $CDCl_3$)

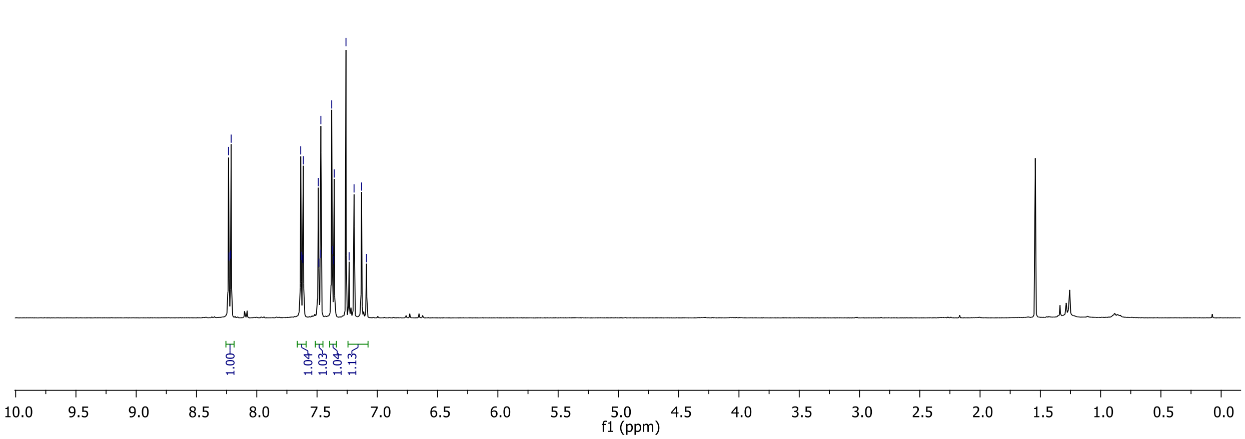

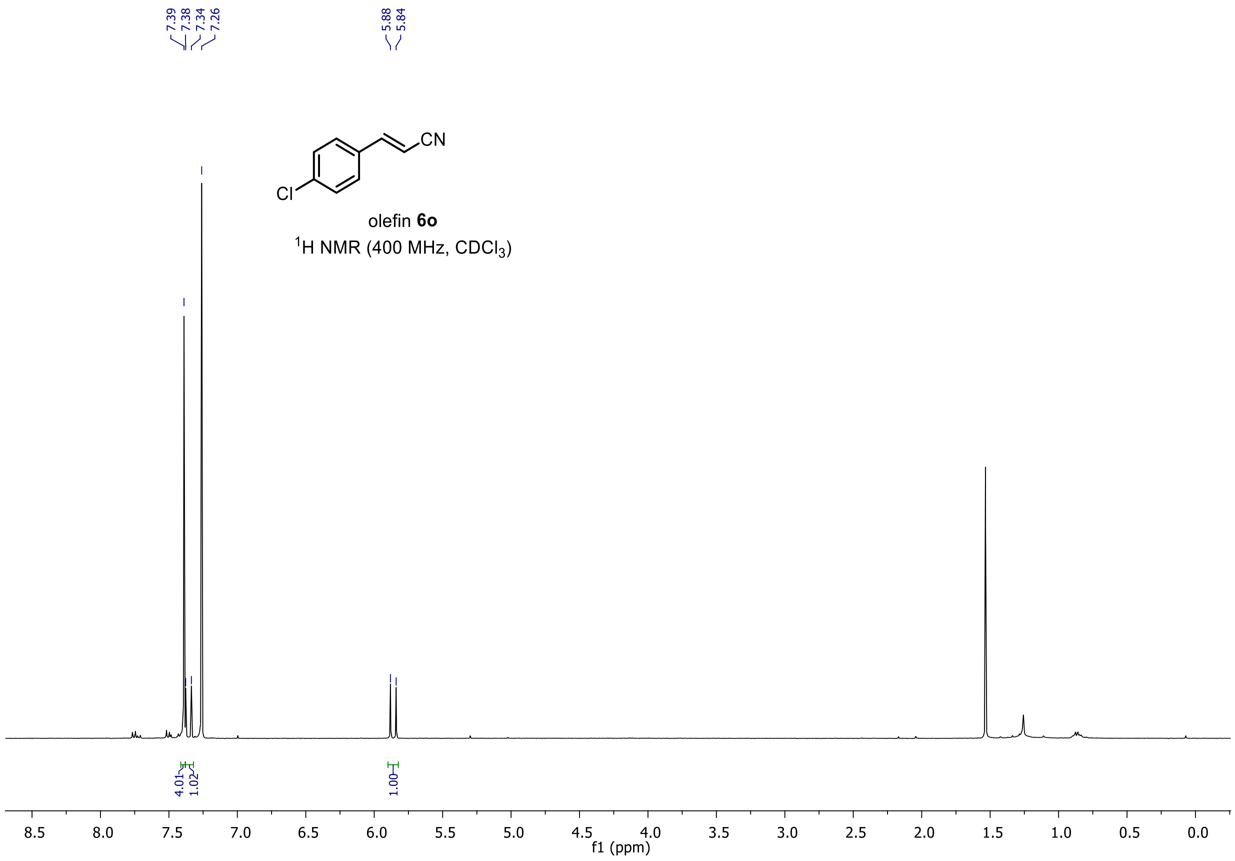

7.39
7.38
7.34
7.26
5.88
5.84
CN
Cl
olefin 6o
1H NMR (400 MHz, CDCl3)
4.01
1.02
1.00
8.5 8.0 7.5 7.0 6.5 6.0 5.5 5.0 4.5 4.0 3.5 3.0 2.5 2.0 1.5 1.0 0.5 0.0
f1 (ppm)

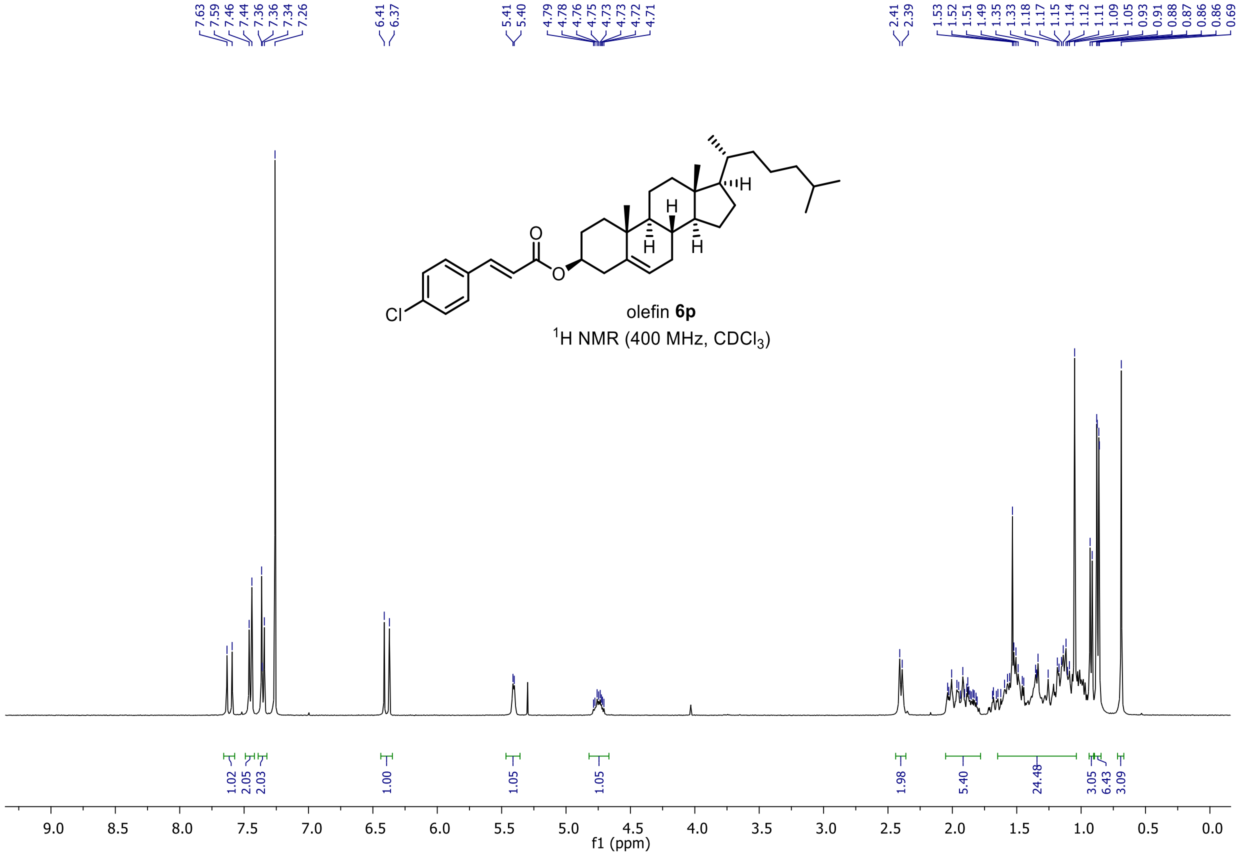
7.63
7.59
7.46
7.44
7.36
7.36
7.34
7.26
6.41
6.37
5.41
5.40
4.79
4.78
4.76
4.75
4.73
4.73
4.72
4.71
2.41
2.39
1.53
1.52
1.51
1.49
1.35
1.33
1.18
1.17
1.15
1.14
1.12
1.11
1.09
1.05
0.93
0.91
0.88
0.87
0.86
0.86
0.69
O
O
Cl
H
H
H
H
olefin **6p**
^{1}H NMR (400 MHz, $CDCl_3$)
1.02
2.05
2.03
1.00
1.05
1.05
1.98
5.40
24.48
3.05
6.43
3.09
9.0
8.5
8.0
7.5
7.0
6.5
6.0
5.5
5.0
4.5
4.0
3.5
3.0
2.5
2.0
1.5
1.0
0.5
0.0
f1 (ppm)

9. References

[1] E. M. Burgess, M. C. Pulcrano, *J. Am. Chem. Soc.* **1978**, *100*, 6538–6539.
[2] P. Pracht, F. Bohle, S. Grimme, *Phys. Chem. Chem. Phys.* **2020**, *22*, 7169–7192.
[3] S. Grimme, *J. Chem. Theory Comput.* **2019**, *15*, 2847–2862.
[4] J. P. Perdew, K. Burke, M. Ernzerhof, *Phys. Rev. Lett.* **1996**, *77*, 3865–3868.
[5] J. P. Perdew, K. Burke, M. Ernzerhof, *Phys. Rev. Lett.* **1997**, *78*, 1396–1396.
[6] C. Adamo, V. Barone, *J. Chem. Phys.* **1999**, *110*, 6158–6170.
[7] S. Grimme, J. Antony, S. Ehrlich, H. Krieg, *J. Chem. Phys.* **2010**, *132*, 154104.
[8] S. Grimme, S. Ehrlich, L. Goerigk, *J. Comput. Chem.* **2011**, *32*, 1456–1465.
[9] "Citation | Gaussian.com," can be found under https://gaussian.com/citation/, **n.d.**
[10] E. Cancès, B. Mennucci, J. Tomasi, *J. Chem. Phys.* **1997**, *107*, 3032–3041.
[11] A. V. Marenich, C. J. Cramer, D. G. Truhlar, *J. Phys. Chem. B* **2009**, *113*, 6378–6396.
[12] E. G. Hohenstein, C. D. Sherrill, *J. Chem. Phys.* **2010**, *132*, 184111.
[13] E. G. Hohenstein, R. M. Parrish, C. D. Sherrill, J. M. Turney, H. F. Schaefer, *J. Chem. Phys.* **2011**, *135*, 174107.
[14] E. Papajak, D. G. Truhlar, *J. Chem. Theory Comput.* **2011**, *7*, 10–18.
[15] D. E. Woon, T. H. Dunning, *J. Chem. Phys.* **1993**, *98*, 1358–1371.
[16] S. Kozuch, S. Shaik, *Acc. Chem. Res.* **2011**, *44*, 101–110.
[17] H. Cai, Y. Zhou, D. Zhang, J. Xu, H. Liu, *Chem. Commun.* **2014**, *50*, 14771–14774.
[18] X. Lu, U. Schneider, *Chem. Commun.* **2016**, *52*, 12980–12983.
[19] M. D. Delost, J. T. Njardarson, *Org. Lett.* **2021**, *23*, 6121–6125.
[20] S. Sauerbrey, P. K. Majhi, G. Schnakenburg, A. J. A. Iii, R. Streubel, *Dalton Trans.* **2012**, *41*, 5368–5376.
[21] Z. Huang, J.-P. Lumb, *Nat. Chem.* **2021**, *13*, 24–32.
[22] D. A. Klumpp, R. M. Sobel, S. G. Kokkinidou, B. Osei-Badu, Z. Liveris, R. A. Klumpp, M. R. Stentzel, *ACS Omega* **2020**, *5*, 4043–4049.
[23] L. Roiser, M. Waser, *Org. Lett.* **2017**, *19*, 2338–2341.
[24] X. Zhu, Y. Lin, Y. Sun, M. C. Beard, Y. Yan, *J. Am. Chem. Soc.* **2019**, *141*, 733–738.
[25] J. D. Neuhaus, P. Angyal, R. Oost, N. Maulide, *J. Org. Chem.* **2018**, *83*, 2479–2485.
[26] F. P. Touchard, *Tetrahedron Lett.* **2004**, *45*, 5519–5523.
[27] F. Seifert, D. Drikermann, J. Steinmetzer, Y. Zi, S. Kupfer, I. Vilotijevic, *Org. Biomol. Chem.* **2021**, *19*, 6092–6097.
[28] T. Ghosh, H. Hart, *J. Org. Chem.* **1989**, *54*, 5073–5085.
[29] P. L. Minin, J. C. Walton, *Org. Biomol. Chem.* **2004**, *2*, 2471–2475.
[30] W. C. Still, C. Gennari, *Tetrahedron Lett.* **1983**, *24*, 4405–4408.
[31] E. N. Jacobsen, L. Deng, Y. Furukawa, L. E. Martínez, *Tetrahedron* **1994**, *50*, 4323–4334.
[32] I. Janicki, P. Kiełbasiński, *Synthesis* **2022**, *54*, 378–382.
[33] S. Sano, K. Yokoyama, M. Fukushima, T. Yagi, Y. Nagao, *Chem. Commun.* **1997**, 559–560.
[34] A. K. Ghosh, S. Leshchenko, M. Noetzel, *J. Org. Chem.* **2004**, *69*, 7822–7829.
[35] T. Gatzenmeier, P. S. J. Kaib, J. B. Lingnau, R. Goddard, B. List, *Angew. Chem. Int. Ed.* **2018**, *57*, 2464–2468.
[36] K. D. Reichl, N. L. Dunn, N. J. Fastuca, A. T. Radosevich, *J. Am. Chem. Soc.* **2015**, *137*, 5292–5295.
[37] S. W. Youn, B. S. Kim, A. R. Jagdale, *J. Am. Chem. Soc.* **2012**, *134*, 11308–11311.
[38] L. Li, J. C. Stimac, L. M. Geary, *Tetrahedron Lett.* **2017**, *58*, 1379–1381.
[39] C. E. Gregerson, K. N. Trentadue, E. J. T. Phipps, J. K. Kirsch, K. M. Reed, G. D. Dyke, J. H. Jansen, C. B. Otteman, J. L. Stachowski, J. B. Johnson, *Org. Biomol. Chem.* **2017**, *15*, 5944–5948.
[40] F. L. Callonnec, E. Fouquet, F.-X. Felpin, *Org. Lett.* **2011**, *13*, 2646–2649.
[41] V. Ortega, E. del Castillo, A. G. Csákÿ, *Org. Lett.* **2017**, *19*, 6236–6239.
[42] *Eur. J. Org. Chem.* **2017**, *2017*, 928–932.
[43] J. McNulty, R. Vemula, V. Krishnamoorthy, A. Robertson, *Tetrahedron* **2012**, *68*, 5415–5421.
[44] C. Hayashi, T. Hayashi, T. Yamada, *Bull. Chem. Soc. Jpn.* **2015**, *88*, 862–870.
[45] S. Frattini, M. Quai, E. Cereda, *Tetrahedron Lett.* **2001**, *42*, 6827–6829.
[46] S. Dhara, C. E. Diesendruck, *Eur. J. Org. Chem.* **2017**, *2017*, 1184–1190.
